# Elucidation of novel compounds and epitope-based peptide vaccine design against C30 endopeptidase regions of SARS-CoV-2 using immunoinformatics approaches

**DOI:** 10.3389/fcimb.2023.1134802

**Published:** 2023-05-24

**Authors:** Saigha Marriam, Muhammad Sher Afghan, Mazhar Nadeem, Muhammad Sajid, Muhammad Ahsan, Abdul Basit, Muhammad Wajid, Sabeen Sabri, Muhammad Sajid, Imran Zafar, Summya Rashid, Sheikh Arslan Sehgal, Dalal Hussien M. Alkhalifah, Wael N. Hozzein, Kow-Tong Chen, Rohit Sharma

**Affiliations:** ^1^ Department of Microbiology and Molecular Genetics, Faculty of Life Sciences, University of Okara, Okara, Pakistan; ^2^ Department of Ear, Nose, and Throat (ENT), District Headquarter (DHQ) Teaching Hospital Faisalabad, Faisalabad, Punjab, Pakistan; ^3^ Department of Biotechnology, Faculty of Life Sciences, University of Okara, Okara, Pakistan; ^4^ Institute of Environmental and Agricultural Sciences, University of Okara, Okara, Pakistan; ^5^ Department of Microbiology, University of Jhang, Jhang, Pakistan; ^6^ Department of Zoology, Faculty of Life Sciences, University of Okara, Okara, Pakistan; ^7^ Department of Bioinformatics and Computational Biology, Virtual University, Punjab, Pakistan; ^8^ Department of Pharmacology and Toxicology, College of Pharmacy, Prince Sattam Bin Abdulaziz University, Al-Kharj, Saudi Arabia; ^9^ Department of Bioinformatics, Faculty of Life Sciences, University of Okara, Okara, Pakistan; ^10^ Department of Bioinformatics, Institute of Biochemistry, Biotechnology and Bioinformatics, The Islamia University of Bahawalpur, Bahawalpur, Pakistan; ^11^ Department of Biology, College of Science, Princess Nourah Bint Abdulrahman University, Riyadh, Saudi Arabia; ^12^ Botany and Microbiology Department, Faculty of Science, Beni-Suef University, Beni-Suef, Egypt; ^13^ Department of Occupational Medicine, Tainan Municipal Hospital (managed by ShowChwan Medical Care Corporation), Tainan, Taiwan; ^14^ Department of Public Health, College of Medicine, National Cheng Kung University, Tainan, Taiwan; ^15^ Department of Rasa Shastra and Bhaishajya Kalpana, Faculty of Ayurveda, Institute of Medical Sciences, Banaras Hindu University, Varanasi, India

**Keywords:** SARS-CoV-2, 3CL pro, CTL epitopes, immunoinformatics, peptide-based vaccine, computational immunology, C30 endopeptidase

## Abstract

There has been progressive improvement in immunoinformatics approaches for epitope-based peptide design. Computational-based immune-informatics approaches were applied to identify the epitopes of SARS-CoV-2 to develop vaccines. The accessibility of the SARS-CoV-2 protein surface was analyzed, and hexa-peptide sequences (KTPKYK) were observed having a maximum score of 8.254, located between amino acids 97 and 102, whereas the FSVLAC at amino acids 112 to 117 showed the lowest score of 0.114. The surface flexibility of the target protein ranged from 0.864 to 1.099 having amino acid ranges of 159 to 165 and 118 to 124, respectively, harboring the FCYMHHM and YNGSPSG hepta-peptide sequences. The surface flexibility was predicted, and a 0.864 score was observed from amino acids 159 to 165 with the hepta-peptide (FCYMHHM) sequence. Moreover, the highest score of 1.099 was observed between amino acids 118 and 124 against YNGSPSG. B-cell epitopes and cytotoxic T-lymphocyte (CTL) epitopes were also identified against SARS-CoV-2. In molecular docking analyses, -0.54 to -26.21 kcal/mol global energy was observed against the selected CTL epitopes, exhibiting binding solid energies of -3.33 to -26.36 kcal/mol. Based on optimization, eight epitopes (SEDMLNPNY, GSVGFNIDY, LLEDEFTPF, DYDCVSFCY, GTDLEGNFY, QTFSVLACY, TVNVLAWLY, and TANPKTPKY) showed reliable findings. The study calculated the associated HLA alleles with MHC-I and MHC-II and found that MHC-I epitopes had higher population coverage (0.9019% and 0.5639%) than MHC-II epitopes, which ranged from 58.49% to 34.71% in Italy and China, respectively. The CTL epitopes were docked with antigenic sites and analyzed with MHC-I HLA protein. In addition, virtual screening was conducted using the ZINC database library, which contained 3,447 compounds. The 10 top-ranked scrutinized molecules (ZINC222731806, ZINC077293241, ZINC014880001, ZINC003830427, ZINC030731133, ZINC003932831, ZINC003816514, ZINC004245650, ZINC000057255, and ZINC011592639) exhibited the least binding energy (-8.8 to -7.5 kcal/mol). The molecular dynamics (MD) and immune simulation data suggest that these epitopes could be used to design an effective SARS-CoV-2 vaccine in the form of a peptide-based vaccine. Our identified CTL epitopes have the potential to inhibit SARS-CoV-2 replication.

## Graphical Abstract

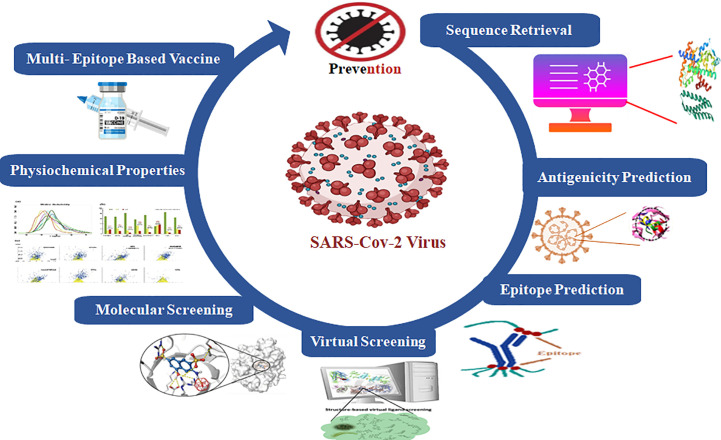

## Introduction

A newly discovered human coronavirus (HCoV) becomes the deadliest pandemic of the 21st century. On December 31st, in Wuhan, China, numerous patients have been identified as asymptomatic carriers of the Severe Acute Respiratory Syndrome-2 (SARS-CoV-2) virus (Agarwal et al., 2022; Rayan et al., 2022). Comparative molecular analyses of SARS-CoV-2 against SARS-CoV-1, SARS-CoV-2, MERS-CoV, and bat-CoV showed 80% to 95% similarity (Ahmad et al., 2022). The World Health Organization (WHO) has declared SARS-CoV-2 disease as COVID-19 (Kannan et al., 2020). SARS-CoV-2 has impacted individuals from diverse nations including Thailand, Japan, and South Korea, who had not previously travelled to the epicenter of the outbreak in Wuhan, China. Based on these outcomes, the researcher emphasizes that diagnosed individuals contracted the virus through anthropogenic actions (Bastola et al., 2020; Huang et al., 2020).

The genomic determinants of pathogenicity and computational analyses of MERS and SARS coronaviruses highlighted the importance of SARS-CoV-2 as a causative agent (Fehr et al., 2017). In January 2020, the Chinese institutes submitted SARS-CoV-2 sequences for conformations and sequence homology targets and predicted a vast reality by using bioinformatics approaches (Riva et al., 2020). In China, researchers emphasized the implications of the novel mutations and mutational diversification of SARS‐CoV‐2 from rapid genome sequencing of ∼30,000 nucleotides (Beniac et al., 2006). Comparative sequence analyses and whole-genome phylogenetic guidelines showed that SARS-CoV-2 has 80% similarity with previously known SARS-CoV (Waqas et al., 2020).

SARS-CoV-2 viral proteins interact with various human proteins, including regulatory proteins (Chen et al., 2021). Specifically, the Nsp1, Nsp5, Nsp8, Nsp13, E, S, ORF3a, ORF8, M, ORF9b, N, and Nsp15 proteins have been identified as affecting critical cellular processes (Li et al., 2021), such as posttranscriptional and epigenetic regulation, epithelial trafficking, lipid changes, and RNA translation and transcription. Additionally, the Nsp1, Nsp4, Nsp8, Nsp9, Nsp13, Nsp15, ORF6, ORF9C, S, E, and ORF proteins also disturb the cytoskeleton, mitochondria, and extracellular matrix (O’Meara et al., 2020). To overcome the SARS-CoV-2, current therapeutic strategies focus to inhibit the 3-chymotrypsin-like protease of SARS-CoV-2. However, there is a critical need to develop novel antiviral drugs and vaccines that specifically target the Nsp, S, E, and ORF proteins to prevent viral replication and proliferation.

**Figure 1:**
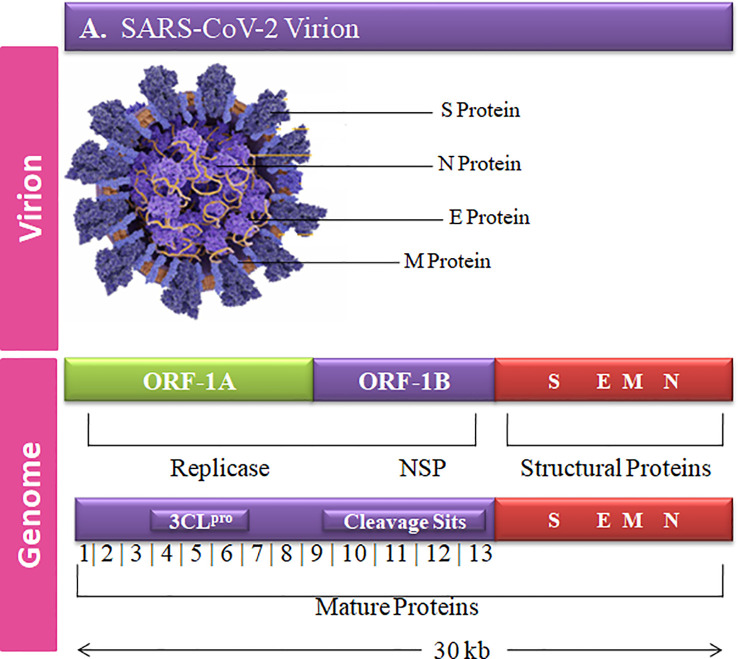
Graphical representation of the SARS-CoV-2 genetic and structural composition. The structural proteins of the SARS-CoV-2 play vital roles in viral replication, amplification, and RNA synthesis. Spike protein (S), envelope protein (E), and membrane protein (M) are enclosed in the lipid bilayer. The nucleocapsid (N) protein interacts with the single-stranded positive-sense viral RNA. The translation of the first two ORFs leads to the formation of replication polyproteins 1a and 1b.

The complete genome sequence of HCoV is more complex than other viruses with ~30-kb size and a unique club-shaped spike morphology (**Figure 1**). The coronavirus (CoV) genome has significant loops to provide genetic evidence at the beginning of the untranslated region (UTR region). The 3′ end of poly-A contain a ladder sequence with unique structures required for genome synthesis and replication and reveal an additional 5′ capped end ladder sequence (Zhao et al., 2012). The genomic variation of the human SARS-CoV-2 viral genome comprises 14 open reading frames (ORFs) and 29,891 nucleotides encoding for 9,860 amino acids (Guan et al., 2020). The cross-functional analyses of CoV assessed to evolve 5′ terminal contain 1–16 non-structural protein (nsp) and 2 key ORFs (ORF1a and ORF1b) translated into polyproteins (pp) 1a (pp1a; nsp 1–11) and 1ab (pp1ab; nsp1–16) (Lu et al., 2020).

Non-structural proteins (nsp) are released from the multimeric complex during viral transcription and replication by cysteine proteases (Oanca et al., 2020). The conserved 3-chymotrypsin-like protease (3CL^pro^), also known as the main protease (M-pro), originates in the polyprotein ORF1ab of SARS-CoV-2 and found within Nsp5. In contrast, the papain-like protease (PLpro) is found inside nsp3 (Chen et al., 2020). 3CL^pro^ is a crucial SARS-CoV-2 receptor that regulates the transcription and viral replication (Jin et al., 2020). 3CL^pro^ cuts the pp at 11 diverse regions to produce numerous parts for viral replication. Proteinases are required for proteolytic processing degradation, and they inhibit the host interpretation by interacting the 40S subunits’ extended head shape (Way, 2021). The C-terminus attaches to prevent the ribosomal mRNA entry tunnels from forming (Schubert et al., 2020). As a result, it suppresses the antiviral activity induced by adaptive immunity. The nsp1–40S ribosome complex also causes endonuclease breaking at the 5′UTR of host mRNAs, resulting in their elimination based on similarities. Viral mRNAs are less vulnerable to nsp1-mediated translational inhibition due to their 5′-end ladder sequence (Crow, 2021). 3CL^pro^ nsp5 causes a conformational shift at the C-terminus of the reverse transcription protein complex (Dai et al., 2020).

SARS-CoV-2 is more lethal than SARS and MERS, so precautionary measures have been taken against the novel SARS-CoV-2 disease globally (Douglas et al., 2018). COVID-19 prevention techniques mostly rely on peptide-based vaccines, and researchers have tried to develop a variety of vaccines against SARS-CoV-2 (Tahir ul Qamar et al., 2020). Bioinformatics techniques, virtual screening, molecular docking, and bioactive compounds have been utilized to inhibit SARS-CoV-2 (Xiao et al., 2021). B-cell and conservation of cytotoxic T lymphocyte (CTL) epitopes are time consuming (Ip et al., 2015). The identification of protein specific peptides which can bind to the major histocompatibility complex (MHC) is a critical step in peptide-based vaccine design. The peptide and MHC molecule binding depends at the association of T-cell immunogenicity (Lazarski et al., 2005). The peptide-based vaccines can activate specific immune responses accurately (Purcell et al., 2007).

The present study was aimed to employ advanced computational analyses and immunoinformatics methodologies to design epitope-based vaccine and to scrutinize novel compounds against SARS-CoV-2. The primary objective was to investigate the linear and conformational B-cell and T-cell potential antigens of 3CLpro. This study was performed to identify peptide-based vaccines against SARS-CoV-2. Through elucidating the current findings, the researchers aspire to make a significant and valuable contribution toward developing a workable vaccine.

## Materials and methods

The genomic and proteomic data of SARS-CoV-2 were retrieved from publicly accessible databases including GISAID, NCBI, GenBank, and UniProtKB. 3CLpro was selected and plays a critical role in transcription and replication processes of SARS-CoV2 and has the potential to be an extremely effective therapeutic target.

### Sequence retrieval and alignment

UniProtKB was utilized to retrieve the amino acid sequence of 3CL^pro^ having accession number P0DTD1 ( , ). The specific section of non-structural 3CL^pro^ was selected consisting of 306 amino acids, whereas the entire P0DTD1·R1AB_SARS2 has a total number of 7,096 amino acids. The 3D structure of the selected protein was determined by X-ray crystallography (PDB ID: 6W63) and was retrieved from PDB to visualize the atomic level of the selected structure. Additionally, ProtParam was utilized to examine the physiochemical properties of 3CL^pro^ and the data were extracted from SWISS-PROT and TrEMBL (O'Donovan et al., 2002).

Comparative genomic analyses of SARS-CoV (NC 004718), MERS-CoV (NC 019843.3), and SARS-CoV-2 (NC 045512.2) were performed by applying the multiple-sequence alignment (MSA) approach. The genomic sequences of the selected viral strains were retrieved from NCBI GenBank (Benson et al., 2018) and GISAID databases (Shu and McCauley, 2017). Clustal Omega (Sievers and Higgins, 2014) was employed to perform MSA. WebLogo3 (https://weblogo.threeplusone.com/) was used to visualize the conserved domain of the selected protein. MSA was cross verified by using pair-score matrices, including the Hidden Markov Model (HMM) (Blunsom, 2004) and OXBench (Raghava et al., 2003). MacVector (Rastogi, 1999) sequence application was used to examine the selected genomes and translated proteins.

### *In silico* prediction of linear and conformational B- and T-cell epitope prediction

Linear B-cell epitope peptides are potential candidates for antigens in vaccine design and immunological regulation. The immune epitope database and analysis resource (IEDB) (Fleri et al., 2016) was applied to conduct *in silico* analyses followed by Karplus and Schulz’s flexibility prediction and the Kolaskar antigenicity scale (Alexander et al., 2011). The surface accessibility predictions were also calculated by using Emini and Parker’s and Hopp and Wood’s hydrophilicity prediction methods (Parker et al., 1986). ElliPro was utilized to predict the B-cell epitope conformation ranges from 0.5 to 6Å. To ensure the reliability of epitope prediction within 95.5% and 99.5%, a cut-off value of 0.85 was used. The protrusion index (pI), adjacent residues, and protein shape approximation algorithms were employed for further analyses (Nain et al., 2020). The 3CL^pro^ 3D structure was visualized through PyMOL and UCSC Chimera visualization tools. Moreover, the anticipated epitopes as spheres to determine the surface accessibility of potential peptides were removed.

### Prediction of CTL epitopes

The NetCTL 1.2 server (https://services.healthtech.dtu.dk/service.php?NetCTL-1.2) was used to predict the cytotoxic T-lymphocyte (CTL) epitopes. CTLs are immune cells that recognize and kill the infected cells by binding to short peptide fragments (epitopes) presented by MHC molecules on the cell surface. A combination of algorithms was utilized to evaluate the MHC-I binding affinity, transporter associated with antigen processing (TAP), transport efficiency (with a threshold of 0.05), and proteasomal C-terminal cleavage (with a threshold of 0.15), at an epitope identification threshold of 0.75 (Larsen et al., 2007). The amino acid sequences of the target protein were submitted to the NetCTL 1.2 server in FASTA format to predict the peptide lengths and human leukocyte antigen (HLA) alleles. To predict TAP utilization, weight matrix, T-cell epitope prediction, and artificial neural network approaches, proteasomal C-terminal cleavage and MHC class-I binding were used.

### Prediction of antigenicity

In order to design an effective antigen construct, it is important to identify potential epitopes with high antigenicity. Initially, the B-cell epitopes were screened and MHC-I and II epitopes were subsequently determined by using ProPred (http://crdd.osdd.net/raghava/propred/, accessed on 10 April 2023) and ProPred 1 (http://crdd.osdd.net/raghava/propred1/, accessed on 10 April 2023) servers. The VaxiJen v2.0 server was used to evaluate the antigenicity of the predicted MHC-I and II epitopes having a threshold value of 0.5. The alignment-independent prediction method was utilized to identify the possible epitopes of 3CL^pro^. The virus cell line was selected as the target organism for vaccine development. The utilized methodology helped to identify the potential epitopes with high antigenicity, which can be incorporated into an optimized antigen construct for the development of a vaccine against SARS-CoV-2.

### An epidemiological strategic and world population coverage analyses

The world population coverage assessments were conducted by using the IEDB server (https://www.iedb.org/) and CTL epitopes against the relevant allele sets to determine whether the chosen candidates were suitable for coverage. Major population coverage evaluations were conducted on China, Japan, Iran, and Korea (Vita et al., 2019).

### Molecular docking and bioinformatics analysis for the peptide–MHC protein complex

SARS-CoV-2-predicted CTL epitope peptides and virulent residues were selected for the molecular docking analyses. For 100 simulation runs, the PEP-FOLD3 server was used to forecast the ideal model configurations and to simulate the 3D structure of selected peptides (Lamiable et al., 2016) and were assessed by SOPEP energy scores (Maupetit et al., 2007). For molecular docking analyses, the score peptides were selected by PatchDock with a clustering RMSD value of 4. Furthermore, the unwanted docked complexes with receptor atom penetrations into ligands were removed (Schneidman-Duhovny et al., 2005). FireDock was used to cross verify and screen the suitable docked complexes (Mashiach et al., 2008). The fast rigid-body docking with clear flexibility and scoring issues were used for docking calculations (Kingsford et al., 2005). The PyMOL (Alexander et al., 2011) and UCSF Chimera 1.15 (Pettersen et al., 2004)were employed to identify the docked complexes having hydrogen-bonding interactions.

### Structure-based molecular docking analyses of potential compounds

The library of 3,447 compounds (FDA, DrugBank approved) from the ZINC database was used for virtual screening through molecular docking analyses. All the compounds were minimized to get stable results through ChemDraw and UCSF Chimera. The molecular docking analyses was performed through AutoDock Vina (Dallakyan and Olson, 2015) and AutoDock Tools. The RMSD values were also calculated based on suitable hits. The admetSAR server (Shen et al., 2010) and ADMETlab 2.0 (Xiong et al., 2021) were used to calculate the drug-like physical and chemical properties of the selected compounds. BIOVIA Discovery studio (Sievers and Higgins, 2014) and Ligplot were used to analyze the resultant interacting residues (Wallace et al., 1995).

### Molecular dynamics simulation

Desmond software from Schrödinger LLC was used to perform the molecular dynamics (MD) simulation of the receptor and ligand complexes for 100 ns (Manandhar et al., 2022). To simulate atomic movements over time, MD simulations with Newton’s classical equation of motion was utilized. For the ligand binding status in physiological conditions, simulation predictions were generated. Maestro’s Protein Preparation Wizard was used to incorporate the complex optimization and minimization, and the receptor–ligand complex was preprocessed (Tabti et al., 2023). Using the OPLS_2005 force field and TIP3P orthorhombic box solvent model, the System Builder tool generated each system. The models were neutralized by using the counter ions. The physiological conditions were simulated by adding 0.15 M sodium chloride (NaCl). For the duration of the simulation, the NPT ensemble at 300 K temperature and 1 atm pressure was used. Before running the simulation, the models were unloaded and trajectories were saved every 100 ns to analyze the stability of the simulation analyses. By contrasting the root mean square deviation (RMSD) of the protein and ligand with time, stability was verified (Knapp et al., 2011; Rather et al., 2020). To assess the stability of the MD simulations, RMSD, radius of gyration (Rg), hydrogen bond number, and solvent accessible surface (SASA) were calculated.

### Immune simulation

To evaluate the immunogenicity and immune responses of *in silico* vaccine design, an agent-based immune simulation technique was utilized. The C-ImmSim webserver was used to simulate the molecular interactions between immunogenic molecules at a mesoscopic level (Rapin et al., 2010). The amino acid sequence of each vaccine was used to conduct the simulation process, and the machine learning method was used to design the epitope constructs for injection. The ability of the vaccine was predicted by using the C-ImmSim web server to induce the differentiation and proliferation of various immune cells. The default algorithm was used, and the refined 3-C-like protease was tested for its efficacy to induce an immune response. Three doses of the designed vaccine were administered through three injections at intervals of 28 days in the immune simulation experiment. The time steps were fixed at 1, 91, and 181, which were equal to 8 h of real-life time, and all other parameters were kept at their default values (Das et al., 2021).

## Results

A recent outbreak of a new type of viral pneumonia has emerged at an alarming pace, causing widespread concern and fear. Surprisingly, this pneumonia is distinct from other highly infectious and disease-causing viruses such as MERS, SARS, adenovirus, and influenza viruses. The implications of this outbreak are still being studied, and much research is being conducted to understand the nature and transmission (Lu et al., 2020). WHO identified the cause of the pneumonia outbreak as a new coronavirus and named it COVID-19. The virus quickly spread across borders and international travel, posing a significant threat to countries with inadequate healthcare systems. The global health emergency declaration was made to prevent the further spread of the disease, and various measures were taken to control its transmission, including social distancing, travel restrictions, and wearing masks. The scientific community also swiftly mobilized to develop vaccines and treatments to combat the deadly virus. Despite the challenges faced, the world has united to fight against COVID-19, highlighting the importance of global cooperation in addressing such health crises (W. H. Organization, 2019). The swift infection of host cells by the emerging virus, SARS-CoV-2, has created an air of uncertainty surrounding its final dimensions and impact (De Wilde et al., 2017). A surveillance system is required, and preventive measures should be taken to combat the rapidly increasing burden of SARS-CoV-2 infections globally.

Peptide-based vaccine mechanisms were extensively utilized to prevent the COVID-19 (Tahir ul Qamar et al., 2020). Immunoinformatics has played a significant role to predict the effective vaccines that lessen the manufacturing costs and consume less time. Developing an effective epitope-based peptide vaccine with the proper selection of immune-dominant epitopes and suitable antigen candidates is difficult. However, predicting the right epitopes of the target protein for designing epitope-based peptide vaccines *via* approaching immunoinformatics tools was necessary (Nain et al., 2020). The main target of immunoinformatics approaches is to predict the epitope-based peptide vaccines by recognizing 3CL^pro^. The pathogenic analyses help to discover the novel vaccines at the genomic level; however, these experimental tools have multiple limitations (Vilela Rodrigues et al., 2019).

Immunoinformatics approaches help *in vitro* expressions of the potential antigen, complete spectrum analysis, and observe pathogen culturing. Researchers have observed many vaccine candidates by using computational methods with promising preclinical outputs (Davies and Flower, 2007). CTL epitopes help to design peptide-based vaccines against human leukocyte antigen-B protein (Tahir et al., 2018). SARS-CoV-2 epitope-based vaccine development targets structural proteins of the virus and CTL epitopes of the selected protein. CTL epitopes support the host immune responses; moreover, the PBD ID 6W63-tagged non-structural protein of SARS-CoV-2 is linked to viral replication (Waqas et al., 2021).

The selected CTL epitopes or allergenicity and antigenicity were optimized (Dimitrov et al., 2014). In China, the predicted epitope population coverage analyses for MHC-I were reported to be 0.0373 with average hits of 0.3. Eight epitopes promising peptides were designed, and molecular docking analyses were performed to identify the effective binding sites (Huang et al., 2010).

### Surface accessibility analysis

**Figure 2:**
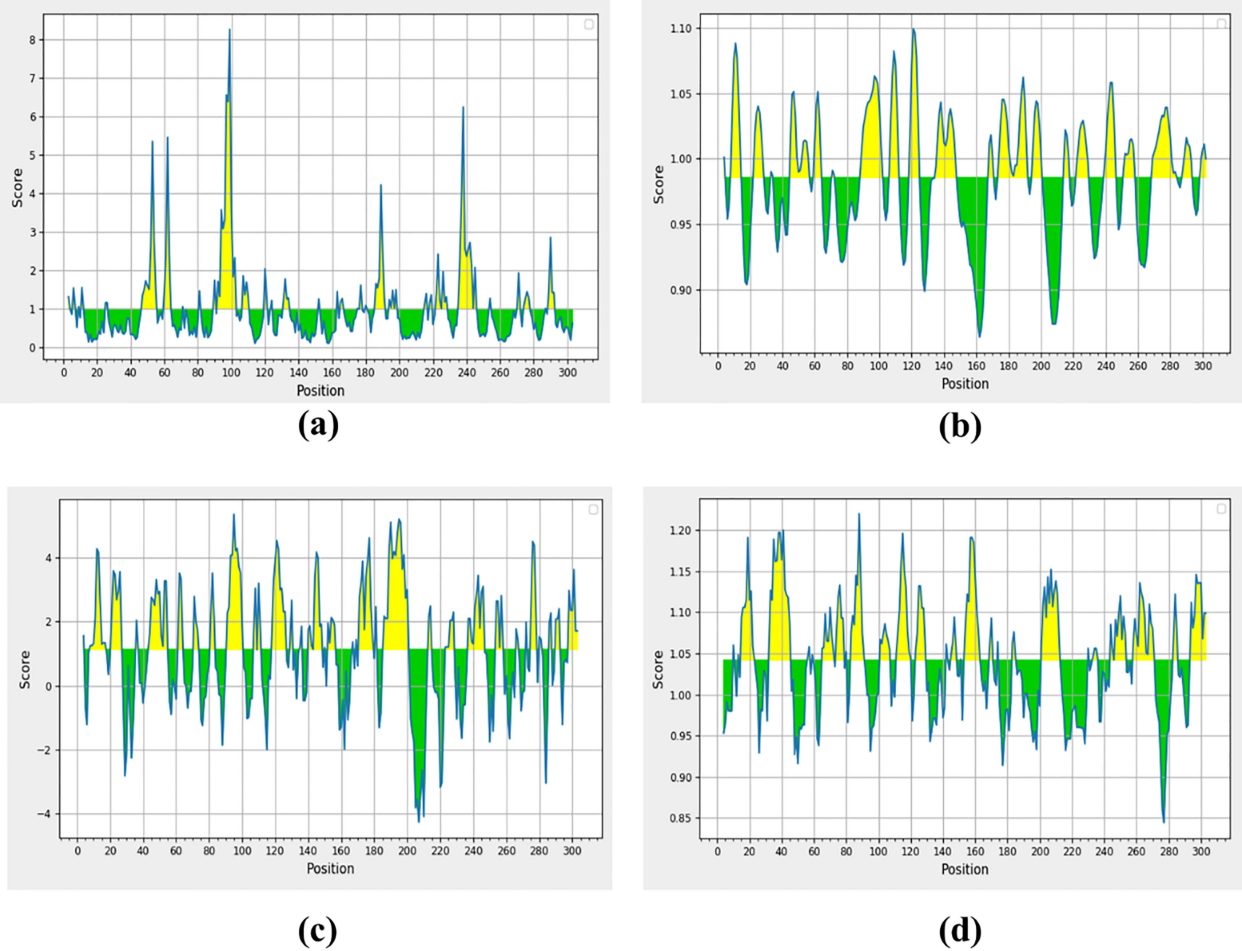
Non-structural protein (PDB: 6W63), **(A)** Parker’s predictions for hydrophilicity, **(B)** surface accessibility, **(C)** flexibility, and **(D)** antigenicity were evaluated. The all figure shows the results as scores, with the x-axis showing sequence positions and the y-axis showing probability values.

A peptide of >1.0 surface accessibility has more probability of being found on the surface (Parker et al., 1986). SARS-CoV-2 top-ranked predicted peptides among numerous peptides were selected for further analyses. The peptide surface probability and sequence position were represented by the y-axis and x-axis (**Figure 2A**). The maximum 8.254 scores of surface probability were observed in hexa-peptide sequence KTPKYK (97 to 102), and the lowest score of 0.114 was observed in hexa-peptide sequence FSVLAC (112 to 117) (**Supplementary S1, S1.1**).

### Surface flexibility prediction

The Schulz and Karplus flexibility method was utilized to calculate the atomic vibrational motions of the protein structure. The selection was made on temperature value and B-factor as the organization of the predicted structure, stability. The quality of the predicted structure was observed proportional to the B-factor. The lower range is more effective as compared to the higher range of the B-factor. SARS-CoV-2 surface flexibility outputs were observed (**Figure 2B**), and the minimum flexibility score of 0.864 ranging from 159 to 165 amino acids with the FCYMHHM hepta-peptide sequence was observed. The maximum flexibility score of 1.099 ranging from 118 to 124 amino acids with the YNGSPSG hepta-peptide sequence was observed (**Supplementary S2**).

### Parker hydrophilicity prediction

Parker’s hydrophilicity scale analysis was performed to find the hydrophilicity of the peptides associated with peptide retention times using HPLC on the reversed-phase column. Hydrophilic regions and associated antigenic sites have been observed through immunological analyses (Parker et al., 1986). Parker’s hydrophilicity method-predicted peptides were visualized (**Figure 2C**), and the residues were positioned along the x-axis and hydrophilicity was positioned along the y-axis. The maximum hydrophilicity score of 5.329 was observed to range from 92 to 98 having a hepta-peptide (DTANPKT) sequence. However, the minimum hydrophilicity score was observed—4.257 ranging from 204 to 210 with the hepta-peptide (VLAWLYA) sequence (**Supplementary S3**).

### Kolaskar and Tongaonkar antigenicity prediction

The Kolaskar and Tongaonkar process was used to measure the antigenicity (**Figure 2D**); the highest antigenicity value of 1.220 was observed in two hepta-peptide sequences, CVLKLKV (85 to 91) and CPRHVIC (38 to 41). The predicted amino acid residues results along with CTL epitopes of SARS-CoV-2 are mentioned in **Table 1**. The minimum antigenicity value of 0.844 was observed for hepta-peptide sequence NGMNGRT from 274 to 280 amino acid positions (**Supplementary S4**).

**Table 1:** Predicted amino acid residues along with CTL epitopes of SARS-CoV-2.

Residue number	Peptide sequence	MHC binding affinity prediction	Rescale binding affinity	C-terminal cleavage affinity	TAP transport efficiency
**146**	GSVGFNIDY	0.3112	1.3211	0.9565	2.8570
**1**	SGFRKMAFP	0.0549	0.2332	0.0275	0.0010
**5**	KMAFPSGKV	0.0729	0.3094	0.9651	0.6920
**110**	QTFSVLACY	0.2625	1.1146	0.9725	2.9980
**9**	PSGKVEGCM	0.0574	0.2438	0.1230	-0.4030
**16**	CMVQVTCGT	0.0649	0.2754	0.0334	-0.5770
**17**	MVQVTCGTT	0.0672	0.2853	0.0501	-0.5590
**10**	SGKVEGCMV	0.0541	0.2297	0.4221	-0.0460
**201**	TVNVLAWLY	0.6255	2.6559	0.8852	2.9570
**4**	RKMAFPSGK	0.0597	0.2533	0.1516	0.7180
**12**	KVEGCMVQV	0.0775	0.3290	0.5447	0.3860
**153**	DYDCVSFCY	0.2097	0.8905	0.9722	2.7060
**174**	GTDLEGNFY	0.7930	3.3669	0.6229	2.7020
**2**	GFRKMAFPS	0.0512	0.2174	0.0246	-2.1540
**15**	GCMVQVTCG	0.0499	0.2118	0.0306	-1.6560
**6**	MAFPSGKVE	0.0476	0.2021	0.0253	-1.2530
**46**	SEDMLNPNY	0.1528	0.6489	0.8406	2.6760
**3**	FRKMAFPSG	0.0505	0.2146	0.0527	-1.3160
**13**	VEGCMVQVT	0.0535	0.2272	0.0339	-0.8410
**93**	TANPKTPKY	0.1676	0.7118	0.9755	2.7230
**7**	AFPSGKVEG	0.0551	0.2341	0.1583	-1.0630
**14**	EGCMVQVC	0.0548	0.2326	0.0285	-0.4000
**8**	FPSGKVEGC	0.0537	0.2278	0.0300	-0.2350
**11**	GKVEGCMVQ	0.0455	0.1932	0.2424	-0.1030
**286**	LLEDEFTPF	0.1132	0.4807	0.9503	2.5680

### Structure-based epitope prediction

ElliPro was used to determine the association of the predicted epitopes, protein structure antigenicity, flexibility, and accessibility within the 3D structure (Ponomarenko et al., 2008). The protein antibody interactions were observed to distinguish the predicted epitopes. The top-ranked four conformational epitopes with ≥0.5 scores were selected. The isoelectric point (pI) (Lamiable et al., 2016) was observed to analyze the atom percentage and molecular bulk responsible for the antibody binding. The pI value of 5.95 was observed for the selected target protein. The name of the residue, lengths, and locations of the top-ranked four conformational predicted epitopes were critically analyzed (**Table 2**), and a 0.517–0.719 score was observed.

**Table 2:** Selected scores and interacted residues of top-ranked discontinuous epitopes.

Serial no.	Predicted discontinuous epitopes residues	Residues	Score
**1**	A:Q244, A:D245, A:V247, A:D248	4	0.719
**2**	A:S1, A:G2, A:F3, A:T198, A:A211, A:V212, A:I213, A:N214, A:G215, A:D216, A:R217, A:W218, A:F219, A:L220, A:N221, A:R222, A:F223, A:T225, A:T226, A:L227, A:N228, A:D229, A:F230, A:N231, A:L232, A:V233, A:A234, A:M235, A:K236, A:Y237, A:N238, A:Y239, A:E240, A:P241, A:L242, A:T243, A:G251, A:P252, A:S254, A:A255, A:Q256, A:T257, A:G258, A:I259, A:A260, A:L262, A:D263, A:A266, A:S267, A:K269, A:E270, A:L271, A:L272, A:Q273, A:N274, A:G275, A:M276, A:N277, A:G278, A:R279, A:T280, A:I281, A:L282, A:G283, A:S284, A:A285, A:L286, A:C300, A:S301, A:G302, A:V303, A:T304, A:F305	73	0.709
**3**	A:G11, A:K12, A:G15, A:C16, A:T21, A:C22, A:G23, A:T24, A:D33, A:D34, A:R40, A:C44, A:T45, A:S46, A:E47, A:D48, A:M49, A:L50, A:N51, A:P52, A:N53, A:Y54, A:E55, A:D56, A:L57, A:L58, A:I59, A:R60, A:K61, A:S62, A:N63, A:H64, A:N65, A:L67, A:Q69, A:A70, A:G71, A:N72, A:V73, A:Q74, A:L75, A:R76, A:V77, A:I78, A:G79, A:H80, A:S81, A:M82, A:K90, A:V91, A:D92, A:T93, A:A94, A:N95, A:P96, A:K97, A:T98, A:P99, A:K100, A:N133, A:T135, A:D155, A:C156, A:G183, A:P184, A:F185, A:V186, A:D187, A:R188, A:Q189, A:T190, A:A191, A:Q192, A:A193, A:A194, A:G195, A:T196, A:D197	78	0.699
**4**	A:L167, A:P168, A:T169, A:V171	4	0.517

### Molecular docking analyses with HLA-B

Molecular docking analyses were performed on the selected CTL epitopes of designed peptides. The global energy of the selected CTL epitopes was observed between -0.54 and -26.21 kcal/mol. Moreover, the binding affinities were also observed with Van der Waals (VdW) energy values of -3.33 to -26.36 kcal/mol (**Table 3**). The HLA-B effective binding affinities were observed through molecular docking of the selected CTL-predicted epitopes (SEDMLNPNY, GSVGFNIDY, LLEDEFTPF, DYDCVSFCY, GTDLEGNFY, QTFSVLACY, TVNVLAWLY, and TANPKTPKY).

**Table 3:** Peptides-MHC class I, HLA-B interaction characteristics of designed peptides against SARS-CoV-2.

Peptide sequence	Global energy (kcal/mol)	Attractive VDW energy (kcal/mol)	H-bond energy (kcal/mol)	Peptidase-MHC pair	Bond distance (Å)	Conserved residues
**GTDLEGNFY**	-1.65	-5.52	0.36	ASN 7 CB-PRO 168.A CB ASN 7 CA-PRO 168.A CB ASN 7 N-PRO 168.A CG ASN 7 N-PRO 168.A CB PHE 8 N-PRO 168.A CA	1.557 1.755 1.880 1.956 2.286	GLN189 ASN142 MET49 GLU166 PRO168
**TVNVLAWLY**	-5.53	-3.33	0.00	TRP 7 O-SER 46.A CB TRP 7 NE1-SER 46.A TRP 7 CH2-GLU 47.A OE1 ASN 3 O-HOH 711.A O TRP 7 N-HOH 711.A O	2.433 2.377 2.711 2.426 2.675	GLN189 ASN142 MET49 GLU166 PRO168
**GSVGFNIDY**	-26.21	-21.80	15.63	YR 9 O2-LEU 27.A CD1 TYR 9 CA-CYS 145.A SG ILE 7 O-HOH 671.A O ASP 8 CG-ASN 142.A CA TYR 9 O2-LEU 27.A	2.172 2.606 1.881 2.779 2.355	GLN189 ASN142 MET49 GLU166 PRO168
**QTFSVLACY**	-17.39	-21.55	20.78	TYR 9 O2-SER 46.A OG TYR 9 O2-SER 46.A CB	2.616 3.049	GLU166 PRO168
**DYDCVSFCY**	-14.90	-5.82	2.72	TYR 2 CZ-GLN 189.A NE2 PHE 7 CG-ASN 142.A ND2 TYR 2 CD2-HOH 711.A O PHE 7 CD2-ASN 142.A PHE 7 CE2-ASN 142.A CB	2.394 2.406 2.411 2.597 2.959	GLN189 ASN142 MET49 GLU166 PRO168
**TANPKTPKY**	-15.11	-26.36	33.19	PRO 7 CB-GLU 166.A CB THR 6 CG2-HOH 650.A O LYS 8 CG-HOH 515.A O TYR 9 O2-HOH 650.A O PRO 7 CB-GLU 166.A CD	2.529 2.132 2.155 1.728 2.654	GLN189 ASN142 MET49 GLU166 PRO168
**SEDMLNPNY**	-17.58	-19.77	25.28	GLU 2 CD-MET 49.A SD TYR 9 C-HOH 671.A O ASN 6 OD1-GLN 189.A ASN 6 CG-GLN 189.A NE2 GLU 2 OE2-MET 49.A SD	2.089 1.865 1.863 2.128 2.079	GLN189 ASN142 MET49 GLU166 PRO168
**LLEDEFTPF**	-0.54	-3.54	0.37	PHE 6 CA-GLU 166.A CB PHE 6 C-GLU 166.A CG THR 7 CG2-HOH 515.A O GLU 5 O-LEU 167.A N LEU 1 CD2-THR 190.A	0.854 0.598 0.610 0.394 0.940	GLN189 ASN142 MET49 GLU166 PRO168

### Population coverage analyses

The population coverage analyses were performed on MHC-I, MHC-II, and associated HLA alleles. MHC-I epitopes resulted in the highest population coverage in Italy and China as 0.9019% and 0.5639% respectively. MHC-II selected epitopes had shown population coverage of 58.49% in Italy and 34.71% in China (**Supplementary S5**)

### Multiple-sequence alignment

The conserved residues of three selected coronavirus genomes (NC_045512.2, NC_004718.3, and NC_006577.2) were analyzed and detected through MSA. MSA has shown conserved domains in all selected strains of the coronavirus restored with a strain of novel SARS-CoV-2 outbreak. Moreover, the binding domains of the previously reported MERS and SARS strains were similar to the novel SARS-CoV-2 outbreak.

### Molecular docking analyses

**Figure 3:**
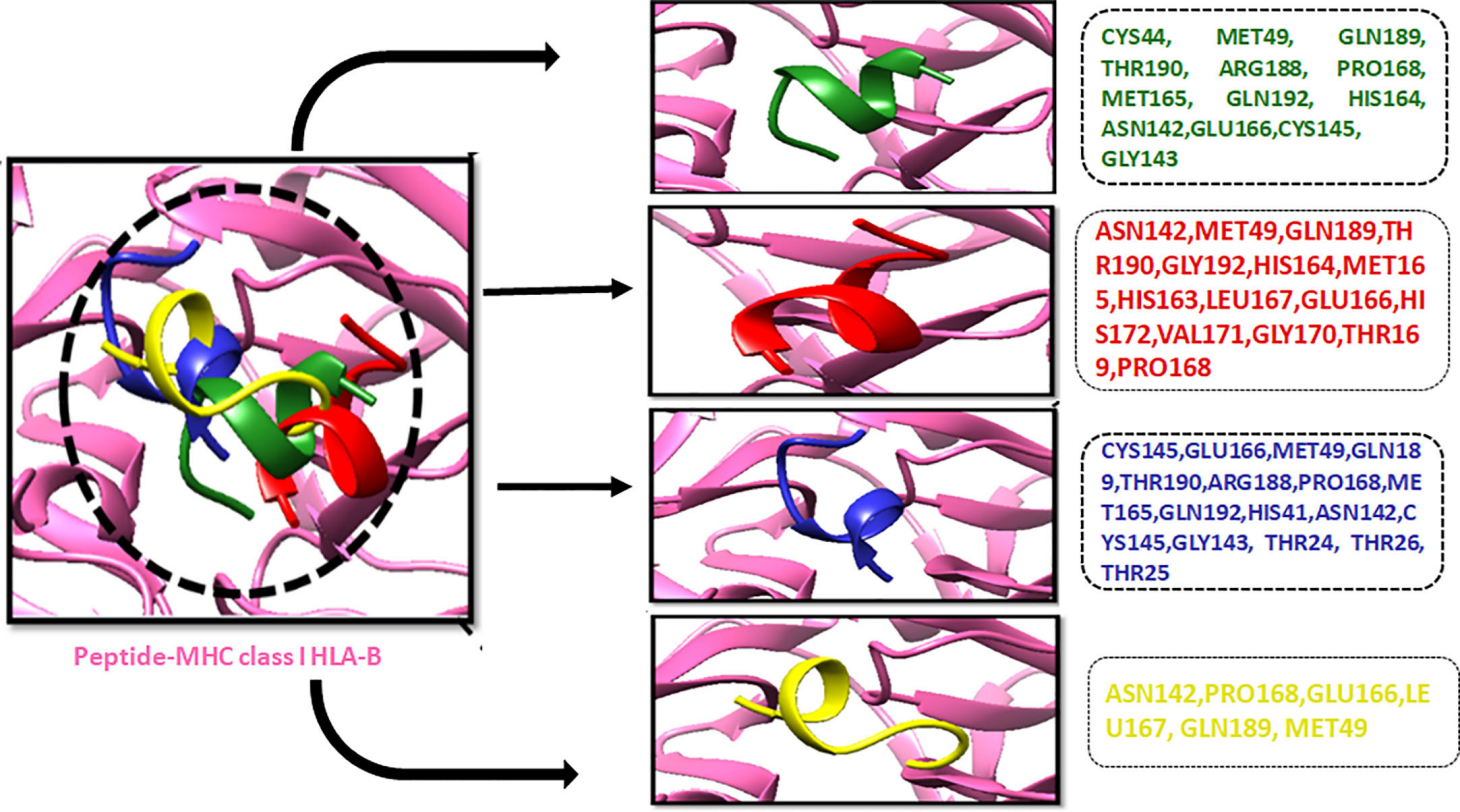
The four top-ranked peptides of MHC class I, HLA-B binding residues, and sequences are displayed in different colors.

The compound library (FDA drugs) was used for virtual screening. Molecular docking analyses revealed the significant values of the selected peptides (Irwin and Shoichet, 2005). There were 3,447 compounds screened, and molecular docking analyses showed variations in binding energy. Molecular docking was performed against the selected library, and the top-ranked docked compounds based on higher binding affinities, interacting residues, least binding energies, and drug properties were selected for further analyses. The top 10 complexes were selected, visualized, and analyzed. The top four interacting docked compounds were analyzed, and their similar binding pockets were observed (**Figure 3**). Met-49, Asn-142, Pro-168, Glu-166, and Gln-189 were observed as conserved residues.

**Figure 4:**
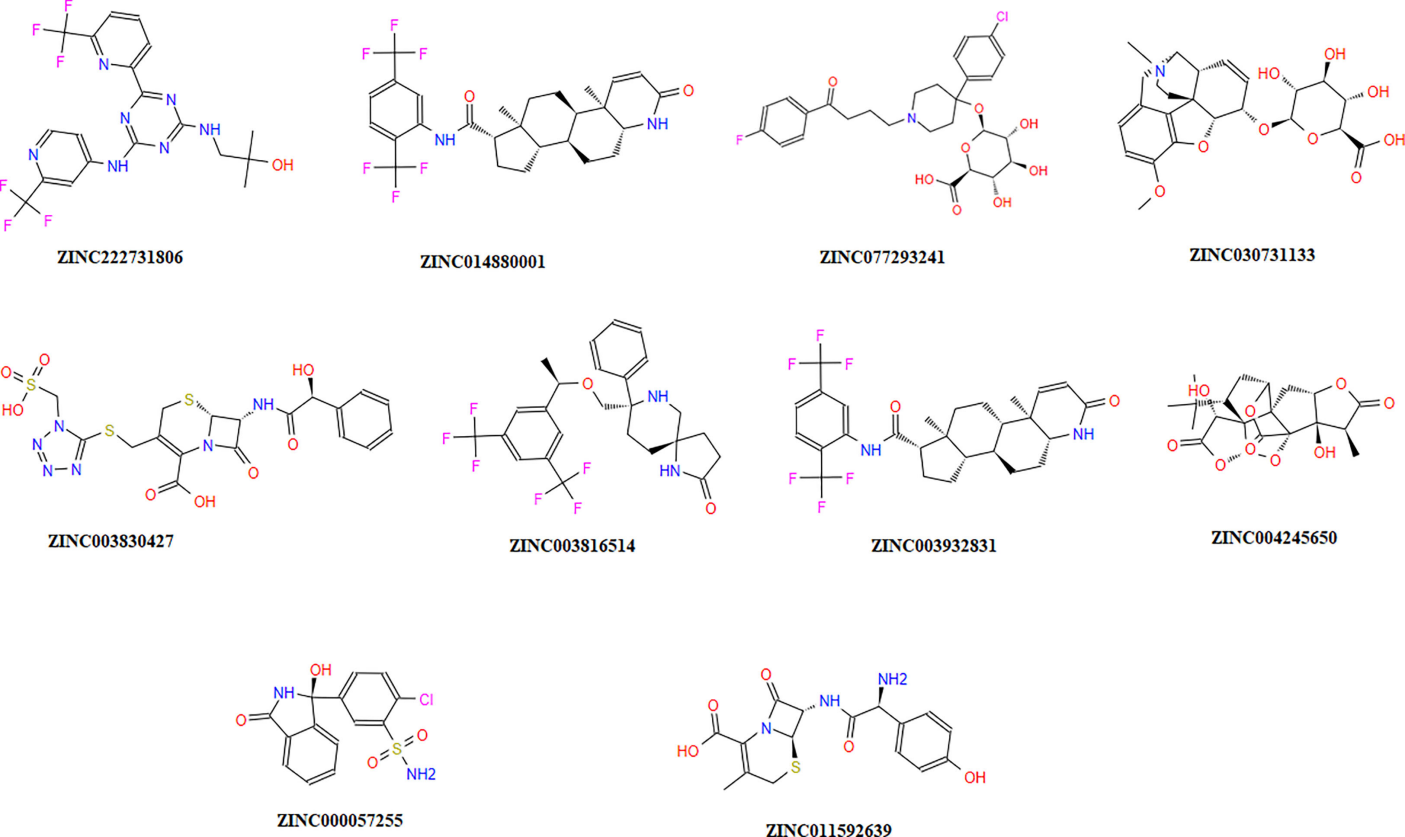
Top-ranked selected compounds (i) ZINC222731806, (ii) ZINC014880001 (iii) ZINC077293241, (iv) ZINC003830427, (v) ZINC030731133, (vi) ZINC003816514 (vii) ZINC003932831, (viii) ZINC004245650, (ix) ZINC000057255, and (x) ZINC011592639.

**Figure 5:**
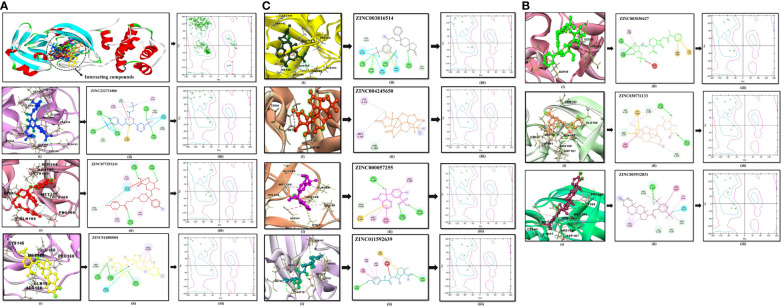
**(A)** Conserved region of the selected non-structural protein (PDB: 6W63). Top-ranked selected compounds (ZINC222731806 (dark blue), ZINC077293241 (red), and ZINC014880001 (yellow)): compound (i) showing an interaction with the selected protein, (ii) depicting the 2D structure of the selected compound as well as interacting residues, (iii) showing Ramachandran conformation. **(B)** The scrutinized compounds showed conserved interacting residues (ZINC003830427 (green), ZINC030731133 (light brown), and ZINC003932831 (magenta)): compound (i) showing interaction with the selected protein, (ii) depicting the 2D structure of the compound as well as interacting residues, and (iii) showing Ramachandran conformation. **(C)** ZINC003816514 (dark green), ZINC004245650 (orange-red), ZINC000057255 (purple), and ZINC011592639 (turquoise) selected compounds: compound (i) showing an interaction with the selected protein, (ii) depicting the 2D structure of the compound as well as interacting residues, (iii) and showing Ramachandran conformation.

The chemical library was used, and ZINC222731806, ZINC014880001, ZINC077293241, ZINC003830427, ZINC030731133, ZINC003816514, ZINC003932831, ZINC004245650, ZINC000057255, and ZINC011592639 compounds were selected with least binding energy ranges from -7.5 to -8.8 kcal/mol, and we draw their promising structure as mentioned in **Figure 4**, at similar binding pocket and common binding sites (**Table 4**). The FDA-approved compounds play a vital role in different diseases and the top-ranked 10 docked complexes bound at the similar binding region. The selected compounds may predict the replication inhibition at observed residues (Pro-168, His-41, Arg-188, Gln-189, Cys-145, Glu-166, Met-49, Asp-187, Met-165, His-164, and CYS44). A plot was generated to analyze the docked complexes (**Figures 5A–C**).

**Table 4:** Drug like properties and molecular docking analyses of the selected ten top-ranked compounds.

Ligands	Binding energy (kcal/Mol)	RMSD value	M. weight (g/Mol)	A-Log P value	Water solubility (logS)	H-Bond acceptor	H-Bond donor	Interacting residues	Lipinski rule
**ZINC222731806**	-8.8	2.284	473.38	4.29	-3.182	8	3	Cys44, Gln192, Pro168, Arg188, Gln189, Thr190, Cys145, Glu166, Asn142, Phe140, Met165, His41	Accepted
**ZINC077293241**	-8.1	2.247	552.00	2.34	-3.419	8	4	Met165, His41, Gln189, Glu166, Pro168, Asn142, Ser144, Cys145	Accepted
**ZINC014880001**	-8	2.207	528.54	6.58	-4.341	2	2	Cys145, Glu166, Met165, Gln189, Pro168, Arg188	Rejected
**ZINC003830427**	-8	2.074	542.58	-0.92	-3.133	12	4	Glu166, Leu167, Pro168, Gln189, Cys44	Rejected
**ZINC030731133**	-7.9	2.32	475.49	-0.58	-2.351	9	4	Asp187, Arg188, Gln189, Glu166, Cys44, His41, Asn142	Accepted
**ZINC003932831**	-7.8	1.815		6.58	-4.341	2	2	Asp187, Arg188, Gln189, Cys44, His41, Met49, Glu166, Met165, Pro168	Rejected
**ZINC003816514**	-7.7	2.812	500.48	5.73	-3.766	3	2	Glu166, Met165, Pro168, Gln192, Thr190, Arg188, Gln189, Met49, Asn142, Leu141	Accepted
**ZINC004245650**	-7.6	1.252	408.40	-0.34	-2.412	9	2	Met49, Gln189, Cys44	Accepted
**ZINC000057255**	-7.5	0.306	318.35	0.58	-3.513	4	3	His164, Met165, Glu166, Gln189, His41, Met49, Cys44, Arg188	Accepted
**ZINC011592639**	-7.5	0.555	363.40	0.15	-3.082	6	4	His163, Met165, Glu166, Asn142, His41, Asp187	Accepted

The drug compounds were selected with the goal that they could inhibit SARS-CoV-2 replication without consuming much time. For toxicity, absorption, excretion, metabolism, and distribution analyses of the selected docked complexes were performed (**Table 4**). All the selected complexes had shown the highest binding affinities with close binding sites. The aqueous solubility prediction of the selected compounds defined water at 25°C and disclosed that selected molecules can dissolve in water. The selected molecules may have inadequate oral bioavailability and lower LogP values following the Lipinski’s rule of five (**Supplementary S6**).

### Molecular dynamics simulation

**Figure 6:**
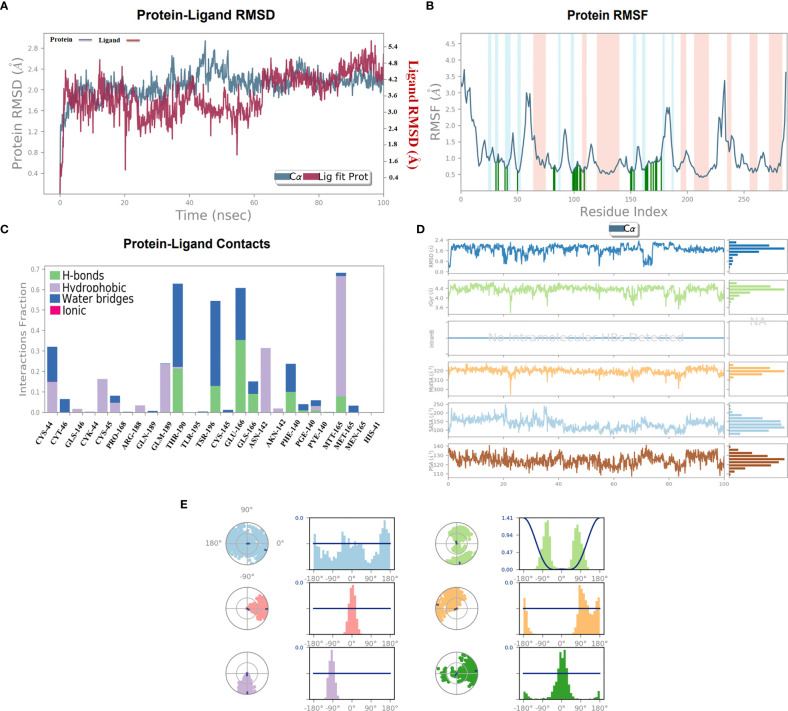
**(A)** 100-ns MD simulation analyses. **(A)** RMSD (A°) plot of the complex. **(B)** 2D graph of residual flexibility analyses. **(C)** The number of hydrogen bonds in the stable complex **(D)** Radius of gyration (Rg, Å) plots, PSA, SASA, MolSA, and RMSD of the complex compactness of unbound and bound states. **(E)** Ligand conformational spaces of the selected ligand.

The stability of the top-ranked complex with non-structural proteins (PDB: 6W63) was investigated through MD simulation. A 100-ns MD simulation was performed, and the RMSD of the protein–ligand complex was analyzed to evaluate the conformational stability of the complex. The RMSD plot showed fluctuations in the conformation of the protein and ligand complexes, with an initial rapid change until 60 ns, after which the complex stabilized with the minimal fluctuations. The RMSD value of the stable conformations of the complex exhibited a high degree of conformational stability, with an average deviation of 2.4 Å due to the conformational change necessary for the protein to interact with the ligand. The ligand remained within the binding pocket, making significant interactions, whereas the backbone remained coherent, with a deviation of 1.6–2.6 Å (**Figure 6A**).

To investigate the flexibility of the complexes and the role of each amino acid in contributing to the overall flexibility, an RMSF analysis was conducted. The utilized methodology is widely used to study the dynamic behavior of protein–protein interactions. The observed results showed that the generated complex had a low RMSF range for most of the residues, indicating high flexibility in binding with the C30 endopeptidase (**Figure 6B**). The finding suggested that the efficacy of the vaccine candidates in enhancing C30 endopeptidase responses may be correlated with their ability to adapt to different conformational states.

The RMSF analysis provided the valuable insights into the dynamic behavior of the complex, which could aid in optimizing the vaccine design and enhancing its efficacy. The generated findings were consistent with earlier research (Padma et al., 2023), which emphasized the importance of flexibility in interactions. Therefore, the RMSF analysis presented the significance of flexibility in the design of effective vaccines.

The formation of the hydrogen bonds between a ligand and the amino acid plays a crucial role in the stabilizing of the protein–ligand complexes. The number of hydrogen bonds present between the protein and the ligand can be calculated through simulation studies, which also allow investigating the variation in the number of changes over time. The simulation analyses indicated that the number of hydrogen bonds formed between the protein and the ligand remained relatively constant during the simulation period indicating a high degree of stability. The stability can be attributed to the critical number of hydrogen bonds that form between the two entities. A substantial number of hydrogen bonds between the protein and the complex were observed with an average of one hydrogen bond being formed throughout the simulation time. It was observed that the formation of the stable complexes may be facilitated by the presence of the observed hydrogen bonds.

The stability of the protein–ligand complex was achieved through a diverse set of interactions, including hydrogen bonds, hydrophobic interactions, and water bridges. Specifically, the ligand formed hydrogen bonds with Thr-190, Tys-145, Phe-140, and Met-165 residues (**Figure 6C**). Moreover, the ligand also participated in hydrophobic interactions with Lys-44, Pro-168, Ans-142, and Met-165. Additionally, a water bridge was established between the ligand positively charged nitrogen atom and the negatively charged side chain of the selected protein.

In order to assess the stability and compactness of the protein–ligand complex (**Figure 6D**), SASA plots were generated to determine the area accessible to the solvent. The SASA of the protein complex displayed minimal fluctuation, indicating a high degree of stability. Moreover, the SASA of the ligand in its bound state was lower (150 Å2) than in its unbound state (220 Å2), indicating a more compact conformation upon the binding. Additionally, the MolSA of the ligand in its bound state was higher (330 Å2) than in its unbound state (310 Å2). The compactness of the protein in the complex was assessed through the measurement of its Radius of Gyration (Rg). During the early stages, Rg values fluctuated up to 60 ns; however, it became stable between 65 and 100 ns. The Rg values observed between 4.0 Å and 4.4 Å suggested a compact protein–ligand-bound state. Moreover, the stable Rg values between 12 and 16 Å suggested that the overall shape and size of the complex remained constant over a certain range of concentration. The observed results indicated that the docked complex possessed strong interactions between its components, which contributed to its greater compactness. Furthermore, the PSA of the ligand in its bound state was lower (130 Å2) than in its unbound state, further indicating a more compact conformation upon binding. Overall, the observed results demonstrated the stability and compactness of the protein–ligand complex and the strong interactions between its components.

The torsion profile is a powerful tool that sheds light on the flexibility of the ligand and the ability to conform the binding site of the protein. The ligand torsion profile of the top-ranked ligands (**Figure 6E**) was analyzed, and the x-axis denoted the torsion angle in degrees and the y-axis represented the associated energy. The distinct energy minima were observed in the plot, indicating the presence of multiple low-energy conformations of the ligand. Further analysis of the torsion profile revealed that the ligand adopts a planar orientation at a torsion angle of approximately -90°, with specific functional group orientations. The second lowest energy conformation occurred at a torsion angle of 180°, where the ligand takes on a twisted orientation. In essence, the torsion profile provided information about the flexibility and behavior of the ligand molecules in bound state.

The changes in protein conformation during and after the interaction were important for the stability of the complex. The conformational changes of the non-structural protein were observed through superimposition of the unbound structure with the vaccine-bound structures over the 100 ns course of MD simulation. The study also utilized a torsion profile to identify the energetically favorable conformations of the ligand, providing insight into ligand behavior and protein–ligand interactions.

### Antibody-mediated immune response

First, we predict Kolaskar and Tongaonkar process-based antigenicity (**Figure 2D**), where we observed two hepta-peptide sequences CVLKLKV (85 to 91) and CPRHVIC (38 to 41) with 1.220 values, and a minimum antigenicity value of 0.844 was observed for hepta-peptide sequence NGMNGRT from 274 to 280 amino acid positions, as mentioned in **Supplementary S4**. Then, we performed immune simulation and examined the immune responses generated in response to repeat the exposure to refined C30 endopeptidase. The simulation analyses revealed that the C30 endopeptidase induces high humoral immune response in the mammalian system. The refined C30 endopeptidase induced weak primary immunoglobulin response after first immunogen exposure; however, the second exposure demonstrated the elevated immunoglobulin response with a high IgM+IgG response. The major share of immune response during this stage was apparently mediated by IgM. Subsequently, the exposure to refined C30 endopeptidase further raised IgM+IgG titers and the intensity of IgM and IgG responses was observed to be similar. IgG response was mainly due to IgG1, whereas the contribution of IgG2 was negligible. Such high IgM+IgG response was further supported by an amplified population of diverse B-cell subpopulations, memory B cells, and B cells expressing IgM and IgG1 isotypes. The simulation assay also suggested persistence of active antibody-producing B cells for a prolonged period.

## Discussion

Animal-derived coronaviruses can traverse species boundaries and transmit illnesses that can be fatal, in contrast to the less severe human viral diseases that are constantly prevalent in the human population (Agarwal et al., 2022; Ahmad et al., 2022). All three SARS-CoVs have caused problems with the outbreak, the original from 2003, the MERS-CoV from 2012, and the current SARS-CoV-2. The present pandemic calls for urgently developing innovative and cost-effective preventative measures (Rayan et al., 2022). Coronaviruses are massive enclosed particles that contain a considerable size of positive-sense single-stranded RNA (+ssRNA), have a genetic code of around 30 kb, and resemble a crown, as identified in numerous studies (Dutta et al., 2022; Chaitanya, 2019; Lythgoe et al., 2022). Coronaviruses play a critical role in the viral replication cycle (Lythgoe et al., 2022). The viral 3CL^pro^ enzyme regulates the life cycle and replication of the virus (Cabero Pérez, 2020; Hu et al., 2022). As per our frontier consequence and identification, 3CL^pro^ has been regarded as a possible target to develop antiviral drugs against SARS-CoV-2.

In contrast to conventional vaccine design, bioinformatics analyses enable the prediction of potent epitopes, which streamlines and expedites the vaccine design (Sajid et al., 2022). A suitable target for the B-cell epitope study might have been 3CL^pro^ since most of it is available outside the virion. Vaccination is a standard method for strengthening the host immune system against a particular infection. Various vaccinations, including natural or recombinant, remain costly and time-consuming and require a very long period to be launched (de Pinho Favaro et al., 2022). The immature vaccine-poor adaptive immunity and high antigenic load also result in allergic reactions. Developing multi-omics and immunoinformatics techniques have made it simpler to identify the epitopes that trigger a potent immune response.

The peptide-based vaccines are essential due to their ultra-fast mechanism of action, fewer side effects, and less toxicity (Albekairi et al., 2022). The peptide-based vaccinations are anticipated to offer a safer option to conventional immunizations. The large-scale manufacture of the peptide-based products would be more straightforward due to their chemical synthesis and high repeatability rate. Scientists have made many efforts as an immediate response to design peptide-based vaccines. The peptide inhibitors play an exciting role in developing the peptide-based vaccines. The immunoinformatics approaches are constructive, reduce the workload of laboratory trials, and are time-saving and cost-effective compared with traditional drug design approaches (Vanhee et al., 2011). The researchers have identified several vaccine candidates by utilizing the computational techniques with promising preclinical results (Davies and Flower, 2007). CTL epitopes help to design the peptide-based vaccines against human leukocyte antigen-B protein (Tahir et al., 2018).

Extensive *in silico* analyses were performed to design epitope-based vaccine by targeting CL^pro^ for CTL epitopes. The current study revealed four epitopes having an immunogenic, non-toxic, and non-allergenic response. The top 25 epitopes showed 97.87% worldwide population coverage. For validation of results, CTL epitopes were optimized for all ergenicity and antigenicity as per the method of an earlier study (Dimitrov et al., 2014). Furthermore, we hypothesized epitope population coverage analyses for MHC-I (**Table 3**) indicating 0.0373 with an estimated hit rate of 0.3. Based on an *in silico* investigation, we predict the peptide designs in contrast to eight epitopes and HLA-B interaction characteristics of designed peptides for effective binding residues. The generated results were reconciled with literature (Rezaei and Nazari, 2022). The pI value was observed 5.95, and top-ranked four conformational predicted epitopes were used to predict the names of the residues, lengths, and locations (**Table 2**) and reconciled with previous literature (Sajid et al., 2022).

Surface accessibility was analyzed, and the hexa-peptide sequence KTPKYK displayed the highest score of 8.254. On the other hand, the lowest score of 0.114 was found in the 97- to 102-amino acid regions. Another sequence, FSVLAC, showed up in the 112- to 117-a.a. region. The surface flexibility analysis revealed that the hepta-peptides FCYMHHM and YNGSPSG had a score of 0.864, with amino acid ranges of 159 to 165 and 118 to 124, respectively, as shown in **Figure 2B**. For surface flexibility prediction, the lowest score was 0.864, ranging from 159 to 165 aa with the FCYMHHM heptapeptide sequence, and the highest score was 1.099, spanning from 118 to 124 aa with the YNGSPSG heptapeptide sequence. Parker’s hydrophilicity scale analyses were also done for the hydrophilicity of peptides associated with peptide retention times using HPLC on a reversed-phase column as per the method of earlier researchers (Iranparast et al., 2022; Sajid et al., 2022). Hydrophilic regions and associated antigenic sites have been observed through immunological analysis (Parker et al., 1986) based on Parker’s hydrophilicity, as shown in **Figure 2C**. Between 204 and 210 amino acids, the VLAWLYA hepta-peptide sequence was observed with a minimum hydrophilicity score of -4.257 and reconciled with earlier research (Sajid et al., 2022). The highest antigenicity value of 1.220 was observed in two hepta-peptide sequences, CVLKLKV (85 to 91) and CPRHVIC (38 to 41). The minimum antigenicity value of 0.844 was reported for hepta-peptide sequence NGMNGRT from 274 to 280, and results were matched with (Adhikari et al., 2022).

The global energy of the CTL epitopes selected for this study ranged from -0.54 to -26.21 kcal/mol, and results were matched with (Joshi et al., 2023). The binding solid affinities were also determined, with binding energies ranging from -3.33 to -26.36 kcal/mol, as mentioned in **Table 3**. HLA-B binding affinities were calculated for the CTL-predicted epitopes, namely, SEDMLNPNY, GSVGFNIDY, LLEDEFTPF, DYDCVSFCY, GTDLEGNFY, QTFSVLACY, TVNVLAWLY, and TANPKTPKY. We selected top 10 complexes and top four interacting docked compounds and their similar binding pockets as shown in **Figure 4**, where we explored Met-49, Asn-142, Pro-168, Glu-166, and Gln-189 residues and observed that Pro-168, His-41, Arg-188, Gln-189, Cys-145, Glu-166, Met-49, Asp-187, Met-165, His-164, and Cys-44 residues are effective binding interactions (**Figures 5A–C**). The virtual screening of potential compounds was performed, and top 10 compounds showing binding affinities between -7.5 and -8.8 kcal/mol were observed. Our MD simulation study results showed that 3CLpro-vaccine is complex and highly stable throughout the simulation time. The interaction between the protein and the vaccine was primarily stabilized by electrostatic configurations. During the dynamics, the docked complex also showed increased rigidity in the motion of residues. The immune simulation data suggested that using the epitopes of 3CLpro could lead to the design of an effective SARS-CoV-2 vaccine.

Our study explored the potential of C30 endopeptidase to elicit an immune response in humans using immunoinformatics and immune simulation techniques. We found that C30 endopeptidase has multiple B-cell and T-cell epitopes, indicating its potential to stimulate high-titered antibody responses and reduce infections, including SARS-CoV-2. The immune simulation data predicted a strong immune response dominated by IgM and IgG1, with long-lived B-cell responses. Therefore, we suggest that refined C30 endopeptidase could be used in immunotherapeutic approaches to provide long-term protection against infections in the population. Moreover, in our experimental study, we identified 10 promising compounds, namely, ZINC222731806, ZINC077293241, ZINC014880001, ZINC003830427, ZINC030731133, ZINC003932831, ZINC003816514, ZINC004245650, ZINC000057255, and ZINC011592639, that could be used in C30 endopeptidase-based antibody therapy to treat infections, including SARS-CoV-2. Additionally, the epitopes of C30 endopeptidase could be used in the design of a potential vaccine against infections. These findings provide a promising avenue for the use of these compounds to target 3CL^pro^ and treat infections caused by SARS-CoV-2.

## Conclusion

Our study formulates a multi-epitope-based peptide vaccine using both T-cell and B-cell epitopes occurring in the 3CLpro protein that efficiently target the SARS-CoV-2-mediated immune response. Extensive *in silico* analyses were performed to scrutinize potential peptide-based inhibitors. CTL epitopes showed potential targets for a peptide-based vaccine. An *in silico* study designed 10 different vaccine candidates’ compounds, which (ZINC222731806, ZINC077293241, ZINC014880001, ZINC003830427, ZINC030731133, ZINC003932831, ZINC003816514, ZINC004245650, ZINC000057255, and ZINC011592639) showed least binding energy and high binding affinity. Molecular dynamics (MD) study of the docked 3CLpro-vaccine complex delineated it to be highly stable during simulation time, and the stabilization of interaction was majorly contributed by electrostatic energy. The docked complex also showed low deformation and increased rigidity in motion of residues during dynamics. The immune simulation data indicated toward the possibility of designing an effective SARS-CoV-2 vaccine using the epitopes of 3CLpro. However, this claim needs additional experimental validation in non-human primates for further preclinical development.

## Data availability statement

The original contributions presented in the study are included in the article/**Supplementary Material**. Further inquiries can be directed to the corresponding authors.

## Author contributions

Conceptualization, Methodology, Investigation, data collection and Writing-original manuscript: SM, MSA, MN, MS, MA, AB. Investigation, data collection: MW, SS, IZ. Editing and proof reading: SR, SAS, RS, MS, DA, WH, K-TC. Supervision: IZ, SAS, RS. All authors contributed to the article and approved the submitted version.
