# Supplementary material for: Elucidation of novel compounds and epitope-based peptide vaccine design against C30 endopeptidase regions of SARS-CoV-2 using immunoinformatics approaches
Source: Front Cell Infect Microbiol. 2023 May 24;13:1134802. doi: 10.3389/fcimb.2023.1134802 (PMC10244718; doi:10.3389/fcimb.2023.1134802)
Supplement: Supplementary file 1 [file DataSheet_1.zip › S2 Surface Flexibility-Karplus and Schulz method.docx]

## Karplus & Schulz Flexibility Prediction Results

**Input Sequences**

| 1 | MESLVPGFNE KTHVQLSLPV LQVRDVLVRG FGDSVEEVLS EARQHLKDGT CGLVEVEKGV |
| --- | --- |
| 61 | LPQLEQPYVF IKRSDARTAP HGHVMVELVA ELEGIQYGRS GETLGVLVPH VGEIPVAYRK |
| 121 | VLLRKNGNKG AGGHSYGADL KSFDLGDELG TDPYEDFQEN WNTKHSSGVT RELMRELNGG |
| 181 | AYTRYVDNNF CGPDGYPLEC IKDLLARAGK ASCTLSEQLD FIDTKRGVYC CREHEHEIAW |
| 241 | YTERSEKSYE LQTPFEIKLA KKFDTFNGEC PNFVFPLNSI IKTIQPRVEK KKLDGFMGRI |
| 301 | RSVYPVASPN ECNQMCLSTL MKCDHCGETS WQTGDFVKAT CEFCGTENLT KEGATTCGYL |
| 361 | PQNAVVKIYC PACHNSEVGP EHSLAEYHNE SGLKTILRKG GRTIAFGGCV FSYVGCHNKC |
| 421 | AYWVPRASAN IGCNHTGVVG EGSEGLNDNL LEILQKEKVN INIVGDFKLN EEIAIILASF |
| 481 | SASTSAFVET VKGLDYKAFK QIVESCGNFK VTKGKAKKGA WNIGEQKSIL SPLYAFASEA |
| 541 | ARVVRSIFSR TLETAQNSVR VLQKAAITIL DGISQYSLRL IDAMMFTSDL ATNNLVVMAY |
| 601 | ITGGVVQLTS QWLTNIFGTV YEKLKPVLDW LEEKFKEGVE FLRDGWEIVK FISTCACEIV |
| 661 | GGQIVTCAKE IKESVQTFFK LVNKFLALCA DSIIIGGAKL KALNLGETFV THSKGLYRKC |
| 721 | VKSREETGLL MPLKAPKEII FLEGETLPTE VLTEEVVLKT GDLQPLEQPT SEAVEAPLVG |
| 781 | TPVCINGLML LEIKDTEKYC ALAPNMMVTN NTFTLKGGAP TKVTFGDDTV IEVQGYKSVN |
| 841 | ITFELDERID KVLNEKCSAY TVELGTEVNE FACVVADAVI KTLQPVSELL TPLGIDLDEW |
| 901 | SMATYYLFDE SGEFKLASHM YCSFYPPDED EEEGDCEEEE FEPSTQYEYG TEDDYQGKPL |
| 961 | EFGATSAALQ PEEEQEEDWL DDDSQQTVGQ QDGSEDNQTT TIQTIVEVQP QLEMELTPVV |
| 1021 | QTIEVNSFSG YLKLTDNVYI KNADIVEEAK KVKPTVVVNA ANVYLKHGGG VAGALNKATN |
| 1081 | NAMQVESDDY IATNGPLKVG GSCVLSGHNL AKHCLHVVGP NVNKGEDIQL LKSAYENFNQ |
| 1141 | HEVLLAPLLS AGIFGADPIH SLRVCVDTVR TNVYLAVFDK NLYDKLVSSF LEMKSEKQVE |
| 1201 | QKIAEIPKEE VKPFITESKP SVEQRKQDDK KIKACVEEVT TTLEETKFLT ENLLLYIDIN |
| 1261 | GNLHPDSATL VSDIDITFLK KDAPYIVGDV VQEGVLTAVV IPTKKAGGTT EMLAKALRKV |
| 1321 | PTDNYITTYP GQGLNGYTVE EAKTVLKKCK SAFYILPSII SNEKQEILGT VSWNLREMLA |
| 1381 | HAEETRKLMP VCVETKAIVS TIQRKYKGIK IQEGVVDYGA RFYFYTSKTT VASLINTLND |
| 1441 | LNETLVTMPL GYVTHGLNLE EAARYMRSLK VPATVSVSSP DAVTAYNGYL TSSSKTPEEH |
| 1501 | FIETISLAGS YKDWSYSGQS TQLGIEFLKR GDKSVYYTSN PTTFHLDGEV ITFDNLKTLL |
| 1561 | SLREVRTIKV FTTVDNINLH TQVVDMSMTY GQQFGPTYLD GADVTKIKPH NSHEGKTFYV |
| 1621 | LPNDDTLRVE AFEYYHTTDP SFLGRYMSAL NHTKKWKYPQ VNGLTSIKWA DNNCYLATAL |
| 1681 | LTLQQIELKF NPPALQDAYY RARAGEAANF CALILAYCNK TVGELGDVRE TMSYLFQHAN |
| 1741 | LDSCKRVLNV VCKTCGQQQT TLKGVEAVMY MGTLSYEQFK KGVQIPCTCG KQATKYLVQQ |
| 1801 | ESPFVMMSAP PAQYELKHGT FTCASEYTGN YQCGHYKHIT SKETLYCIDG ALLTKSSEYK |
| 1861 | GPITDVFYKE NSYTTTIKPV TYKLDGVVCT EIDPKLDNYY KKDNSYFTEQ PIDLVPNQPY |
| 1921 | PNASFDNFKF VCDNIKFADD LNQLTGYKKP ASRELKVTFF PDLNGDVVAI DYKHYTPSFK |
| 1981 | KGAKLLHKPI VWHVNNATNK ATYKPNTWCI RCLWSTKPVE TSNSFDVLKS EDAQGMDNLA |
| 2041 | CEDLKPVSEE VVENPTIQKD VLECNVKTTE VVGDIILKPA NNSLKITEEV GHTDLMAAYV |
| 2101 | DNSSLTIKKP NELSRVLGLK TLATHGLAAV NSVPWDTIAN YAKPFLNKVV STTTNIVTRC |
| 2161 | LNRVCTNYMP YFFTLLLQLC TFTRSTNSRI KASMPTTIAK NTVKSVGKFC LEASFNYLKS |
| 2221 | PNFSKLINII IWFLLLSVCL GSLIYSTAAL GVLMSNLGMP SYCTGYREGY LNSTNVTIAT |
| 2281 | YCTGSIPCSV CLSGLDSLDT YPSLETIQIT ISSFKWDLTA FGLVAEWFLA YILFTRFFYV |
| 2341 | LGLAAIMQLF FSYFAVHFIS NSWLMWLIIN LVQMAPISAM VRMYIFFASF YYVWKSYVHV |
| 2401 | VDGCNSSTCM MCYKRNRATR VECTTIVNGV RRSFYVYANG GKGFCKLHNW NCVNCDTFCA |
| 2461 | GSTFISDEVA RDLSLQFKRP INPTDQSSYI VDSVTVKNGS IHLYFDKAGQ KTYERHSLSH |
| 2521 | FVNLDNLRAN NTKGSLPINV IVFDGKSKCE ESSAKSASVY YSQLMCQPIL LLDQALVSDV |
| 2581 | GDSAEVAVKM FDAYVNTFSS TFNVPMEKLK TLVATAEAEL AKNVSLDNVL STFISAARQG |
| 2641 | FVDSDVETKD VVECLKLSHQ SDIEVTGDSC NNYMLTYNKV ENMTPRDLGA CIDCSARHIN |
| 2701 | AQVAKSHNIA LIWNVKDFMS LSEQLRKQIR SAAKKNNLPF KLTCATTRQV VNVVTTKIAL |
| 2761 | KGGKIVNNWL KQLIKVTLVF LFVAAIFYLI TPVHVMSKHT DFSSEIIGYK AIDGGVTRDI |
| 2821 | ASTDTCFANK HADFDTWFSQ RGGSYTNDKA CPLIAAVITR EVGFVVPGLP GTILRTTNGD |
| 2881 | FLHFLPRVFS AVGNICYTPS KLIEYTDFAT SACVLAAECT IFKDASGKPV PYCYDTNVLE |
| 2941 | GSVAYESLRP DTRYVLMDGS IIQFPNTYLE GSVRVVTTFD SEYCRHGTCE RSEAGVCVST |
| 3001 | SGRWVLNNDY YRSLPGVFCG VDAVNLLTNM FTPLIQPIGA LDISASIVAG GIVAIVVTCL |
| 3061 | AYYFMRFRRA FGEYSHVVAF NTLLFLMSFT VLCLTPVYSF LPGVYSVIYL YLTFYLTNDV |
| 3121 | SFLAHIQWMV MFTPLVPFWI TIAYIICIST KHFYWFFSNY LKRRVVFNGV SFSTFEEAAL |
| 3181 | CTFLLNKEMY LKLRSDVLLP LTQYNRYLAL YNKYKYFSGA MDTTSYREAA CCHLAKALND |
| 3241 | FSNSGSDVLY QPPQTSITSA VLQSGFRKMA FPSGKVEGCM VQVTCGTTTL NGLWLDDVVY |
| 3301 | CPRHVICTSE DMLNPNYEDL LIRKSNHNFL VQAGNVQLRV IGHSMQNCVL KLKVDTANPK |
| 3361 | TPKYKFVRIQ PGQTFSVLAC YNGSPSGVYQ CAMRPNFTIK GSFLNGSCGS VGFNIDYDCV |
| 3421 | SFCYMHHMEL PTGVHAGTDL EGNFYGPFVD RQTAQAAGTD TTITVNVLAW LYAAVINGDR |
| 3481 | WFLNRFTTTL NDFNLVAMKY NYEPLTQDHV DILGPLSAQT GIAVLDMCAS LKELLQNGMN |
| 3541 | GRTILGSALL EDEFTPFDVV RQCSGVTFQS AVKRTIKGTH HWLLLTILTS LLVLVQSTQW |
| 3601 | SLFFFLYENA FLPFAMGIIA MSAFAMMFVK HKHAFLCLFL LPSLATVAYF NMVYMPASWV |
| 3661 | MRIMTWLDMV DTSLSGFKLK DCVMYASAVV LLILMTARTV YDDGARRVWT LMNVLTLVYK |
| 3721 | VYYGNALDQA ISMWALIISV TSNYSGVVTT VMFLARGIVF MCVEYCPIFF ITGNTLQCIM |
| 3781 | LVYCFLGYFC TCYFGLFCLL NRYFRLTLGV YDYLVSTQEF RYMNSQGLLP PKNSIDAFKL |
| 3841 | NIKLLGVGGK PCIKVATVQS KMSDVKCTSV VLLSVLQQLR VESSSKLWAQ CVQLHNDILL |
| 3901 | AKDTTEAFEK MVSLLSVLLS MQGAVDINKL CEEMLDNRAT LQAIASEFSS LPSYAAFATA |
| 3961 | QEAYEQAVAN GDSEVVLKKL KKSLNVAKSE FDRDAAMQRK LEKMADQAMT QMYKQARSED |
| 4021 | KRAKVTSAMQ TMLFTMLRKL DNDALNNIIN NARDGCVPLN IIPLTTAAKL MVVIPDYNTY |
| 4081 | KNTCDGTTFT YASALWEIQQ VVDADSKIVQ LSEISMDNSP NLAWPLIVTA LRANSAVKLQ |
| 4141 | NNELSPVALR QMSCAAGTTQ TACTDDNALA YYNTTKGGRF VLALLSDLQD LKWARFPKSD |
| 4201 | GTGTIYTELE PPCRFVTDTP KGPKVKYLYF IKGLNNLNRG MVLGSLAATV RLQAGNATEV |
| 4261 | PANSTVLSFC AFAVDAAKAY KDYLASGGQP ITNCVKMLCT HTGTGQAITV TPEANMDQES |
| 4321 | FGGASCCLYC RCHIDHPNPK GFCDLKGKYV QIPTTCANDP VGFTLKNTVC TVCGMWKGYG |
| 4381 | CSCDQLREPM LQSADAQSFL NRVCGVSAAR LTPCGTGTST DVVYRAFDIY NDKVAGFAKF |
| 4441 | LKTNCCRFQE KDEDDNLIDS YFVVKRHTFS NYQHEETIYN LLKDCPAVAK HDFFKFRIDG |
| 4501 | DMVPHISRQR LTKYTMADLV YALRHFDEGN CDTLKEILVT YNCCDDDYFN KKDWYDFVEN |
| 4561 | PDILRVYANL GERVRQALLK TVQFCDAMRN AGIVGVLTLD NQDLNGNWYD FGDFIQTTPG |
| 4621 | SGVPVVDSYY SLLMPILTLT RALTAESHVD TDLTKPYIKW DLLKYDFTEE RLKLFDRYFK |
| 4681 | YWDQTYHPNC VNCLDDRCIL HCANFNVLFS TVFPPTSFGP LVRKIFVDGV PFVVSTGYHF |
| 4741 | RELGVVHNQD VNLHSSRLSF KELLVYAADP AMHAASGNLL LDKRTTCFSV AALTNNVAFQ |
| 4801 | TVKPGNFNKD FYDFAVSKGF FKEGSSVELK HFFFAQDGNA AISDYDYYRY NLPTMCDIRQ |
| 4861 | LLFVVEVVDK YFDCYDGGCI NANQVIVNNL DKSAGFPFNK WGKARLYYDS MSYEDQDALF |
| 4921 | AYTKRNVIPT ITQMNLKYAI SAKNRARTVA GVSICSTMTN RQFHQKLLKS IAATRGATVV |
| 4981 | IGTSKFYGGW HNMLKTVYSD VENPHLMGWD YPKCDRAMPN MLRIMASLVL ARKHTTCCSL |
| 5041 | SHRFYRLANE CAQVLSEMVM CGGSLYVKPG GTSSGDATTA YANSVFNICQ AVTANVNALL |
| 5101 | STDGNKIADK YVRNLQHRLY ECLYRNRDVD TDFVNEFYAY LRKHFSMMIL SDDAVVCFNS |
| 5161 | TYASQGLVAS IKNFKSVLYY QNNVFMSEAK CWTETDLTKG PHEFCSQHTM LVKQGDDYVY |
| 5221 | LPYPDPSRIL GAGCFVDDIV KTDGTLMIER FVSLAIDAYP LTKHPNQEYA DVFHLYLQYI |
| 5281 | RKLHDELTGH MLDMYSVMLT NDNTSRYWEP EFYEAMYTPH TVLQAVGACV LCNSQTSLRC |
| 5341 | GACIRRPFLC CKCCYDHVIS TSHKLVLSVN PYVCNAPGCD VTDVTQLYLG GMSYYCKSHK |
| 5401 | PPISFPLCAN GQVFGLYKNT CVGSDNVTDF NAIATCDWTN AGDYILANTC TERLKLFAAE |
| 5461 | TLKATEETFK LSYGIATVRE VLSDRELHLS WEVGKPRPPL NRNYVFTGYR VTKNSKVQIG |
| 5521 | EYTFEKGDYG DAVVYRGTTT YKLNVGDYFV LTSHTVMPLS APTLVPQEHY VRITGLYPTL |
| 5581 | NISDEFSSNV ANYQKVGMQK YSTLQGPPGT GKSHFAIGLA LYYPSARIVY TACSHAAVDA |
| 5641 | LCEKALKYLP IDKCSRIIPA RARVECFDKF KVNSTLEQYV FCTVNALPET TADIVVFDEI |
| 5701 | SMATNYDLSV VNARLRAKHY VYIGDPAQLP APRTLLTKGT LEPEYFNSVC RLMKTIGPDM |
| 5761 | FLGTCRRCPA EIVDTVSALV YDNKLKAHKD KSAQCFKMFY KGVITHDVSS AINRPQIGVV |
| 5821 | REFLTRNPAW RKAVFISPYN SQNAVASKIL GLPTQTVDSS QGSEYDYVIF TQTTETAHSC |
| 5881 | NVNRFNVAIT RAKVGILCIM SDRDLYDKLQ FTSLEIPRRN VATLQAENVT GLFKDCSKVI |
| 5941 | TGLHPTQAPT HLSVDTKFKT EGLCVDIPGI PKDMTYRRLI SMMGFKMNYQ VNGYPNMFIT |
| 6001 | REEAIRHVRA WIGFDVEGCH ATREAVGTNL PLQLGFSTGV NLVAVPTGYV DTPNNTDFSR |
| 6061 | VSAKPPPGDQ FKHLIPLMYK GLPWNVVRIK IVQMLSDTLK NLSDRVVFVL WAHGFELTSM |
| 6121 | KYFVKIGPER TCCLCDRRAT CFSTASDTYA CWHHSIGFDY VYNPFMIDVQ QWGFTGNLQS |
| 6181 | NHDLYCQVHG NAHVASCDAI MTRCLAVHEC FVKRVDWTIE YPIIGDELKI NAACRKVQHM |
| 6241 | VVKAALLADK FPVLHDIGNP KAIKCVPQAD VEWKFYDAQP CSDKAYKIEE LFYSYATHSD |
| 6301 | KFTDGVCLFW NCNVDRYPAN SIVCRFDTRV LSNLNLPGCD GGSLYVNKHA FHTPAFDKSA |
| 6361 | FVNLKQLPFF YYSDSPCESH GKQVVSDIDY VPLKSATCIT RCNLGGAVCR HHANEYRLYL |
| 6421 | DAYNMMISAG FSLWVYKQFD TYNLWNTFTR LQSLENVAFN VVNKGHFDGQ QGEVPVSIIN |
| 6481 | NTVYTKVDGV DVELFENKTT LPVNVAFELW AKRNIKPVPE VKILNNLGVD IAANTVIWDY |
| 6541 | KRDAPAHIST IGVCSMTDIA KKPTETICAP LTVFFDGRVD GQVDLFRNAR NGVLITEGSV |
| 6601 | KGLQPSVGPK QASLNGVTLI GEAVKTQFNY YKKVDGVVQQ LPETYFTQSR NLQEFKPRSQ |
| 6661 | MEIDFLELAM DEFIERYKLE GYAFEHIVYG DFSHSQLGGL HLLIGLAKRF KESPFELEDF |
| 6721 | IPMDSTVKNY FITDAQTGSS KCVCSVIDLL LDDFVEIIKS QDLSVVSKVV KVTIDYTEIS |
| 6781 | FMLWCKDGHV ETFYPKLQSS QAWQPGVAMP NLYKMQRMLL EKCDLQNYGD SATLPKGIMM |
| 6841 | NVAKYTQLCQ YLNTLTLAVP YNMRVIHFGA GSDKGVAPGT AVLRQWLPTG TLLVDSDLND |
| 6901 | FVSDADSTLI GDCATVHTAN KWDLIISDMY DPKTKNVTKE NDSKEGFFTY ICGFIQQKLA |
| 6961 | LGGSVAIKIT EHSWNADLYK LMGHFAWWTA FVTNVNASSS EAFLIGCNYL GKPREQIDGY |
| 7021 | VMHANYIFWR NTNPIQLSSY SLFDMSKFPL KLRGTAVMSL KEGQINDMIL SLLSKGRLII |
| 7081 | RENNRVVISS DVLVNN |

Top of Form

**Center position:** 4   **Window size:**   **Threshold:**   

Bottom of Form

**Average:** 0.988   **Minimum:** 0.861   **Maximum:** 1.150

**Predicted residue scores:**

| **Position** | **Residue** | **Start** | **End** | **Peptide** | **Score** |
| --- | --- | --- | --- | --- | --- |
| 4 | **L** | 1 | 7 | MESLVPG | 1.0 |
| 5 | **V** | 2 | 8 | ESLVPGF | 1.0 |
| 6 | **P** | 3 | 9 | SLVPGFN | 1.005 |
| 7 | **G** | 4 | 10 | LVPGFNE | 1.008 |
| 8 | **F** | 5 | 11 | VPGFNEK | 1.015 |
| 9 | **N** | 6 | 12 | PGFNEKT | 1.031 |
| 10 | **E** | 7 | 13 | GFNEKTH | 1.039 |
| 11 | **K** | 8 | 14 | FNEKTHV | 1.036 |
| 12 | **T** | 9 | 15 | NEKTHVQ | 1.017 |
| 13 | **H** | 10 | 16 | EKTHVQL | 0.985 |
| 14 | **V** | 11 | 17 | KTHVQLS | 0.954 |
| 15 | **Q** | 12 | 18 | THVQLSL | 0.937 |
| 16 | **L** | 13 | 19 | HVQLSLP | 0.935 |
| 17 | **S** | 14 | 20 | VQLSLPV | 0.936 |
| 18 | **L** | 15 | 21 | QLSLPVL | 0.942 |
| 19 | **P** | 16 | 22 | LSLPVLQ | 0.939 |
| 20 | **V** | 17 | 23 | SLPVLQV | 0.937 |
| 21 | **L** | 18 | 24 | LPVLQVR | 0.944 |
| 22 | **Q** | 19 | 25 | PVLQVRD | 0.956 |
| 23 | **V** | 20 | 26 | VLQVRDV | 0.976 |
| 24 | **R** | 21 | 27 | LQVRDVL | 0.989 |
| 25 | **D** | 22 | 28 | QVRDVLV | 0.99 |
| 26 | **V** | 23 | 29 | VRDVLVR | 0.979 |
| 27 | **L** | 24 | 30 | RDVLVRG | 0.972 |
| 28 | **V** | 25 | 31 | DVLVRGF | 0.969 |
| 29 | **R** | 26 | 32 | VLVRGFG | 0.981 |
| 30 | **G** | 27 | 33 | LVRGFGD | 0.996 |
| 31 | **F** | 28 | 34 | VRGFGDS | 1.004 |
| 32 | **G** | 29 | 35 | RGFGDSV | 1.015 |
| 33 | **D** | 30 | 36 | GFGDSVE | 1.019 |
| 34 | **S** | 31 | 37 | FGDSVEE | 1.022 |
| 35 | **V** | 32 | 38 | GDSVEEV | 1.018 |
| 36 | **E** | 33 | 39 | DSVEEVL | 1.009 |
| 37 | **E** | 34 | 40 | SVEEVLS | 1.001 |
| 38 | **V** | 35 | 41 | VEEVLSE | 0.993 |
| 39 | **L** | 36 | 42 | EEVLSEA | 0.999 |
| 40 | **S** | 37 | 43 | EVLSEAR | 1.011 |
| 41 | **E** | 38 | 44 | VLSEARQ | 1.021 |
| 42 | **A** | 39 | 45 | LSEARQH | 1.026 |
| 43 | **R** | 40 | 46 | SEARQHL | 1.019 |
| 44 | **Q** | 41 | 47 | EARQHLK | 1.011 |
| 45 | **H** | 42 | 48 | ARQHLKD | 1.004 |
| 46 | **L** | 43 | 49 | RQHLKDG | 1.015 |
| 47 | **K** | 44 | 50 | QHLKDGT | 1.036 |
| 48 | **D** | 45 | 51 | HLKDGTC | 1.046 |
| 49 | **G** | 46 | 52 | LKDGTCG | 1.049 |
| 50 | **T** | 47 | 53 | KDGTCGL | 1.029 |
| 51 | **C** | 48 | 54 | DGTCGLV | 0.995 |
| 52 | **G** | 49 | 55 | GTCGLVE | 0.968 |
| 53 | **L** | 50 | 56 | TCGLVEV | 0.951 |
| 54 | **V** | 51 | 57 | CGLVEVE | 0.95 |
| 55 | **E** | 52 | 58 | GLVEVEK | 0.967 |
| 56 | **V** | 53 | 59 | LVEVEKG | 0.992 |
| 57 | **E** | 54 | 60 | VEVEKGV | 1.011 |
| 58 | **K** | 55 | 61 | EVEKGVL | 1.02 |
| 59 | **G** | 56 | 62 | VEKGVLP | 1.018 |
| 60 | **V** | 57 | 63 | EKGVLPQ | 1.009 |
| 61 | **L** | 58 | 64 | KGVLPQL | 1.005 |
| 62 | **P** | 59 | 65 | GVLPQLE | 1.011 |
| 63 | **Q** | 60 | 66 | VLPQLEQ | 1.022 |
| 64 | **L** | 61 | 67 | LPQLEQP | 1.038 |
| 65 | **E** | 62 | 68 | PQLEQPY | 1.049 |
| 66 | **Q** | 63 | 69 | QLEQPYV | 1.047 |
| 67 | **P** | 64 | 70 | LEQPYVF | 1.025 |
| 68 | **Y** | 65 | 71 | EQPYVFI | 0.988 |
| 69 | **V** | 66 | 72 | QPYVFIK | 0.962 |
| 70 | **F** | 67 | 73 | PYVFIKR | 0.951 |
| 71 | **I** | 68 | 74 | YVFIKRS | 0.972 |
| 72 | **K** | 69 | 75 | VFIKRSD | 1.018 |
| 73 | **R** | 70 | 76 | FIKRSDA | 1.051 |
| 74 | **S** | 71 | 77 | IKRSDAR | 1.076 |
| 75 | **D** | 72 | 78 | KRSDART | 1.078 |
| 76 | **A** | 73 | 79 | RSDARTA | 1.065 |
| 77 | **R** | 74 | 80 | SDARTAP | 1.047 |
| 78 | **T** | 75 | 81 | DARTAPH | 1.027 |
| 79 | **A** | 76 | 82 | ARTAPHG | 1.006 |
| 80 | **P** | 77 | 83 | RTAPHGH | 0.983 |
| 81 | **H** | 78 | 84 | TAPHGHV | 0.965 |
| 82 | **G** | 79 | 85 | APHGHVM | 0.939 |
| 83 | **H** | 80 | 86 | PHGHVMV | 0.922 |
| 84 | **V** | 81 | 87 | HGHVMVE | 0.908 |
| 85 | **M** | 82 | 88 | GHVMVEL | 0.899 |
| 86 | **V** | 83 | 89 | HVMVELV | 0.908 |
| 87 | **E** | 84 | 90 | VMVELVA | 0.918 |
| 88 | **L** | 85 | 91 | MVELVAE | 0.929 |
| 89 | **V** | 86 | 92 | VELVAEL | 0.937 |
| 90 | **A** | 87 | 93 | ELVAELE | 0.947 |
| 91 | **E** | 88 | 94 | LVAELEG | 0.961 |
| 92 | **L** | 89 | 95 | VAELEGI | 0.979 |
| 93 | **E** | 90 | 96 | AELEGIQ | 0.992 |
| 94 | **G** | 91 | 97 | ELEGIQY | 0.993 |
| 95 | **I** | 92 | 98 | LEGIQYG | 0.987 |
| 96 | **Q** | 93 | 99 | EGIQYGR | 0.979 |
| 97 | **Y** | 94 | 100 | GIQYGRS | 0.995 |
| 98 | **G** | 95 | 101 | IQYGRSG | 1.026 |
| 99 | **R** | 96 | 102 | QYGRSGE | 1.061 |
| 100 | **S** | 97 | 103 | YGRSGET | 1.094 |
| 101 | **G** | 98 | 104 | GRSGETL | 1.096 |
| 102 | **E** | 99 | 105 | RSGETLG | 1.074 |
| 103 | **T** | 100 | 106 | SGETLGV | 1.038 |
| 104 | **L** | 101 | 107 | GETLGVL | 0.993 |
| 105 | **G** | 102 | 108 | ETLGVLV | 0.959 |
| 106 | **V** | 103 | 109 | TLGVLVP | 0.938 |
| 107 | **L** | 104 | 110 | LGVLVPH | 0.93 |
| 108 | **V** | 105 | 111 | GVLVPHV | 0.93 |
| 109 | **P** | 106 | 112 | VLVPHVG | 0.939 |
| 110 | **H** | 107 | 113 | LVPHVGE | 0.955 |
| 111 | **V** | 108 | 114 | VPHVGEI | 0.972 |
| 112 | **G** | 109 | 115 | PHVGEIP | 0.989 |
| 113 | **E** | 110 | 116 | HVGEIPV | 0.992 |
| 114 | **I** | 111 | 117 | VGEIPVA | 0.979 |
| 115 | **P** | 112 | 118 | GEIPVAY | 0.959 |
| 116 | **V** | 113 | 119 | EIPVAYR | 0.944 |
| 117 | **A** | 114 | 120 | IPVAYRK | 0.946 |
| 118 | **Y** | 115 | 121 | PVAYRKV | 0.96 |
| 119 | **R** | 116 | 122 | VAYRKVL | 0.977 |
| 120 | **K** | 117 | 123 | AYRKVLL | 0.984 |
| 121 | **V** | 118 | 124 | YRKVLLR | 0.978 |
| 122 | **L** | 119 | 125 | RKVLLRK | 0.981 |
| 123 | **L** | 120 | 126 | KVLLRKN | 0.996 |
| 124 | **R** | 121 | 127 | VLLRKNG | 1.026 |
| 125 | **K** | 122 | 128 | LLRKNGN | 1.066 |
| 126 | **N** | 123 | 129 | LRKNGNK | 1.095 |
| 127 | **G** | 124 | 130 | RKNGNKG | 1.107 |
| 128 | **N** | 125 | 131 | KNGNKGA | 1.102 |
| 129 | **K** | 126 | 132 | NGNKGAG | 1.086 |
| 130 | **G** | 127 | 133 | GNKGAGG | 1.067 |
| 131 | **A** | 128 | 134 | NKGAGGH | 1.049 |
| 132 | **G** | 129 | 135 | KGAGGHS | 1.03 |
| 133 | **G** | 130 | 136 | GAGGHSY | 1.011 |
| 134 | **H** | 131 | 137 | AGGHSYG | 0.987 |
| 135 | **S** | 132 | 138 | GGHSYGA | 0.971 |
| 136 | **Y** | 133 | 139 | GHSYGAD | 0.963 |
| 137 | **G** | 134 | 140 | HSYGADL | 0.96 |
| 138 | **A** | 135 | 141 | SYGADLK | 0.974 |
| 139 | **D** | 136 | 142 | YGADLKS | 0.986 |
| 140 | **L** | 137 | 143 | GADLKSF | 0.996 |
| 141 | **K** | 138 | 144 | ADLKSFD | 1.004 |
| 142 | **S** | 139 | 145 | DLKSFDL | 0.995 |
| 143 | **F** | 140 | 146 | LKSFDLG | 0.985 |
| 144 | **D** | 141 | 147 | KSFDLGD | 0.982 |
| 145 | **L** | 142 | 148 | SFDLGDE | 0.987 |
| 146 | **G** | 143 | 149 | FDLGDEL | 1.0 |
| 147 | **D** | 144 | 150 | DLGDELG | 1.013 |
| 148 | **E** | 145 | 151 | LGDELGT | 1.022 |
| 149 | **L** | 146 | 152 | GDELGTD | 1.024 |
| 150 | **G** | 147 | 153 | DELGTDP | 1.034 |
| 151 | **T** | 148 | 154 | ELGTDPY | 1.039 |
| 152 | **D** | 149 | 155 | LGTDPYE | 1.038 |
| 153 | **P** | 150 | 156 | GTDPYED | 1.042 |
| 154 | **Y** | 151 | 157 | TDPYEDF | 1.028 |
| 155 | **E** | 152 | 158 | DPYEDFQ | 1.024 |
| 156 | **D** | 153 | 159 | PYEDFQE | 1.026 |
| 157 | **F** | 154 | 160 | YEDFQEN | 1.019 |
| 158 | **Q** | 155 | 161 | EDFQENW | 1.021 |
| 159 | **E** | 156 | 162 | DFQENWN | 1.018 |
| 160 | **N** | 157 | 163 | FQENWNT | 1.01 |
| 161 | **W** | 158 | 164 | QENWNTK | 1.011 |
| 162 | **N** | 159 | 165 | ENWNTKH | 1.017 |
| 163 | **T** | 160 | 166 | NWNTKHS | 1.026 |
| 164 | **K** | 161 | 167 | WNTKHSS | 1.043 |
| 165 | **H** | 162 | 168 | NTKHSSG | 1.053 |
| 166 | **S** | 163 | 169 | TKHSSGV | 1.059 |
| 167 | **S** | 164 | 170 | KHSSGVT | 1.063 |
| 168 | **G** | 165 | 171 | HSSGVTR | 1.052 |
| 169 | **V** | 166 | 172 | SSGVTRE | 1.044 |
| 170 | **T** | 167 | 173 | SGVTREL | 1.034 |
| 171 | **R** | 168 | 174 | GVTRELM | 1.013 |
| 172 | **E** | 169 | 175 | VTRELMR | 0.999 |
| 173 | **L** | 170 | 176 | TRELMRE | 0.985 |
| 174 | **M** | 171 | 177 | RELMREL | 0.973 |
| 175 | **R** | 172 | 178 | ELMRELN | 0.982 |
| 176 | **E** | 173 | 179 | LMRELNG | 0.998 |
| 177 | **L** | 174 | 180 | MRELNGG | 1.015 |
| 178 | **N** | 175 | 181 | RELNGGA | 1.03 |
| 179 | **G** | 176 | 182 | ELNGGAY | 1.031 |
| 180 | **G** | 177 | 183 | LNGGAYT | 1.02 |
| 181 | **A** | 178 | 184 | NGGAYTR | 1.007 |
| 182 | **Y** | 179 | 185 | GGAYTRY | 0.995 |
| 183 | **T** | 180 | 186 | GAYTRYV | 0.987 |
| 184 | **R** | 181 | 187 | AYTRYVD | 0.988 |
| 185 | **Y** | 182 | 188 | YTRYVDN | 0.995 |
| 186 | **V** | 183 | 189 | TRYVDNN | 1.007 |
| 187 | **D** | 184 | 190 | RYVDNNF | 1.019 |
| 188 | **N** | 185 | 191 | YVDNNFC | 1.015 |
| 189 | **N** | 186 | 192 | VDNNFCG | 1.001 |
| 190 | **F** | 187 | 193 | DNNFCGP | 0.985 |
| 191 | **C** | 188 | 194 | NNFCGPD | 0.978 |
| 192 | **G** | 189 | 195 | NFCGPDG | 0.994 |
| 193 | **P** | 190 | 196 | FCGPDGY | 1.01 |
| 194 | **D** | 191 | 197 | CGPDGYP | 1.015 |
| 195 | **G** | 192 | 198 | GPDGYPL | 1.008 |
| 196 | **Y** | 193 | 199 | PDGYPLE | 0.985 |
| 197 | **P** | 194 | 200 | DGYPLEC | 0.961 |
| 198 | **L** | 195 | 201 | GYPLECI | 0.942 |
| 199 | **E** | 196 | 202 | YPLECIK | 0.935 |
| 200 | **C** | 197 | 203 | PLECIKD | 0.944 |
| 201 | **I** | 198 | 204 | LECIKDL | 0.964 |
| 202 | **K** | 199 | 205 | ECIKDLL | 0.988 |
| 203 | **D** | 200 | 206 | CIKDLLA | 0.996 |
| 204 | **L** | 201 | 207 | IKDLLAR | 0.983 |
| 205 | **L** | 202 | 208 | KDLLARA | 0.969 |
| 206 | **A** | 203 | 209 | DLLARAG | 0.962 |
| 207 | **R** | 204 | 210 | LLARAGK | 0.971 |
| 208 | **A** | 205 | 211 | LARAGKA | 1.001 |
| 209 | **G** | 206 | 212 | ARAGKAS | 1.018 |
| 210 | **K** | 207 | 213 | RAGKASC | 1.023 |
| 211 | **A** | 208 | 214 | AGKASCT | 1.01 |
| 212 | **S** | 209 | 215 | GKASCTL | 0.984 |
| 213 | **C** | 210 | 216 | KASCTLS | 0.972 |
| 214 | **T** | 211 | 217 | ASCTLSE | 0.975 |
| 215 | **L** | 212 | 218 | SCTLSEQ | 0.992 |
| 216 | **S** | 213 | 219 | CTLSEQL | 1.014 |
| 217 | **E** | 214 | 220 | TLSEQLD | 1.021 |
| 218 | **Q** | 215 | 221 | LSEQLDF | 1.008 |
| 219 | **L** | 216 | 222 | SEQLDFI | 0.981 |
| 220 | **D** | 217 | 223 | EQLDFID | 0.962 |
| 221 | **F** | 218 | 224 | QLDFIDT | 0.958 |
| 222 | **I** | 219 | 225 | LDFIDTK | 0.978 |
| 223 | **D** | 220 | 226 | DFIDTKR | 1.014 |
| 224 | **T** | 221 | 227 | FIDTKRG | 1.041 |
| 225 | **K** | 222 | 228 | IDTKRGV | 1.049 |
| 226 | **R** | 223 | 229 | DTKRGVY | 1.03 |
| 227 | **G** | 224 | 230 | TKRGVYC | 0.995 |
| 228 | **V** | 225 | 231 | KRGVYCC | 0.953 |
| 229 | **Y** | 226 | 232 | RGVYCCR | 0.926 |
| 230 | **C** | 227 | 233 | GVYCCRE | 0.927 |
| 231 | **C** | 228 | 234 | VYCCREH | 0.939 |
| 232 | **R** | 229 | 235 | YCCREHE | 0.965 |
| 233 | **E** | 230 | 236 | CCREHEH | 0.981 |
| 234 | **H** | 231 | 237 | CREHEHE | 0.979 |
| 235 | **E** | 232 | 238 | REHEHEI | 0.968 |
| 236 | **H** | 233 | 239 | EHEHEIA | 0.95 |
| 237 | **E** | 234 | 240 | HEHEIAW | 0.924 |
| 238 | **I** | 235 | 241 | EHEIAWY | 0.905 |
| 239 | **A** | 236 | 242 | HEIAWYT | 0.901 |
| 240 | **W** | 237 | 243 | EIAWYTE | 0.912 |
| 241 | **Y** | 238 | 244 | IAWYTER | 0.949 |
| 242 | **T** | 239 | 245 | AWYTERS | 1.001 |
| 243 | **E** | 240 | 246 | WYTERSE | 1.046 |
| 244 | **R** | 241 | 247 | YTERSEK | 1.078 |
| 245 | **S** | 242 | 248 | TERSEKS | 1.097 |
| 246 | **E** | 243 | 249 | ERSEKSY | 1.087 |
| 247 | **K** | 244 | 250 | RSEKSYE | 1.064 |
| 248 | **S** | 245 | 251 | SEKSYEL | 1.034 |
| 249 | **Y** | 246 | 252 | EKSYELQ | 1.002 |
| 250 | **E** | 247 | 253 | KSYELQT | 0.99 |
| 251 | **L** | 248 | 254 | SYELQTP | 0.997 |
| 252 | **Q** | 249 | 255 | YELQTPF | 1.01 |
| 253 | **T** | 250 | 256 | ELQTPFE | 1.018 |
| 254 | **P** | 251 | 257 | LQTPFEI | 1.015 |
| 255 | **F** | 252 | 258 | QTPFEIK | 1.001 |
| 256 | **E** | 253 | 259 | TPFEIKL | 0.99 |
| 257 | **I** | 254 | 260 | PFEIKLA | 0.987 |
| 258 | **K** | 255 | 261 | FEIKLAK | 0.993 |
| 259 | **L** | 256 | 262 | EIKLAKK | 1.002 |
| 260 | **A** | 257 | 263 | IKLAKKF | 1.008 |
| 261 | **K** | 258 | 264 | KLAKKFD | 1.021 |
| 262 | **K** | 259 | 265 | LAKKFDT | 1.028 |
| 263 | **F** | 260 | 266 | AKKFDTF | 1.023 |
| 264 | **D** | 261 | 267 | KKFDTFN | 1.026 |
| 265 | **T** | 262 | 268 | KFDTFNG | 1.022 |
| 266 | **F** | 263 | 269 | FDTFNGE | 1.02 |
| 267 | **N** | 264 | 270 | DTFNGEC | 1.029 |
| 268 | **G** | 265 | 271 | TFNGECP | 1.038 |
| 269 | **E** | 266 | 272 | FNGECPN | 1.035 |
| 270 | **C** | 267 | 273 | NGECPNF | 1.026 |
| 271 | **P** | 268 | 274 | GECPNFV | 1.012 |
| 272 | **N** | 269 | 275 | ECPNFVF | 0.982 |
| 273 | **F** | 270 | 276 | CPNFVFP | 0.956 |
| 274 | **V** | 271 | 277 | PNFVFPL | 0.941 |
| 275 | **F** | 272 | 278 | NFVFPLN | 0.935 |
| 276 | **P** | 273 | 279 | FVFPLNS | 0.948 |
| 277 | **L** | 274 | 280 | VFPLNSI | 0.963 |
| 278 | **N** | 275 | 281 | FPLNSII | 0.97 |
| 279 | **S** | 276 | 282 | PLNSIIK | 0.976 |
| 280 | **I** | 277 | 283 | LNSIIKT | 0.974 |
| 281 | **I** | 278 | 284 | NSIIKTI | 0.981 |
| 282 | **K** | 279 | 285 | SIIKTIQ | 1.001 |
| 283 | **T** | 280 | 286 | IIKTIQP | 1.015 |
| 284 | **I** | 281 | 287 | IKTIQPR | 1.027 |
| 285 | **Q** | 282 | 288 | KTIQPRV | 1.032 |
| 286 | **P** | 283 | 289 | TIQPRVE | 1.028 |
| 287 | **R** | 284 | 290 | IQPRVEK | 1.028 |
| 288 | **V** | 285 | 291 | QPRVEKK | 1.034 |
| 289 | **E** | 286 | 292 | PRVEKKK | 1.047 |
| 290 | **K** | 287 | 293 | RVEKKKL | 1.055 |
| 291 | **K** | 288 | 294 | VEKKKLD | 1.061 |
| 292 | **K** | 289 | 295 | EKKKLDG | 1.059 |
| 293 | **L** | 290 | 296 | KKKLDGF | 1.041 |
| 294 | **D** | 291 | 297 | KKLDGFM | 1.02 |
| 295 | **G** | 292 | 298 | KLDGFMG | 0.997 |
| 296 | **F** | 293 | 299 | LDGFMGR | 0.976 |
| 297 | **M** | 294 | 300 | DGFMGRI | 0.971 |
| 298 | **G** | 295 | 301 | GFMGRIR | 0.984 |
| 299 | **R** | 296 | 302 | FMGRIRS | 0.999 |
| 300 | **I** | 297 | 303 | MGRIRSV | 1.009 |
| 301 | **R** | 298 | 304 | GRIRSVY | 1.009 |
| 302 | **S** | 299 | 305 | RIRSVYP | 0.993 |
| 303 | **V** | 300 | 306 | IRSVYPV | 0.968 |
| 304 | **Y** | 301 | 307 | RSVYPVA | 0.951 |
| 305 | **P** | 302 | 308 | SVYPVAS | 0.946 |
| 306 | **V** | 303 | 309 | VYPVASP | 0.955 |
| 307 | **A** | 304 | 310 | YPVASPN | 0.983 |
| 308 | **S** | 305 | 311 | PVASPNE | 1.016 |
| 309 | **P** | 306 | 312 | VASPNEC | 1.035 |
| 310 | **N** | 307 | 313 | ASPNECN | 1.044 |
| 311 | **E** | 308 | 314 | SPNECNQ | 1.036 |
| 312 | **C** | 309 | 315 | PNECNQM | 1.011 |
| 313 | **N** | 310 | 316 | NECNQMC | 0.989 |
| 314 | **Q** | 311 | 317 | ECNQMCL | 0.968 |
| 315 | **M** | 312 | 318 | CNQMCLS | 0.953 |
| 316 | **C** | 313 | 319 | NQMCLST | 0.961 |
| 317 | **L** | 314 | 320 | QMCLSTL | 0.974 |
| 318 | **S** | 315 | 321 | MCLSTLM | 0.983 |
| 319 | **T** | 316 | 322 | CLSTLMK | 0.991 |
| 320 | **L** | 317 | 323 | LSTLMKC | 0.982 |
| 321 | **M** | 318 | 324 | STLMKCD | 0.969 |
| 322 | **K** | 319 | 325 | TLMKCDH | 0.968 |
| 323 | **C** | 320 | 326 | LMKCDHC | 0.955 |
| 324 | **D** | 321 | 327 | MKCDHCG | 0.952 |
| 325 | **H** | 322 | 328 | KCDHCGE | 0.962 |
| 326 | **C** | 323 | 329 | CDHCGET | 0.974 |
| 327 | **G** | 324 | 330 | DHCGETS | 1.007 |
| 328 | **E** | 325 | 331 | HCGETSW | 1.028 |
| 329 | **T** | 326 | 332 | CGETSWQ | 1.035 |
| 330 | **S** | 327 | 333 | GETSWQT | 1.034 |
| 331 | **W** | 328 | 334 | ETSWQTG | 1.028 |
| 332 | **Q** | 329 | 335 | TSWQTGD | 1.04 |
| 333 | **T** | 330 | 336 | SWQTGDF | 1.049 |
| 334 | **G** | 331 | 337 | WQTGDFV | 1.049 |
| 335 | **D** | 332 | 338 | QTGDFVK | 1.038 |
| 336 | **F** | 333 | 339 | TGDFVKA | 1.013 |
| 337 | **V** | 334 | 340 | GDFVKAT | 0.997 |
| 338 | **K** | 335 | 341 | DFVKATC | 0.992 |
| 339 | **A** | 336 | 342 | FVKATCE | 0.985 |
| 340 | **T** | 337 | 343 | VKATCEF | 0.972 |
| 341 | **C** | 338 | 344 | KATCEFC | 0.955 |
| 342 | **E** | 339 | 345 | ATCEFCG | 0.941 |
| 343 | **F** | 340 | 346 | TCEFCGT | 0.943 |
| 344 | **C** | 341 | 347 | CEFCGTE | 0.965 |
| 345 | **G** | 342 | 348 | EFCGTEN | 0.998 |
| 346 | **T** | 343 | 349 | FCGTENL | 1.022 |
| 347 | **E** | 344 | 350 | CGTENLT | 1.035 |
| 348 | **N** | 345 | 351 | GTENLTK | 1.037 |
| 349 | **L** | 346 | 352 | TENLTKE | 1.036 |
| 350 | **T** | 347 | 353 | ENLTKEG | 1.045 |
| 351 | **K** | 348 | 354 | NLTKEGA | 1.054 |
| 352 | **E** | 349 | 355 | LTKEGAT | 1.061 |
| 353 | **G** | 350 | 356 | TKEGATT | 1.06 |
| 354 | **A** | 351 | 357 | KEGATTC | 1.049 |
| 355 | **T** | 352 | 358 | EGATTCG | 1.031 |
| 356 | **T** | 353 | 359 | GATTCGY | 1.009 |
| 357 | **C** | 354 | 360 | ATTCGYL | 0.983 |
| 358 | **G** | 355 | 361 | TTCGYLP | 0.97 |
| 359 | **Y** | 356 | 362 | TCGYLPQ | 0.98 |
| 360 | **L** | 357 | 363 | CGYLPQN | 1.002 |
| 361 | **P** | 358 | 364 | GYLPQNA | 1.029 |
| 362 | **Q** | 359 | 365 | YLPQNAV | 1.037 |
| 363 | **N** | 360 | 366 | LPQNAVV | 1.015 |
| 364 | **A** | 361 | 367 | PQNAVVK | 0.992 |
| 365 | **V** | 362 | 368 | QNAVVKI | 0.966 |
| 366 | **V** | 363 | 369 | NAVVKIY | 0.946 |
| 367 | **K** | 364 | 370 | AVVKIYC | 0.938 |
| 368 | **I** | 365 | 371 | VVKIYCP | 0.919 |
| 369 | **Y** | 366 | 372 | VKIYCPA | 0.907 |
| 370 | **C** | 367 | 373 | KIYCPAC | 0.905 |
| 371 | **P** | 368 | 374 | IYCPACH | 0.91 |
| 372 | **A** | 369 | 375 | YCPACHN | 0.929 |
| 373 | **C** | 370 | 376 | CPACHNS | 0.957 |
| 374 | **H** | 371 | 377 | PACHNSE | 0.987 |
| 375 | **N** | 372 | 378 | ACHNSEV | 1.015 |
| 376 | **S** | 373 | 379 | CHNSEVG | 1.04 |
| 377 | **E** | 374 | 380 | HNSEVGP | 1.044 |
| 378 | **V** | 375 | 381 | NSEVGPE | 1.041 |
| 379 | **G** | 376 | 382 | SEVGPEH | 1.036 |
| 380 | **P** | 377 | 383 | EVGPEHS | 1.021 |
| 381 | **E** | 378 | 384 | VGPEHSL | 1.008 |
| 382 | **H** | 379 | 385 | GPEHSLA | 0.989 |
| 383 | **S** | 380 | 386 | PEHSLAE | 0.967 |
| 384 | **L** | 381 | 387 | EHSLAEY | 0.953 |
| 385 | **A** | 382 | 388 | HSLAEYH | 0.944 |
| 386 | **E** | 383 | 389 | SLAEYHN | 0.945 |
| 387 | **Y** | 384 | 390 | LAEYHNE | 0.958 |
| 388 | **H** | 385 | 391 | AEYHNES | 0.987 |
| 389 | **N** | 386 | 392 | EYHNESG | 1.021 |
| 390 | **E** | 387 | 393 | YHNESGL | 1.049 |
| 391 | **S** | 388 | 394 | HNESGLK | 1.067 |
| 392 | **G** | 389 | 395 | NESGLKT | 1.062 |
| 393 | **L** | 390 | 396 | ESGLKTI | 1.042 |
| 394 | **K** | 391 | 397 | SGLKTIL | 1.024 |
| 395 | **T** | 392 | 398 | GLKTILR | 1.003 |
| 396 | **I** | 393 | 399 | LKTILRK | 0.993 |
| 397 | **L** | 394 | 400 | KTILRKG | 1.007 |
| 398 | **R** | 395 | 401 | TILRKGG | 1.033 |
| 399 | **K** | 396 | 402 | ILRKGGR | 1.064 |
| 400 | **G** | 397 | 403 | LRKGGRT | 1.089 |
| 401 | **G** | 398 | 404 | RKGGRTI | 1.082 |
| 402 | **R** | 399 | 405 | KGGRTIA | 1.049 |
| 403 | **T** | 400 | 406 | GGRTIAF | 1.007 |
| 404 | **I** | 401 | 407 | GRTIAFG | 0.968 |
| 405 | **A** | 402 | 408 | RTIAFGG | 0.953 |
| 406 | **F** | 403 | 409 | TIAFGGC | 0.953 |
| 407 | **G** | 404 | 410 | IAFGGCV | 0.961 |
| 408 | **G** | 405 | 411 | AFGGCVF | 0.961 |
| 409 | **C** | 406 | 412 | FGGCVFS | 0.945 |
| 410 | **V** | 407 | 413 | GGCVFSY | 0.933 |
| 411 | **F** | 408 | 414 | GCVFSYV | 0.921 |
| 412 | **S** | 409 | 415 | CVFSYVG | 0.919 |
| 413 | **Y** | 410 | 416 | VFSYVGC | 0.921 |
| 414 | **V** | 411 | 417 | FSYVGCH | 0.921 |
| 415 | **G** | 412 | 418 | SYVGCHN | 0.925 |
| 416 | **C** | 413 | 419 | YVGCHNK | 0.938 |
| 417 | **H** | 414 | 420 | VGCHNKC | 0.955 |
| 418 | **N** | 415 | 421 | GCHNKCA | 0.966 |
| 419 | **K** | 416 | 422 | CHNKCAY | 0.961 |
| 420 | **C** | 417 | 423 | HNKCAYW | 0.93 |
| 421 | **A** | 418 | 424 | NKCAYWV | 0.901 |
| 422 | **Y** | 419 | 425 | KCAYWVP | 0.888 |
| 423 | **W** | 420 | 426 | CAYWVPR | 0.898 |
| 424 | **V** | 421 | 427 | AYWVPRA | 0.94 |
| 425 | **P** | 422 | 428 | YWVPRAS | 0.978 |
| 426 | **R** | 423 | 429 | WVPRASA | 1.002 |
| 427 | **A** | 424 | 430 | VPRASAN | 1.008 |
| 428 | **S** | 425 | 431 | PRASANI | 0.996 |
| 429 | **A** | 426 | 432 | RASANIG | 0.985 |
| 430 | **N** | 427 | 433 | ASANIGC | 0.971 |
| 431 | **I** | 428 | 434 | SANIGCN | 0.964 |
| 432 | **G** | 429 | 435 | ANIGCNH | 0.958 |
| 433 | **C** | 430 | 436 | NIGCNHT | 0.959 |
| 434 | **N** | 431 | 437 | IGCNHTG | 0.971 |
| 435 | **H** | 432 | 438 | GCNHTGV | 0.983 |
| 436 | **T** | 433 | 439 | CNHTGVV | 0.992 |
| 437 | **G** | 434 | 440 | NHTGVVG | 0.993 |
| 438 | **V** | 435 | 441 | HTGVVGE | 0.992 |
| 439 | **V** | 436 | 442 | TGVVGEG | 1.005 |
| 440 | **G** | 437 | 443 | GVVGEGS | 1.036 |
| 441 | **E** | 438 | 444 | VVGEGSE | 1.071 |
| 442 | **G** | 439 | 445 | VGEGSEG | 1.1 |
| 443 | **S** | 440 | 446 | GEGSEGL | 1.104 |
| 444 | **E** | 441 | 447 | EGSEGLN | 1.083 |
| 445 | **G** | 442 | 448 | GSEGLND | 1.055 |
| 446 | **L** | 443 | 449 | SEGLNDN | 1.028 |
| 447 | **N** | 444 | 450 | EGLNDNL | 1.011 |
| 448 | **D** | 445 | 451 | GLNDNLL | 1.002 |
| 449 | **N** | 446 | 452 | LNDNLLE | 0.99 |
| 450 | **L** | 447 | 453 | NDNLLEI | 0.973 |
| 451 | **L** | 448 | 454 | DNLLEIL | 0.957 |
| 452 | **E** | 449 | 455 | NLLEILQ | 0.948 |
| 453 | **I** | 450 | 456 | LLEILQK | 0.955 |
| 454 | **L** | 451 | 457 | LEILQKE | 0.982 |
| 455 | **Q** | 452 | 458 | EILQKEK | 1.016 |
| 456 | **K** | 453 | 459 | ILQKEKV | 1.044 |
| 457 | **E** | 454 | 460 | LQKEKVN | 1.051 |
| 458 | **K** | 455 | 461 | QKEKVNI | 1.04 |
| 459 | **V** | 456 | 462 | KEKVNIN | 1.011 |
| 460 | **N** | 457 | 463 | EKVNINI | 0.98 |
| 461 | **I** | 458 | 464 | KVNINIV | 0.959 |
| 462 | **N** | 459 | 465 | VNINIVG | 0.946 |
| 463 | **I** | 460 | 466 | NINIVGD | 0.953 |
| 464 | **V** | 461 | 467 | INIVGDF | 0.968 |
| 465 | **G** | 462 | 468 | NIVGDFK | 0.99 |
| 466 | **D** | 463 | 469 | IVGDFKL | 1.006 |
| 467 | **F** | 464 | 470 | VGDFKLN | 1.007 |
| 468 | **K** | 465 | 471 | GDFKLNE | 1.015 |
| 469 | **L** | 466 | 472 | DFKLNEE | 1.014 |
| 470 | **N** | 467 | 473 | FKLNEEI | 1.013 |
| 471 | **E** | 468 | 474 | KLNEEIA | 1.011 |
| 472 | **E** | 469 | 475 | LNEEIAI | 0.984 |
| 473 | **I** | 470 | 476 | NEEIAII | 0.948 |
| 474 | **A** | 471 | 477 | EEIAIIL | 0.918 |
| 475 | **I** | 472 | 478 | EIAIILA | 0.899 |
| 476 | **I** | 473 | 479 | IAIILAS | 0.897 |
| 477 | **L** | 474 | 480 | AIILASF | 0.909 |
| 478 | **A** | 475 | 481 | IILASFS | 0.92 |
| 479 | **S** | 476 | 482 | ILASFSA | 0.933 |
| 480 | **F** | 477 | 483 | LASFSAS | 0.95 |
| 481 | **S** | 478 | 484 | ASFSAST | 0.973 |
| 482 | **A** | 479 | 485 | SFSASTS | 1.003 |
| 483 | **S** | 480 | 486 | FSASTSA | 1.022 |
| 484 | **T** | 481 | 487 | SASTSAF | 1.024 |
| 485 | **S** | 482 | 488 | ASTSAFV | 1.009 |
| 486 | **A** | 483 | 489 | STSAFVE | 0.985 |
| 487 | **F** | 484 | 490 | TSAFVET | 0.973 |
| 488 | **V** | 485 | 491 | SAFVETV | 0.974 |
| 489 | **E** | 486 | 492 | AFVETVK | 0.994 |
| 490 | **T** | 487 | 493 | FVETVKG | 1.014 |
| 491 | **V** | 488 | 494 | VETVKGL | 1.024 |
| 492 | **K** | 489 | 495 | ETVKGLD | 1.025 |
| 493 | **G** | 490 | 496 | TVKGLDY | 1.01 |
| 494 | **L** | 491 | 497 | VKGLDYK | 0.995 |
| 495 | **D** | 492 | 498 | KGLDYKA | 0.984 |
| 496 | **Y** | 493 | 499 | GLDYKAF | 0.974 |
| 497 | **K** | 494 | 500 | LDYKAFK | 0.98 |
| 498 | **A** | 495 | 501 | DYKAFKQ | 0.984 |
| 499 | **F** | 496 | 502 | YKAFKQI | 0.985 |
| 500 | **K** | 497 | 503 | KAFKQIV | 0.988 |
| 501 | **Q** | 498 | 504 | AFKQIVE | 0.981 |
| 502 | **I** | 499 | 505 | FKQIVES | 0.977 |
| 503 | **V** | 500 | 506 | KQIVESC | 0.98 |
| 504 | **E** | 501 | 507 | QIVESCG | 0.99 |
| 505 | **S** | 502 | 508 | IVESCGN | 1.001 |
| 506 | **C** | 503 | 509 | VESCGNF | 1.003 |
| 507 | **G** | 504 | 510 | ESCGNFK | 1.007 |
| 508 | **N** | 505 | 511 | SCGNFKV | 1.0 |
| 509 | **F** | 506 | 512 | CGNFKVT | 0.998 |
| 510 | **K** | 507 | 513 | GNFKVTK | 1.013 |
| 511 | **V** | 508 | 514 | NFKVTKG | 1.028 |
| 512 | **T** | 509 | 515 | FKVTKGK | 1.052 |
| 513 | **K** | 510 | 516 | KVTKGKA | 1.074 |
| 514 | **G** | 511 | 517 | VTKGKAK | 1.084 |
| 515 | **K** | 512 | 518 | TKGKAKK | 1.086 |
| 516 | **A** | 513 | 519 | KGKAKKG | 1.079 |
| 517 | **K** | 514 | 520 | GKAKKGA | 1.067 |
| 518 | **K** | 515 | 521 | KAKKGAW | 1.045 |
| 519 | **G** | 516 | 522 | AKKGAWN | 1.016 |
| 520 | **A** | 517 | 523 | KKGAWNI | 0.987 |
| 521 | **W** | 518 | 524 | KGAWNIG | 0.97 |
| 522 | **N** | 519 | 525 | GAWNIGE | 0.974 |
| 523 | **I** | 520 | 526 | AWNIGEQ | 1.004 |
| 524 | **G** | 521 | 527 | WNIGEQK | 1.041 |
| 525 | **E** | 522 | 528 | NIGEQKS | 1.073 |
| 526 | **Q** | 523 | 529 | IGEQKSI | 1.081 |
| 527 | **K** | 524 | 530 | GEQKSIL | 1.062 |
| 528 | **S** | 525 | 531 | EQKSILS | 1.034 |
| 529 | **I** | 526 | 532 | QKSILSP | 1.008 |
| 530 | **L** | 527 | 533 | KSILSPL | 0.999 |
| 531 | **S** | 528 | 534 | SILSPLY | 0.995 |
| 532 | **P** | 529 | 535 | ILSPLYA | 0.984 |
| 533 | **L** | 530 | 536 | LSPLYAF | 0.96 |
| 534 | **Y** | 531 | 537 | SPLYAFA | 0.931 |
| 535 | **A** | 532 | 538 | PLYAFAS | 0.923 |
| 536 | **F** | 533 | 539 | LYAFASE | 0.934 |
| 537 | **A** | 534 | 540 | YAFASEA | 0.957 |
| 538 | **S** | 535 | 541 | AFASEAA | 0.981 |
| 539 | **E** | 536 | 542 | FASEAAR | 0.983 |
| 540 | **A** | 537 | 543 | ASEAARV | 0.969 |
| 541 | **A** | 538 | 544 | SEAARVV | 0.952 |
| 542 | **R** | 539 | 545 | EAARVVR | 0.94 |
| 543 | **V** | 540 | 546 | AARVVRS | 0.946 |
| 544 | **V** | 541 | 547 | ARVVRSI | 0.957 |
| 545 | **R** | 542 | 548 | RVVRSIF | 0.968 |
| 546 | **S** | 543 | 549 | VVRSIFS | 0.975 |
| 547 | **I** | 544 | 550 | VRSIFSR | 0.973 |
| 548 | **F** | 545 | 551 | RSIFSRT | 0.982 |
| 549 | **S** | 546 | 552 | SIFSRTL | 0.996 |
| 550 | **R** | 547 | 553 | IFSRTLE | 1.008 |
| 551 | **T** | 548 | 554 | FSRTLET | 1.022 |
| 552 | **L** | 549 | 555 | SRTLETA | 1.025 |
| 553 | **E** | 550 | 556 | RTLETAQ | 1.028 |
| 554 | **T** | 551 | 557 | TLETAQN | 1.037 |
| 555 | **A** | 552 | 558 | LETAQNS | 1.045 |
| 556 | **Q** | 553 | 559 | ETAQNSV | 1.05 |
| 557 | **N** | 554 | 560 | TAQNSVR | 1.043 |
| 558 | **S** | 555 | 561 | AQNSVRV | 1.02 |
| 559 | **V** | 556 | 562 | QNSVRVL | 0.991 |
| 560 | **R** | 557 | 563 | NSVRVLQ | 0.968 |
| 561 | **V** | 558 | 564 | SVRVLQK | 0.965 |
| 562 | **L** | 559 | 565 | VRVLQKA | 0.975 |
| 563 | **Q** | 560 | 566 | RVLQKAA | 0.986 |
| 564 | **K** | 561 | 567 | VLQKAAI | 0.986 |
| 565 | **A** | 562 | 568 | LQKAAIT | 0.965 |
| 566 | **A** | 563 | 569 | QKAAITI | 0.94 |
| 567 | **I** | 564 | 570 | KAAITIL | 0.923 |
| 568 | **T** | 565 | 571 | AAITILD | 0.927 |
| 569 | **I** | 566 | 572 | AITILDG | 0.947 |
| 570 | **L** | 567 | 573 | ITILDGI | 0.977 |
| 571 | **D** | 568 | 574 | TILDGIS | 1.008 |
| 572 | **G** | 569 | 575 | ILDGISQ | 1.024 |
| 573 | **I** | 570 | 576 | LDGISQY | 1.027 |
| 574 | **S** | 571 | 577 | DGISQYS | 1.019 |
| 575 | **Q** | 572 | 578 | GISQYSL | 1.0 |
| 576 | **Y** | 573 | 579 | ISQYSLR | 0.977 |
| 577 | **S** | 574 | 580 | SQYSLRL | 0.959 |
| 578 | **L** | 575 | 581 | QYSLRLI | 0.944 |
| 579 | **R** | 576 | 582 | YSLRLID | 0.932 |
| 580 | **L** | 577 | 583 | SLRLIDA | 0.931 |
| 581 | **I** | 578 | 584 | LRLIDAM | 0.92 |
| 582 | **D** | 579 | 585 | RLIDAMM | 0.905 |
| 583 | **A** | 580 | 586 | LIDAMMF | 0.891 |
| 584 | **M** | 581 | 587 | IDAMMFT | 0.881 |
| 585 | **M** | 582 | 588 | DAMMFTS | 0.904 |
| 586 | **F** | 583 | 589 | AMMFTSD | 0.95 |
| 587 | **T** | 584 | 590 | MMFTSDL | 1.0 |
| 588 | **S** | 585 | 591 | MFTSDLA | 1.037 |
| 589 | **D** | 586 | 592 | FTSDLAT | 1.044 |
| 590 | **L** | 587 | 593 | TSDLATN | 1.035 |
| 591 | **A** | 588 | 594 | SDLATNN | 1.025 |
| 592 | **T** | 589 | 595 | DLATNNL | 1.024 |
| 593 | **N** | 590 | 596 | LATNNLV | 1.02 |
| 594 | **N** | 591 | 597 | ATNNLVV | 1.003 |
| 595 | **L** | 592 | 598 | TNNLVVM | 0.97 |
| 596 | **V** | 593 | 599 | NNLVVMA | 0.931 |
| 597 | **V** | 594 | 600 | NLVVMAY | 0.897 |
| 598 | **M** | 595 | 601 | LVVMAYI | 0.874 |
| 599 | **A** | 596 | 602 | VVMAYIT | 0.879 |
| 600 | **Y** | 597 | 603 | VMAYITG | 0.904 |
| 601 | **I** | 598 | 604 | MAYITGG | 0.947 |
| 602 | **T** | 599 | 605 | AYITGGV | 0.993 |
| 603 | **G** | 600 | 606 | YITGGVV | 1.016 |
| 604 | **G** | 601 | 607 | ITGGVVQ | 1.007 |
| 605 | **V** | 602 | 608 | TGGVVQL | 0.98 |
| 606 | **V** | 603 | 609 | GGVVQLT | 0.96 |
| 607 | **Q** | 604 | 610 | GVVQLTS | 0.962 |
| 608 | **L** | 605 | 611 | VVQLTSQ | 0.989 |
| 609 | **T** | 606 | 612 | VQLTSQW | 1.018 |
| 610 | **S** | 607 | 613 | QLTSQWL | 1.033 |
| 611 | **Q** | 608 | 614 | LTSQWLT | 1.026 |
| 612 | **W** | 609 | 615 | TSQWLTN | 1.008 |
| 613 | **L** | 610 | 616 | SQWLTNI | 0.992 |
| 614 | **T** | 611 | 617 | QWLTNIF | 0.979 |
| 615 | **N** | 612 | 618 | WLTNIFG | 0.972 |
| 616 | **I** | 613 | 619 | LTNIFGT | 0.97 |
| 617 | **F** | 614 | 620 | TNIFGTV | 0.971 |
| 618 | **G** | 615 | 621 | NIFGTVY | 0.977 |
| 619 | **T** | 616 | 622 | IFGTVYE | 0.983 |
| 620 | **V** | 617 | 623 | FGTVYEK | 0.988 |
| 621 | **Y** | 618 | 624 | GTVYEKL | 0.993 |
| 622 | **E** | 619 | 625 | TVYEKLK | 1.006 |
| 623 | **K** | 620 | 626 | VYEKLKP | 1.023 |
| 624 | **L** | 621 | 627 | YEKLKPV | 1.029 |
| 625 | **K** | 622 | 628 | EKLKPVL | 1.031 |
| 626 | **P** | 623 | 629 | KLKPVLD | 1.015 |
| 627 | **V** | 624 | 630 | LKPVLDW | 0.985 |
| 628 | **L** | 625 | 631 | KPVLDWL | 0.967 |
| 629 | **D** | 626 | 632 | PVLDWLE | 0.954 |
| 630 | **W** | 627 | 633 | VLDWLEE | 0.96 |
| 631 | **L** | 628 | 634 | LDWLEEK | 0.987 |
| 632 | **E** | 629 | 635 | DWLEEKF | 1.011 |
| 633 | **E** | 630 | 636 | WLEEKFK | 1.032 |
| 634 | **K** | 631 | 637 | LEEKFKE | 1.043 |
| 635 | **F** | 632 | 638 | EEKFKEG | 1.042 |
| 636 | **K** | 633 | 639 | EKFKEGV | 1.045 |
| 637 | **E** | 634 | 640 | KFKEGVE | 1.037 |
| 638 | **G** | 635 | 641 | FKEGVEF | 1.017 |
| 639 | **V** | 636 | 642 | KEGVEFL | 0.994 |
| 640 | **E** | 637 | 643 | EGVEFLR | 0.971 |
| 641 | **F** | 638 | 644 | GVEFLRD | 0.964 |
| 642 | **L** | 639 | 645 | VEFLRDG | 0.976 |
| 643 | **R** | 640 | 646 | EFLRDGW | 0.991 |
| 644 | **D** | 641 | 647 | FLRDGWE | 0.997 |
| 645 | **G** | 642 | 648 | LRDGWEI | 0.989 |
| 646 | **W** | 643 | 649 | RDGWEIV | 0.964 |
| 647 | **E** | 644 | 650 | DGWEIVK | 0.951 |
| 648 | **I** | 645 | 651 | GWEIVKF | 0.942 |
| 649 | **V** | 646 | 652 | WEIVKFI | 0.941 |
| 650 | **K** | 647 | 653 | EIVKFIS | 0.956 |
| 651 | **F** | 648 | 654 | IVKFIST | 0.962 |
| 652 | **I** | 649 | 655 | VKFISTC | 0.967 |
| 653 | **S** | 650 | 656 | KFISTCA | 0.972 |
| 654 | **T** | 651 | 657 | FISTCAC | 0.959 |
| 655 | **C** | 652 | 658 | ISTCACE | 0.939 |
| 656 | **A** | 653 | 659 | STCACEI | 0.921 |
| 657 | **C** | 654 | 660 | TCACEIV | 0.907 |
| 658 | **E** | 655 | 661 | CACEIVG | 0.912 |
| 659 | **I** | 656 | 662 | ACEIVGG | 0.939 |
| 660 | **V** | 657 | 663 | CEIVGGQ | 0.973 |
| 661 | **G** | 658 | 664 | EIVGGQI | 1.002 |
| 662 | **G** | 659 | 665 | IVGGQIV | 1.015 |
| 663 | **Q** | 660 | 666 | VGGQIVT | 1.001 |
| 664 | **I** | 661 | 667 | GGQIVTC | 0.969 |
| 665 | **V** | 662 | 668 | GQIVTCA | 0.943 |
| 666 | **T** | 663 | 669 | QIVTCAK | 0.934 |
| 667 | **C** | 664 | 670 | IVTCAKE | 0.944 |
| 668 | **A** | 665 | 671 | VTCAKEI | 0.971 |
| 669 | **K** | 666 | 672 | TCAKEIK | 1.003 |
| 670 | **E** | 667 | 673 | CAKEIKE | 1.026 |
| 671 | **I** | 668 | 674 | AKEIKES | 1.044 |
| 672 | **K** | 669 | 675 | KEIKESV | 1.053 |
| 673 | **E** | 670 | 676 | EIKESVQ | 1.05 |
| 674 | **S** | 671 | 677 | IKESVQT | 1.043 |
| 675 | **V** | 672 | 678 | KESVQTF | 1.028 |
| 676 | **Q** | 673 | 679 | ESVQTFF | 1.009 |
| 677 | **T** | 674 | 680 | SVQTFFK | 0.995 |
| 678 | **F** | 675 | 681 | VQTFFKL | 0.978 |
| 679 | **F** | 676 | 682 | QTFFKLV | 0.971 |
| 680 | **K** | 677 | 683 | TFFKLVN | 0.974 |
| 681 | **L** | 678 | 684 | FFKLVNK | 0.977 |
| 682 | **V** | 679 | 685 | FKLVNKF | 0.982 |
| 683 | **N** | 680 | 686 | KLVNKFL | 0.986 |
| 684 | **K** | 681 | 687 | LVNKFLA | 0.977 |
| 685 | **F** | 682 | 688 | VNKFLAL | 0.956 |
| 686 | **L** | 683 | 689 | NKFLALC | 0.94 |
| 687 | **A** | 684 | 690 | KFLALCA | 0.925 |
| 688 | **L** | 685 | 691 | FLALCAD | 0.929 |
| 689 | **C** | 686 | 692 | LALCADS | 0.952 |
| 690 | **A** | 687 | 693 | ALCADSI | 0.972 |
| 691 | **D** | 688 | 694 | LCADSII | 0.985 |
| 692 | **S** | 689 | 695 | CADSIII | 0.974 |
| 693 | **I** | 690 | 696 | ADSIIIG | 0.955 |
| 694 | **I** | 691 | 697 | DSIIIGG | 0.947 |
| 695 | **I** | 692 | 698 | SIIIGGA | 0.954 |
| 696 | **G** | 693 | 699 | IIIGGAK | 0.984 |
| 697 | **G** | 694 | 700 | IIGGAKL | 1.01 |
| 698 | **A** | 695 | 701 | IGGAKLK | 1.027 |
| 699 | **K** | 696 | 702 | GGAKLKA | 1.027 |
| 700 | **L** | 697 | 703 | GAKLKAL | 1.012 |
| 701 | **K** | 698 | 704 | AKLKALN | 0.998 |
| 702 | **A** | 699 | 705 | KLKALNL | 0.978 |
| 703 | **L** | 700 | 706 | LKALNLG | 0.971 |
| 704 | **N** | 701 | 707 | KALNLGE | 0.977 |
| 705 | **L** | 702 | 708 | ALNLGET | 0.993 |
| 706 | **G** | 703 | 709 | LNLGETF | 1.012 |
| 707 | **E** | 704 | 710 | NLGETFV | 1.017 |
| 708 | **T** | 705 | 711 | LGETFVT | 1.004 |
| 709 | **F** | 706 | 712 | GETFVTH | 0.979 |
| 710 | **V** | 707 | 713 | ETFVTHS | 0.966 |
| 711 | **T** | 708 | 714 | TFVTHSK | 0.97 |
| 712 | **H** | 709 | 715 | FVTHSKG | 0.992 |
| 713 | **S** | 710 | 716 | VTHSKGL | 1.016 |
| 714 | **K** | 711 | 717 | THSKGLY | 1.024 |
| 715 | **G** | 712 | 718 | HSKGLYR | 1.018 |
| 716 | **L** | 713 | 719 | SKGLYRK | 1.008 |
| 717 | **Y** | 714 | 720 | KGLYRKC | 0.994 |
| 718 | **R** | 715 | 721 | GLYRKCV | 0.987 |
| 719 | **K** | 716 | 722 | LYRKCVK | 0.988 |
| 720 | **C** | 717 | 723 | YRKCVKS | 0.991 |
| 721 | **V** | 718 | 724 | RKCVKSR | 1.01 |
| 722 | **K** | 719 | 725 | KCVKSRE | 1.039 |
| 723 | **S** | 720 | 726 | CVKSREE | 1.066 |
| 724 | **R** | 721 | 727 | VKSREET | 1.081 |
| 725 | **E** | 722 | 728 | KSREETG | 1.086 |
| 726 | **E** | 723 | 729 | SREETGL | 1.073 |
| 727 | **T** | 724 | 730 | REETGLL | 1.048 |
| 728 | **G** | 725 | 731 | EETGLLM | 1.016 |
| 729 | **L** | 726 | 732 | ETGLLMP | 0.977 |
| 730 | **L** | 727 | 733 | TGLLMPL | 0.946 |
| 731 | **M** | 728 | 734 | GLLMPLK | 0.935 |
| 732 | **P** | 729 | 735 | LLMPLKA | 0.948 |
| 733 | **L** | 730 | 736 | LMPLKAP | 0.978 |
| 734 | **K** | 731 | 737 | MPLKAPK | 1.015 |
| 735 | **A** | 732 | 738 | PLKAPKE | 1.042 |
| 736 | **P** | 733 | 739 | LKAPKEI | 1.049 |
| 737 | **K** | 734 | 740 | KAPKEII | 1.033 |
| 738 | **E** | 735 | 741 | APKEIIF | 0.998 |
| 739 | **I** | 736 | 742 | PKEIIFL | 0.959 |
| 740 | **I** | 737 | 743 | KEIIFLE | 0.939 |
| 741 | **F** | 738 | 744 | EIIFLEG | 0.949 |
| 742 | **L** | 739 | 745 | IIFLEGE | 0.982 |
| 743 | **E** | 740 | 746 | IFLEGET | 1.024 |
| 744 | **G** | 741 | 747 | FLEGETL | 1.054 |
| 745 | **E** | 742 | 748 | LEGETLP | 1.063 |
| 746 | **T** | 743 | 749 | EGETLPT | 1.059 |
| 747 | **L** | 744 | 750 | GETLPTE | 1.049 |
| 748 | **P** | 745 | 751 | ETLPTEV | 1.041 |
| 749 | **T** | 746 | 752 | TLPTEVL | 1.028 |
| 750 | **E** | 747 | 753 | LPTEVLT | 1.016 |
| 751 | **V** | 748 | 754 | PTEVLTE | 1.008 |
| 752 | **L** | 749 | 755 | TEVLTEE | 1.009 |
| 753 | **T** | 750 | 756 | EVLTEEV | 1.016 |
| 754 | **E** | 751 | 757 | VLTEEVV | 1.016 |
| 755 | **E** | 752 | 758 | LTEEVVL | 1.004 |
| 756 | **V** | 753 | 759 | TEEVVLK | 0.987 |
| 757 | **V** | 754 | 760 | EEVVLKT | 0.982 |
| 758 | **L** | 755 | 761 | EVVLKTG | 1.0 |
| 759 | **K** | 756 | 762 | VVLKTGD | 1.035 |
| 760 | **T** | 757 | 763 | VLKTGDL | 1.059 |
| 761 | **G** | 758 | 764 | LKTGDLQ | 1.071 |
| 762 | **D** | 759 | 765 | KTGDLQP | 1.066 |
| 763 | **L** | 760 | 766 | TGDLQPL | 1.045 |
| 764 | **Q** | 761 | 767 | GDLQPLE | 1.035 |
| 765 | **P** | 762 | 768 | DLQPLEQ | 1.037 |
| 766 | **L** | 763 | 769 | LQPLEQP | 1.04 |
| 767 | **E** | 764 | 770 | QPLEQPT | 1.058 |
| 768 | **Q** | 765 | 771 | PLEQPTS | 1.079 |
| 769 | **P** | 766 | 772 | LEQPTSE | 1.084 |
| 770 | **T** | 767 | 773 | EQPTSEA | 1.084 |
| 771 | **S** | 768 | 774 | QPTSEAV | 1.069 |
| 772 | **E** | 769 | 775 | PTSEAVE | 1.03 |
| 773 | **A** | 770 | 776 | TSEAVEA | 0.999 |
| 774 | **V** | 771 | 777 | SEAVEAP | 0.975 |
| 775 | **E** | 772 | 778 | EAVEAPL | 0.962 |
| 776 | **A** | 773 | 779 | AVEAPLV | 0.963 |
| 777 | **P** | 774 | 780 | VEAPLVG | 0.964 |
| 778 | **L** | 775 | 781 | EAPLVGT | 0.975 |
| 779 | **V** | 776 | 782 | APLVGTP | 0.991 |
| 780 | **G** | 777 | 783 | PLVGTPV | 1.007 |
| 781 | **T** | 778 | 784 | LVGTPVC | 1.017 |
| 782 | **P** | 779 | 785 | VGTPVCI | 1.006 |
| 783 | **V** | 780 | 786 | GTPVCIN | 0.982 |
| 784 | **C** | 781 | 787 | TPVCING | 0.966 |
| 785 | **I** | 782 | 788 | PVCINGL | 0.959 |
| 786 | **N** | 783 | 789 | VCINGLM | 0.958 |
| 787 | **G** | 784 | 790 | CINGLML | 0.957 |
| 788 | **L** | 785 | 791 | INGLMLL | 0.943 |
| 789 | **M** | 786 | 792 | NGLMLLE | 0.925 |
| 790 | **L** | 787 | 793 | GLMLLEI | 0.926 |
| 791 | **L** | 788 | 794 | LMLLEIK | 0.941 |
| 792 | **E** | 789 | 795 | MLLEIKD | 0.966 |
| 793 | **I** | 790 | 796 | LLEIKDT | 1.002 |
| 794 | **K** | 791 | 797 | LEIKDTE | 1.031 |
| 795 | **D** | 792 | 798 | EIKDTEK | 1.05 |
| 796 | **T** | 793 | 799 | IKDTEKY | 1.058 |
| 797 | **E** | 794 | 800 | KDTEKYC | 1.048 |
| 798 | **K** | 795 | 801 | DTEKYCA | 1.02 |
| 799 | **Y** | 796 | 802 | TEKYCAL | 0.982 |
| 800 | **C** | 797 | 803 | EKYCALA | 0.951 |
| 801 | **A** | 798 | 804 | KYCALAP | 0.939 |
| 802 | **L** | 799 | 805 | YCALAPN | 0.947 |
| 803 | **A** | 800 | 806 | CALAPNM | 0.962 |
| 804 | **P** | 801 | 807 | ALAPNMM | 0.966 |
| 805 | **N** | 802 | 808 | LAPNMMV | 0.951 |
| 806 | **M** | 803 | 809 | APNMMVT | 0.931 |
| 807 | **M** | 804 | 810 | PNMMVTN | 0.931 |
| 808 | **V** | 805 | 811 | NMMVTNN | 0.96 |
| 809 | **T** | 806 | 812 | MMVTNNT | 1.006 |
| 810 | **N** | 807 | 813 | MVTNNTF | 1.041 |
| 811 | **N** | 808 | 814 | VTNNTFT | 1.05 |
| 812 | **T** | 809 | 815 | TNNTFTL | 1.029 |
| 813 | **F** | 810 | 816 | NNTFTLK | 1.003 |
| 814 | **T** | 811 | 817 | NTFTLKG | 0.997 |
| 815 | **L** | 812 | 818 | TFTLKGG | 1.01 |
| 816 | **K** | 813 | 819 | FTLKGGA | 1.036 |
| 817 | **G** | 814 | 820 | TLKGGAP | 1.061 |
| 818 | **G** | 815 | 821 | LKGGAPT | 1.068 |
| 819 | **A** | 816 | 822 | KGGAPTK | 1.071 |
| 820 | **P** | 817 | 823 | GGAPTKV | 1.066 |
| 821 | **T** | 818 | 824 | GAPTKVT | 1.051 |
| 822 | **K** | 819 | 825 | APTKVTF | 1.031 |
| 823 | **V** | 820 | 826 | PTKVTFG | 1.007 |
| 824 | **T** | 821 | 827 | TKVTFGD | 0.989 |
| 825 | **F** | 822 | 828 | KVTFGDD | 0.987 |
| 826 | **G** | 823 | 829 | VTFGDDT | 1.001 |
| 827 | **D** | 824 | 830 | TFGDDTV | 1.011 |
| 828 | **D** | 825 | 831 | FGDDTVI | 1.009 |
| 829 | **T** | 826 | 832 | GDDTVIE | 0.994 |
| 830 | **V** | 827 | 833 | DDTVIEV | 0.968 |
| 831 | **I** | 828 | 834 | DTVIEVQ | 0.955 |
| 832 | **E** | 829 | 835 | TVIEVQG | 0.96 |
| 833 | **V** | 830 | 836 | VIEVQGY | 0.973 |
| 834 | **Q** | 831 | 837 | IEVQGYK | 0.997 |
| 835 | **G** | 832 | 838 | EVQGYKS | 1.015 |
| 836 | **Y** | 833 | 839 | VQGYKSV | 1.021 |
| 837 | **K** | 834 | 840 | QGYKSVN | 1.023 |
| 838 | **S** | 835 | 841 | GYKSVNI | 1.013 |
| 839 | **V** | 836 | 842 | YKSVNIT | 0.995 |
| 840 | **N** | 837 | 843 | KSVNITF | 0.978 |
| 841 | **I** | 838 | 844 | SVNITFE | 0.963 |
| 842 | **T** | 839 | 845 | VNITFEL | 0.95 |
| 843 | **F** | 840 | 846 | NITFELD | 0.955 |
| 844 | **E** | 841 | 847 | ITFELDE | 0.973 |
| 845 | **L** | 842 | 848 | TFELDER | 0.997 |
| 846 | **D** | 843 | 849 | FELDERI | 1.025 |
| 847 | **E** | 844 | 850 | ELDERID | 1.043 |
| 848 | **R** | 845 | 851 | LDERIDK | 1.05 |
| 849 | **I** | 846 | 852 | DERIDKV | 1.045 |
| 850 | **D** | 847 | 853 | ERIDKVL | 1.036 |
| 851 | **K** | 848 | 854 | RIDKVLN | 1.021 |
| 852 | **V** | 849 | 855 | IDKVLNE | 1.008 |
| 853 | **L** | 850 | 856 | DKVLNEK | 1.01 |
| 854 | **N** | 851 | 857 | KVLNEKC | 1.016 |
| 855 | **E** | 852 | 858 | VLNEKCS | 1.021 |
| 856 | **K** | 853 | 859 | LNEKCSA | 1.016 |
| 857 | **C** | 854 | 860 | NEKCSAY | 0.992 |
| 858 | **S** | 855 | 861 | EKCSAYT | 0.966 |
| 859 | **A** | 856 | 862 | KCSAYTV | 0.95 |
| 860 | **Y** | 857 | 863 | CSAYTVE | 0.941 |
| 861 | **T** | 858 | 864 | SAYTVEL | 0.945 |
| 862 | **V** | 859 | 865 | AYTVELG | 0.957 |
| 863 | **E** | 860 | 866 | YTVELGT | 0.971 |
| 864 | **L** | 861 | 867 | TVELGTE | 0.992 |
| 865 | **G** | 862 | 868 | VELGTEV | 1.012 |
| 866 | **T** | 863 | 869 | ELGTEVN | 1.023 |
| 867 | **E** | 864 | 870 | LGTEVNE | 1.026 |
| 868 | **V** | 865 | 871 | GTEVNEF | 1.014 |
| 869 | **N** | 866 | 872 | TEVNEFA | 0.996 |
| 870 | **E** | 867 | 873 | EVNEFAC | 0.975 |
| 871 | **F** | 868 | 874 | VNEFACV | 0.949 |
| 872 | **A** | 869 | 875 | NEFACVV | 0.931 |
| 873 | **C** | 870 | 876 | EFACVVA | 0.922 |
| 874 | **V** | 871 | 877 | FACVVAD | 0.918 |
| 875 | **V** | 872 | 878 | ACVVADA | 0.924 |
| 876 | **A** | 873 | 879 | CVVADAV | 0.93 |
| 877 | **D** | 874 | 880 | VVADAVI | 0.929 |
| 878 | **A** | 875 | 881 | VADAVIK | 0.937 |
| 879 | **V** | 876 | 882 | ADAVIKT | 0.949 |
| 880 | **I** | 877 | 883 | DAVIKTL | 0.965 |
| 881 | **K** | 878 | 884 | AVIKTLQ | 0.993 |
| 882 | **T** | 879 | 885 | VIKTLQP | 1.012 |
| 883 | **L** | 880 | 886 | IKTLQPV | 1.02 |
| 884 | **Q** | 881 | 887 | KTLQPVS | 1.029 |
| 885 | **P** | 882 | 888 | TLQPVSE | 1.03 |
| 886 | **V** | 883 | 889 | LQPVSEL | 1.024 |
| 887 | **S** | 884 | 890 | QPVSELL | 1.02 |
| 888 | **E** | 885 | 891 | PVSELLT | 1.012 |
| 889 | **L** | 886 | 892 | VSELLTP | 1.006 |
| 890 | **L** | 887 | 893 | SELLTPL | 1.009 |
| 891 | **T** | 888 | 894 | ELLTPLG | 1.01 |
| 892 | **P** | 889 | 895 | LLTPLGI | 1.008 |
| 893 | **L** | 890 | 896 | LTPLGID | 0.993 |
| 894 | **G** | 891 | 897 | TPLGIDL | 0.978 |
| 895 | **I** | 892 | 898 | PLGIDLD | 0.976 |
| 896 | **D** | 893 | 899 | LGIDLDE | 0.979 |
| 897 | **L** | 894 | 900 | GIDLDEW | 0.991 |
| 898 | **D** | 895 | 901 | IDLDEWS | 1.0 |
| 899 | **E** | 896 | 902 | DLDEWSM | 0.985 |
| 900 | **W** | 897 | 903 | LDEWSMA | 0.962 |
| 901 | **S** | 898 | 904 | DEWSMAT | 0.94 |
| 902 | **M** | 899 | 905 | EWSMATY | 0.921 |
| 903 | **A** | 900 | 906 | WSMATYY | 0.915 |
| 904 | **T** | 901 | 907 | SMATYYL | 0.913 |
| 905 | **Y** | 902 | 908 | MATYYLF | 0.909 |
| 906 | **Y** | 903 | 909 | ATYYLFD | 0.914 |
| 907 | **L** | 904 | 910 | TYYLFDE | 0.937 |
| 908 | **F** | 905 | 911 | YYLFDES | 0.977 |
| 909 | **D** | 906 | 912 | YLFDESG | 1.033 |
| 910 | **E** | 907 | 913 | LFDESGE | 1.076 |
| 911 | **S** | 908 | 914 | FDESGEF | 1.092 |
| 912 | **G** | 909 | 915 | DESGEFK | 1.086 |
| 913 | **E** | 910 | 916 | ESGEFKL | 1.054 |
| 914 | **F** | 911 | 917 | SGEFKLA | 1.02 |
| 915 | **K** | 912 | 918 | GEFKLAS | 0.996 |
| 916 | **L** | 913 | 919 | EFKLASH | 0.972 |
| 917 | **A** | 914 | 920 | FKLASHM | 0.949 |
| 918 | **S** | 915 | 921 | KLASHMY | 0.926 |
| 919 | **H** | 916 | 922 | LASHMYC | 0.9 |
| 920 | **M** | 917 | 923 | ASHMYCS | 0.878 |
| 921 | **Y** | 918 | 924 | SHMYCSF | 0.874 |
| 922 | **C** | 919 | 925 | HMYCSFY | 0.882 |
| 923 | **S** | 920 | 926 | MYCSFYP | 0.905 |
| 924 | **F** | 921 | 927 | YCSFYPP | 0.939 |
| 925 | **Y** | 922 | 928 | CSFYPPD | 0.974 |
| 926 | **P** | 923 | 929 | SFYPPDE | 1.013 |
| 927 | **P** | 924 | 930 | FYPPDED | 1.035 |
| 928 | **D** | 925 | 931 | YPPDEDE | 1.052 |
| 929 | **E** | 926 | 932 | PPDEDEE | 1.066 |
| 930 | **D** | 927 | 933 | PDEDEEE | 1.069 |
| 931 | **E** | 928 | 934 | DEDEEEG | 1.082 |
| 932 | **E** | 929 | 935 | EDEEEGD | 1.092 |
| 933 | **E** | 930 | 936 | DEEEGDC | 1.09 |
| 934 | **G** | 931 | 937 | EEEGDCE | 1.085 |
| 935 | **D** | 932 | 938 | EEGDCEE | 1.069 |
| 936 | **C** | 933 | 939 | EGDCEEE | 1.055 |
| 937 | **E** | 934 | 940 | GDCEEEE | 1.053 |
| 938 | **E** | 935 | 941 | DCEEEEF | 1.049 |
| 939 | **E** | 936 | 942 | CEEEEFE | 1.043 |
| 940 | **E** | 937 | 943 | EEEEFEP | 1.035 |
| 941 | **F** | 938 | 944 | EEEFEPS | 1.031 |
| 942 | **E** | 939 | 945 | EEFEPST | 1.042 |
| 943 | **P** | 940 | 946 | EFEPSTQ | 1.057 |
| 944 | **S** | 941 | 947 | FEPSTQY | 1.067 |
| 945 | **T** | 942 | 948 | EPSTQYE | 1.055 |
| 946 | **Q** | 943 | 949 | PSTQYEY | 1.027 |
| 947 | **Y** | 944 | 950 | STQYEYG | 1.0 |
| 948 | **E** | 945 | 951 | TQYEYGT | 0.986 |
| 949 | **Y** | 946 | 952 | QYEYGTE | 0.997 |
| 950 | **G** | 947 | 953 | YEYGTED | 1.02 |
| 951 | **T** | 948 | 954 | EYGTEDD | 1.044 |
| 952 | **E** | 949 | 955 | YGTEDDY | 1.055 |
| 953 | **D** | 950 | 956 | GTEDDYQ | 1.051 |
| 954 | **D** | 951 | 957 | TEDDYQG | 1.05 |
| 955 | **Y** | 952 | 958 | EDDYQGK | 1.046 |
| 956 | **Q** | 953 | 959 | DDYQGKP | 1.056 |
| 957 | **G** | 954 | 960 | DYQGKPL | 1.067 |
| 958 | **K** | 955 | 961 | YQGKPLE | 1.059 |
| 959 | **P** | 956 | 962 | QGKPLEF | 1.039 |
| 960 | **L** | 957 | 963 | GKPLEFG | 1.002 |
| 961 | **E** | 958 | 964 | KPLEFGA | 0.973 |
| 962 | **F** | 959 | 965 | PLEFGAT | 0.965 |
| 963 | **G** | 960 | 966 | LEFGATS | 0.974 |
| 964 | **A** | 961 | 967 | EFGATSA | 0.995 |
| 965 | **T** | 962 | 968 | FGATSAA | 1.002 |
| 966 | **S** | 963 | 969 | GATSAAL | 0.996 |
| 967 | **A** | 964 | 970 | ATSAALQ | 0.981 |
| 968 | **A** | 965 | 971 | TSAALQP | 0.972 |
| 969 | **L** | 966 | 972 | SAALQPE | 0.984 |
| 970 | **Q** | 967 | 973 | AALQPEE | 1.011 |
| 971 | **P** | 968 | 974 | ALQPEEE | 1.043 |
| 972 | **E** | 969 | 975 | LQPEEEQ | 1.075 |
| 973 | **E** | 970 | 976 | QPEEEQE | 1.094 |
| 974 | **E** | 971 | 977 | PEEEQEE | 1.105 |
| 975 | **Q** | 972 | 978 | EEEQEED | 1.111 |
| 976 | **E** | 973 | 979 | EEQEEDW | 1.096 |
| 977 | **E** | 974 | 980 | EQEEDWL | 1.071 |
| 978 | **D** | 975 | 981 | QEEDWLD | 1.047 |
| 979 | **W** | 976 | 982 | EEDWLDD | 1.019 |
| 980 | **L** | 977 | 983 | EDWLDDD | 1.015 |
| 981 | **D** | 978 | 984 | DWLDDDS | 1.031 |
| 982 | **D** | 979 | 985 | WLDDDSQ | 1.052 |
| 983 | **D** | 980 | 986 | LDDDSQQ | 1.086 |
| 984 | **S** | 981 | 987 | DDDSQQT | 1.113 |
| 985 | **Q** | 982 | 988 | DDSQQTV | 1.115 |
| 986 | **Q** | 983 | 989 | DSQQTVG | 1.105 |
| 987 | **T** | 984 | 990 | SQQTVGQ | 1.087 |
| 988 | **V** | 985 | 991 | QQTVGQQ | 1.075 |
| 989 | **G** | 986 | 992 | QTVGQQD | 1.077 |
| 990 | **Q** | 987 | 993 | TVGQQDG | 1.094 |
| 991 | **Q** | 988 | 994 | VGQQDGS | 1.111 |
| 992 | **D** | 989 | 995 | GQQDGSE | 1.116 |
| 993 | **G** | 990 | 996 | QQDGSED | 1.118 |
| 994 | **S** | 991 | 997 | QDGSEDN | 1.112 |
| 995 | **E** | 992 | 998 | DGSEDNQ | 1.106 |
| 996 | **D** | 993 | 999 | GSEDNQT | 1.103 |
| 997 | **N** | 994 | 1000 | SEDNQTT | 1.102 |
| 998 | **Q** | 995 | 1001 | EDNQTTT | 1.099 |
| 999 | **T** | 996 | 1002 | DNQTTTI | 1.086 |
| 1000 | **T** | 997 | 1003 | NQTTTIQ | 1.071 |
| 1001 | **T** | 998 | 1004 | QTTTIQT | 1.053 |
| 1002 | **I** | 999 | 1005 | TTTIQTI | 1.029 |
| 1003 | **Q** | 1000 | 1006 | TTIQTIV | 1.01 |
| 1004 | **T** | 1001 | 1007 | TIQTIVE | 0.988 |
| 1005 | **I** | 1002 | 1008 | IQTIVEV | 0.963 |
| 1006 | **V** | 1003 | 1009 | QTIVEVQ | 0.957 |
| 1007 | **E** | 1004 | 1010 | TIVEVQP | 0.963 |
| 1008 | **V** | 1005 | 1011 | IVEVQPQ | 0.981 |
| 1009 | **Q** | 1006 | 1012 | VEVQPQL | 1.002 |
| 1010 | **P** | 1007 | 1013 | EVQPQLE | 1.01 |
| 1011 | **Q** | 1008 | 1014 | VQPQLEM | 1.002 |
| 1012 | **L** | 1009 | 1015 | QPQLEME | 0.982 |
| 1013 | **E** | 1010 | 1016 | PQLEMEL | 0.964 |
| 1014 | **M** | 1011 | 1017 | QLEMELT | 0.958 |
| 1015 | **E** | 1012 | 1018 | LEMELTP | 0.968 |
| 1016 | **L** | 1013 | 1019 | EMELTPV | 0.984 |
| 1017 | **T** | 1014 | 1020 | MELTPVV | 0.997 |
| 1018 | **P** | 1015 | 1021 | ELTPVVQ | 1.001 |
| 1019 | **V** | 1016 | 1022 | LTPVVQT | 0.995 |
| 1020 | **V** | 1017 | 1023 | TPVVQTI | 0.994 |
| 1021 | **Q** | 1018 | 1024 | PVVQTIE | 0.995 |
| 1022 | **T** | 1019 | 1025 | VVQTIEV | 0.995 |
| 1023 | **I** | 1020 | 1026 | VQTIEVN | 0.995 |
| 1024 | **E** | 1021 | 1027 | QTIEVNS | 0.992 |
| 1025 | **V** | 1022 | 1028 | TIEVNSF | 0.989 |
| 1026 | **N** | 1023 | 1029 | IEVNSFS | 0.993 |
| 1027 | **S** | 1024 | 1030 | EVNSFSG | 1.002 |
| 1028 | **F** | 1025 | 1031 | VNSFSGY | 1.001 |
| 1029 | **S** | 1026 | 1032 | NSFSGYL | 1.002 |
| 1030 | **G** | 1027 | 1033 | SFSGYLK | 0.999 |
| 1031 | **Y** | 1028 | 1034 | FSGYLKL | 0.99 |
| 1032 | **L** | 1029 | 1035 | SGYLKLT | 0.995 |
| 1033 | **K** | 1030 | 1036 | GYLKLTD | 1.003 |
| 1034 | **L** | 1031 | 1037 | YLKLTDN | 1.007 |
| 1035 | **T** | 1032 | 1038 | LKLTDNV | 1.014 |
| 1036 | **D** | 1033 | 1039 | KLTDNVY | 0.999 |
| 1037 | **N** | 1034 | 1040 | LTDNVYI | 0.971 |
| 1038 | **V** | 1035 | 1041 | TDNVYIK | 0.951 |
| 1039 | **Y** | 1036 | 1042 | DNVYIKN | 0.939 |
| 1040 | **I** | 1037 | 1043 | NVYIKNA | 0.952 |
| 1041 | **K** | 1038 | 1044 | VYIKNAD | 0.977 |
| 1042 | **N** | 1039 | 1045 | YIKNADI | 0.986 |
| 1043 | **A** | 1040 | 1046 | IKNADIV | 0.984 |
| 1044 | **D** | 1041 | 1047 | KNADIVE | 0.969 |
| 1045 | **I** | 1042 | 1048 | NADIVEE | 0.959 |
| 1046 | **V** | 1043 | 1049 | ADIVEEA | 0.969 |
| 1047 | **E** | 1044 | 1050 | DIVEEAK | 0.995 |
| 1048 | **E** | 1045 | 1051 | IVEEAKK | 1.023 |
| 1049 | **A** | 1046 | 1052 | VEEAKKV | 1.041 |
| 1050 | **K** | 1047 | 1053 | EEAKKVK | 1.053 |
| 1051 | **K** | 1048 | 1054 | EAKKVKP | 1.054 |
| 1052 | **V** | 1049 | 1055 | AKKVKPT | 1.049 |
| 1053 | **K** | 1050 | 1056 | KKVKPTV | 1.045 |
| 1054 | **P** | 1051 | 1057 | KVKPTVV | 1.027 |
| 1055 | **T** | 1052 | 1058 | VKPTVVV | 1.003 |
| 1056 | **V** | 1053 | 1059 | KPTVVVN | 0.974 |
| 1057 | **V** | 1054 | 1060 | PTVVVNA | 0.949 |
| 1058 | **V** | 1055 | 1061 | TVVVNAA | 0.936 |
| 1059 | **N** | 1056 | 1062 | VVVNAAN | 0.932 |
| 1060 | **A** | 1057 | 1063 | VVNAANV | 0.935 |
| 1061 | **A** | 1058 | 1064 | VNAANVY | 0.931 |
| 1062 | **N** | 1059 | 1065 | NAANVYL | 0.925 |
| 1063 | **V** | 1060 | 1066 | AANVYLK | 0.927 |
| 1064 | **Y** | 1061 | 1067 | ANVYLKH | 0.932 |
| 1065 | **L** | 1062 | 1068 | NVYLKHG | 0.957 |
| 1066 | **K** | 1063 | 1069 | VYLKHGG | 0.993 |
| 1067 | **H** | 1064 | 1070 | YLKHGGG | 1.019 |
| 1068 | **G** | 1065 | 1071 | LKHGGGV | 1.039 |
| 1069 | **G** | 1066 | 1072 | KHGGGVA | 1.04 |
| 1070 | **G** | 1067 | 1073 | HGGGVAG | 1.016 |
| 1071 | **V** | 1068 | 1074 | GGGVAGA | 0.987 |
| 1072 | **A** | 1069 | 1075 | GGVAGAL | 0.963 |
| 1073 | **G** | 1070 | 1076 | GVAGALN | 0.95 |
| 1074 | **A** | 1071 | 1077 | VAGALNK | 0.959 |
| 1075 | **L** | 1072 | 1078 | AGALNKA | 0.981 |
| 1076 | **N** | 1073 | 1079 | GALNKAT | 1.006 |
| 1077 | **K** | 1074 | 1080 | ALNKATN | 1.035 |
| 1078 | **A** | 1075 | 1081 | LNKATNN | 1.048 |
| 1079 | **T** | 1076 | 1082 | NKATNNA | 1.05 |
| 1080 | **N** | 1077 | 1083 | KATNNAM | 1.035 |
| 1081 | **N** | 1078 | 1084 | ATNNAMQ | 0.998 |
| 1082 | **A** | 1079 | 1085 | TNNAMQV | 0.964 |
| 1083 | **M** | 1080 | 1086 | NNAMQVE | 0.942 |
| 1084 | **Q** | 1081 | 1087 | NAMQVES | 0.951 |
| 1085 | **V** | 1082 | 1088 | AMQVESD | 0.983 |
| 1086 | **E** | 1083 | 1089 | MQVESDD | 1.024 |
| 1087 | **S** | 1084 | 1090 | QVESDDY | 1.052 |
| 1088 | **D** | 1085 | 1091 | VESDDYI | 1.043 |
| 1089 | **D** | 1086 | 1092 | ESDDYIA | 1.019 |
| 1090 | **Y** | 1087 | 1093 | SDDYIAT | 0.986 |
| 1091 | **I** | 1088 | 1094 | DDYIATN | 0.972 |
| 1092 | **A** | 1089 | 1095 | DYIATNG | 0.992 |
| 1093 | **T** | 1090 | 1096 | YIATNGP | 1.027 |
| 1094 | **N** | 1091 | 1097 | IATNGPL | 1.059 |
| 1095 | **G** | 1092 | 1098 | ATNGPLK | 1.076 |
| 1096 | **P** | 1093 | 1099 | TNGPLKV | 1.065 |
| 1097 | **L** | 1094 | 1100 | NGPLKVG | 1.044 |
| 1098 | **K** | 1095 | 1101 | GPLKVGG | 1.038 |
| 1099 | **V** | 1096 | 1102 | PLKVGGS | 1.036 |
| 1100 | **G** | 1097 | 1103 | LKVGGSC | 1.037 |
| 1101 | **G** | 1098 | 1104 | KVGGSCV | 1.033 |
| 1102 | **S** | 1099 | 1105 | VGGSCVL | 1.007 |
| 1103 | **C** | 1100 | 1106 | GGSCVLS | 0.981 |
| 1104 | **V** | 1101 | 1107 | GSCVLSG | 0.972 |
| 1105 | **L** | 1102 | 1108 | SCVLSGH | 0.975 |
| 1106 | **S** | 1103 | 1109 | CVLSGHN | 0.987 |
| 1107 | **G** | 1104 | 1110 | VLSGHNL | 0.995 |
| 1108 | **H** | 1105 | 1111 | LSGHNLA | 0.986 |
| 1109 | **N** | 1106 | 1112 | SGHNLAK | 0.977 |
| 1110 | **L** | 1107 | 1113 | GHNLAKH | 0.972 |
| 1111 | **A** | 1108 | 1114 | HNLAKHC | 0.969 |
| 1112 | **K** | 1109 | 1115 | NLAKHCL | 0.972 |
| 1113 | **H** | 1110 | 1116 | LAKHCLH | 0.959 |
| 1114 | **C** | 1111 | 1117 | AKHCLHV | 0.943 |
| 1115 | **L** | 1112 | 1118 | KHCLHVV | 0.928 |
| 1116 | **H** | 1113 | 1119 | HCLHVVG | 0.923 |
| 1117 | **V** | 1114 | 1120 | CLHVVGP | 0.939 |
| 1118 | **V** | 1115 | 1121 | LHVVGPN | 0.962 |
| 1119 | **G** | 1116 | 1122 | HVVGPNV | 0.989 |
| 1120 | **P** | 1117 | 1123 | VVGPNVN | 1.006 |
| 1121 | **N** | 1118 | 1124 | VGPNVNK | 1.016 |
| 1122 | **V** | 1119 | 1125 | GPNVNKG | 1.028 |
| 1123 | **N** | 1120 | 1126 | PNVNKGE | 1.043 |
| 1124 | **K** | 1121 | 1127 | NVNKGED | 1.066 |
| 1125 | **G** | 1122 | 1128 | VNKGEDI | 1.081 |
| 1126 | **E** | 1123 | 1129 | NKGEDIQ | 1.072 |
| 1127 | **D** | 1124 | 1130 | KGEDIQL | 1.047 |
| 1128 | **I** | 1125 | 1131 | GEDIQLL | 1.009 |
| 1129 | **Q** | 1126 | 1132 | EDIQLLK | 0.982 |
| 1130 | **L** | 1127 | 1133 | DIQLLKS | 0.98 |
| 1131 | **L** | 1128 | 1134 | IQLLKSA | 0.987 |
| 1132 | **K** | 1129 | 1135 | QLLKSAY | 0.999 |
| 1133 | **S** | 1130 | 1136 | LLKSAYE | 1.003 |
| 1134 | **A** | 1131 | 1137 | LKSAYEN | 0.995 |
| 1135 | **Y** | 1132 | 1138 | KSAYENF | 0.987 |
| 1136 | **E** | 1133 | 1139 | SAYENFN | 0.985 |
| 1137 | **N** | 1134 | 1140 | AYENFNQ | 0.985 |
| 1138 | **F** | 1135 | 1141 | YENFNQH | 0.987 |
| 1139 | **N** | 1136 | 1142 | ENFNQHE | 0.99 |
| 1140 | **Q** | 1137 | 1143 | NFNQHEV | 0.983 |
| 1141 | **H** | 1138 | 1144 | FNQHEVL | 0.97 |
| 1142 | **E** | 1139 | 1145 | NQHEVLL | 0.955 |
| 1143 | **V** | 1140 | 1146 | QHEVLLA | 0.941 |
| 1144 | **L** | 1141 | 1147 | HEVLLAP | 0.931 |
| 1145 | **L** | 1142 | 1148 | EVLLAPL | 0.931 |
| 1146 | **A** | 1143 | 1149 | VLLAPLL | 0.937 |
| 1147 | **P** | 1144 | 1150 | LLAPLLS | 0.941 |
| 1148 | **L** | 1145 | 1151 | LAPLLSA | 0.951 |
| 1149 | **L** | 1146 | 1152 | APLLSAG | 0.957 |
| 1150 | **S** | 1147 | 1153 | PLLSAGI | 0.956 |
| 1151 | **A** | 1148 | 1154 | LLSAGIF | 0.955 |
| 1152 | **G** | 1149 | 1155 | LSAGIFG | 0.94 |
| 1153 | **I** | 1150 | 1156 | SAGIFGA | 0.935 |
| 1154 | **F** | 1151 | 1157 | AGIFGAD | 0.947 |
| 1155 | **G** | 1152 | 1158 | GIFGADP | 0.97 |
| 1156 | **A** | 1153 | 1159 | IFGADPI | 0.999 |
| 1157 | **D** | 1154 | 1160 | FGADPIH | 1.014 |
| 1158 | **P** | 1155 | 1161 | GADPIHS | 1.007 |
| 1159 | **I** | 1156 | 1162 | ADPIHSL | 0.982 |
| 1160 | **H** | 1157 | 1163 | DPIHSLR | 0.959 |
| 1161 | **S** | 1158 | 1164 | PIHSLRV | 0.94 |
| 1162 | **L** | 1159 | 1165 | IHSLRVC | 0.932 |
| 1163 | **R** | 1160 | 1166 | HSLRVCV | 0.929 |
| 1164 | **V** | 1161 | 1167 | SLRVCVD | 0.937 |
| 1165 | **C** | 1162 | 1168 | LRVCVDT | 0.954 |
| 1166 | **V** | 1163 | 1169 | RVCVDTV | 0.974 |
| 1167 | **D** | 1164 | 1170 | VCVDTVR | 1.004 |
| 1168 | **T** | 1165 | 1171 | CVDTVRT | 1.02 |
| 1169 | **V** | 1166 | 1172 | VDTVRTN | 1.026 |
| 1170 | **R** | 1167 | 1173 | DTVRTNV | 1.025 |
| 1171 | **T** | 1168 | 1174 | TVRTNVY | 1.006 |
| 1172 | **N** | 1169 | 1175 | VRTNVYL | 0.979 |
| 1173 | **V** | 1170 | 1176 | RTNVYLA | 0.947 |
| 1174 | **Y** | 1171 | 1177 | TNVYLAV | 0.917 |
| 1175 | **L** | 1172 | 1178 | NVYLAVF | 0.904 |
| 1176 | **A** | 1173 | 1179 | VYLAVFD | 0.911 |
| 1177 | **V** | 1174 | 1180 | YLAVFDK | 0.938 |
| 1178 | **F** | 1175 | 1181 | LAVFDKN | 0.972 |
| 1179 | **D** | 1176 | 1182 | AVFDKNL | 1.004 |
| 1180 | **K** | 1177 | 1183 | VFDKNLY | 1.015 |
| 1181 | **N** | 1178 | 1184 | FDKNLYD | 1.014 |
| 1182 | **L** | 1179 | 1185 | DKNLYDK | 1.012 |
| 1183 | **Y** | 1180 | 1186 | KNLYDKL | 1.006 |
| 1184 | **D** | 1181 | 1187 | NLYDKLV | 1.011 |
| 1185 | **K** | 1182 | 1188 | LYDKLVS | 1.013 |
| 1186 | **L** | 1183 | 1189 | YDKLVSS | 1.008 |
| 1187 | **V** | 1184 | 1190 | DKLVSSF | 1.0 |
| 1188 | **S** | 1185 | 1191 | KLVSSFL | 0.994 |
| 1189 | **S** | 1186 | 1192 | LVSSFLE | 0.984 |
| 1190 | **F** | 1187 | 1193 | VSSFLEM | 0.969 |
| 1191 | **L** | 1188 | 1194 | SSFLEMK | 0.969 |
| 1192 | **E** | 1189 | 1195 | SFLEMKS | 0.979 |
| 1193 | **M** | 1190 | 1196 | FLEMKSE | 1.006 |
| 1194 | **K** | 1191 | 1197 | LEMKSEK | 1.049 |
| 1195 | **S** | 1192 | 1198 | EMKSEKQ | 1.078 |
| 1196 | **E** | 1193 | 1199 | MKSEKQV | 1.082 |
| 1197 | **K** | 1194 | 1200 | KSEKQVE | 1.072 |
| 1198 | **Q** | 1195 | 1201 | SEKQVEQ | 1.058 |
| 1199 | **V** | 1196 | 1202 | EKQVEQK | 1.051 |
| 1200 | **E** | 1197 | 1203 | KQVEQKI | 1.049 |
| 1201 | **Q** | 1198 | 1204 | QVEQKIA | 1.046 |
| 1202 | **K** | 1199 | 1205 | VEQKIAE | 1.024 |
| 1203 | **I** | 1200 | 1206 | EQKIAEI | 0.993 |
| 1204 | **A** | 1201 | 1207 | QKIAEIP | 0.98 |
| 1205 | **E** | 1202 | 1208 | KIAEIPK | 0.982 |
| 1206 | **I** | 1203 | 1209 | IAEIPKE | 1.008 |
| 1207 | **P** | 1204 | 1210 | AEIPKEE | 1.041 |
| 1208 | **K** | 1205 | 1211 | EIPKEEV | 1.056 |
| 1209 | **E** | 1206 | 1212 | IPKEEVK | 1.061 |
| 1210 | **E** | 1207 | 1213 | PKEEVKP | 1.056 |
| 1211 | **V** | 1208 | 1214 | KEEVKPF | 1.04 |
| 1212 | **K** | 1209 | 1215 | EEVKPFI | 1.026 |
| 1213 | **P** | 1210 | 1216 | EVKPFIT | 1.01 |
| 1214 | **F** | 1211 | 1217 | VKPFITE | 0.995 |
| 1215 | **I** | 1212 | 1218 | KPFITES | 1.004 |
| 1216 | **T** | 1213 | 1219 | PFITESK | 1.031 |
| 1217 | **E** | 1214 | 1220 | FITESKP | 1.061 |
| 1218 | **S** | 1215 | 1221 | ITESKPS | 1.087 |
| 1219 | **K** | 1216 | 1222 | TESKPSV | 1.085 |
| 1220 | **P** | 1217 | 1223 | ESKPSVE | 1.067 |
| 1221 | **S** | 1218 | 1224 | SKPSVEQ | 1.056 |
| 1222 | **V** | 1219 | 1225 | KPSVEQR | 1.047 |
| 1223 | **E** | 1220 | 1226 | PSVEQRK | 1.057 |
| 1224 | **Q** | 1221 | 1227 | SVEQRKQ | 1.078 |
| 1225 | **R** | 1222 | 1228 | VEQRKQD | 1.084 |
| 1226 | **K** | 1223 | 1229 | EQRKQDD | 1.09 |
| 1227 | **Q** | 1224 | 1230 | QRKQDDK | 1.09 |
| 1228 | **D** | 1225 | 1231 | RKQDDKK | 1.076 |
| 1229 | **D** | 1226 | 1232 | KQDDKKI | 1.069 |
| 1230 | **K** | 1227 | 1233 | QDDKKIK | 1.063 |
| 1231 | **K** | 1228 | 1234 | DDKKIKA | 1.048 |
| 1232 | **I** | 1229 | 1235 | DKKIKAC | 1.029 |
| 1233 | **K** | 1230 | 1236 | KKIKACV | 1.007 |
| 1234 | **A** | 1231 | 1237 | KIKACVE | 0.982 |
| 1235 | **C** | 1232 | 1238 | IKACVEE | 0.971 |
| 1236 | **V** | 1233 | 1239 | KACVEEV | 0.975 |
| 1237 | **E** | 1234 | 1240 | ACVEEVT | 0.99 |
| 1238 | **E** | 1235 | 1241 | CVEEVTT | 1.01 |
| 1239 | **V** | 1236 | 1242 | VEEVTTT | 1.024 |
| 1240 | **T** | 1237 | 1243 | EEVTTTL | 1.034 |
| 1241 | **T** | 1238 | 1244 | EVTTTLE | 1.035 |
| 1242 | **T** | 1239 | 1245 | VTTTLEE | 1.036 |
| 1243 | **L** | 1240 | 1246 | TTTLEET | 1.037 |
| 1244 | **E** | 1241 | 1247 | TTLEETK | 1.046 |
| 1245 | **E** | 1242 | 1248 | TLEETKF | 1.048 |
| 1246 | **T** | 1243 | 1249 | LEETKFL | 1.04 |
| 1247 | **K** | 1244 | 1250 | EETKFLT | 1.03 |
| 1248 | **F** | 1245 | 1251 | ETKFLTE | 1.013 |
| 1249 | **L** | 1246 | 1252 | TKFLTEN | 1.01 |
| 1250 | **T** | 1247 | 1253 | KFLTENL | 1.015 |
| 1251 | **E** | 1248 | 1254 | FLTENLL | 1.014 |
| 1252 | **N** | 1249 | 1255 | LTENLLL | 1.001 |
| 1253 | **L** | 1250 | 1256 | TENLLLY | 0.971 |
| 1254 | **L** | 1251 | 1257 | ENLLLYI | 0.938 |
| 1255 | **L** | 1252 | 1258 | NLLLYID | 0.913 |
| 1256 | **Y** | 1253 | 1259 | LLLYIDI | 0.904 |
| 1257 | **I** | 1254 | 1260 | LLYIDIN | 0.916 |
| 1258 | **D** | 1255 | 1261 | LYIDING | 0.947 |
| 1259 | **I** | 1256 | 1262 | YIDINGN | 0.983 |
| 1260 | **N** | 1257 | 1263 | IDINGNL | 1.012 |
| 1261 | **G** | 1258 | 1264 | DINGNLH | 1.026 |
| 1262 | **N** | 1259 | 1265 | INGNLHP | 1.021 |
| 1263 | **L** | 1260 | 1266 | NGNLHPD | 1.013 |
| 1264 | **H** | 1261 | 1267 | GNLHPDS | 1.013 |
| 1265 | **P** | 1262 | 1268 | NLHPDSA | 1.022 |
| 1266 | **D** | 1263 | 1269 | LHPDSAT | 1.026 |
| 1267 | **S** | 1264 | 1270 | HPDSATL | 1.023 |
| 1268 | **A** | 1265 | 1271 | PDSATLV | 1.007 |
| 1269 | **T** | 1266 | 1272 | DSATLVS | 0.986 |
| 1270 | **L** | 1267 | 1273 | SATLVSD | 0.984 |
| 1271 | **V** | 1268 | 1274 | ATLVSDI | 0.989 |
| 1272 | **S** | 1269 | 1275 | TLVSDID | 1.002 |
| 1273 | **D** | 1270 | 1276 | LVSDIDI | 1.012 |
| 1274 | **I** | 1271 | 1277 | VSDIDIT | 1.002 |
| 1275 | **D** | 1272 | 1278 | SDIDITF | 0.984 |
| 1276 | **I** | 1273 | 1279 | DIDITFL | 0.968 |
| 1277 | **T** | 1274 | 1280 | IDITFLK | 0.959 |
| 1278 | **F** | 1275 | 1281 | DITFLKK | 0.97 |
| 1279 | **L** | 1276 | 1282 | ITFLKKD | 0.998 |
| 1280 | **K** | 1277 | 1283 | TFLKKDA | 1.029 |
| 1281 | **K** | 1278 | 1284 | FLKKDAP | 1.046 |
| 1282 | **D** | 1279 | 1285 | LKKDAPY | 1.042 |
| 1283 | **A** | 1280 | 1286 | KKDAPYI | 1.014 |
| 1284 | **P** | 1281 | 1287 | KDAPYIV | 0.974 |
| 1285 | **Y** | 1282 | 1288 | DAPYIVG | 0.95 |
| 1286 | **I** | 1283 | 1289 | APYIVGD | 0.946 |
| 1287 | **V** | 1284 | 1290 | PYIVGDV | 0.959 |
| 1288 | **G** | 1285 | 1291 | YIVGDVV | 0.979 |
| 1289 | **D** | 1286 | 1292 | IVGDVVQ | 0.992 |
| 1290 | **V** | 1287 | 1293 | VGDVVQE | 0.995 |
| 1291 | **V** | 1288 | 1294 | GDVVQEG | 1.001 |
| 1292 | **Q** | 1289 | 1295 | DVVQEGV | 1.008 |
| 1293 | **E** | 1290 | 1296 | VVQEGVL | 1.011 |
| 1294 | **G** | 1291 | 1297 | VQEGVLT | 1.004 |
| 1295 | **V** | 1292 | 1298 | QEGVLTA | 0.984 |
| 1296 | **L** | 1293 | 1299 | EGVLTAV | 0.963 |
| 1297 | **T** | 1294 | 1300 | GVLTAVV | 0.943 |
| 1298 | **A** | 1295 | 1301 | VLTAVVI | 0.931 |
| 1299 | **V** | 1296 | 1302 | LTAVVIP | 0.933 |
| 1300 | **V** | 1297 | 1303 | TAVVIPT | 0.946 |
| 1301 | **I** | 1298 | 1304 | AVVIPTK | 0.973 |
| 1302 | **P** | 1299 | 1305 | VVIPTKK | 1.015 |
| 1303 | **T** | 1300 | 1306 | VIPTKKA | 1.045 |
| 1304 | **K** | 1301 | 1307 | IPTKKAG | 1.064 |
| 1305 | **K** | 1302 | 1308 | PTKKAGG | 1.074 |
| 1306 | **A** | 1303 | 1309 | TKKAGGT | 1.072 |
| 1307 | **G** | 1304 | 1310 | KKAGGTT | 1.075 |
| 1308 | **G** | 1305 | 1311 | KAGGTTE | 1.079 |
| 1309 | **T** | 1306 | 1312 | AGGTTEM | 1.062 |
| 1310 | **T** | 1307 | 1313 | GGTTEML | 1.037 |
| 1311 | **E** | 1308 | 1314 | GTTEMLA | 1.002 |
| 1312 | **M** | 1309 | 1315 | TTEMLAK | 0.968 |
| 1313 | **L** | 1310 | 1316 | TEMLAKA | 0.957 |
| 1314 | **A** | 1311 | 1317 | EMLAKAL | 0.958 |
| 1315 | **K** | 1312 | 1318 | MLAKALR | 0.972 |
| 1316 | **A** | 1313 | 1319 | LAKALRK | 0.987 |
| 1317 | **L** | 1314 | 1320 | AKALRKV | 0.998 |
| 1318 | **R** | 1315 | 1321 | KALRKVP | 1.015 |
| 1319 | **K** | 1316 | 1322 | ALRKVPT | 1.029 |
| 1320 | **V** | 1317 | 1323 | LRKVPTD | 1.039 |
| 1321 | **P** | 1318 | 1324 | RKVPTDN | 1.048 |
| 1322 | **T** | 1319 | 1325 | KVPTDNY | 1.04 |
| 1323 | **D** | 1320 | 1326 | VPTDNYI | 1.017 |
| 1324 | **N** | 1321 | 1327 | PTDNYIT | 0.999 |
| 1325 | **Y** | 1322 | 1328 | TDNYITT | 0.982 |
| 1326 | **I** | 1323 | 1329 | DNYITTY | 0.976 |
| 1327 | **T** | 1324 | 1330 | NYITTYP | 0.994 |
| 1328 | **T** | 1325 | 1331 | YITTYPG | 1.017 |
| 1329 | **Y** | 1326 | 1332 | ITTYPGQ | 1.043 |
| 1330 | **P** | 1327 | 1333 | TTYPGQG | 1.073 |
| 1331 | **G** | 1328 | 1334 | TYPGQGL | 1.084 |
| 1332 | **Q** | 1329 | 1335 | YPGQGLN | 1.08 |
| 1333 | **G** | 1330 | 1336 | PGQGLNG | 1.062 |
| 1334 | **L** | 1331 | 1337 | GQGLNGY | 1.033 |
| 1335 | **N** | 1332 | 1338 | QGLNGYT | 1.01 |
| 1336 | **G** | 1333 | 1339 | GLNGYTV | 0.993 |
| 1337 | **Y** | 1334 | 1340 | LNGYTVE | 0.984 |
| 1338 | **T** | 1335 | 1341 | NGYTVEE | 0.985 |
| 1339 | **V** | 1336 | 1342 | GYTVEEA | 0.995 |
| 1340 | **E** | 1337 | 1343 | YTVEEAK | 1.012 |
| 1341 | **E** | 1338 | 1344 | TVEEAKT | 1.03 |
| 1342 | **A** | 1339 | 1345 | VEEAKTV | 1.038 |
| 1343 | **K** | 1340 | 1346 | EEAKTVL | 1.033 |
| 1344 | **T** | 1341 | 1347 | EAKTVLK | 1.022 |
| 1345 | **V** | 1342 | 1348 | AKTVLKK | 1.012 |
| 1346 | **L** | 1343 | 1349 | KTVLKKC | 1.011 |
| 1347 | **K** | 1344 | 1350 | TVLKKCK | 1.023 |
| 1348 | **K** | 1345 | 1351 | VLKKCKS | 1.032 |
| 1349 | **C** | 1346 | 1352 | LKKCKSA | 1.031 |
| 1350 | **K** | 1347 | 1353 | KKCKSAF | 1.025 |
| 1351 | **S** | 1348 | 1354 | KCKSAFY | 0.996 |
| 1352 | **A** | 1349 | 1355 | CKSAFYI | 0.959 |
| 1353 | **F** | 1350 | 1356 | KSAFYIL | 0.93 |
| 1354 | **Y** | 1351 | 1357 | SAFYILP | 0.916 |
| 1355 | **I** | 1352 | 1358 | AFYILPS | 0.93 |
| 1356 | **L** | 1353 | 1359 | FYILPSI | 0.956 |
| 1357 | **P** | 1354 | 1360 | YILPSII | 0.977 |
| 1358 | **S** | 1355 | 1361 | ILPSIIS | 0.984 |
| 1359 | **I** | 1356 | 1362 | LPSIISN | 0.983 |
| 1360 | **I** | 1357 | 1363 | PSIISNE | 0.994 |
| 1361 | **S** | 1358 | 1364 | SIISNEK | 1.021 |
| 1362 | **N** | 1359 | 1365 | IISNEKQ | 1.058 |
| 1363 | **E** | 1360 | 1366 | ISNEKQE | 1.085 |
| 1364 | **K** | 1361 | 1367 | SNEKQEI | 1.087 |
| 1365 | **Q** | 1362 | 1368 | NEKQEIL | 1.069 |
| 1366 | **E** | 1363 | 1369 | EKQEILG | 1.035 |
| 1367 | **I** | 1364 | 1370 | KQEILGT | 1.007 |
| 1368 | **L** | 1365 | 1371 | QEILGTV | 0.998 |
| 1369 | **G** | 1366 | 1372 | EILGTVS | 0.994 |
| 1370 | **T** | 1367 | 1373 | ILGTVSW | 0.991 |
| 1371 | **V** | 1368 | 1374 | LGTVSWN | 0.98 |
| 1372 | **S** | 1369 | 1375 | GTVSWNL | 0.962 |
| 1373 | **W** | 1370 | 1376 | TVSWNLR | 0.952 |
| 1374 | **N** | 1371 | 1377 | VSWNLRE | 0.957 |
| 1375 | **L** | 1372 | 1378 | SWNLREM | 0.966 |
| 1376 | **R** | 1373 | 1379 | WNLREML | 0.972 |
| 1377 | **E** | 1374 | 1380 | NLREMLA | 0.963 |
| 1378 | **M** | 1375 | 1381 | LREMLAH | 0.939 |
| 1379 | **L** | 1376 | 1382 | REMLAHA | 0.924 |
| 1380 | **A** | 1377 | 1383 | EMLAHAE | 0.919 |
| 1381 | **H** | 1378 | 1384 | MLAHAEE | 0.935 |
| 1382 | **A** | 1379 | 1385 | LAHAEET | 0.971 |
| 1383 | **E** | 1380 | 1386 | AHAEETR | 1.008 |
| 1384 | **E** | 1381 | 1387 | HAEETRK | 1.04 |
| 1385 | **T** | 1382 | 1388 | AEETRKL | 1.052 |
| 1386 | **R** | 1383 | 1389 | EETRKLM | 1.039 |
| 1387 | **K** | 1384 | 1390 | ETRKLMP | 1.014 |
| 1388 | **L** | 1385 | 1391 | TRKLMPV | 0.976 |
| 1389 | **M** | 1386 | 1392 | RKLMPVC | 0.944 |
| 1390 | **P** | 1387 | 1393 | KLMPVCV | 0.93 |
| 1391 | **V** | 1388 | 1394 | LMPVCVE | 0.928 |
| 1392 | **C** | 1389 | 1395 | MPVCVET | 0.946 |
| 1393 | **V** | 1390 | 1396 | PVCVETK | 0.975 |
| 1394 | **E** | 1391 | 1397 | VCVETKA | 1.002 |
| 1395 | **T** | 1392 | 1398 | CVETKAI | 1.012 |
| 1396 | **K** | 1393 | 1399 | VETKAIV | 1.003 |
| 1397 | **A** | 1394 | 1400 | ETKAIVS | 0.983 |
| 1398 | **I** | 1395 | 1401 | TKAIVST | 0.968 |
| 1399 | **V** | 1396 | 1402 | KAIVSTI | 0.972 |
| 1400 | **S** | 1397 | 1403 | AIVSTIQ | 0.99 |
| 1401 | **T** | 1398 | 1404 | IVSTIQR | 1.011 |
| 1402 | **I** | 1399 | 1405 | VSTIQRK | 1.027 |
| 1403 | **Q** | 1400 | 1406 | STIQRKY | 1.032 |
| 1404 | **R** | 1401 | 1407 | TIQRKYK | 1.034 |
| 1405 | **K** | 1402 | 1408 | IQRKYKG | 1.037 |
| 1406 | **Y** | 1403 | 1409 | QRKYKGI | 1.033 |
| 1407 | **K** | 1404 | 1410 | RKYKGIK | 1.038 |
| 1408 | **G** | 1405 | 1411 | KYKGIKI | 1.034 |
| 1409 | **I** | 1406 | 1412 | YKGIKIQ | 1.029 |
| 1410 | **K** | 1407 | 1413 | KGIKIQE | 1.035 |
| 1411 | **I** | 1408 | 1414 | GIKIQEG | 1.034 |
| 1412 | **Q** | 1409 | 1415 | IKIQEGV | 1.033 |
| 1413 | **E** | 1410 | 1416 | KIQEGVV | 1.027 |
| 1414 | **G** | 1411 | 1417 | IQEGVVD | 1.005 |
| 1415 | **V** | 1412 | 1418 | QEGVVDY | 0.979 |
| 1416 | **V** | 1413 | 1419 | EGVVDYG | 0.957 |
| 1417 | **D** | 1414 | 1420 | GVVDYGA | 0.948 |
| 1418 | **Y** | 1415 | 1421 | VVDYGAR | 0.948 |
| 1419 | **G** | 1416 | 1422 | VDYGARF | 0.95 |
| 1420 | **A** | 1417 | 1423 | DYGARFY | 0.947 |
| 1421 | **R** | 1418 | 1424 | YGARFYF | 0.929 |
| 1422 | **F** | 1419 | 1425 | GARFYFY | 0.914 |
| 1423 | **Y** | 1420 | 1426 | ARFYFYT | 0.911 |
| 1424 | **F** | 1421 | 1427 | RFYFYTS | 0.935 |
| 1425 | **Y** | 1422 | 1428 | FYFYTSK | 0.977 |
| 1426 | **T** | 1423 | 1429 | YFYTSKT | 1.027 |
| 1427 | **S** | 1424 | 1430 | FYTSKTT | 1.067 |
| 1428 | **K** | 1425 | 1431 | YTSKTTV | 1.072 |
| 1429 | **T** | 1426 | 1432 | TSKTTVA | 1.057 |
| 1430 | **T** | 1427 | 1433 | SKTTVAS | 1.023 |
| 1431 | **V** | 1428 | 1434 | KTTVASL | 0.984 |
| 1432 | **A** | 1429 | 1435 | TTVASLI | 0.958 |
| 1433 | **S** | 1430 | 1436 | TVASLIN | 0.944 |
| 1434 | **L** | 1431 | 1437 | VASLINT | 0.948 |
| 1435 | **I** | 1432 | 1438 | ASLINTL | 0.958 |
| 1436 | **N** | 1433 | 1439 | SLINTLN | 0.977 |
| 1437 | **T** | 1434 | 1440 | LINTLND | 0.998 |
| 1438 | **L** | 1435 | 1441 | INTLNDL | 1.005 |
| 1439 | **N** | 1436 | 1442 | NTLNDLN | 1.015 |
| 1440 | **D** | 1437 | 1443 | TLNDLNE | 1.023 |
| 1441 | **L** | 1438 | 1444 | LNDLNET | 1.023 |
| 1442 | **N** | 1439 | 1445 | NDLNETL | 1.028 |
| 1443 | **E** | 1440 | 1446 | DLNETLV | 1.026 |
| 1444 | **T** | 1441 | 1447 | LNETLVT | 1.008 |
| 1445 | **L** | 1442 | 1448 | NETLVTM | 0.987 |
| 1446 | **V** | 1443 | 1449 | ETLVTMP | 0.963 |
| 1447 | **T** | 1444 | 1450 | TLVTMPL | 0.948 |
| 1448 | **M** | 1445 | 1451 | LVTMPLG | 0.941 |
| 1449 | **P** | 1446 | 1452 | VTMPLGY | 0.94 |
| 1450 | **L** | 1447 | 1453 | TMPLGYV | 0.941 |
| 1451 | **G** | 1448 | 1454 | MPLGYVT | 0.936 |
| 1452 | **Y** | 1449 | 1455 | PLGYVTH | 0.937 |
| 1453 | **V** | 1450 | 1456 | LGYVTHG | 0.938 |
| 1454 | **T** | 1451 | 1457 | GYVTHGL | 0.941 |
| 1455 | **H** | 1452 | 1458 | YVTHGLN | 0.947 |
| 1456 | **G** | 1453 | 1459 | VTHGLNL | 0.948 |
| 1457 | **L** | 1454 | 1460 | THGLNLE | 0.956 |
| 1458 | **N** | 1455 | 1461 | HGLNLEE | 0.966 |
| 1459 | **L** | 1456 | 1462 | GLNLEEA | 0.978 |
| 1460 | **E** | 1457 | 1463 | LNLEEAA | 0.989 |
| 1461 | **E** | 1458 | 1464 | NLEEAAR | 0.984 |
| 1462 | **A** | 1459 | 1465 | LEEAARY | 0.969 |
| 1463 | **A** | 1460 | 1466 | EEAARYM | 0.947 |
| 1464 | **R** | 1461 | 1467 | EAARYMR | 0.932 |
| 1465 | **Y** | 1462 | 1468 | AARYMRS | 0.934 |
| 1466 | **M** | 1463 | 1469 | ARYMRSL | 0.946 |
| 1467 | **R** | 1464 | 1470 | RYMRSLK | 0.975 |
| 1468 | **S** | 1465 | 1471 | YMRSLKV | 0.995 |
| 1469 | **L** | 1466 | 1472 | MRSLKVP | 1.0 |
| 1470 | **K** | 1467 | 1473 | RSLKVPA | 1.006 |
| 1471 | **V** | 1468 | 1474 | SLKVPAT | 0.993 |
| 1472 | **P** | 1469 | 1475 | LKVPATV | 0.983 |
| 1473 | **A** | 1470 | 1476 | KVPATVS | 0.979 |
| 1474 | **T** | 1471 | 1477 | VPATVSV | 0.967 |
| 1475 | **V** | 1472 | 1478 | PATVSVS | 0.97 |
| 1476 | **S** | 1473 | 1479 | ATVSVSS | 0.985 |
| 1477 | **V** | 1474 | 1480 | TVSVSSP | 1.008 |
| 1478 | **S** | 1475 | 1481 | VSVSSPD | 1.042 |
| 1479 | **S** | 1476 | 1482 | SVSSPDA | 1.062 |
| 1480 | **P** | 1477 | 1483 | VSSPDAV | 1.056 |
| 1481 | **D** | 1478 | 1484 | SSPDAVT | 1.033 |
| 1482 | **A** | 1479 | 1485 | SPDAVTA | 0.995 |
| 1483 | **V** | 1480 | 1486 | PDAVTAY | 0.963 |
| 1484 | **T** | 1481 | 1487 | DAVTAYN | 0.95 |
| 1485 | **A** | 1482 | 1488 | AVTAYNG | 0.952 |
| 1486 | **Y** | 1483 | 1489 | VTAYNGY | 0.962 |
| 1487 | **N** | 1484 | 1490 | TAYNGYL | 0.974 |
| 1488 | **G** | 1485 | 1491 | AYNGYLT | 0.985 |
| 1489 | **Y** | 1486 | 1492 | YNGYLTS | 0.996 |
| 1490 | **L** | 1487 | 1493 | NGYLTSS | 1.024 |
| 1491 | **T** | 1488 | 1494 | GYLTSSS | 1.063 |
| 1492 | **S** | 1489 | 1495 | YLTSSSK | 1.101 |
| 1493 | **S** | 1490 | 1496 | LTSSSKT | 1.126 |
| 1494 | **S** | 1491 | 1497 | TSSSKTP | 1.128 |
| 1495 | **K** | 1492 | 1498 | SSSKTPE | 1.113 |
| 1496 | **T** | 1493 | 1499 | SSKTPEE | 1.092 |
| 1497 | **P** | 1494 | 1500 | SKTPEEH | 1.069 |
| 1498 | **E** | 1495 | 1501 | KTPEEHF | 1.044 |
| 1499 | **E** | 1496 | 1502 | TPEEHFI | 1.012 |
| 1500 | **H** | 1497 | 1503 | PEEHFIE | 0.983 |
| 1501 | **F** | 1498 | 1504 | EEHFIET | 0.967 |
| 1502 | **I** | 1499 | 1505 | EHFIETI | 0.966 |
| 1503 | **E** | 1500 | 1506 | HFIETIS | 0.98 |
| 1504 | **T** | 1501 | 1507 | FIETISL | 0.989 |
| 1505 | **I** | 1502 | 1508 | IETISLA | 0.985 |
| 1506 | **S** | 1503 | 1509 | ETISLAG | 0.978 |
| 1507 | **L** | 1504 | 1510 | TISLAGS | 0.977 |
| 1508 | **A** | 1505 | 1511 | ISLAGSY | 0.981 |
| 1509 | **G** | 1506 | 1512 | SLAGSYK | 1.0 |
| 1510 | **S** | 1507 | 1513 | LAGSYKD | 1.019 |
| 1511 | **Y** | 1508 | 1514 | AGSYKDW | 1.023 |
| 1512 | **K** | 1509 | 1515 | GSYKDWS | 1.024 |
| 1513 | **D** | 1510 | 1516 | SYKDWSY | 1.01 |
| 1514 | **W** | 1511 | 1517 | YKDWSYS | 0.989 |
| 1515 | **S** | 1512 | 1518 | KDWSYSG | 0.99 |
| 1516 | **Y** | 1513 | 1519 | DWSYSGQ | 1.009 |
| 1517 | **S** | 1514 | 1520 | WSYSGQS | 1.048 |
| 1518 | **G** | 1515 | 1521 | SYSGQST | 1.091 |
| 1519 | **Q** | 1516 | 1522 | YSGQSTQ | 1.114 |
| 1520 | **S** | 1517 | 1523 | SGQSTQL | 1.109 |
| 1521 | **T** | 1518 | 1524 | GQSTQLG | 1.076 |
| 1522 | **Q** | 1519 | 1525 | QSTQLGI | 1.036 |
| 1523 | **L** | 1520 | 1526 | STQLGIE | 0.998 |
| 1524 | **G** | 1521 | 1527 | TQLGIEF | 0.969 |
| 1525 | **I** | 1522 | 1528 | QLGIEFL | 0.958 |
| 1526 | **E** | 1523 | 1529 | LGIEFLK | 0.956 |
| 1527 | **F** | 1524 | 1530 | GIEFLKR | 0.966 |
| 1528 | **L** | 1525 | 1531 | IEFLKRG | 0.995 |
| 1529 | **K** | 1526 | 1532 | EFLKRGD | 1.025 |
| 1530 | **R** | 1527 | 1533 | FLKRGDK | 1.051 |
| 1531 | **G** | 1528 | 1534 | LKRGDKS | 1.071 |
| 1532 | **D** | 1529 | 1535 | KRGDKSV | 1.064 |
| 1533 | **K** | 1530 | 1536 | RGDKSVY | 1.039 |
| 1534 | **S** | 1531 | 1537 | GDKSVYY | 1.004 |
| 1535 | **V** | 1532 | 1538 | DKSVYYT | 0.968 |
| 1536 | **Y** | 1533 | 1539 | KSVYYTS | 0.961 |
| 1537 | **Y** | 1534 | 1540 | SVYYTSN | 0.984 |
| 1538 | **T** | 1535 | 1541 | VYYTSNP | 1.024 |
| 1539 | **S** | 1536 | 1542 | YYTSNPT | 1.066 |
| 1540 | **N** | 1537 | 1543 | YTSNPTT | 1.086 |
| 1541 | **P** | 1538 | 1544 | TSNPTTF | 1.075 |
| 1542 | **T** | 1539 | 1545 | SNPTTFH | 1.046 |
| 1543 | **T** | 1540 | 1546 | NPTTFHL | 1.008 |
| 1544 | **F** | 1541 | 1547 | PTTFHLD | 0.981 |
| 1545 | **H** | 1542 | 1548 | TTFHLDG | 0.981 |
| 1546 | **L** | 1543 | 1549 | TFHLDGE | 0.999 |
| 1547 | **D** | 1544 | 1550 | FHLDGEV | 1.023 |
| 1548 | **G** | 1545 | 1551 | HLDGEVI | 1.032 |
| 1549 | **E** | 1546 | 1552 | LDGEVIT | 1.013 |
| 1550 | **V** | 1547 | 1553 | DGEVITF | 0.979 |
| 1551 | **I** | 1548 | 1554 | GEVITFD | 0.957 |
| 1552 | **T** | 1549 | 1555 | EVITFDN | 0.955 |
| 1553 | **F** | 1550 | 1556 | VITFDNL | 0.967 |
| 1554 | **D** | 1551 | 1557 | ITFDNLK | 0.996 |
| 1555 | **N** | 1552 | 1558 | TFDNLKT | 1.013 |
| 1556 | **L** | 1553 | 1559 | FDNLKTL | 1.019 |
| 1557 | **K** | 1554 | 1560 | DNLKTLL | 1.023 |
| 1558 | **T** | 1555 | 1561 | NLKTLLS | 1.007 |
| 1559 | **L** | 1556 | 1562 | LKTLLSL | 0.989 |
| 1560 | **L** | 1557 | 1563 | KTLLSLR | 0.978 |
| 1561 | **S** | 1558 | 1564 | TLLSLRE | 0.971 |
| 1562 | **L** | 1559 | 1565 | LLSLREV | 0.979 |
| 1563 | **R** | 1560 | 1566 | LSLREVR | 0.995 |
| 1564 | **E** | 1561 | 1567 | SLREVRT | 1.009 |
| 1565 | **V** | 1562 | 1568 | LREVRTI | 1.015 |
| 1566 | **R** | 1563 | 1569 | REVRTIK | 1.023 |
| 1567 | **T** | 1564 | 1570 | EVRTIKV | 1.021 |
| 1568 | **I** | 1565 | 1571 | VRTIKVF | 1.008 |
| 1569 | **K** | 1566 | 1572 | RTIKVFT | 1.001 |
| 1570 | **V** | 1567 | 1573 | TIKVFTT | 0.989 |
| 1571 | **F** | 1568 | 1574 | IKVFTTV | 0.986 |
| 1572 | **T** | 1569 | 1575 | KVFTTVD | 1.004 |
| 1573 | **T** | 1570 | 1576 | VFTTVDN | 1.015 |
| 1574 | **V** | 1571 | 1577 | FTTVDNI | 1.023 |
| 1575 | **D** | 1572 | 1578 | TTVDNIN | 1.025 |
| 1576 | **N** | 1573 | 1579 | TVDNINL | 1.008 |
| 1577 | **I** | 1574 | 1580 | VDNINLH | 0.991 |
| 1578 | **N** | 1575 | 1581 | DNINLHT | 0.979 |
| 1579 | **L** | 1576 | 1582 | NINLHTQ | 0.977 |
| 1580 | **H** | 1577 | 1583 | INLHTQV | 0.981 |
| 1581 | **T** | 1578 | 1584 | NLHTQVV | 0.986 |
| 1582 | **Q** | 1579 | 1585 | LHTQVVD | 0.981 |
| 1583 | **V** | 1580 | 1586 | HTQVVDM | 0.965 |
| 1584 | **V** | 1581 | 1587 | TQVVDMS | 0.951 |
| 1585 | **D** | 1582 | 1588 | QVVDMSM | 0.939 |
| 1586 | **M** | 1583 | 1589 | VVDMSMT | 0.935 |
| 1587 | **S** | 1584 | 1590 | VDMSMTY | 0.937 |
| 1588 | **M** | 1585 | 1591 | DMSMTYG | 0.947 |
| 1589 | **T** | 1586 | 1592 | MSMTYGQ | 0.969 |
| 1590 | **Y** | 1587 | 1593 | SMTYGQQ | 0.997 |
| 1591 | **G** | 1588 | 1594 | MTYGQQF | 1.022 |
| 1592 | **Q** | 1589 | 1595 | TYGQQFG | 1.039 |
| 1593 | **Q** | 1590 | 1596 | YGQQFGP | 1.036 |
| 1594 | **F** | 1591 | 1597 | GQQFGPT | 1.029 |
| 1595 | **G** | 1592 | 1598 | QQFGPTY | 1.024 |
| 1596 | **P** | 1593 | 1599 | QFGPTYL | 1.013 |
| 1597 | **T** | 1594 | 1600 | FGPTYLD | 1.011 |
| 1598 | **Y** | 1595 | 1601 | GPTYLDG | 1.008 |
| 1599 | **L** | 1596 | 1602 | PTYLDGA | 1.011 |
| 1600 | **D** | 1597 | 1603 | TYLDGAD | 1.018 |
| 1601 | **G** | 1598 | 1604 | YLDGADV | 1.016 |
| 1602 | **A** | 1599 | 1605 | LDGADVT | 1.015 |
| 1603 | **D** | 1600 | 1606 | DGADVTK | 1.01 |
| 1604 | **V** | 1601 | 1607 | GADVTKI | 1.01 |
| 1605 | **T** | 1602 | 1608 | ADVTKIK | 1.024 |
| 1606 | **K** | 1603 | 1609 | DVTKIKP | 1.039 |
| 1607 | **I** | 1604 | 1610 | VTKIKPH | 1.046 |
| 1608 | **K** | 1605 | 1611 | TKIKPHN | 1.048 |
| 1609 | **P** | 1606 | 1612 | KIKPHNS | 1.042 |
| 1610 | **H** | 1607 | 1613 | IKPHNSH | 1.028 |
| 1611 | **N** | 1608 | 1614 | KPHNSHE | 1.023 |
| 1612 | **S** | 1609 | 1615 | PHNSHEG | 1.026 |
| 1613 | **H** | 1610 | 1616 | HNSHEGK | 1.034 |
| 1614 | **E** | 1611 | 1617 | NSHEGKT | 1.053 |
| 1615 | **G** | 1612 | 1618 | SHEGKTF | 1.061 |
| 1616 | **K** | 1613 | 1619 | HEGKTFY | 1.042 |
| 1617 | **T** | 1614 | 1620 | EGKTFYV | 1.008 |
| 1618 | **F** | 1615 | 1621 | GKTFYVL | 0.964 |
| 1619 | **Y** | 1616 | 1622 | KTFYVLP | 0.939 |
| 1620 | **V** | 1617 | 1623 | TFYVLPN | 0.95 |
| 1621 | **L** | 1618 | 1624 | FYVLPND | 0.981 |
| 1622 | **P** | 1619 | 1625 | YVLPNDD | 1.021 |
| 1623 | **N** | 1620 | 1626 | VLPNDDT | 1.048 |
| 1624 | **D** | 1621 | 1627 | LPNDDTL | 1.049 |
| 1625 | **D** | 1622 | 1628 | PNDDTLR | 1.034 |
| 1626 | **T** | 1623 | 1629 | NDDTLRV | 1.011 |
| 1627 | **L** | 1624 | 1630 | DDTLRVE | 0.983 |
| 1628 | **R** | 1625 | 1631 | DTLRVEA | 0.962 |
| 1629 | **V** | 1626 | 1632 | TLRVEAF | 0.951 |
| 1630 | **E** | 1627 | 1633 | LRVEAFE | 0.94 |
| 1631 | **A** | 1628 | 1634 | RVEAFEY | 0.936 |
| 1632 | **F** | 1629 | 1635 | VEAFEYY | 0.927 |
| 1633 | **E** | 1630 | 1636 | EAFEYYH | 0.919 |
| 1634 | **Y** | 1631 | 1637 | AFEYYHT | 0.922 |
| 1635 | **Y** | 1632 | 1638 | FEYYHTT | 0.934 |
| 1636 | **H** | 1633 | 1639 | EYYHTTD | 0.965 |
| 1637 | **T** | 1634 | 1640 | YYHTTDP | 1.0 |
| 1638 | **T** | 1635 | 1641 | YHTTDPS | 1.028 |
| 1639 | **D** | 1636 | 1642 | HTTDPSF | 1.036 |
| 1640 | **P** | 1637 | 1643 | TTDPSFL | 1.028 |
| 1641 | **S** | 1638 | 1644 | TDPSFLG | 1.012 |
| 1642 | **F** | 1639 | 1645 | DPSFLGR | 0.996 |
| 1643 | **L** | 1640 | 1646 | PSFLGRY | 0.99 |
| 1644 | **G** | 1641 | 1647 | SFLGRYM | 0.983 |
| 1645 | **R** | 1642 | 1648 | FLGRYMS | 0.969 |
| 1646 | **Y** | 1643 | 1649 | LGRYMSA | 0.952 |
| 1647 | **M** | 1644 | 1650 | GRYMSAL | 0.935 |
| 1648 | **S** | 1645 | 1651 | RYMSALN | 0.929 |
| 1649 | **A** | 1646 | 1652 | YMSALNH | 0.933 |
| 1650 | **L** | 1647 | 1653 | MSALNHT | 0.95 |
| 1651 | **N** | 1648 | 1654 | SALNHTK | 0.972 |
| 1652 | **H** | 1649 | 1655 | ALNHTKK | 1.0 |
| 1653 | **T** | 1650 | 1656 | LNHTKKW | 1.021 |
| 1654 | **K** | 1651 | 1657 | NHTKKWK | 1.036 |
| 1655 | **K** | 1652 | 1658 | HTKKWKY | 1.034 |
| 1656 | **W** | 1653 | 1659 | TKKWKYP | 1.023 |
| 1657 | **K** | 1654 | 1660 | KKWKYPQ | 1.021 |
| 1658 | **Y** | 1655 | 1661 | KWKYPQV | 1.015 |
| 1659 | **P** | 1656 | 1662 | WKYPQVN | 1.02 |
| 1660 | **Q** | 1657 | 1663 | KYPQVNG | 1.022 |
| 1661 | **V** | 1658 | 1664 | YPQVNGL | 1.013 |
| 1662 | **N** | 1659 | 1665 | PQVNGLT | 1.014 |
| 1663 | **G** | 1660 | 1666 | QVNGLTS | 1.014 |
| 1664 | **L** | 1661 | 1667 | VNGLTSI | 1.015 |
| 1665 | **T** | 1662 | 1668 | NGLTSIK | 1.025 |
| 1666 | **S** | 1663 | 1669 | GLTSIKW | 1.022 |
| 1667 | **I** | 1664 | 1670 | LTSIKWA | 1.011 |
| 1668 | **K** | 1665 | 1671 | TSIKWAD | 1.007 |
| 1669 | **W** | 1666 | 1672 | SIKWADN | 1.002 |
| 1670 | **A** | 1667 | 1673 | IKWADNN | 1.01 |
| 1671 | **D** | 1668 | 1674 | KWADNNC | 1.02 |
| 1672 | **N** | 1669 | 1675 | WADNNCY | 1.01 |
| 1673 | **N** | 1670 | 1676 | ADNNCYL | 0.983 |
| 1674 | **C** | 1671 | 1677 | DNNCYLA | 0.947 |
| 1675 | **Y** | 1672 | 1678 | NNCYLAT | 0.919 |
| 1676 | **L** | 1673 | 1679 | NCYLATA | 0.913 |
| 1677 | **A** | 1674 | 1680 | CYLATAL | 0.92 |
| 1678 | **T** | 1675 | 1681 | YLATALL | 0.931 |
| 1679 | **A** | 1676 | 1682 | LATALLT | 0.939 |
| 1680 | **L** | 1677 | 1683 | ATALLTL | 0.941 |
| 1681 | **L** | 1678 | 1684 | TALLTLQ | 0.95 |
| 1682 | **T** | 1679 | 1685 | ALLTLQQ | 0.962 |
| 1683 | **L** | 1680 | 1686 | LLTLQQI | 0.978 |
| 1684 | **Q** | 1681 | 1687 | LTLQQIE | 0.991 |
| 1685 | **Q** | 1682 | 1688 | TLQQIEL | 0.994 |
| 1686 | **I** | 1683 | 1689 | LQQIELK | 0.994 |
| 1687 | **E** | 1684 | 1690 | QQIELKF | 0.985 |
| 1688 | **L** | 1685 | 1691 | QIELKFN | 0.983 |
| 1689 | **K** | 1686 | 1692 | IELKFNP | 0.991 |
| 1690 | **F** | 1687 | 1693 | ELKFNPP | 0.998 |
| 1691 | **N** | 1688 | 1694 | LKFNPPA | 1.011 |
| 1692 | **P** | 1689 | 1695 | KFNPPAL | 1.016 |
| 1693 | **P** | 1690 | 1696 | FNPPALQ | 1.015 |
| 1694 | **A** | 1691 | 1697 | NPPALQD | 1.011 |
| 1695 | **L** | 1692 | 1698 | PPALQDA | 1.007 |
| 1696 | **Q** | 1693 | 1699 | PALQDAY | 0.998 |
| 1697 | **D** | 1694 | 1700 | ALQDAYY | 0.984 |
| 1698 | **A** | 1695 | 1701 | LQDAYYR | 0.959 |
| 1699 | **Y** | 1696 | 1702 | QDAYYRA | 0.939 |
| 1700 | **Y** | 1697 | 1703 | DAYYRAR | 0.931 |
| 1701 | **R** | 1698 | 1704 | AYYRARA | 0.936 |
| 1702 | **A** | 1699 | 1705 | YYRARAG | 0.962 |
| 1703 | **R** | 1700 | 1706 | YRARAGE | 0.981 |
| 1704 | **A** | 1701 | 1707 | RARAGEA | 1.0 |
| 1705 | **G** | 1702 | 1708 | ARAGEAA | 1.005 |
| 1706 | **E** | 1703 | 1709 | RAGEAAN | 0.995 |
| 1707 | **A** | 1704 | 1710 | AGEAANF | 0.977 |
| 1708 | **A** | 1705 | 1711 | GEAANFC | 0.955 |
| 1709 | **N** | 1706 | 1712 | EAANFCA | 0.935 |
| 1710 | **F** | 1707 | 1713 | AANFCAL | 0.922 |
| 1711 | **C** | 1708 | 1714 | ANFCALI | 0.915 |
| 1712 | **A** | 1709 | 1715 | NFCALIL | 0.908 |
| 1713 | **L** | 1710 | 1716 | FCALILA | 0.904 |
| 1714 | **I** | 1711 | 1717 | CALILAY | 0.896 |
| 1715 | **L** | 1712 | 1718 | ALILAYC | 0.891 |
| 1716 | **A** | 1713 | 1719 | LILAYCN | 0.892 |
| 1717 | **Y** | 1714 | 1720 | ILAYCNK | 0.905 |
| 1718 | **C** | 1715 | 1721 | LAYCNKT | 0.936 |
| 1719 | **N** | 1716 | 1722 | AYCNKTV | 0.974 |
| 1720 | **K** | 1717 | 1723 | YCNKTVG | 1.009 |
| 1721 | **T** | 1718 | 1724 | CNKTVGE | 1.027 |
| 1722 | **V** | 1719 | 1725 | NKTVGEL | 1.027 |
| 1723 | **G** | 1720 | 1726 | KTVGELG | 1.025 |
| 1724 | **E** | 1721 | 1727 | TVGELGD | 1.022 |
| 1725 | **L** | 1722 | 1728 | VGELGDV | 1.02 |
| 1726 | **G** | 1723 | 1729 | GELGDVR | 1.028 |
| 1727 | **D** | 1724 | 1730 | ELGDVRE | 1.034 |
| 1728 | **V** | 1725 | 1731 | LGDVRET | 1.036 |
| 1729 | **R** | 1726 | 1732 | GDVRETM | 1.038 |
| 1730 | **E** | 1727 | 1733 | DVRETMS | 1.03 |
| 1731 | **T** | 1728 | 1734 | VRETMSY | 1.009 |
| 1732 | **M** | 1729 | 1735 | RETMSYL | 0.982 |
| 1733 | **S** | 1730 | 1736 | ETMSYLF | 0.955 |
| 1734 | **Y** | 1731 | 1737 | TMSYLFQ | 0.932 |
| 1735 | **L** | 1732 | 1738 | MSYLFQH | 0.92 |
| 1736 | **F** | 1733 | 1739 | SYLFQHA | 0.919 |
| 1737 | **Q** | 1734 | 1740 | YLFQHAN | 0.92 |
| 1738 | **H** | 1735 | 1741 | LFQHANL | 0.93 |
| 1739 | **A** | 1736 | 1742 | FQHANLD | 0.946 |
| 1740 | **N** | 1737 | 1743 | QHANLDS | 0.967 |
| 1741 | **L** | 1738 | 1744 | HANLDSC | 0.989 |
| 1742 | **D** | 1739 | 1745 | ANLDSCK | 1.013 |
| 1743 | **S** | 1740 | 1746 | NLDSCKR | 1.025 |
| 1744 | **C** | 1741 | 1747 | LDSCKRV | 1.022 |
| 1745 | **K** | 1742 | 1748 | DSCKRVL | 1.018 |
| 1746 | **R** | 1743 | 1749 | SCKRVLN | 0.997 |
| 1747 | **V** | 1744 | 1750 | CKRVLNV | 0.974 |
| 1748 | **L** | 1745 | 1751 | KRVLNVV | 0.958 |
| 1749 | **N** | 1746 | 1752 | RVLNVVC | 0.936 |
| 1750 | **V** | 1747 | 1753 | VLNVVCK | 0.933 |
| 1751 | **V** | 1748 | 1754 | LNVVCKT | 0.944 |
| 1752 | **C** | 1749 | 1755 | NVVCKTC | 0.959 |
| 1753 | **K** | 1750 | 1756 | VVCKTCG | 0.989 |
| 1754 | **T** | 1751 | 1757 | VCKTCGQ | 1.015 |
| 1755 | **C** | 1752 | 1758 | CKTCGQQ | 1.041 |
| 1756 | **G** | 1753 | 1759 | KTCGQQQ | 1.076 |
| 1757 | **Q** | 1754 | 1760 | TCGQQQT | 1.103 |
| 1758 | **Q** | 1755 | 1761 | CGQQQTT | 1.118 |
| 1759 | **Q** | 1756 | 1762 | GQQQTTL | 1.113 |
| 1760 | **T** | 1757 | 1763 | QQQTTLK | 1.091 |
| 1761 | **T** | 1758 | 1764 | QQTTLKG | 1.064 |
| 1762 | **L** | 1759 | 1765 | QTTLKGV | 1.04 |
| 1763 | **K** | 1760 | 1766 | TTLKGVE | 1.027 |
| 1764 | **G** | 1761 | 1767 | TLKGVEA | 1.01 |
| 1765 | **V** | 1762 | 1768 | LKGVEAV | 0.987 |
| 1766 | **E** | 1763 | 1769 | KGVEAVM | 0.957 |
| 1767 | **A** | 1764 | 1770 | GVEAVMY | 0.923 |
| 1768 | **V** | 1765 | 1771 | VEAVMYM | 0.893 |
| 1769 | **M** | 1766 | 1772 | EAVMYMG | 0.879 |
| 1770 | **Y** | 1767 | 1773 | AVMYMGT | 0.891 |
| 1771 | **M** | 1768 | 1774 | VMYMGTL | 0.917 |
| 1772 | **G** | 1769 | 1775 | MYMGTLS | 0.953 |
| 1773 | **T** | 1770 | 1776 | YMGTLSY | 0.975 |
| 1774 | **L** | 1771 | 1777 | MGTLSYE | 0.981 |
| 1775 | **S** | 1772 | 1778 | GTLSYEQ | 0.983 |
| 1776 | **Y** | 1773 | 1779 | TLSYEQF | 0.981 |
| 1777 | **E** | 1774 | 1780 | LSYEQFK | 0.992 |
| 1778 | **Q** | 1775 | 1781 | SYEQFKK | 1.007 |
| 1779 | **F** | 1776 | 1782 | YEQFKKG | 1.019 |
| 1780 | **K** | 1777 | 1783 | EQFKKGV | 1.035 |
| 1781 | **K** | 1778 | 1784 | QFKKGVQ | 1.03 |
| 1782 | **G** | 1779 | 1785 | FKKGVQI | 1.016 |
| 1783 | **V** | 1780 | 1786 | KKGVQIP | 0.995 |
| 1784 | **Q** | 1781 | 1787 | KGVQIPC | 0.968 |
| 1785 | **I** | 1782 | 1788 | GVQIPCT | 0.957 |
| 1786 | **P** | 1783 | 1789 | VQIPCTC | 0.95 |
| 1787 | **C** | 1784 | 1790 | QIPCTCG | 0.956 |
| 1788 | **T** | 1785 | 1791 | IPCTCGK | 0.971 |
| 1789 | **C** | 1786 | 1792 | PCTCGKQ | 0.99 |
| 1790 | **G** | 1787 | 1793 | CTCGKQA | 1.016 |
| 1791 | **K** | 1788 | 1794 | TCGKQAT | 1.036 |
| 1792 | **Q** | 1789 | 1795 | CGKQATK | 1.046 |
| 1793 | **A** | 1790 | 1796 | GKQATKY | 1.045 |
| 1794 | **T** | 1791 | 1797 | KQATKYL | 1.031 |
| 1795 | **K** | 1792 | 1798 | QATKYLV | 1.009 |
| 1796 | **Y** | 1793 | 1799 | ATKYLVQ | 0.985 |
| 1797 | **L** | 1794 | 1800 | TKYLVQQ | 0.981 |
| 1798 | **V** | 1795 | 1801 | KYLVQQE | 0.995 |
| 1799 | **Q** | 1796 | 1802 | YLVQQES | 1.032 |
| 1800 | **Q** | 1797 | 1803 | LVQQESP | 1.077 |
| 1801 | **E** | 1798 | 1804 | VQQESPF | 1.09 |
| 1802 | **S** | 1799 | 1805 | QQESPFV | 1.082 |
| 1803 | **P** | 1800 | 1806 | QESPFVM | 1.035 |
| 1804 | **F** | 1801 | 1807 | ESPFVMM | 0.972 |
| 1805 | **V** | 1802 | 1808 | SPFVMMS | 0.924 |
| 1806 | **M** | 1803 | 1809 | PFVMMSA | 0.896 |
| 1807 | **M** | 1804 | 1810 | FVMMSAP | 0.908 |
| 1808 | **S** | 1805 | 1811 | VMMSAPP | 0.949 |
| 1809 | **A** | 1806 | 1812 | MMSAPPA | 0.995 |
| 1810 | **P** | 1807 | 1813 | MSAPPAQ | 1.025 |
| 1811 | **P** | 1808 | 1814 | SAPPAQY | 1.028 |
| 1812 | **A** | 1809 | 1815 | APPAQYE | 1.009 |
| 1813 | **Q** | 1810 | 1816 | PPAQYEL | 0.977 |
| 1814 | **Y** | 1811 | 1817 | PAQYELK | 0.966 |
| 1815 | **E** | 1812 | 1818 | AQYELKH | 0.964 |
| 1816 | **L** | 1813 | 1819 | QYELKHG | 0.978 |
| 1817 | **K** | 1814 | 1820 | YELKHGT | 1.002 |
| 1818 | **H** | 1815 | 1821 | ELKHGTF | 1.008 |
| 1819 | **G** | 1816 | 1822 | LKHGTFT | 1.009 |
| 1820 | **T** | 1817 | 1823 | KHGTFTC | 0.993 |
| 1821 | **F** | 1818 | 1824 | HGTFTCA | 0.965 |
| 1822 | **T** | 1819 | 1825 | GTFTCAS | 0.953 |
| 1823 | **C** | 1820 | 1826 | TFTCASE | 0.95 |
| 1824 | **A** | 1821 | 1827 | FTCASEY | 0.962 |
| 1825 | **S** | 1822 | 1828 | TCASEYT | 0.988 |
| 1826 | **E** | 1823 | 1829 | CASEYTG | 1.012 |
| 1827 | **Y** | 1824 | 1830 | ASEYTGN | 1.027 |
| 1828 | **T** | 1825 | 1831 | SEYTGNY | 1.038 |
| 1829 | **G** | 1826 | 1832 | EYTGNYQ | 1.032 |
| 1830 | **N** | 1827 | 1833 | YTGNYQC | 1.008 |
| 1831 | **Y** | 1828 | 1834 | TGNYQCG | 0.981 |
| 1832 | **Q** | 1829 | 1835 | GNYQCGH | 0.953 |
| 1833 | **C** | 1830 | 1836 | NYQCGHY | 0.939 |
| 1834 | **G** | 1831 | 1837 | YQCGHYK | 0.942 |
| 1835 | **H** | 1832 | 1838 | QCGHYKH | 0.952 |
| 1836 | **Y** | 1833 | 1839 | CGHYKHI | 0.959 |
| 1837 | **K** | 1834 | 1840 | GHYKHIT | 0.971 |
| 1838 | **H** | 1835 | 1841 | HYKHITS | 0.984 |
| 1839 | **I** | 1836 | 1842 | YKHITSK | 1.003 |
| 1840 | **T** | 1837 | 1843 | KHITSKE | 1.039 |
| 1841 | **S** | 1838 | 1844 | HITSKET | 1.068 |
| 1842 | **K** | 1839 | 1845 | ITSKETL | 1.076 |
| 1843 | **E** | 1840 | 1846 | TSKETLY | 1.06 |
| 1844 | **T** | 1841 | 1847 | SKETLYC | 1.02 |
| 1845 | **L** | 1842 | 1848 | KETLYCI | 0.971 |
| 1846 | **Y** | 1843 | 1849 | ETLYCID | 0.942 |
| 1847 | **C** | 1844 | 1850 | TLYCIDG | 0.943 |
| 1848 | **I** | 1845 | 1851 | LYCIDGA | 0.955 |
| 1849 | **D** | 1846 | 1852 | YCIDGAL | 0.979 |
| 1850 | **G** | 1847 | 1853 | CIDGALL | 0.987 |
| 1851 | **A** | 1848 | 1854 | IDGALLT | 0.982 |
| 1852 | **L** | 1849 | 1855 | DGALLTK | 0.986 |
| 1853 | **L** | 1850 | 1856 | GALLTKS | 1.003 |
| 1854 | **T** | 1851 | 1857 | ALLTKSS | 1.041 |
| 1855 | **K** | 1852 | 1858 | LLTKSSE | 1.078 |
| 1856 | **S** | 1853 | 1859 | LTKSSEY | 1.097 |
| 1857 | **S** | 1854 | 1860 | TKSSEYK | 1.096 |
| 1858 | **E** | 1855 | 1861 | KSSEYKG | 1.079 |
| 1859 | **Y** | 1856 | 1862 | SSEYKGP | 1.067 |
| 1860 | **K** | 1857 | 1863 | SEYKGPI | 1.066 |
| 1861 | **G** | 1858 | 1864 | EYKGPIT | 1.068 |
| 1862 | **P** | 1859 | 1865 | YKGPITD | 1.068 |
| 1863 | **I** | 1860 | 1866 | KGPITDV | 1.055 |
| 1864 | **T** | 1861 | 1867 | GPITDVF | 1.035 |
| 1865 | **D** | 1862 | 1868 | PITDVFY | 1.009 |
| 1866 | **V** | 1863 | 1869 | ITDVFYK | 0.985 |
| 1867 | **F** | 1864 | 1870 | TDVFYKE | 0.982 |
| 1868 | **Y** | 1865 | 1871 | DVFYKEN | 0.997 |
| 1869 | **K** | 1866 | 1872 | VFYKENS | 1.027 |
| 1870 | **E** | 1867 | 1873 | FYKENSY | 1.05 |
| 1871 | **N** | 1868 | 1874 | YKENSYT | 1.06 |
| 1872 | **S** | 1869 | 1875 | KENSYTT | 1.054 |
| 1873 | **Y** | 1870 | 1876 | ENSYTTT | 1.042 |
| 1874 | **T** | 1871 | 1877 | NSYTTTI | 1.039 |
| 1875 | **T** | 1872 | 1878 | SYTTTIK | 1.041 |
| 1876 | **T** | 1873 | 1879 | YTTTIKP | 1.046 |
| 1877 | **I** | 1874 | 1880 | TTTIKPV | 1.047 |
| 1878 | **K** | 1875 | 1881 | TTIKPVT | 1.041 |
| 1879 | **P** | 1876 | 1882 | TIKPVTY | 1.026 |
| 1880 | **V** | 1877 | 1883 | IKPVTYK | 1.008 |
| 1881 | **T** | 1878 | 1884 | KPVTYKL | 0.994 |
| 1882 | **Y** | 1879 | 1885 | PVTYKLD | 0.993 |
| 1883 | **K** | 1880 | 1886 | VTYKLDG | 1.005 |
| 1884 | **L** | 1881 | 1887 | TYKLDGV | 1.011 |
| 1885 | **D** | 1882 | 1888 | YKLDGVV | 1.014 |
| 1886 | **G** | 1883 | 1889 | KLDGVVC | 0.994 |
| 1887 | **V** | 1884 | 1890 | LDGVVCT | 0.97 |
| 1888 | **V** | 1885 | 1891 | DGVVCTE | 0.961 |
| 1889 | **C** | 1886 | 1892 | GVVCTEI | 0.961 |
| 1890 | **T** | 1887 | 1893 | VVCTEID | 0.987 |
| 1891 | **E** | 1888 | 1894 | VCTEIDP | 1.013 |
| 1892 | **I** | 1889 | 1895 | CTEIDPK | 1.035 |
| 1893 | **D** | 1890 | 1896 | TEIDPKL | 1.049 |
| 1894 | **P** | 1891 | 1897 | EIDPKLD | 1.05 |
| 1895 | **K** | 1892 | 1898 | IDPKLDN | 1.047 |
| 1896 | **L** | 1893 | 1899 | DPKLDNY | 1.033 |
| 1897 | **D** | 1894 | 1900 | PKLDNYY | 1.018 |
| 1898 | **N** | 1895 | 1901 | KLDNYYK | 1.002 |
| 1899 | **Y** | 1896 | 1902 | LDNYYKK | 0.996 |
| 1900 | **Y** | 1897 | 1903 | DNYYKKD | 1.005 |
| 1901 | **K** | 1898 | 1904 | NYYKKDN | 1.028 |
| 1902 | **K** | 1899 | 1905 | YYKKDNS | 1.049 |
| 1903 | **D** | 1900 | 1906 | YKKDNSY | 1.055 |
| 1904 | **N** | 1901 | 1907 | KKDNSYF | 1.047 |
| 1905 | **S** | 1902 | 1908 | KDNSYFT | 1.023 |
| 1906 | **Y** | 1903 | 1909 | DNSYFTE | 1.004 |
| 1907 | **F** | 1904 | 1910 | NSYFTEQ | 1.01 |
| 1908 | **T** | 1905 | 1911 | SYFTEQP | 1.034 |
| 1909 | **E** | 1906 | 1912 | YFTEQPI | 1.059 |
| 1910 | **Q** | 1907 | 1913 | FTEQPID | 1.072 |
| 1911 | **P** | 1908 | 1914 | TEQPIDL | 1.057 |
| 1912 | **I** | 1909 | 1915 | EQPIDLV | 1.021 |
| 1913 | **D** | 1910 | 1916 | QPIDLVP | 0.993 |
| 1914 | **L** | 1911 | 1917 | PIDLVPN | 0.987 |
| 1915 | **V** | 1912 | 1918 | IDLVPNQ | 1.007 |
| 1916 | **P** | 1913 | 1919 | DLVPNQP | 1.046 |
| 1917 | **N** | 1914 | 1920 | LVPNQPY | 1.073 |
| 1918 | **Q** | 1915 | 1921 | VPNQPYP | 1.086 |
| 1919 | **P** | 1916 | 1922 | PNQPYPN | 1.076 |
| 1920 | **Y** | 1917 | 1923 | NQPYPNA | 1.053 |
| 1921 | **P** | 1918 | 1924 | QPYPNAS | 1.036 |
| 1922 | **N** | 1919 | 1925 | PYPNASF | 1.012 |
| 1923 | **A** | 1920 | 1926 | YPNASFD | 1.002 |
| 1924 | **S** | 1921 | 1927 | PNASFDN | 0.993 |
| 1925 | **F** | 1922 | 1928 | NASFDNF | 0.987 |
| 1926 | **D** | 1923 | 1929 | ASFDNFK | 0.998 |
| 1927 | **N** | 1924 | 1930 | SFDNFKF | 0.993 |
| 1928 | **F** | 1925 | 1931 | FDNFKFV | 0.985 |
| 1929 | **K** | 1926 | 1932 | DNFKFVC | 0.972 |
| 1930 | **F** | 1927 | 1933 | NFKFVCD | 0.954 |
| 1931 | **V** | 1928 | 1934 | FKFVCDN | 0.953 |
| 1932 | **C** | 1929 | 1935 | KFVCDNI | 0.963 |
| 1933 | **D** | 1930 | 1936 | FVCDNIK | 0.988 |
| 1934 | **N** | 1931 | 1937 | VCDNIKF | 1.0 |
| 1935 | **I** | 1932 | 1938 | CDNIKFA | 1.001 |
| 1936 | **K** | 1933 | 1939 | DNIKFAD | 1.003 |
| 1937 | **F** | 1934 | 1940 | NIKFADD | 0.996 |
| 1938 | **A** | 1935 | 1941 | IKFADDL | 1.003 |
| 1939 | **D** | 1936 | 1942 | KFADDLN | 1.018 |
| 1940 | **D** | 1937 | 1943 | FADDLNQ | 1.023 |
| 1941 | **L** | 1938 | 1944 | ADDLNQL | 1.019 |
| 1942 | **N** | 1939 | 1945 | DDLNQLT | 1.016 |
| 1943 | **Q** | 1940 | 1946 | DLNQLTG | 1.012 |
| 1944 | **L** | 1941 | 1947 | LNQLTGY | 1.008 |
| 1945 | **T** | 1942 | 1948 | NQLTGYK | 1.019 |
| 1946 | **G** | 1943 | 1949 | QLTGYKK | 1.026 |
| 1947 | **Y** | 1944 | 1950 | LTGYKKP | 1.035 |
| 1948 | **K** | 1945 | 1951 | TGYKKPA | 1.052 |
| 1949 | **K** | 1946 | 1952 | GYKKPAS | 1.06 |
| 1950 | **P** | 1947 | 1953 | YKKPASR | 1.063 |
| 1951 | **A** | 1948 | 1954 | KKPASRE | 1.059 |
| 1952 | **S** | 1949 | 1955 | KPASREL | 1.046 |
| 1953 | **R** | 1950 | 1956 | PASRELK | 1.035 |
| 1954 | **E** | 1951 | 1957 | ASRELKV | 1.024 |
| 1955 | **L** | 1952 | 1958 | SRELKVT | 1.011 |
| 1956 | **K** | 1953 | 1959 | RELKVTF | 0.998 |
| 1957 | **V** | 1954 | 1960 | ELKVTFF | 0.975 |
| 1958 | **T** | 1955 | 1961 | LKVTFFP | 0.963 |
| 1959 | **F** | 1956 | 1962 | KVTFFPD | 0.967 |
| 1960 | **F** | 1957 | 1963 | VTFFPDL | 0.977 |
| 1961 | **P** | 1958 | 1964 | TFFPDLN | 1.003 |
| 1962 | **D** | 1959 | 1965 | FFPDLNG | 1.025 |
| 1963 | **L** | 1960 | 1966 | FPDLNGD | 1.038 |
| 1964 | **N** | 1961 | 1967 | PDLNGDV | 1.045 |
| 1965 | **G** | 1962 | 1968 | DLNGDVV | 1.04 |
| 1966 | **D** | 1963 | 1969 | LNGDVVA | 1.016 |
| 1967 | **V** | 1964 | 1970 | NGDVVAI | 0.98 |
| 1968 | **V** | 1965 | 1971 | GDVVAID | 0.947 |
| 1969 | **A** | 1966 | 1972 | DVVAIDY | 0.922 |
| 1970 | **I** | 1967 | 1973 | VVAIDYK | 0.923 |
| 1971 | **D** | 1968 | 1974 | VAIDYKH | 0.941 |
| 1972 | **Y** | 1969 | 1975 | AIDYKHY | 0.958 |
| 1973 | **K** | 1970 | 1976 | IDYKHYT | 0.977 |
| 1974 | **H** | 1971 | 1977 | DYKHYTP | 0.986 |
| 1975 | **Y** | 1972 | 1978 | YKHYTPS | 0.998 |
| 1976 | **T** | 1973 | 1979 | KHYTPSF | 1.009 |
| 1977 | **P** | 1974 | 1980 | HYTPSFK | 1.017 |
| 1978 | **S** | 1975 | 1981 | YTPSFKK | 1.027 |
| 1979 | **F** | 1976 | 1982 | TPSFKKG | 1.031 |
| 1980 | **K** | 1977 | 1983 | PSFKKGA | 1.042 |
| 1981 | **K** | 1978 | 1984 | SFKKGAK | 1.049 |
| 1982 | **G** | 1979 | 1985 | FKKGAKL | 1.046 |
| 1983 | **A** | 1980 | 1986 | KKGAKLL | 1.036 |
| 1984 | **K** | 1981 | 1987 | KGAKLLH | 1.013 |
| 1985 | **L** | 1982 | 1988 | GAKLLHK | 0.993 |
| 1986 | **L** | 1983 | 1989 | AKLLHKP | 0.989 |
| 1987 | **H** | 1984 | 1990 | KLLHKPI | 0.991 |
| 1988 | **K** | 1985 | 1991 | LLHKPIV | 0.996 |
| 1989 | **P** | 1986 | 1992 | LHKPIVW | 0.982 |
| 1990 | **I** | 1987 | 1993 | HKPIVWH | 0.949 |
| 1991 | **V** | 1988 | 1994 | KPIVWHV | 0.919 |
| 1992 | **W** | 1989 | 1995 | PIVWHVN | 0.898 |
| 1993 | **H** | 1990 | 1996 | IVWHVNN | 0.906 |
| 1994 | **V** | 1991 | 1997 | VWHVNNA | 0.936 |
| 1995 | **N** | 1992 | 1998 | WHVNNAT | 0.972 |
| 1996 | **N** | 1993 | 1999 | HVNNATN | 1.008 |
| 1997 | **A** | 1994 | 2000 | VNNATNK | 1.037 |
| 1998 | **T** | 1995 | 2001 | NNATNKA | 1.056 |
| 1999 | **N** | 1996 | 2002 | NATNKAT | 1.061 |
| 2000 | **K** | 1997 | 2003 | ATNKATY | 1.048 |
| 2001 | **A** | 1998 | 2004 | TNKATYK | 1.031 |
| 2002 | **T** | 1999 | 2005 | NKATYKP | 1.015 |
| 2003 | **Y** | 2000 | 2006 | KATYKPN | 1.018 |
| 2004 | **K** | 2001 | 2007 | ATYKPNT | 1.036 |
| 2005 | **P** | 2002 | 2008 | TYKPNTW | 1.043 |
| 2006 | **N** | 2003 | 2009 | YKPNTWC | 1.042 |
| 2007 | **T** | 2004 | 2010 | KPNTWCI | 1.015 |
| 2008 | **W** | 2005 | 2011 | PNTWCIR | 0.973 |
| 2009 | **C** | 2006 | 2012 | NTWCIRC | 0.939 |
| 2010 | **I** | 2007 | 2013 | TWCIRCL | 0.913 |
| 2011 | **R** | 2008 | 2014 | WCIRCLW | 0.903 |
| 2012 | **C** | 2009 | 2015 | CIRCLWS | 0.911 |
| 2013 | **L** | 2010 | 2016 | IRCLWST | 0.933 |
| 2014 | **W** | 2011 | 2017 | RCLWSTK | 0.967 |
| 2015 | **S** | 2012 | 2018 | CLWSTKP | 1.01 |
| 2016 | **T** | 2013 | 2019 | LWSTKPV | 1.039 |
| 2017 | **K** | 2014 | 2020 | WSTKPVE | 1.054 |
| 2018 | **P** | 2015 | 2021 | STKPVET | 1.056 |
| 2019 | **V** | 2016 | 2022 | TKPVETS | 1.054 |
| 2020 | **E** | 2017 | 2023 | KPVETSN | 1.064 |
| 2021 | **T** | 2018 | 2024 | PVETSNS | 1.077 |
| 2022 | **S** | 2019 | 2025 | VETSNSF | 1.083 |
| 2023 | **N** | 2020 | 2026 | ETSNSFD | 1.068 |
| 2024 | **S** | 2021 | 2027 | TSNSFDV | 1.033 |
| 2025 | **F** | 2022 | 2028 | SNSFDVL | 0.992 |
| 2026 | **D** | 2023 | 2029 | NSFDVLK | 0.97 |
| 2027 | **V** | 2024 | 2030 | SFDVLKS | 0.977 |
| 2028 | **L** | 2025 | 2031 | FDVLKSE | 1.006 |
| 2029 | **K** | 2026 | 2032 | DVLKSED | 1.049 |
| 2030 | **S** | 2027 | 2033 | VLKSEDA | 1.08 |
| 2031 | **E** | 2028 | 2034 | LKSEDAQ | 1.087 |
| 2032 | **D** | 2029 | 2035 | KSEDAQG | 1.08 |
| 2033 | **A** | 2030 | 2036 | SEDAQGM | 1.056 |
| 2034 | **Q** | 2031 | 2037 | EDAQGMD | 1.038 |
| 2035 | **G** | 2032 | 2038 | DAQGMDN | 1.028 |
| 2036 | **M** | 2033 | 2039 | AQGMDNL | 1.016 |
| 2037 | **D** | 2034 | 2040 | QGMDNLA | 1.009 |
| 2038 | **N** | 2035 | 2041 | GMDNLAC | 0.986 |
| 2039 | **L** | 2036 | 2042 | MDNLACE | 0.966 |
| 2040 | **A** | 2037 | 2043 | DNLACED | 0.959 |
| 2041 | **C** | 2038 | 2044 | NLACEDL | 0.961 |
| 2042 | **E** | 2039 | 2045 | LACEDLK | 0.988 |
| 2043 | **D** | 2040 | 2046 | ACEDLKP | 1.016 |
| 2044 | **L** | 2041 | 2047 | CEDLKPV | 1.03 |
| 2045 | **K** | 2042 | 2048 | EDLKPVS | 1.044 |
| 2046 | **P** | 2043 | 2049 | DLKPVSE | 1.047 |
| 2047 | **V** | 2044 | 2050 | LKPVSEE | 1.043 |
| 2048 | **S** | 2045 | 2051 | KPVSEEV | 1.042 |
| 2049 | **E** | 2046 | 2052 | PVSEEVV | 1.029 |
| 2050 | **E** | 2047 | 2053 | VSEEVVE | 1.011 |
| 2051 | **V** | 2048 | 2054 | SEEVVEN | 1.001 |
| 2052 | **V** | 2049 | 2055 | EEVVENP | 1.003 |
| 2053 | **E** | 2050 | 2056 | EVVENPT | 1.02 |
| 2054 | **N** | 2051 | 2057 | VVENPTI | 1.039 |
| 2055 | **P** | 2052 | 2058 | VENPTIQ | 1.047 |
| 2056 | **T** | 2053 | 2059 | ENPTIQK | 1.05 |
| 2057 | **I** | 2054 | 2060 | NPTIQKD | 1.047 |
| 2058 | **Q** | 2055 | 2061 | PTIQKDV | 1.041 |
| 2059 | **K** | 2056 | 2062 | TIQKDVL | 1.037 |
| 2060 | **D** | 2057 | 2063 | IQKDVLE | 1.021 |
| 2061 | **V** | 2058 | 2064 | QKDVLEC | 0.994 |
| 2062 | **L** | 2059 | 2065 | KDVLECN | 0.972 |
| 2063 | **E** | 2060 | 2066 | DVLECNV | 0.955 |
| 2064 | **C** | 2061 | 2067 | VLECNVK | 0.958 |
| 2065 | **N** | 2062 | 2068 | LECNVKT | 0.976 |
| 2066 | **V** | 2063 | 2069 | ECNVKTT | 1.002 |
| 2067 | **K** | 2064 | 2070 | CNVKTTE | 1.031 |
| 2068 | **T** | 2065 | 2071 | NVKTTEV | 1.041 |
| 2069 | **T** | 2066 | 2072 | VKTTEVV | 1.034 |
| 2070 | **E** | 2067 | 2073 | KTTEVVG | 1.017 |
| 2071 | **V** | 2068 | 2074 | TTEVVGD | 0.999 |
| 2072 | **V** | 2069 | 2075 | TEVVGDI | 0.989 |
| 2073 | **G** | 2070 | 2076 | EVVGDII | 0.985 |
| 2074 | **D** | 2071 | 2077 | VVGDIIL | 0.978 |
| 2075 | **I** | 2072 | 2078 | VGDIILK | 0.967 |
| 2076 | **I** | 2073 | 2079 | GDIILKP | 0.97 |
| 2077 | **L** | 2074 | 2080 | DIILKPA | 0.987 |
| 2078 | **K** | 2075 | 2081 | IILKPAN | 1.012 |
| 2079 | **P** | 2076 | 2082 | ILKPANN | 1.04 |
| 2080 | **A** | 2077 | 2083 | LKPANNS | 1.053 |
| 2081 | **N** | 2078 | 2084 | KPANNSL | 1.051 |
| 2082 | **N** | 2079 | 2085 | PANNSLK | 1.049 |
| 2083 | **S** | 2080 | 2086 | ANNSLKI | 1.038 |
| 2084 | **L** | 2081 | 2087 | NNSLKIT | 1.03 |
| 2085 | **K** | 2082 | 2088 | NSLKITE | 1.034 |
| 2086 | **I** | 2083 | 2089 | SLKITEE | 1.034 |
| 2087 | **T** | 2084 | 2090 | LKITEEV | 1.039 |
| 2088 | **E** | 2085 | 2091 | KITEEVG | 1.037 |
| 2089 | **E** | 2086 | 2092 | ITEEVGH | 1.019 |
| 2090 | **V** | 2087 | 2093 | TEEVGHT | 1.004 |
| 2091 | **G** | 2088 | 2094 | EEVGHTD | 0.996 |
| 2092 | **H** | 2089 | 2095 | EVGHTDL | 0.999 |
| 2093 | **T** | 2090 | 2096 | VGHTDLM | 0.998 |
| 2094 | **D** | 2091 | 2097 | GHTDLMA | 0.986 |
| 2095 | **L** | 2092 | 2098 | HTDLMAA | 0.955 |
| 2096 | **M** | 2093 | 2099 | TDLMAAY | 0.914 |
| 2097 | **A** | 2094 | 2100 | DLMAAYV | 0.892 |
| 2098 | **A** | 2095 | 2101 | LMAAYVD | 0.892 |
| 2099 | **Y** | 2096 | 2102 | MAAYVDN | 0.918 |
| 2100 | **V** | 2097 | 2103 | AAYVDNS | 0.973 |
| 2101 | **D** | 2098 | 2104 | AYVDNSS | 1.027 |
| 2102 | **N** | 2099 | 2105 | YVDNSSL | 1.062 |
| 2103 | **S** | 2100 | 2106 | VDNSSLT | 1.072 |
| 2104 | **S** | 2101 | 2107 | DNSSLTI | 1.05 |
| 2105 | **L** | 2102 | 2108 | NSSLTIK | 1.022 |
| 2106 | **T** | 2103 | 2109 | SSLTIKK | 1.01 |
| 2107 | **I** | 2104 | 2110 | SLTIKKP | 1.017 |
| 2108 | **K** | 2105 | 2111 | LTIKKPN | 1.042 |
| 2109 | **K** | 2106 | 2112 | TIKKPNE | 1.062 |
| 2110 | **P** | 2107 | 2113 | IKKPNEL | 1.066 |
| 2111 | **N** | 2108 | 2114 | KKPNELS | 1.062 |
| 2112 | **E** | 2109 | 2115 | KPNELSR | 1.045 |
| 2113 | **L** | 2110 | 2116 | PNELSRV | 1.024 |
| 2114 | **S** | 2111 | 2117 | NELSRVL | 1.011 |
| 2115 | **R** | 2112 | 2118 | ELSRVLG | 0.991 |
| 2116 | **V** | 2113 | 2119 | LSRVLGL | 0.972 |
| 2117 | **L** | 2114 | 2120 | SRVLGLK | 0.97 |
| 2118 | **G** | 2115 | 2121 | RVLGLKT | 0.973 |
| 2119 | **L** | 2116 | 2122 | VLGLKTL | 0.987 |
| 2120 | **K** | 2117 | 2123 | LGLKTLA | 1.004 |
| 2121 | **T** | 2118 | 2124 | GLKTLAT | 1.001 |
| 2122 | **L** | 2119 | 2125 | LKTLATH | 0.989 |
| 2123 | **A** | 2120 | 2126 | KTLATHG | 0.971 |
| 2124 | **T** | 2121 | 2127 | TLATHGL | 0.956 |
| 2125 | **H** | 2122 | 2128 | LATHGLA | 0.948 |
| 2126 | **G** | 2123 | 2129 | ATHGLAA | 0.938 |
| 2127 | **L** | 2124 | 2130 | THGLAAV | 0.931 |
| 2128 | **A** | 2125 | 2131 | HGLAAVN | 0.926 |
| 2129 | **A** | 2126 | 2132 | GLAAVNS | 0.933 |
| 2130 | **V** | 2127 | 2133 | LAAVNSV | 0.952 |
| 2131 | **N** | 2128 | 2134 | AAVNSVP | 0.97 |
| 2132 | **S** | 2129 | 2135 | AVNSVPW | 0.981 |
| 2133 | **V** | 2130 | 2136 | VNSVPWD | 0.984 |
| 2134 | **P** | 2131 | 2137 | NSVPWDT | 0.986 |
| 2135 | **W** | 2132 | 2138 | SVPWDTI | 0.986 |
| 2136 | **D** | 2133 | 2139 | VPWDTIA | 0.991 |
| 2137 | **T** | 2134 | 2140 | PWDTIAN | 0.984 |
| 2138 | **I** | 2135 | 2141 | WDTIANY | 0.966 |
| 2139 | **A** | 2136 | 2142 | DTIANYA | 0.953 |
| 2140 | **N** | 2137 | 2143 | TIANYAK | 0.947 |
| 2141 | **Y** | 2138 | 2144 | IANYAKP | 0.961 |
| 2142 | **A** | 2139 | 2145 | ANYAKPF | 0.982 |
| 2143 | **K** | 2140 | 2146 | NYAKPFL | 1.0 |
| 2144 | **P** | 2141 | 2147 | YAKPFLN | 1.005 |
| 2145 | **F** | 2142 | 2148 | AKPFLNK | 0.999 |
| 2146 | **L** | 2143 | 2149 | KPFLNKV | 0.996 |
| 2147 | **N** | 2144 | 2150 | PFLNKVV | 0.99 |
| 2148 | **K** | 2145 | 2151 | FLNKVVS | 0.991 |
| 2149 | **V** | 2146 | 2152 | LNKVVST | 0.992 |
| 2150 | **V** | 2147 | 2153 | NKVVSTT | 1.001 |
| 2151 | **S** | 2148 | 2154 | KVVSTTT | 1.022 |
| 2152 | **T** | 2149 | 2155 | VVSTTTN | 1.037 |
| 2153 | **T** | 2150 | 2156 | VSTTTNI | 1.041 |
| 2154 | **T** | 2151 | 2157 | STTTNIV | 1.027 |
| 2155 | **N** | 2152 | 2158 | TTTNIVT | 1.003 |
| 2156 | **I** | 2153 | 2159 | TTNIVTR | 0.982 |
| 2157 | **V** | 2154 | 2160 | TNIVTRC | 0.972 |
| 2158 | **T** | 2155 | 2161 | NIVTRCL | 0.973 |
| 2159 | **R** | 2156 | 2162 | IVTRCLN | 0.973 |
| 2160 | **C** | 2157 | 2163 | VTRCLNR | 0.972 |
| 2161 | **L** | 2158 | 2164 | TRCLNRV | 0.974 |
| 2162 | **N** | 2159 | 2165 | RCLNRVC | 0.969 |
| 2163 | **R** | 2160 | 2166 | CLNRVCT | 0.97 |
| 2164 | **V** | 2161 | 2167 | LNRVCTN | 0.969 |
| 2165 | **C** | 2162 | 2168 | NRVCTNY | 0.966 |
| 2166 | **T** | 2163 | 2169 | RVCTNYM | 0.966 |
| 2167 | **N** | 2164 | 2170 | VCTNYMP | 0.957 |
| 2168 | **Y** | 2165 | 2171 | CTNYMPY | 0.944 |
| 2169 | **M** | 2166 | 2172 | TNYMPYF | 0.929 |
| 2170 | **P** | 2167 | 2173 | NYMPYFF | 0.919 |
| 2171 | **Y** | 2168 | 2174 | YMPYFFT | 0.917 |
| 2172 | **F** | 2169 | 2175 | MPYFFTL | 0.921 |
| 2173 | **F** | 2170 | 2176 | PYFFTLL | 0.927 |
| 2174 | **T** | 2171 | 2177 | YFFTLLL | 0.932 |
| 2175 | **L** | 2172 | 2178 | FFTLLLQ | 0.935 |
| 2176 | **L** | 2173 | 2179 | FTLLLQL | 0.935 |
| 2177 | **L** | 2174 | 2180 | TLLLQLC | 0.932 |
| 2178 | **Q** | 2175 | 2181 | LLLQLCT | 0.925 |
| 2179 | **L** | 2176 | 2182 | LLQLCTF | 0.923 |
| 2180 | **C** | 2177 | 2183 | LQLCTFT | 0.927 |
| 2181 | **T** | 2178 | 2184 | QLCTFTR | 0.944 |
| 2182 | **F** | 2179 | 2185 | LCTFTRS | 0.977 |
| 2183 | **T** | 2180 | 2186 | CTFTRST | 1.017 |
| 2184 | **R** | 2181 | 2187 | TFTRSTN | 1.054 |
| 2185 | **S** | 2182 | 2188 | FTRSTNS | 1.09 |
| 2186 | **T** | 2183 | 2189 | TRSTNSR | 1.103 |
| 2187 | **N** | 2184 | 2190 | RSTNSRI | 1.102 |
| 2188 | **S** | 2185 | 2191 | STNSRIK | 1.093 |
| 2189 | **R** | 2186 | 2192 | TNSRIKA | 1.068 |
| 2190 | **I** | 2187 | 2193 | NSRIKAS | 1.045 |
| 2191 | **K** | 2188 | 2194 | SRIKASM | 1.023 |
| 2192 | **A** | 2189 | 2195 | RIKASMP | 1.007 |
| 2193 | **S** | 2190 | 2196 | IKASMPT | 1.001 |
| 2194 | **M** | 2191 | 2197 | KASMPTT | 1.009 |
| 2195 | **P** | 2192 | 2198 | ASMPTTI | 1.018 |
| 2196 | **T** | 2193 | 2199 | SMPTTIA | 1.015 |
| 2197 | **T** | 2194 | 2200 | MPTTIAK | 1.012 |
| 2198 | **I** | 2195 | 2201 | PTTIAKN | 1.004 |
| 2199 | **A** | 2196 | 2202 | TTIAKNT | 1.01 |
| 2200 | **K** | 2197 | 2203 | TIAKNTV | 1.027 |
| 2201 | **N** | 2198 | 2204 | IAKNTVK | 1.044 |
| 2202 | **T** | 2199 | 2205 | AKNTVKS | 1.051 |
| 2203 | **V** | 2200 | 2206 | KNTVKSV | 1.045 |
| 2204 | **K** | 2201 | 2207 | NTVKSVG | 1.04 |
| 2205 | **S** | 2202 | 2208 | TVKSVGK | 1.035 |
| 2206 | **V** | 2203 | 2209 | VKSVGKF | 1.026 |
| 2207 | **G** | 2204 | 2210 | KSVGKFC | 1.018 |
| 2208 | **K** | 2205 | 2211 | SVGKFCL | 1.001 |
| 2209 | **F** | 2206 | 2212 | VGKFCLE | 0.974 |
| 2210 | **C** | 2207 | 2213 | GKFCLEA | 0.964 |
| 2211 | **L** | 2208 | 2214 | KFCLEAS | 0.958 |
| 2212 | **E** | 2209 | 2215 | FCLEASF | 0.955 |
| 2213 | **A** | 2210 | 2216 | CLEASFN | 0.961 |
| 2214 | **S** | 2211 | 2217 | LEASFNY | 0.951 |
| 2215 | **F** | 2212 | 2218 | EASFNYL | 0.945 |
| 2216 | **N** | 2213 | 2219 | ASFNYLK | 0.949 |
| 2217 | **Y** | 2214 | 2220 | SFNYLKS | 0.969 |
| 2218 | **L** | 2215 | 2221 | FNYLKSP | 1.004 |
| 2219 | **K** | 2216 | 2222 | NYLKSPN | 1.039 |
| 2220 | **S** | 2217 | 2223 | YLKSPNF | 1.055 |
| 2221 | **P** | 2218 | 2224 | LKSPNFS | 1.049 |
| 2222 | **N** | 2219 | 2225 | KSPNFSK | 1.036 |
| 2223 | **F** | 2220 | 2226 | SPNFSKL | 1.018 |
| 2224 | **S** | 2221 | 2227 | PNFSKLI | 1.007 |
| 2225 | **K** | 2222 | 2228 | NFSKLIN | 0.996 |
| 2226 | **L** | 2223 | 2229 | FSKLINI | 0.971 |
| 2227 | **I** | 2224 | 2230 | SKLINII | 0.944 |
| 2228 | **N** | 2225 | 2231 | KLINIII | 0.918 |
| 2229 | **I** | 2226 | 2232 | LINIIIW | 0.892 |
| 2230 | **I** | 2227 | 2233 | INIIIWF | 0.878 |
| 2231 | **I** | 2228 | 2234 | NIIIWFL | 0.873 |
| 2232 | **W** | 2229 | 2235 | IIIWFLL | 0.873 |
| 2233 | **F** | 2230 | 2236 | IIWFLLL | 0.89 |
| 2234 | **L** | 2231 | 2237 | IWFLLLS | 0.907 |
| 2235 | **L** | 2232 | 2238 | WFLLLSV | 0.921 |
| 2236 | **L** | 2233 | 2239 | FLLLSVC | 0.932 |
| 2237 | **S** | 2234 | 2240 | LLLSVCL | 0.933 |
| 2238 | **V** | 2235 | 2241 | LLSVCLG | 0.941 |
| 2239 | **C** | 2236 | 2242 | LSVCLGS | 0.956 |
| 2240 | **L** | 2237 | 2243 | SVCLGSL | 0.974 |
| 2241 | **G** | 2238 | 2244 | VCLGSLI | 0.985 |
| 2242 | **S** | 2239 | 2245 | CLGSLIY | 0.983 |
| 2243 | **L** | 2240 | 2246 | LGSLIYS | 0.972 |
| 2244 | **I** | 2241 | 2247 | GSLIYST | 0.965 |
| 2245 | **Y** | 2242 | 2248 | SLIYSTA | 0.969 |
| 2246 | **S** | 2243 | 2249 | LIYSTAA | 0.976 |
| 2247 | **T** | 2244 | 2250 | IYSTAAL | 0.979 |
| 2248 | **A** | 2245 | 2251 | YSTAALG | 0.968 |
| 2249 | **A** | 2246 | 2252 | STAALGV | 0.951 |
| 2250 | **L** | 2247 | 2253 | TAALGVL | 0.938 |
| 2251 | **G** | 2248 | 2254 | AALGVLM | 0.924 |
| 2252 | **V** | 2249 | 2255 | ALGVLMS | 0.927 |
| 2253 | **L** | 2250 | 2256 | LGVLMSN | 0.935 |
| 2254 | **M** | 2251 | 2257 | GVLMSNL | 0.944 |
| 2255 | **S** | 2252 | 2258 | VLMSNLG | 0.964 |
| 2256 | **N** | 2253 | 2259 | LMSNLGM | 0.969 |
| 2257 | **L** | 2254 | 2260 | MSNLGMP | 0.974 |
| 2258 | **G** | 2255 | 2261 | SNLGMPS | 0.982 |
| 2259 | **M** | 2256 | 2262 | NLGMPSY | 0.986 |
| 2260 | **P** | 2257 | 2263 | LGMPSYC | 0.992 |
| 2261 | **S** | 2258 | 2264 | GMPSYCT | 0.991 |
| 2262 | **Y** | 2259 | 2265 | MPSYCTG | 0.985 |
| 2263 | **C** | 2260 | 2266 | PSYCTGY | 0.98 |
| 2264 | **T** | 2261 | 2267 | SYCTGYR | 0.989 |
| 2265 | **G** | 2262 | 2268 | YCTGYRE | 1.003 |
| 2266 | **Y** | 2263 | 2269 | CTGYREG | 1.016 |
| 2267 | **R** | 2264 | 2270 | TGYREGY | 1.027 |
| 2268 | **E** | 2265 | 2271 | GYREGYL | 1.023 |
| 2269 | **G** | 2266 | 2272 | YREGYLN | 1.012 |
| 2270 | **Y** | 2267 | 2273 | REGYLNS | 1.008 |
| 2271 | **L** | 2268 | 2274 | EGYLNST | 1.015 |
| 2272 | **N** | 2269 | 2275 | GYLNSTN | 1.029 |
| 2273 | **S** | 2270 | 2276 | YLNSTNV | 1.047 |
| 2274 | **T** | 2271 | 2277 | LNSTNVT | 1.043 |
| 2275 | **N** | 2272 | 2278 | NSTNVTI | 1.018 |
| 2276 | **V** | 2273 | 2279 | STNVTIA | 0.987 |
| 2277 | **T** | 2274 | 2280 | TNVTIAT | 0.954 |
| 2278 | **I** | 2275 | 2281 | NVTIATY | 0.936 |
| 2279 | **A** | 2276 | 2282 | VTIATYC | 0.928 |
| 2280 | **T** | 2277 | 2283 | TIATYCT | 0.931 |
| 2281 | **Y** | 2278 | 2284 | IATYCTG | 0.949 |
| 2282 | **C** | 2279 | 2285 | ATYCTGS | 0.975 |
| 2283 | **T** | 2280 | 2286 | TYCTGSI | 1.01 |
| 2284 | **G** | 2281 | 2287 | YCTGSIP | 1.03 |
| 2285 | **S** | 2282 | 2288 | CTGSIPC | 1.027 |
| 2286 | **I** | 2283 | 2289 | TGSIPCS | 1.008 |
| 2287 | **P** | 2284 | 2290 | GSIPCSV | 0.977 |
| 2288 | **C** | 2285 | 2291 | SIPCSVC | 0.952 |
| 2289 | **S** | 2286 | 2292 | IPCSVCL | 0.939 |
| 2290 | **V** | 2287 | 2293 | PCSVCLS | 0.942 |
| 2291 | **C** | 2288 | 2294 | CSVCLSG | 0.957 |
| 2292 | **L** | 2289 | 2295 | SVCLSGL | 0.974 |
| 2293 | **S** | 2290 | 2296 | VCLSGLD | 1.0 |
| 2294 | **G** | 2291 | 2297 | CLSGLDS | 1.018 |
| 2295 | **L** | 2292 | 2298 | LSGLDSL | 1.024 |
| 2296 | **D** | 2293 | 2299 | SGLDSLD | 1.035 |
| 2297 | **S** | 2294 | 2300 | GLDSLDT | 1.035 |
| 2298 | **L** | 2295 | 2301 | LDSLDTY | 1.03 |
| 2299 | **D** | 2296 | 2302 | DSLDTYP | 1.038 |
| 2300 | **T** | 2297 | 2303 | SLDTYPS | 1.035 |
| 2301 | **Y** | 2298 | 2304 | LDTYPSL | 1.029 |
| 2302 | **P** | 2299 | 2305 | DTYPSLE | 1.033 |
| 2303 | **S** | 2300 | 2306 | TYPSLET | 1.028 |
| 2304 | **L** | 2301 | 2307 | YPSLETI | 1.022 |
| 2305 | **E** | 2302 | 2308 | PSLETIQ | 1.017 |
| 2306 | **T** | 2303 | 2309 | SLETIQI | 1.004 |
| 2307 | **I** | 2304 | 2310 | LETIQIT | 0.987 |
| 2308 | **Q** | 2305 | 2311 | ETIQITI | 0.973 |
| 2309 | **I** | 2306 | 2312 | TIQITIS | 0.973 |
| 2310 | **T** | 2307 | 2313 | IQITISS | 0.979 |
| 2311 | **I** | 2308 | 2314 | QITISSF | 0.992 |
| 2312 | **S** | 2309 | 2315 | ITISSFK | 1.008 |
| 2313 | **S** | 2310 | 2316 | TISSFKW | 1.006 |
| 2314 | **F** | 2311 | 2317 | ISSFKWD | 0.995 |
| 2315 | **K** | 2312 | 2318 | SSFKWDL | 0.986 |
| 2316 | **W** | 2313 | 2319 | SFKWDLT | 0.965 |
| 2317 | **D** | 2314 | 2320 | FKWDLTA | 0.954 |
| 2318 | **L** | 2315 | 2321 | KWDLTAF | 0.949 |
| 2319 | **T** | 2316 | 2322 | WDLTAFG | 0.938 |
| 2320 | **A** | 2317 | 2323 | DLTAFGL | 0.937 |
| 2321 | **F** | 2318 | 2324 | LTAFGLV | 0.933 |
| 2322 | **G** | 2319 | 2325 | TAFGLVA | 0.932 |
| 2323 | **L** | 2320 | 2326 | AFGLVAE | 0.934 |
| 2324 | **V** | 2321 | 2327 | FGLVAEW | 0.932 |
| 2325 | **A** | 2322 | 2328 | GLVAEWF | 0.932 |
| 2326 | **E** | 2323 | 2329 | LVAEWFL | 0.929 |
| 2327 | **W** | 2324 | 2330 | VAEWFLA | 0.922 |
| 2328 | **F** | 2325 | 2331 | AEWFLAY | 0.913 |
| 2329 | **L** | 2326 | 2332 | EWFLAYI | 0.901 |
| 2330 | **A** | 2327 | 2333 | WFLAYIL | 0.891 |
| 2331 | **Y** | 2328 | 2334 | FLAYILF | 0.884 |
| 2332 | **I** | 2329 | 2335 | LAYILFT | 0.896 |
| 2333 | **L** | 2330 | 2336 | AYILFTR | 0.921 |
| 2334 | **F** | 2331 | 2337 | YILFTRF | 0.945 |
| 2335 | **T** | 2332 | 2338 | ILFTRFF | 0.967 |
| 2336 | **R** | 2333 | 2339 | LFTRFFY | 0.963 |
| 2337 | **F** | 2334 | 2340 | FTRFFYV | 0.942 |
| 2338 | **F** | 2335 | 2341 | TRFFYVL | 0.925 |
| 2339 | **Y** | 2336 | 2342 | RFFYVLG | 0.908 |
| 2340 | **V** | 2337 | 2343 | FFYVLGL | 0.912 |
| 2341 | **L** | 2338 | 2344 | FYVLGLA | 0.922 |
| 2342 | **G** | 2339 | 2345 | YVLGLAA | 0.925 |
| 2343 | **L** | 2340 | 2346 | VLGLAAI | 0.924 |
| 2344 | **A** | 2341 | 2347 | LGLAAIM | 0.909 |
| 2345 | **A** | 2342 | 2348 | GLAAIMQ | 0.895 |
| 2346 | **I** | 2343 | 2349 | LAAIMQL | 0.889 |
| 2347 | **M** | 2344 | 2350 | AAIMQLF | 0.889 |
| 2348 | **Q** | 2345 | 2351 | AIMQLFF | 0.899 |
| 2349 | **L** | 2346 | 2352 | IMQLFFS | 0.911 |
| 2350 | **F** | 2347 | 2353 | MQLFFSY | 0.918 |
| 2351 | **F** | 2348 | 2354 | QLFFSYF | 0.921 |
| 2352 | **S** | 2349 | 2355 | LFFSYFA | 0.92 |
| 2353 | **Y** | 2350 | 2356 | FFSYFAV | 0.917 |
| 2354 | **F** | 2351 | 2357 | FSYFAVH | 0.913 |
| 2355 | **A** | 2352 | 2358 | SYFAVHF | 0.908 |
| 2356 | **V** | 2353 | 2359 | YFAVHFI | 0.905 |
| 2357 | **H** | 2354 | 2360 | FAVHFIS | 0.912 |
| 2358 | **F** | 2355 | 2361 | AVHFISN | 0.934 |
| 2359 | **I** | 2356 | 2362 | VHFISNS | 0.965 |
| 2360 | **S** | 2357 | 2363 | HFISNSW | 0.997 |
| 2361 | **N** | 2358 | 2364 | FISNSWL | 1.013 |
| 2362 | **S** | 2359 | 2365 | ISNSWLM | 0.996 |
| 2363 | **W** | 2360 | 2366 | SNSWLMW | 0.954 |
| 2364 | **L** | 2361 | 2367 | NSWLMWL | 0.912 |
| 2365 | **M** | 2362 | 2368 | SWLMWLI | 0.874 |
| 2366 | **W** | 2363 | 2369 | WLMWLII | 0.861 |
| 2367 | **L** | 2364 | 2370 | LMWLIIN | 0.872 |
| 2368 | **I** | 2365 | 2371 | MWLIINL | 0.885 |
| 2369 | **I** | 2366 | 2372 | WLIINLV | 0.904 |
| 2370 | **N** | 2367 | 2373 | LIINLVQ | 0.918 |
| 2371 | **L** | 2368 | 2374 | IINLVQM | 0.919 |
| 2372 | **V** | 2369 | 2375 | INLVQMA | 0.917 |
| 2373 | **Q** | 2370 | 2376 | NLVQMAP | 0.911 |
| 2374 | **M** | 2371 | 2377 | LVQMAPI | 0.914 |
| 2375 | **A** | 2372 | 2378 | VQMAPIS | 0.924 |
| 2376 | **P** | 2373 | 2379 | QMAPISA | 0.936 |
| 2377 | **I** | 2374 | 2380 | MAPISAM | 0.939 |
| 2378 | **S** | 2375 | 2381 | APISAMV | 0.93 |
| 2379 | **A** | 2376 | 2382 | PISAMVR | 0.916 |
| 2380 | **M** | 2377 | 2383 | ISAMVRM | 0.897 |
| 2381 | **V** | 2378 | 2384 | SAMVRMY | 0.887 |
| 2382 | **R** | 2379 | 2385 | AMVRMYI | 0.879 |
| 2383 | **M** | 2380 | 2386 | MVRMYIF | 0.874 |
| 2384 | **Y** | 2381 | 2387 | VRMYIFF | 0.876 |
| 2385 | **I** | 2382 | 2388 | RMYIFFA | 0.884 |
| 2386 | **F** | 2383 | 2389 | MYIFFAS | 0.898 |
| 2387 | **F** | 2384 | 2390 | YIFFASF | 0.911 |
| 2388 | **A** | 2385 | 2391 | IFFASFY | 0.916 |
| 2389 | **S** | 2386 | 2392 | FFASFYY | 0.907 |
| 2390 | **F** | 2387 | 2393 | FASFYYV | 0.895 |
| 2391 | **Y** | 2388 | 2394 | ASFYYVW | 0.883 |
| 2392 | **Y** | 2389 | 2395 | SFYYVWK | 0.891 |
| 2393 | **V** | 2390 | 2396 | FYYVWKS | 0.919 |
| 2394 | **W** | 2391 | 2397 | YYVWKSY | 0.949 |
| 2395 | **K** | 2392 | 2398 | YVWKSYV | 0.979 |
| 2396 | **S** | 2393 | 2399 | VWKSYVH | 0.981 |
| 2397 | **Y** | 2394 | 2400 | WKSYVHV | 0.962 |
| 2398 | **V** | 2395 | 2401 | KSYVHVV | 0.941 |
| 2399 | **H** | 2396 | 2402 | SYVHVVD | 0.932 |
| 2400 | **V** | 2397 | 2403 | YVHVVDG | 0.943 |
| 2401 | **V** | 2398 | 2404 | VHVVDGC | 0.966 |
| 2402 | **D** | 2399 | 2405 | HVVDGCN | 0.994 |
| 2403 | **G** | 2400 | 2406 | VVDGCNS | 1.016 |
| 2404 | **C** | 2401 | 2407 | VDGCNSS | 1.037 |
| 2405 | **N** | 2402 | 2408 | DGCNSST | 1.061 |
| 2406 | **S** | 2403 | 2409 | GCNSSTC | 1.071 |
| 2407 | **S** | 2404 | 2410 | CNSSTCM | 1.054 |
| 2408 | **T** | 2405 | 2411 | NSSTCMM | 1.006 |
| 2409 | **C** | 2406 | 2412 | SSTCMMC | 0.945 |
| 2410 | **M** | 2407 | 2413 | STCMMCY | 0.895 |
| 2411 | **M** | 2408 | 2414 | TCMMCYK | 0.884 |
| 2412 | **C** | 2409 | 2415 | CMMCYKR | 0.912 |
| 2413 | **Y** | 2410 | 2416 | MMCYKRN | 0.959 |
| 2414 | **K** | 2411 | 2417 | MCYKRNR | 1.009 |
| 2415 | **R** | 2412 | 2418 | CYKRNRA | 1.039 |
| 2416 | **N** | 2413 | 2419 | YKRNRAT | 1.056 |
| 2417 | **R** | 2414 | 2420 | KRNRATR | 1.055 |
| 2418 | **A** | 2415 | 2421 | RNRATRV | 1.044 |
| 2419 | **T** | 2416 | 2422 | NRATRVE | 1.03 |
| 2420 | **R** | 2417 | 2423 | RATRVEC | 1.009 |
| 2421 | **V** | 2418 | 2424 | ATRVECT | 0.995 |
| 2422 | **E** | 2419 | 2425 | TRVECTT | 0.989 |
| 2423 | **C** | 2420 | 2426 | RVECTTI | 0.986 |
| 2424 | **T** | 2421 | 2427 | VECTTIV | 0.987 |
| 2425 | **T** | 2422 | 2428 | ECTTIVN | 0.984 |
| 2426 | **I** | 2423 | 2429 | CTTIVNG | 0.976 |
| 2427 | **V** | 2424 | 2430 | TTIVNGV | 0.976 |
| 2428 | **N** | 2425 | 2431 | TIVNGVR | 0.985 |
| 2429 | **G** | 2426 | 2432 | IVNGVRR | 0.998 |
| 2430 | **V** | 2427 | 2433 | VNGVRRS | 1.013 |
| 2431 | **R** | 2428 | 2434 | NGVRRSF | 1.017 |
| 2432 | **R** | 2429 | 2435 | GVRRSFY | 1.003 |
| 2433 | **S** | 2430 | 2436 | VRRSFYV | 0.979 |
| 2434 | **F** | 2431 | 2437 | RRSFYVY | 0.942 |
| 2435 | **Y** | 2432 | 2438 | RSFYVYA | 0.911 |
| 2436 | **V** | 2433 | 2439 | SFYVYAN | 0.903 |
| 2437 | **Y** | 2434 | 2440 | FYVYANG | 0.917 |
| 2438 | **A** | 2435 | 2441 | YVYANGG | 0.963 |
| 2439 | **N** | 2436 | 2442 | VYANGGK | 1.016 |
| 2440 | **G** | 2437 | 2443 | YANGGKG | 1.061 |
| 2441 | **G** | 2438 | 2444 | ANGGKGF | 1.077 |
| 2442 | **K** | 2439 | 2445 | NGGKGFC | 1.057 |
| 2443 | **G** | 2440 | 2446 | GGKGFCK | 1.026 |
| 2444 | **F** | 2441 | 2447 | GKGFCKL | 0.988 |
| 2445 | **C** | 2442 | 2448 | KGFCKLH | 0.967 |
| 2446 | **K** | 2443 | 2449 | GFCKLHN | 0.965 |
| 2447 | **L** | 2444 | 2450 | FCKLHNW | 0.958 |
| 2448 | **H** | 2445 | 2451 | CKLHNWN | 0.953 |
| 2449 | **N** | 2446 | 2452 | KLHNWNC | 0.942 |
| 2450 | **W** | 2447 | 2453 | LHNWNCV | 0.927 |
| 2451 | **N** | 2448 | 2454 | HNWNCVN | 0.92 |
| 2452 | **C** | 2449 | 2455 | NWNCVNC | 0.918 |
| 2453 | **V** | 2450 | 2456 | WNCVNCD | 0.933 |
| 2454 | **N** | 2451 | 2457 | NCVNCDT | 0.956 |
| 2455 | **C** | 2452 | 2458 | CVNCDTF | 0.978 |
| 2456 | **D** | 2453 | 2459 | VNCDTFC | 0.995 |
| 2457 | **T** | 2454 | 2460 | NCDTFCA | 0.991 |
| 2458 | **F** | 2455 | 2461 | CDTFCAG | 0.978 |
| 2459 | **C** | 2456 | 2462 | DTFCAGS | 0.982 |
| 2460 | **A** | 2457 | 2463 | TFCAGST | 0.997 |
| 2461 | **G** | 2458 | 2464 | FCAGSTF | 1.018 |
| 2462 | **S** | 2459 | 2465 | CAGSTFI | 1.03 |
| 2463 | **T** | 2460 | 2466 | AGSTFIS | 1.019 |
| 2464 | **F** | 2461 | 2467 | GSTFISD | 0.999 |
| 2465 | **I** | 2462 | 2468 | STFISDE | 0.989 |
| 2466 | **S** | 2463 | 2469 | TFISDEV | 0.99 |
| 2467 | **D** | 2464 | 2470 | FISDEVA | 0.993 |
| 2468 | **E** | 2465 | 2471 | ISDEVAR | 0.996 |
| 2469 | **V** | 2466 | 2472 | SDEVARD | 0.995 |
| 2470 | **A** | 2467 | 2473 | DEVARDL | 0.994 |
| 2471 | **R** | 2468 | 2474 | EVARDLS | 0.998 |
| 2472 | **D** | 2469 | 2475 | VARDLSL | 0.998 |
| 2473 | **L** | 2470 | 2476 | ARDLSLQ | 0.983 |
| 2474 | **S** | 2471 | 2477 | RDLSLQF | 0.962 |
| 2475 | **L** | 2472 | 2478 | DLSLQFK | 0.954 |
| 2476 | **Q** | 2473 | 2479 | LSLQFKR | 0.953 |
| 2477 | **F** | 2474 | 2480 | SLQFKRP | 0.977 |
| 2478 | **K** | 2475 | 2481 | LQFKRPI | 1.009 |
| 2479 | **R** | 2476 | 2482 | QFKRPIN | 1.025 |
| 2480 | **P** | 2477 | 2483 | FKRPINP | 1.039 |
| 2481 | **I** | 2478 | 2484 | KRPINPT | 1.039 |
| 2482 | **N** | 2479 | 2485 | RPINPTD | 1.036 |
| 2483 | **P** | 2480 | 2486 | PINPTDQ | 1.049 |
| 2484 | **T** | 2481 | 2487 | INPTDQS | 1.067 |
| 2485 | **D** | 2482 | 2488 | NPTDQSS | 1.084 |
| 2486 | **Q** | 2483 | 2489 | PTDQSSY | 1.093 |
| 2487 | **S** | 2484 | 2490 | TDQSSYI | 1.074 |
| 2488 | **S** | 2485 | 2491 | DQSSYIV | 1.033 |
| 2489 | **Y** | 2486 | 2492 | QSSYIVD | 0.995 |
| 2490 | **I** | 2487 | 2493 | SSYIVDS | 0.972 |
| 2491 | **V** | 2488 | 2494 | SYIVDSV | 0.974 |
| 2492 | **D** | 2489 | 2495 | YIVDSVT | 0.991 |
| 2493 | **S** | 2490 | 2496 | IVDSVTV | 0.999 |
| 2494 | **V** | 2491 | 2497 | VDSVTVK | 1.002 |
| 2495 | **T** | 2492 | 2498 | DSVTVKN | 1.006 |
| 2496 | **V** | 2493 | 2499 | SVTVKNG | 1.023 |
| 2497 | **K** | 2494 | 2500 | VTVKNGS | 1.05 |
| 2498 | **N** | 2495 | 2501 | TVKNGSI | 1.064 |
| 2499 | **G** | 2496 | 2502 | VKNGSIH | 1.055 |
| 2500 | **S** | 2497 | 2503 | KNGSIHL | 1.02 |
| 2501 | **I** | 2498 | 2504 | NGSIHLY | 0.967 |
| 2502 | **H** | 2499 | 2505 | GSIHLYF | 0.927 |
| 2503 | **L** | 2500 | 2506 | SIHLYFD | 0.914 |
| 2504 | **Y** | 2501 | 2507 | IHLYFDK | 0.924 |
| 2505 | **F** | 2502 | 2508 | HLYFDKA | 0.96 |
| 2506 | **D** | 2503 | 2509 | LYFDKAG | 1.004 |
| 2507 | **K** | 2504 | 2510 | YFDKAGQ | 1.039 |
| 2508 | **A** | 2505 | 2511 | FDKAGQK | 1.066 |
| 2509 | **G** | 2506 | 2512 | DKAGQKT | 1.08 |
| 2510 | **Q** | 2507 | 2513 | KAGQKTY | 1.081 |
| 2511 | **K** | 2508 | 2514 | AGQKTYE | 1.069 |
| 2512 | **T** | 2509 | 2515 | GQKTYER | 1.052 |
| 2513 | **Y** | 2510 | 2516 | QKTYERH | 1.031 |
| 2514 | **E** | 2511 | 2517 | KTYERHS | 1.012 |
| 2515 | **R** | 2512 | 2518 | TYERHSL | 0.997 |
| 2516 | **H** | 2513 | 2519 | YERHSLS | 0.979 |
| 2517 | **S** | 2514 | 2520 | ERHSLSH | 0.964 |
| 2518 | **L** | 2515 | 2521 | RHSLSHF | 0.951 |
| 2519 | **S** | 2516 | 2522 | HSLSHFV | 0.939 |
| 2520 | **H** | 2517 | 2523 | SLSHFVN | 0.935 |
| 2521 | **F** | 2518 | 2524 | LSHFVNL | 0.933 |
| 2522 | **V** | 2519 | 2525 | SHFVNLD | 0.943 |
| 2523 | **N** | 2520 | 2526 | HFVNLDN | 0.96 |
| 2524 | **L** | 2521 | 2527 | FVNLDNL | 0.98 |
| 2525 | **D** | 2522 | 2528 | VNLDNLR | 0.994 |
| 2526 | **N** | 2523 | 2529 | NLDNLRA | 0.994 |
| 2527 | **L** | 2524 | 2530 | LDNLRAN | 0.989 |
| 2528 | **R** | 2525 | 2531 | DNLRANN | 0.991 |
| 2529 | **A** | 2526 | 2532 | NLRANNT | 1.008 |
| 2530 | **N** | 2527 | 2533 | LRANNTK | 1.032 |
| 2531 | **N** | 2528 | 2534 | RANNTKG | 1.063 |
| 2532 | **T** | 2529 | 2535 | ANNTKGS | 1.082 |
| 2533 | **K** | 2530 | 2536 | NNTKGSL | 1.083 |
| 2534 | **G** | 2531 | 2537 | NTKGSLP | 1.07 |
| 2535 | **S** | 2532 | 2538 | TKGSLPI | 1.04 |
| 2536 | **L** | 2533 | 2539 | KGSLPIN | 1.007 |
| 2537 | **P** | 2534 | 2540 | GSLPINV | 0.979 |
| 2538 | **I** | 2535 | 2541 | SLPINVI | 0.956 |
| 2539 | **N** | 2536 | 2542 | LPINVIV | 0.937 |
| 2540 | **V** | 2537 | 2543 | PINVIVF | 0.924 |
| 2541 | **I** | 2538 | 2544 | INVIVFD | 0.924 |
| 2542 | **V** | 2539 | 2545 | NVIVFDG | 0.944 |
| 2543 | **F** | 2540 | 2546 | VIVFDGK | 0.981 |
| 2544 | **D** | 2541 | 2547 | IVFDGKS | 1.036 |
| 2545 | **G** | 2542 | 2548 | VFDGKSK | 1.079 |
| 2546 | **K** | 2543 | 2549 | FDGKSKC | 1.095 |
| 2547 | **S** | 2544 | 2550 | DGKSKCE | 1.096 |
| 2548 | **K** | 2545 | 2551 | GKSKCEE | 1.076 |
| 2549 | **C** | 2546 | 2552 | KSKCEES | 1.061 |
| 2550 | **E** | 2547 | 2553 | SKCEESS | 1.064 |
| 2551 | **E** | 2548 | 2554 | KCEESSA | 1.071 |
| 2552 | **S** | 2549 | 2555 | CEESSAK | 1.081 |
| 2553 | **S** | 2550 | 2556 | EESSAKS | 1.079 |
| 2554 | **A** | 2551 | 2557 | ESSAKSA | 1.07 |
| 2555 | **K** | 2552 | 2558 | SSAKSAS | 1.054 |
| 2556 | **S** | 2553 | 2559 | SAKSASV | 1.029 |
| 2557 | **A** | 2554 | 2560 | AKSASVY | 0.998 |
| 2558 | **S** | 2555 | 2561 | KSASVYY | 0.961 |
| 2559 | **V** | 2556 | 2562 | SASVYYS | 0.939 |
| 2560 | **Y** | 2557 | 2563 | ASVYYSQ | 0.933 |
| 2561 | **Y** | 2558 | 2564 | SVYYSQL | 0.948 |
| 2562 | **S** | 2559 | 2565 | VYYSQLM | 0.962 |
| 2563 | **Q** | 2560 | 2566 | YYSQLMC | 0.958 |
| 2564 | **L** | 2561 | 2567 | YSQLMCQ | 0.947 |
| 2565 | **M** | 2562 | 2568 | SQLMCQP | 0.936 |
| 2566 | **C** | 2563 | 2569 | QLMCQPI | 0.939 |
| 2567 | **Q** | 2564 | 2570 | LMCQPIL | 0.955 |
| 2568 | **P** | 2565 | 2571 | MCQPILL | 0.964 |
| 2569 | **I** | 2566 | 2572 | CQPILLL | 0.958 |
| 2570 | **L** | 2567 | 2573 | QPILLLD | 0.958 |
| 2571 | **L** | 2568 | 2574 | PILLLDQ | 0.963 |
| 2572 | **L** | 2569 | 2575 | ILLLDQA | 0.976 |
| 2573 | **D** | 2570 | 2576 | LLLDQAL | 0.994 |
| 2574 | **Q** | 2571 | 2577 | LLDQALV | 0.989 |
| 2575 | **A** | 2572 | 2578 | LDQALVS | 0.979 |
| 2576 | **L** | 2573 | 2579 | DQALVSD | 0.977 |
| 2577 | **V** | 2574 | 2580 | QALVSDV | 0.981 |
| 2578 | **S** | 2575 | 2581 | ALVSDVG | 1.002 |
| 2579 | **D** | 2576 | 2582 | LVSDVGD | 1.021 |
| 2580 | **V** | 2577 | 2583 | VSDVGDS | 1.029 |
| 2581 | **G** | 2578 | 2584 | SDVGDSA | 1.036 |
| 2582 | **D** | 2579 | 2585 | DVGDSAE | 1.029 |
| 2583 | **S** | 2580 | 2586 | VGDSAEV | 1.017 |
| 2584 | **A** | 2581 | 2587 | GDSAEVA | 0.998 |
| 2585 | **E** | 2582 | 2588 | DSAEVAV | 0.967 |
| 2586 | **V** | 2583 | 2589 | SAEVAVK | 0.952 |
| 2587 | **A** | 2584 | 2590 | AEVAVKM | 0.938 |
| 2588 | **V** | 2585 | 2591 | EVAVKMF | 0.936 |
| 2589 | **K** | 2586 | 2592 | VAVKMFD | 0.941 |
| 2590 | **M** | 2587 | 2593 | AVKMFDA | 0.932 |
| 2591 | **F** | 2588 | 2594 | VKMFDAY | 0.925 |
| 2592 | **D** | 2589 | 2595 | KMFDAYV | 0.918 |
| 2593 | **A** | 2590 | 2596 | MFDAYVN | 0.915 |
| 2594 | **Y** | 2591 | 2597 | FDAYVNT | 0.925 |
| 2595 | **V** | 2592 | 2598 | DAYVNTF | 0.943 |
| 2596 | **N** | 2593 | 2599 | AYVNTFS | 0.968 |
| 2597 | **T** | 2594 | 2600 | YVNTFSS | 0.998 |
| 2598 | **F** | 2595 | 2601 | VNTFSST | 1.022 |
| 2599 | **S** | 2596 | 2602 | NTFSSTF | 1.039 |
| 2600 | **S** | 2597 | 2603 | TFSSTFN | 1.042 |
| 2601 | **T** | 2598 | 2604 | FSSTFNV | 1.023 |
| 2602 | **F** | 2599 | 2605 | SSTFNVP | 0.997 |
| 2603 | **N** | 2600 | 2606 | STFNVPM | 0.971 |
| 2604 | **V** | 2601 | 2607 | TFNVPME | 0.96 |
| 2605 | **P** | 2602 | 2608 | FNVPMEK | 0.966 |
| 2606 | **M** | 2603 | 2609 | NVPMEKL | 0.982 |
| 2607 | **E** | 2604 | 2610 | VPMEKLK | 1.006 |
| 2608 | **K** | 2605 | 2611 | PMEKLKT | 1.024 |
| 2609 | **L** | 2606 | 2612 | MEKLKTL | 1.028 |
| 2610 | **K** | 2607 | 2613 | EKLKTLV | 1.026 |
| 2611 | **T** | 2608 | 2614 | KLKTLVA | 1.008 |
| 2612 | **L** | 2609 | 2615 | LKTLVAT | 0.981 |
| 2613 | **V** | 2610 | 2616 | KTLVATA | 0.967 |
| 2614 | **A** | 2611 | 2617 | TLVATAE | 0.957 |
| 2615 | **T** | 2612 | 2618 | LVATAEA | 0.962 |
| 2616 | **A** | 2613 | 2619 | VATAEAE | 0.974 |
| 2617 | **E** | 2614 | 2620 | ATAEAEL | 0.976 |
| 2618 | **A** | 2615 | 2621 | TAEAELA | 0.978 |
| 2619 | **E** | 2616 | 2622 | AEAELAK | 0.976 |
| 2620 | **L** | 2617 | 2623 | EAELAKN | 0.979 |
| 2621 | **A** | 2618 | 2624 | AELAKNV | 0.988 |
| 2622 | **K** | 2619 | 2625 | ELAKNVS | 0.995 |
| 2623 | **N** | 2620 | 2626 | LAKNVSL | 0.993 |
| 2624 | **V** | 2621 | 2627 | AKNVSLD | 0.991 |
| 2625 | **S** | 2622 | 2628 | KNVSLDN | 0.989 |
| 2626 | **L** | 2623 | 2629 | NVSLDNV | 0.988 |
| 2627 | **D** | 2624 | 2630 | VSLDNVL | 0.995 |
| 2628 | **N** | 2625 | 2631 | SLDNVLS | 0.994 |
| 2629 | **V** | 2626 | 2632 | LDNVLST | 0.994 |
| 2630 | **L** | 2627 | 2633 | DNVLSTF | 0.993 |
| 2631 | **S** | 2628 | 2634 | NVLSTFI | 0.988 |
| 2632 | **T** | 2629 | 2635 | VLSTFIS | 0.978 |
| 2633 | **F** | 2630 | 2636 | LSTFISA | 0.958 |
| 2634 | **I** | 2631 | 2637 | STFISAA | 0.941 |
| 2635 | **S** | 2632 | 2638 | TFISAAR | 0.938 |
| 2636 | **A** | 2633 | 2639 | FISAARQ | 0.957 |
| 2637 | **A** | 2634 | 2640 | ISAARQG | 0.988 |
| 2638 | **R** | 2635 | 2641 | SAARQGF | 1.016 |
| 2639 | **Q** | 2636 | 2642 | AARQGFV | 1.029 |
| 2640 | **G** | 2637 | 2643 | ARQGFVD | 1.021 |
| 2641 | **F** | 2638 | 2644 | RQGFVDS | 1.016 |
| 2642 | **V** | 2639 | 2645 | QGFVDSD | 1.024 |
| 2643 | **D** | 2640 | 2646 | GFVDSDV | 1.042 |
| 2644 | **S** | 2641 | 2647 | FVDSDVE | 1.061 |
| 2645 | **D** | 2642 | 2648 | VDSDVET | 1.066 |
| 2646 | **V** | 2643 | 2649 | DSDVETK | 1.061 |
| 2647 | **E** | 2644 | 2650 | SDVETKD | 1.058 |
| 2648 | **T** | 2645 | 2651 | DVETKDV | 1.052 |
| 2649 | **K** | 2646 | 2652 | VETKDVV | 1.043 |
| 2650 | **D** | 2647 | 2653 | ETKDVVE | 1.024 |
| 2651 | **V** | 2648 | 2654 | TKDVVEC | 0.985 |
| 2652 | **V** | 2649 | 2655 | KDVVECL | 0.955 |
| 2653 | **E** | 2650 | 2656 | DVVECLK | 0.942 |
| 2654 | **C** | 2651 | 2657 | VVECLKL | 0.941 |
| 2655 | **L** | 2652 | 2658 | VECLKLS | 0.956 |
| 2656 | **K** | 2653 | 2659 | ECLKLSH | 0.971 |
| 2657 | **L** | 2654 | 2660 | CLKLSHQ | 0.975 |
| 2658 | **S** | 2655 | 2661 | LKLSHQS | 0.99 |
| 2659 | **H** | 2656 | 2662 | KLSHQSD | 1.012 |
| 2660 | **Q** | 2657 | 2663 | LSHQSDI | 1.035 |
| 2661 | **S** | 2658 | 2664 | SHQSDIE | 1.053 |
| 2662 | **D** | 2659 | 2665 | HQSDIEV | 1.047 |
| 2663 | **I** | 2660 | 2666 | QSDIEVT | 1.028 |
| 2664 | **E** | 2661 | 2667 | SDIEVTG | 1.017 |
| 2665 | **V** | 2662 | 2668 | DIEVTGD | 1.018 |
| 2666 | **T** | 2663 | 2669 | IEVTGDS | 1.035 |
| 2667 | **G** | 2664 | 2670 | EVTGDSC | 1.048 |
| 2668 | **D** | 2665 | 2671 | VTGDSCN | 1.044 |
| 2669 | **S** | 2666 | 2672 | TGDSCNN | 1.033 |
| 2670 | **C** | 2667 | 2673 | GDSCNNY | 1.01 |
| 2671 | **N** | 2668 | 2674 | DSCNNYM | 0.982 |
| 2672 | **N** | 2669 | 2675 | SCNNYML | 0.961 |
| 2673 | **Y** | 2670 | 2676 | CNNYMLT | 0.936 |
| 2674 | **M** | 2671 | 2677 | NNYMLTY | 0.921 |
| 2675 | **L** | 2672 | 2678 | NYMLTYN | 0.928 |
| 2676 | **T** | 2673 | 2679 | YMLTYNK | 0.946 |
| 2677 | **Y** | 2674 | 2680 | MLTYNKV | 0.971 |
| 2678 | **N** | 2675 | 2681 | LTYNKVE | 0.999 |
| 2679 | **K** | 2676 | 2682 | TYNKVEN | 1.014 |
| 2680 | **V** | 2677 | 2683 | YNKVENM | 1.013 |
| 2681 | **E** | 2678 | 2684 | NKVENMT | 1.014 |
| 2682 | **N** | 2679 | 2685 | KVENMTP | 1.011 |
| 2683 | **M** | 2680 | 2686 | VENMTPR | 1.01 |
| 2684 | **T** | 2681 | 2687 | ENMTPRD | 1.026 |
| 2685 | **P** | 2682 | 2688 | NMTPRDL | 1.033 |
| 2686 | **R** | 2683 | 2689 | MTPRDLG | 1.031 |
| 2687 | **D** | 2684 | 2690 | TPRDLGA | 1.02 |
| 2688 | **L** | 2685 | 2691 | PRDLGAC | 0.991 |
| 2689 | **G** | 2686 | 2692 | RDLGACI | 0.962 |
| 2690 | **A** | 2687 | 2693 | DLGACID | 0.942 |
| 2691 | **C** | 2688 | 2694 | LGACIDC | 0.928 |
| 2692 | **I** | 2689 | 2695 | GACIDCS | 0.925 |
| 2693 | **D** | 2690 | 2696 | ACIDCSA | 0.935 |
| 2694 | **C** | 2691 | 2697 | CIDCSAR | 0.944 |
| 2695 | **S** | 2692 | 2698 | IDCSARH | 0.95 |
| 2696 | **A** | 2693 | 2699 | DCSARHI | 0.955 |
| 2697 | **R** | 2694 | 2700 | CSARHIN | 0.944 |
| 2698 | **H** | 2695 | 2701 | SARHINA | 0.943 |
| 2699 | **I** | 2696 | 2702 | ARHINAQ | 0.939 |
| 2700 | **N** | 2697 | 2703 | RHINAQV | 0.939 |
| 2701 | **A** | 2698 | 2704 | HINAQVA | 0.947 |
| 2702 | **Q** | 2699 | 2705 | INAQVAK | 0.948 |
| 2703 | **V** | 2700 | 2706 | NAQVAKS | 0.964 |
| 2704 | **A** | 2701 | 2707 | AQVAKSH | 0.981 |
| 2705 | **K** | 2702 | 2708 | QVAKSHN | 0.996 |
| 2706 | **S** | 2703 | 2709 | VAKSHNI | 0.997 |
| 2707 | **H** | 2704 | 2710 | AKSHNIA | 0.978 |
| 2708 | **N** | 2705 | 2711 | KSHNIAL | 0.952 |
| 2709 | **I** | 2706 | 2712 | SHNIALI | 0.922 |
| 2710 | **A** | 2707 | 2713 | HNIALIW | 0.907 |
| 2711 | **L** | 2708 | 2714 | NIALIWN | 0.903 |
| 2712 | **I** | 2709 | 2715 | IALIWNV | 0.907 |
| 2713 | **W** | 2710 | 2716 | ALIWNVK | 0.928 |
| 2714 | **N** | 2711 | 2717 | LIWNVKD | 0.958 |
| 2715 | **V** | 2712 | 2718 | IWNVKDF | 0.985 |
| 2716 | **K** | 2713 | 2719 | WNVKDFM | 1.0 |
| 2717 | **D** | 2714 | 2720 | NVKDFMS | 0.992 |
| 2718 | **F** | 2715 | 2721 | VKDFMSL | 0.966 |
| 2719 | **M** | 2716 | 2722 | KDFMSLS | 0.95 |
| 2720 | **S** | 2717 | 2723 | DFMSLSE | 0.955 |
| 2721 | **L** | 2718 | 2724 | FMSLSEQ | 0.977 |
| 2722 | **S** | 2719 | 2725 | MSLSEQL | 1.007 |
| 2723 | **E** | 2720 | 2726 | SLSEQLR | 1.026 |
| 2724 | **Q** | 2721 | 2727 | LSEQLRK | 1.032 |
| 2725 | **L** | 2722 | 2728 | SEQLRKQ | 1.03 |
| 2726 | **R** | 2723 | 2729 | EQLRKQI | 1.031 |
| 2727 | **K** | 2724 | 2730 | QLRKQIR | 1.033 |
| 2728 | **Q** | 2725 | 2731 | LRKQIRS | 1.033 |
| 2729 | **I** | 2726 | 2732 | RKQIRSA | 1.027 |
| 2730 | **R** | 2727 | 2733 | KQIRSAA | 1.016 |
| 2731 | **S** | 2728 | 2734 | QIRSAAK | 1.008 |
| 2732 | **A** | 2729 | 2735 | IRSAAKK | 1.005 |
| 2733 | **A** | 2730 | 2736 | RSAAKKN | 1.018 |
| 2734 | **K** | 2731 | 2737 | SAAKKNN | 1.039 |
| 2735 | **K** | 2732 | 2738 | AAKKNNL | 1.049 |
| 2736 | **N** | 2733 | 2739 | AKKNNLP | 1.046 |
| 2737 | **N** | 2734 | 2740 | KKNNLPF | 1.021 |
| 2738 | **L** | 2735 | 2741 | KNNLPFK | 0.995 |
| 2739 | **P** | 2736 | 2742 | NNLPFKL | 0.977 |
| 2740 | **F** | 2737 | 2743 | NLPFKLT | 0.968 |
| 2741 | **K** | 2738 | 2744 | LPFKLTC | 0.969 |
| 2742 | **L** | 2739 | 2745 | PFKLTCA | 0.958 |
| 2743 | **T** | 2740 | 2746 | FKLTCAT | 0.954 |
| 2744 | **C** | 2741 | 2747 | KLTCATT | 0.957 |
| 2745 | **A** | 2742 | 2748 | LTCATTR | 0.974 |
| 2746 | **T** | 2743 | 2749 | TCATTRQ | 1.003 |
| 2747 | **T** | 2744 | 2750 | CATTRQV | 1.02 |
| 2748 | **R** | 2745 | 2751 | ATTRQVV | 1.018 |
| 2749 | **Q** | 2746 | 2752 | TTRQVVN | 0.999 |
| 2750 | **V** | 2747 | 2753 | TRQVVNV | 0.969 |
| 2751 | **V** | 2748 | 2754 | RQVVNVV | 0.947 |
| 2752 | **N** | 2749 | 2755 | QVVNVVT | 0.942 |
| 2753 | **V** | 2750 | 2756 | VVNVVTT | 0.952 |
| 2754 | **V** | 2751 | 2757 | VNVVTTK | 0.979 |
| 2755 | **T** | 2752 | 2758 | NVVTTKI | 1.003 |
| 2756 | **T** | 2753 | 2759 | VVTTKIA | 1.009 |
| 2757 | **K** | 2754 | 2760 | VTTKIAL | 1.0 |
| 2758 | **I** | 2755 | 2761 | TTKIALK | 0.981 |
| 2759 | **A** | 2756 | 2762 | TKIALKG | 0.979 |
| 2760 | **L** | 2757 | 2763 | KIALKGG | 1.004 |
| 2761 | **K** | 2758 | 2764 | IALKGGK | 1.042 |
| 2762 | **G** | 2759 | 2765 | ALKGGKI | 1.069 |
| 2763 | **G** | 2760 | 2766 | LKGGKIV | 1.067 |
| 2764 | **K** | 2761 | 2767 | KGGKIVN | 1.041 |
| 2765 | **I** | 2762 | 2768 | GGKIVNN | 1.002 |
| 2766 | **V** | 2763 | 2769 | GKIVNNW | 0.977 |
| 2767 | **N** | 2764 | 2770 | KIVNNWL | 0.968 |
| 2768 | **N** | 2765 | 2771 | IVNNWLK | 0.971 |
| 2769 | **W** | 2766 | 2772 | VNNWLKQ | 0.981 |
| 2770 | **L** | 2767 | 2773 | NNWLKQL | 0.992 |
| 2771 | **K** | 2768 | 2774 | NWLKQLI | 0.997 |
| 2772 | **Q** | 2769 | 2775 | WLKQLIK | 0.995 |
| 2773 | **L** | 2770 | 2776 | LKQLIKV | 0.989 |
| 2774 | **I** | 2771 | 2777 | KQLIKVT | 0.979 |
| 2775 | **K** | 2772 | 2778 | QLIKVTL | 0.977 |
| 2776 | **V** | 2773 | 2779 | LIKVTLV | 0.968 |
| 2777 | **T** | 2774 | 2780 | IKVTLVF | 0.957 |
| 2778 | **L** | 2775 | 2781 | KVTLVFL | 0.947 |
| 2779 | **V** | 2776 | 2782 | VTLVFLF | 0.93 |
| 2780 | **F** | 2777 | 2783 | TLVFLFV | 0.922 |
| 2781 | **L** | 2778 | 2784 | LVFLFVA | 0.917 |
| 2782 | **F** | 2779 | 2785 | VFLFVAA | 0.911 |
| 2783 | **V** | 2780 | 2786 | FLFVAAI | 0.905 |
| 2784 | **A** | 2781 | 2787 | LFVAAIF | 0.899 |
| 2785 | **A** | 2782 | 2788 | FVAAIFY | 0.892 |
| 2786 | **I** | 2783 | 2789 | VAAIFYL | 0.887 |
| 2787 | **F** | 2784 | 2790 | AAIFYLI | 0.887 |
| 2788 | **Y** | 2785 | 2791 | AIFYLIT | 0.895 |
| 2789 | **L** | 2786 | 2792 | IFYLITP | 0.922 |
| 2790 | **I** | 2787 | 2793 | FYLITPV | 0.948 |
| 2791 | **T** | 2788 | 2794 | YLITPVH | 0.973 |
| 2792 | **P** | 2789 | 2795 | LITPVHV | 0.98 |
| 2793 | **V** | 2790 | 2796 | ITPVHVM | 0.958 |
| 2794 | **H** | 2791 | 2797 | TPVHVMS | 0.943 |
| 2795 | **V** | 2792 | 2798 | PVHVMSK | 0.94 |
| 2796 | **M** | 2793 | 2799 | VHVMSKH | 0.949 |
| 2797 | **S** | 2794 | 2800 | HVMSKHT | 0.985 |
| 2798 | **K** | 2795 | 2801 | VMSKHTD | 1.015 |
| 2799 | **H** | 2796 | 2802 | MSKHTDF | 1.025 |
| 2800 | **T** | 2797 | 2803 | SKHTDFS | 1.034 |
| 2801 | **D** | 2798 | 2804 | KHTDFSS | 1.038 |
| 2802 | **F** | 2799 | 2805 | HTDFSSE | 1.037 |
| 2803 | **S** | 2800 | 2806 | TDFSSEI | 1.043 |
| 2804 | **S** | 2801 | 2807 | DFSSEII | 1.035 |
| 2805 | **E** | 2802 | 2808 | FSSEIIG | 1.004 |
| 2806 | **I** | 2803 | 2809 | SSEIIGY | 0.972 |
| 2807 | **I** | 2804 | 2810 | SEIIGYK | 0.952 |
| 2808 | **G** | 2805 | 2811 | EIIGYKA | 0.946 |
| 2809 | **Y** | 2806 | 2812 | IIGYKAI | 0.953 |
| 2810 | **K** | 2807 | 2813 | IGYKAID | 0.973 |
| 2811 | **A** | 2808 | 2814 | GYKAIDG | 0.987 |
| 2812 | **I** | 2809 | 2815 | YKAIDGG | 1.005 |
| 2813 | **D** | 2810 | 2816 | KAIDGGV | 1.03 |
| 2814 | **G** | 2811 | 2817 | AIDGGVT | 1.044 |
| 2815 | **G** | 2812 | 2818 | IDGGVTR | 1.047 |
| 2816 | **V** | 2813 | 2819 | DGGVTRD | 1.047 |
| 2817 | **T** | 2814 | 2820 | GGVTRDI | 1.035 |
| 2818 | **R** | 2815 | 2821 | GVTRDIA | 1.019 |
| 2819 | **D** | 2816 | 2822 | VTRDIAS | 1.011 |
| 2820 | **I** | 2817 | 2823 | TRDIAST | 0.998 |
| 2821 | **A** | 2818 | 2824 | RDIASTD | 1.0 |
| 2822 | **S** | 2819 | 2825 | DIASTDT | 1.015 |
| 2823 | **T** | 2820 | 2826 | IASTDTC | 1.019 |
| 2824 | **D** | 2821 | 2827 | ASTDTCF | 1.014 |
| 2825 | **T** | 2822 | 2828 | STDTCFA | 0.994 |
| 2826 | **C** | 2823 | 2829 | TDTCFAN | 0.965 |
| 2827 | **F** | 2824 | 2830 | DTCFANK | 0.96 |
| 2828 | **A** | 2825 | 2831 | TCFANKH | 0.967 |
| 2829 | **N** | 2826 | 2832 | CFANKHA | 0.979 |
| 2830 | **K** | 2827 | 2833 | FANKHAD | 0.99 |
| 2831 | **H** | 2828 | 2834 | ANKHADF | 0.978 |
| 2832 | **A** | 2829 | 2835 | NKHADFD | 0.972 |
| 2833 | **D** | 2830 | 2836 | KHADFDT | 0.973 |
| 2834 | **F** | 2831 | 2837 | HADFDTW | 0.978 |
| 2835 | **D** | 2832 | 2838 | ADFDTWF | 0.991 |
| 2836 | **T** | 2833 | 2839 | DFDTWFS | 0.993 |
| 2837 | **W** | 2834 | 2840 | FDTWFSQ | 0.995 |
| 2838 | **F** | 2835 | 2841 | DTWFSQR | 1.006 |
| 2839 | **S** | 2836 | 2842 | TWFSQRG | 1.033 |
| 2840 | **Q** | 2837 | 2843 | WFSQRGG | 1.068 |
| 2841 | **R** | 2838 | 2844 | FSQRGGS | 1.088 |
| 2842 | **G** | 2839 | 2845 | SQRGGSY | 1.096 |
| 2843 | **G** | 2840 | 2846 | QRGGSYT | 1.084 |
| 2844 | **S** | 2841 | 2847 | RGGSYTN | 1.065 |
| 2845 | **Y** | 2842 | 2848 | GGSYTND | 1.052 |
| 2846 | **T** | 2843 | 2849 | GSYTNDK | 1.051 |
| 2847 | **N** | 2844 | 2850 | SYTNDKA | 1.05 |
| 2848 | **D** | 2845 | 2851 | YTNDKAC | 1.035 |
| 2849 | **K** | 2846 | 2852 | TNDKACP | 1.015 |
| 2850 | **A** | 2847 | 2853 | NDKACPL | 0.98 |
| 2851 | **C** | 2848 | 2854 | DKACPLI | 0.946 |
| 2852 | **P** | 2849 | 2855 | KACPLIA | 0.928 |
| 2853 | **L** | 2850 | 2856 | ACPLIAA | 0.915 |
| 2854 | **I** | 2851 | 2857 | CPLIAAV | 0.905 |
| 2855 | **A** | 2852 | 2858 | PLIAAVI | 0.902 |
| 2856 | **A** | 2853 | 2859 | LIAAVIT | 0.908 |
| 2857 | **V** | 2854 | 2860 | IAAVITR | 0.925 |
| 2858 | **I** | 2855 | 2861 | AAVITRE | 0.953 |
| 2859 | **T** | 2856 | 2862 | AVITREV | 0.985 |
| 2860 | **R** | 2857 | 2863 | VITREVG | 1.0 |
| 2861 | **E** | 2858 | 2864 | ITREVGF | 0.997 |
| 2862 | **V** | 2859 | 2865 | TREVGFV | 0.979 |
| 2863 | **G** | 2860 | 2866 | REVGFVV | 0.952 |
| 2864 | **F** | 2861 | 2867 | EVGFVVP | 0.943 |
| 2865 | **V** | 2862 | 2868 | VGFVVPG | 0.951 |
| 2866 | **V** | 2863 | 2869 | GFVVPGL | 0.969 |
| 2867 | **P** | 2864 | 2870 | FVVPGLP | 1.0 |
| 2868 | **G** | 2865 | 2871 | VVPGLPG | 1.025 |
| 2869 | **L** | 2866 | 2872 | VPGLPGT | 1.043 |
| 2870 | **P** | 2867 | 2873 | PGLPGTI | 1.052 |
| 2871 | **G** | 2868 | 2874 | GLPGTIL | 1.044 |
| 2872 | **T** | 2869 | 2875 | LPGTILR | 1.025 |
| 2873 | **I** | 2870 | 2876 | PGTILRT | 1.006 |
| 2874 | **L** | 2871 | 2877 | GTILRTT | 1.004 |
| 2875 | **R** | 2872 | 2878 | TILRTTN | 1.02 |
| 2876 | **T** | 2873 | 2879 | ILRTTNG | 1.049 |
| 2877 | **T** | 2874 | 2880 | LRTTNGD | 1.078 |
| 2878 | **N** | 2875 | 2881 | RTTNGDF | 1.086 |
| 2879 | **G** | 2876 | 2882 | TTNGDFL | 1.072 |
| 2880 | **D** | 2877 | 2883 | TNGDFLH | 1.035 |
| 2881 | **F** | 2878 | 2884 | NGDFLHF | 0.986 |
| 2882 | **L** | 2879 | 2885 | GDFLHFL | 0.951 |
| 2883 | **H** | 2880 | 2886 | DFLHFLP | 0.938 |
| 2884 | **F** | 2881 | 2887 | FLHFLPR | 0.948 |
| 2885 | **L** | 2882 | 2888 | LHFLPRV | 0.971 |
| 2886 | **P** | 2883 | 2889 | HFLPRVF | 0.987 |
| 2887 | **R** | 2884 | 2890 | FLPRVFS | 0.983 |
| 2888 | **V** | 2885 | 2891 | LPRVFSA | 0.966 |
| 2889 | **F** | 2886 | 2892 | PRVFSAV | 0.947 |
| 2890 | **S** | 2887 | 2893 | RVFSAVG | 0.94 |
| 2891 | **A** | 2888 | 2894 | VFSAVGN | 0.948 |
| 2892 | **V** | 2889 | 2895 | FSAVGNI | 0.958 |
| 2893 | **G** | 2890 | 2896 | SAVGNIC | 0.968 |
| 2894 | **N** | 2891 | 2897 | AVGNICY | 0.963 |
| 2895 | **I** | 2892 | 2898 | VGNICYT | 0.955 |
| 2896 | **C** | 2893 | 2899 | GNICYTP | 0.961 |
| 2897 | **Y** | 2894 | 2900 | NICYTPS | 0.982 |
| 2898 | **T** | 2895 | 2901 | ICYTPSK | 1.02 |
| 2899 | **P** | 2896 | 2902 | CYTPSKL | 1.049 |
| 2900 | **S** | 2897 | 2903 | YTPSKLI | 1.058 |
| 2901 | **K** | 2898 | 2904 | TPSKLIE | 1.037 |
| 2902 | **L** | 2899 | 2905 | PSKLIEY | 0.999 |
| 2903 | **I** | 2900 | 2906 | SKLIEYT | 0.972 |
| 2904 | **E** | 2901 | 2907 | KLIEYTD | 0.968 |
| 2905 | **Y** | 2902 | 2908 | LIEYTDF | 0.977 |
| 2906 | **T** | 2903 | 2909 | IEYTDFA | 0.993 |
| 2907 | **D** | 2904 | 2910 | EYTDFAT | 1.003 |
| 2908 | **F** | 2905 | 2911 | YTDFATS | 0.998 |
| 2909 | **A** | 2906 | 2912 | TDFATSA | 0.996 |
| 2910 | **T** | 2907 | 2913 | DFATSAC | 0.995 |
| 2911 | **S** | 2908 | 2914 | FATSACV | 0.984 |
| 2912 | **A** | 2909 | 2915 | ATSACVL | 0.969 |
| 2913 | **C** | 2910 | 2916 | TSACVLA | 0.947 |
| 2914 | **V** | 2911 | 2917 | SACVLAA | 0.929 |
| 2915 | **L** | 2912 | 2918 | ACVLAAE | 0.92 |
| 2916 | **A** | 2913 | 2919 | CVLAAEC | 0.922 |
| 2917 | **A** | 2914 | 2920 | VLAAECT | 0.929 |
| 2918 | **E** | 2915 | 2921 | LAAECTI | 0.932 |
| 2919 | **C** | 2916 | 2922 | AAECTIF | 0.933 |
| 2920 | **T** | 2917 | 2923 | AECTIFK | 0.938 |
| 2921 | **I** | 2918 | 2924 | ECTIFKD | 0.951 |
| 2922 | **F** | 2919 | 2925 | CTIFKDA | 0.976 |
| 2923 | **K** | 2920 | 2926 | TIFKDAS | 1.011 |
| 2924 | **D** | 2921 | 2927 | IFKDASG | 1.042 |
| 2925 | **A** | 2922 | 2928 | FKDASGK | 1.064 |
| 2926 | **S** | 2923 | 2929 | KDASGKP | 1.079 |
| 2927 | **G** | 2924 | 2930 | DASGKPV | 1.082 |
| 2928 | **K** | 2925 | 2931 | ASGKPVP | 1.068 |
| 2929 | **P** | 2926 | 2932 | SGKPVPY | 1.043 |
| 2930 | **V** | 2927 | 2933 | GKPVPYC | 1.006 |
| 2931 | **P** | 2928 | 2934 | KPVPYCY | 0.969 |
| 2932 | **Y** | 2929 | 2935 | PVPYCYD | 0.956 |
| 2933 | **C** | 2930 | 2936 | VPYCYDT | 0.961 |
| 2934 | **Y** | 2931 | 2937 | PYCYDTN | 0.982 |
| 2935 | **D** | 2932 | 2938 | YCYDTNV | 1.005 |
| 2936 | **T** | 2933 | 2939 | CYDTNVL | 1.011 |
| 2937 | **N** | 2934 | 2940 | YDTNVLE | 1.006 |
| 2938 | **V** | 2935 | 2941 | DTNVLEG | 1.004 |
| 2939 | **L** | 2936 | 2942 | TNVLEGS | 1.009 |
| 2940 | **E** | 2937 | 2943 | NVLEGSV | 1.021 |
| 2941 | **G** | 2938 | 2944 | VLEGSVA | 1.026 |
| 2942 | **S** | 2939 | 2945 | LEGSVAY | 1.009 |
| 2943 | **V** | 2940 | 2946 | EGSVAYE | 0.984 |
| 2944 | **A** | 2941 | 2947 | GSVAYES | 0.969 |
| 2945 | **Y** | 2942 | 2948 | SVAYESL | 0.967 |
| 2946 | **E** | 2943 | 2949 | VAYESLR | 0.984 |
| 2947 | **S** | 2944 | 2950 | AYESLRP | 1.004 |
| 2948 | **L** | 2945 | 2951 | YESLRPD | 1.015 |
| 2949 | **R** | 2946 | 2952 | ESLRPDT | 1.028 |
| 2950 | **P** | 2947 | 2953 | SLRPDTR | 1.035 |
| 2951 | **D** | 2948 | 2954 | LRPDTRY | 1.033 |
| 2952 | **T** | 2949 | 2955 | RPDTRYV | 1.024 |
| 2953 | **R** | 2950 | 2956 | PDTRYVL | 0.999 |
| 2954 | **Y** | 2951 | 2957 | DTRYVLM | 0.964 |
| 2955 | **V** | 2952 | 2958 | TRYVLMD | 0.947 |
| 2956 | **L** | 2953 | 2959 | RYVLMDG | 0.951 |
| 2957 | **M** | 2954 | 2960 | YVLMDGS | 0.973 |
| 2958 | **D** | 2955 | 2961 | VLMDGSI | 1.007 |
| 2959 | **G** | 2956 | 2962 | LMDGSII | 1.019 |
| 2960 | **S** | 2957 | 2963 | MDGSIIQ | 1.0 |
| 2961 | **I** | 2958 | 2964 | DGSIIQF | 0.966 |
| 2962 | **I** | 2959 | 2965 | GSIIQFP | 0.943 |
| 2963 | **Q** | 2960 | 2966 | SIIQFPN | 0.945 |
| 2964 | **F** | 2961 | 2967 | IIQFPNT | 0.974 |
| 2965 | **P** | 2962 | 2968 | IQFPNTY | 1.011 |
| 2966 | **N** | 2963 | 2969 | QFPNTYL | 1.028 |
| 2967 | **T** | 2964 | 2970 | FPNTYLE | 1.025 |
| 2968 | **Y** | 2965 | 2971 | PNTYLEG | 1.018 |
| 2969 | **L** | 2966 | 2972 | NTYLEGS | 1.018 |
| 2970 | **E** | 2967 | 2973 | TYLEGSV | 1.028 |
| 2971 | **G** | 2968 | 2974 | YLEGSVR | 1.034 |
| 2972 | **S** | 2969 | 2975 | LEGSVRV | 1.02 |
| 2973 | **V** | 2970 | 2976 | EGSVRVV | 0.992 |
| 2974 | **R** | 2971 | 2977 | GSVRVVT | 0.967 |
| 2975 | **V** | 2972 | 2978 | SVRVVTT | 0.96 |
| 2976 | **V** | 2973 | 2979 | VRVVTTF | 0.966 |
| 2977 | **T** | 2974 | 2980 | RVVTTFD | 0.99 |
| 2978 | **T** | 2975 | 2981 | VVTTFDS | 1.017 |
| 2979 | **F** | 2976 | 2982 | VTTFDSE | 1.034 |
| 2980 | **D** | 2977 | 2983 | TTFDSEY | 1.05 |
| 2981 | **S** | 2978 | 2984 | TFDSEYC | 1.044 |
| 2982 | **E** | 2979 | 2985 | FDSEYCR | 1.013 |
| 2983 | **Y** | 2980 | 2986 | DSEYCRH | 0.98 |
| 2984 | **C** | 2981 | 2987 | SEYCRHG | 0.953 |
| 2985 | **R** | 2982 | 2988 | EYCRHGT | 0.951 |
| 2986 | **H** | 2983 | 2989 | YCRHGTC | 0.969 |
| 2987 | **G** | 2984 | 2990 | CRHGTCE | 0.994 |
| 2988 | **T** | 2985 | 2991 | RHGTCER | 1.012 |
| 2989 | **C** | 2986 | 2992 | HGTCERS | 1.026 |
| 2990 | **E** | 2987 | 2993 | GTCERSE | 1.041 |
| 2991 | **R** | 2988 | 2994 | TCERSEA | 1.053 |
| 2992 | **S** | 2989 | 2995 | CERSEAG | 1.058 |
| 2993 | **E** | 2990 | 2996 | ERSEAGV | 1.041 |
| 2994 | **A** | 2991 | 2997 | RSEAGVC | 1.012 |
| 2995 | **G** | 2992 | 2998 | SEAGVCV | 0.976 |
| 2996 | **V** | 2993 | 2999 | EAGVCVS | 0.955 |
| 2997 | **C** | 2994 | 3000 | AGVCVST | 0.957 |
| 2998 | **V** | 2995 | 3001 | GVCVSTS | 0.982 |
| 2999 | **S** | 2996 | 3002 | VCVSTSG | 1.028 |
| 3000 | **T** | 2997 | 3003 | CVSTSGR | 1.065 |
| 3001 | **S** | 2998 | 3004 | VSTSGRW | 1.082 |
| 3002 | **G** | 2999 | 3005 | STSGRWV | 1.069 |
| 3003 | **R** | 3000 | 3006 | TSGRWVL | 1.03 |
| 3004 | **W** | 3001 | 3007 | SGRWVLN | 0.992 |
| 3005 | **V** | 3002 | 3008 | GRWVLNN | 0.976 |
| 3006 | **L** | 3003 | 3009 | RWVLNND | 0.987 |
| 3007 | **N** | 3004 | 3010 | WVLNNDY | 1.007 |
| 3008 | **N** | 3005 | 3011 | VLNNDYY | 1.024 |
| 3009 | **D** | 3006 | 3012 | LNNDYYR | 1.022 |
| 3010 | **Y** | 3007 | 3013 | NNDYYRS | 1.008 |
| 3011 | **Y** | 3008 | 3014 | NDYYRSL | 0.997 |
| 3012 | **R** | 3009 | 3015 | DYYRSLP | 1.001 |
| 3013 | **S** | 3010 | 3016 | YYRSLPG | 1.011 |
| 3014 | **L** | 3011 | 3017 | YRSLPGV | 1.016 |
| 3015 | **P** | 3012 | 3018 | RSLPGVF | 1.016 |
| 3016 | **G** | 3013 | 3019 | SLPGVFC | 0.993 |
| 3017 | **V** | 3014 | 3020 | LPGVFCG | 0.962 |
| 3018 | **F** | 3015 | 3021 | PGVFCGV | 0.942 |
| 3019 | **C** | 3016 | 3022 | GVFCGVD | 0.926 |
| 3020 | **G** | 3017 | 3023 | VFCGVDA | 0.927 |
| 3021 | **V** | 3018 | 3024 | FCGVDAV | 0.936 |
| 3022 | **D** | 3019 | 3025 | CGVDAVN | 0.939 |
| 3023 | **A** | 3020 | 3026 | GVDAVNL | 0.942 |
| 3024 | **V** | 3021 | 3027 | VDAVNLL | 0.942 |
| 3025 | **N** | 3022 | 3028 | DAVNLLT | 0.949 |
| 3026 | **L** | 3023 | 3029 | AVNLLTN | 0.964 |
| 3027 | **L** | 3024 | 3030 | VNLLTNM | 0.971 |
| 3028 | **T** | 3025 | 3031 | NLLTNMF | 0.975 |
| 3029 | **N** | 3026 | 3032 | LLTNMFT | 0.97 |
| 3030 | **M** | 3027 | 3033 | LTNMFTP | 0.966 |
| 3031 | **F** | 3028 | 3034 | TNMFTPL | 0.974 |
| 3032 | **T** | 3029 | 3035 | NMFTPLI | 0.984 |
| 3033 | **P** | 3030 | 3036 | MFTPLIQ | 0.992 |
| 3034 | **L** | 3031 | 3037 | FTPLIQP | 0.996 |
| 3035 | **I** | 3032 | 3038 | TPLIQPI | 0.996 |
| 3036 | **Q** | 3033 | 3039 | PLIQPIG | 0.999 |
| 3037 | **P** | 3034 | 3040 | LIQPIGA | 0.998 |
| 3038 | **I** | 3035 | 3041 | IQPIGAL | 0.99 |
| 3039 | **G** | 3036 | 3042 | QPIGALD | 0.974 |
| 3040 | **A** | 3037 | 3043 | PIGALDI | 0.962 |
| 3041 | **L** | 3038 | 3044 | IGALDIS | 0.953 |
| 3042 | **D** | 3039 | 3045 | GALDISA | 0.957 |
| 3043 | **I** | 3040 | 3046 | ALDISAS | 0.965 |
| 3044 | **S** | 3041 | 3047 | LDISASI | 0.961 |
| 3045 | **A** | 3042 | 3048 | DISASIV | 0.958 |
| 3046 | **S** | 3043 | 3049 | ISASIVA | 0.944 |
| 3047 | **I** | 3044 | 3050 | SASIVAG | 0.938 |
| 3048 | **V** | 3045 | 3051 | ASIVAGG | 0.949 |
| 3049 | **A** | 3046 | 3052 | SIVAGGI | 0.958 |
| 3050 | **G** | 3047 | 3053 | IVAGGIV | 0.972 |
| 3051 | **G** | 3048 | 3054 | VAGGIVA | 0.968 |
| 3052 | **I** | 3049 | 3055 | AGGIVAI | 0.945 |
| 3053 | **V** | 3050 | 3056 | GGIVAIV | 0.924 |
| 3054 | **A** | 3051 | 3057 | GIVAIVV | 0.906 |
| 3055 | **I** | 3052 | 3058 | IVAIVVT | 0.901 |
| 3056 | **V** | 3053 | 3059 | VAIVVTC | 0.906 |
| 3057 | **V** | 3054 | 3060 | AIVVTCL | 0.91 |
| 3058 | **T** | 3055 | 3061 | IVVTCLA | 0.911 |
| 3059 | **C** | 3056 | 3062 | VVTCLAY | 0.904 |
| 3060 | **L** | 3057 | 3063 | VTCLAYY | 0.894 |
| 3061 | **A** | 3058 | 3064 | TCLAYYF | 0.882 |
| 3062 | **Y** | 3059 | 3065 | CLAYYFM | 0.872 |
| 3063 | **Y** | 3060 | 3066 | LAYYFMR | 0.871 |
| 3064 | **F** | 3061 | 3067 | AYYFMRF | 0.878 |
| 3065 | **M** | 3062 | 3068 | YYFMRFR | 0.893 |
| 3066 | **R** | 3063 | 3069 | YFMRFRR | 0.921 |
| 3067 | **F** | 3064 | 3070 | FMRFRRA | 0.947 |
| 3068 | **R** | 3065 | 3071 | MRFRRAF | 0.966 |
| 3069 | **R** | 3066 | 3072 | RFRRAFG | 0.979 |
| 3070 | **A** | 3067 | 3073 | FRRAFGE | 0.982 |
| 3071 | **F** | 3068 | 3074 | RRAFGEY | 0.983 |
| 3072 | **G** | 3069 | 3075 | RAFGEYS | 0.986 |
| 3073 | **E** | 3070 | 3076 | AFGEYSH | 0.983 |
| 3074 | **Y** | 3071 | 3077 | FGEYSHV | 0.971 |
| 3075 | **S** | 3072 | 3078 | GEYSHVV | 0.955 |
| 3076 | **H** | 3073 | 3079 | EYSHVVA | 0.937 |
| 3077 | **V** | 3074 | 3080 | YSHVVAF | 0.922 |
| 3078 | **V** | 3075 | 3081 | SHVVAFN | 0.92 |
| 3079 | **A** | 3076 | 3082 | HVVAFNT | 0.93 |
| 3080 | **F** | 3077 | 3083 | VVAFNTL | 0.947 |
| 3081 | **N** | 3078 | 3084 | VAFNTLL | 0.966 |
| 3082 | **T** | 3079 | 3085 | AFNTLLF | 0.974 |
| 3083 | **L** | 3080 | 3086 | FNTLLFL | 0.965 |
| 3084 | **L** | 3081 | 3087 | NTLLFLM | 0.945 |
| 3085 | **F** | 3082 | 3088 | TLLFLMS | 0.925 |
| 3086 | **L** | 3083 | 3089 | LLFLMSF | 0.912 |
| 3087 | **M** | 3084 | 3090 | LFLMSFT | 0.908 |
| 3088 | **S** | 3085 | 3091 | FLMSFTV | 0.914 |
| 3089 | **F** | 3086 | 3092 | LMSFTVL | 0.919 |
| 3090 | **T** | 3087 | 3093 | MSFTVLC | 0.924 |
| 3091 | **V** | 3088 | 3094 | SFTVLCL | 0.929 |
| 3092 | **L** | 3089 | 3095 | FTVLCLT | 0.938 |
| 3093 | **C** | 3090 | 3096 | TVLCLTP | 0.958 |
| 3094 | **L** | 3091 | 3097 | VLCLTPV | 0.977 |
| 3095 | **T** | 3092 | 3098 | LCLTPVY | 0.994 |
| 3096 | **P** | 3093 | 3099 | CLTPVYS | 0.994 |
| 3097 | **V** | 3094 | 3100 | LTPVYSF | 0.973 |
| 3098 | **Y** | 3095 | 3101 | TPVYSFL | 0.955 |
| 3099 | **S** | 3096 | 3102 | PVYSFLP | 0.948 |
| 3100 | **F** | 3097 | 3103 | VYSFLPG | 0.956 |
| 3101 | **L** | 3098 | 3104 | YSFLPGV | 0.976 |
| 3102 | **P** | 3099 | 3105 | SFLPGVY | 0.992 |
| 3103 | **G** | 3100 | 3106 | FLPGVYS | 0.989 |
| 3104 | **V** | 3101 | 3107 | LPGVYSV | 0.97 |
| 3105 | **Y** | 3102 | 3108 | PGVYSVI | 0.948 |
| 3106 | **S** | 3103 | 3109 | GVYSVIY | 0.921 |
| 3107 | **V** | 3104 | 3110 | VYSVIYL | 0.905 |
| 3108 | **I** | 3105 | 3111 | YSVIYLY | 0.89 |
| 3109 | **Y** | 3106 | 3112 | SVIYLYL | 0.884 |
| 3110 | **L** | 3107 | 3113 | VIYLYLT | 0.89 |
| 3111 | **Y** | 3108 | 3114 | IYLYLTF | 0.895 |
| 3112 | **L** | 3109 | 3115 | YLYLTFY | 0.906 |
| 3113 | **T** | 3110 | 3116 | LYLTFYL | 0.912 |
| 3114 | **F** | 3111 | 3117 | YLTFYLT | 0.918 |
| 3115 | **Y** | 3112 | 3118 | LTFYLTN | 0.938 |
| 3116 | **L** | 3113 | 3119 | TFYLTND | 0.974 |
| 3117 | **T** | 3114 | 3120 | FYLTNDV | 1.011 |
| 3118 | **N** | 3115 | 3121 | YLTNDVS | 1.033 |
| 3119 | **D** | 3116 | 3122 | LTNDVSF | 1.03 |
| 3120 | **V** | 3117 | 3123 | TNDVSFL | 1.0 |
| 3121 | **S** | 3118 | 3124 | NDVSFLA | 0.963 |
| 3122 | **F** | 3119 | 3125 | DVSFLAH | 0.932 |
| 3123 | **L** | 3120 | 3126 | VSFLAHI | 0.913 |
| 3124 | **A** | 3121 | 3127 | SFLAHIQ | 0.902 |
| 3125 | **H** | 3122 | 3128 | FLAHIQW | 0.898 |
| 3126 | **I** | 3123 | 3129 | LAHIQWM | 0.89 |
| 3127 | **Q** | 3124 | 3130 | AHIQWMV | 0.886 |
| 3128 | **W** | 3125 | 3131 | HIQWMVM | 0.878 |
| 3129 | **M** | 3126 | 3132 | IQWMVMF | 0.868 |
| 3130 | **V** | 3127 | 3133 | QWMVMFT | 0.879 |
| 3131 | **M** | 3128 | 3134 | WMVMFTP | 0.9 |
| 3132 | **F** | 3129 | 3135 | MVMFTPL | 0.936 |
| 3133 | **T** | 3130 | 3136 | VMFTPLV | 0.973 |
| 3134 | **P** | 3131 | 3137 | MFTPLVP | 0.987 |
| 3135 | **L** | 3132 | 3138 | FTPLVPF | 0.979 |
| 3136 | **V** | 3133 | 3139 | TPLVPFW | 0.952 |
| 3137 | **P** | 3134 | 3140 | PLVPFWI | 0.922 |
| 3138 | **F** | 3135 | 3141 | LVPFWIT | 0.899 |
| 3139 | **W** | 3136 | 3142 | VPFWITI | 0.886 |
| 3140 | **I** | 3137 | 3143 | PFWITIA | 0.888 |
| 3141 | **T** | 3138 | 3144 | FWITIAY | 0.889 |
| 3142 | **I** | 3139 | 3145 | WITIAYI | 0.886 |
| 3143 | **A** | 3140 | 3146 | ITIAYII | 0.883 |
| 3144 | **Y** | 3141 | 3147 | TIAYIIC | 0.877 |
| 3145 | **I** | 3142 | 3148 | IAYIICI | 0.877 |
| 3146 | **I** | 3143 | 3149 | AYIICIS | 0.892 |
| 3147 | **C** | 3144 | 3150 | YIICIST | 0.921 |
| 3148 | **I** | 3145 | 3151 | IICISTK | 0.958 |
| 3149 | **S** | 3146 | 3152 | ICISTKH | 0.995 |
| 3150 | **T** | 3147 | 3153 | CISTKHF | 1.013 |
| 3151 | **K** | 3148 | 3154 | ISTKHFY | 1.004 |
| 3152 | **H** | 3149 | 3155 | STKHFYW | 0.967 |
| 3153 | **F** | 3150 | 3156 | TKHFYWF | 0.924 |
| 3154 | **Y** | 3151 | 3157 | KHFYWFF | 0.889 |
| 3155 | **W** | 3152 | 3158 | HFYWFFS | 0.882 |
| 3156 | **F** | 3153 | 3159 | FYWFFSN | 0.906 |
| 3157 | **F** | 3154 | 3160 | YWFFSNY | 0.932 |
| 3158 | **S** | 3155 | 3161 | WFFSNYL | 0.962 |
| 3159 | **N** | 3156 | 3162 | FFSNYLK | 0.981 |
| 3160 | **Y** | 3157 | 3163 | FSNYLKR | 0.989 |
| 3161 | **L** | 3158 | 3164 | SNYLKRR | 1.003 |
| 3162 | **K** | 3159 | 3165 | NYLKRRV | 1.011 |
| 3163 | **R** | 3160 | 3166 | YLKRRVV | 1.006 |
| 3164 | **R** | 3161 | 3167 | LKRRVVF | 0.992 |
| 3165 | **V** | 3162 | 3168 | KRRVVFN | 0.97 |
| 3166 | **V** | 3163 | 3169 | RRVVFNG | 0.957 |
| 3167 | **F** | 3164 | 3170 | RVVFNGV | 0.96 |
| 3168 | **N** | 3165 | 3171 | VVFNGVS | 0.97 |
| 3169 | **G** | 3166 | 3172 | VFNGVSF | 0.978 |
| 3170 | **V** | 3167 | 3173 | FNGVSFS | 0.978 |
| 3171 | **S** | 3168 | 3174 | NGVSFST | 0.979 |
| 3172 | **F** | 3169 | 3175 | GVSFSTF | 0.979 |
| 3173 | **S** | 3170 | 3176 | VSFSTFE | 0.991 |
| 3174 | **T** | 3171 | 3177 | SFSTFEE | 1.002 |
| 3175 | **F** | 3172 | 3178 | FSTFEEA | 1.002 |
| 3176 | **E** | 3173 | 3179 | STFEEAA | 0.998 |
| 3177 | **E** | 3174 | 3180 | TFEEAAL | 0.982 |
| 3178 | **A** | 3175 | 3181 | FEEAALC | 0.956 |
| 3179 | **A** | 3176 | 3182 | EEAALCT | 0.935 |
| 3180 | **L** | 3177 | 3183 | EAALCTF | 0.919 |
| 3181 | **C** | 3178 | 3184 | AALCTFL | 0.909 |
| 3182 | **T** | 3179 | 3185 | ALCTFLL | 0.915 |
| 3183 | **F** | 3180 | 3186 | LCTFLLN | 0.926 |
| 3184 | **L** | 3181 | 3187 | CTFLLNK | 0.947 |
| 3185 | **L** | 3182 | 3188 | TFLLNKE | 0.975 |
| 3186 | **N** | 3183 | 3189 | FLLNKEM | 0.992 |
| 3187 | **K** | 3184 | 3190 | LLNKEMY | 0.994 |
| 3188 | **E** | 3185 | 3191 | LNKEMYL | 0.976 |
| 3189 | **M** | 3186 | 3192 | NKEMYLK | 0.952 |
| 3190 | **Y** | 3187 | 3193 | KEMYLKL | 0.941 |
| 3191 | **L** | 3188 | 3194 | EMYLKLR | 0.953 |
| 3192 | **K** | 3189 | 3195 | MYLKLRS | 0.986 |
| 3193 | **L** | 3190 | 3196 | YLKLRSD | 1.019 |
| 3194 | **R** | 3191 | 3197 | LKLRSDV | 1.044 |
| 3195 | **S** | 3192 | 3198 | KLRSDVL | 1.05 |
| 3196 | **D** | 3193 | 3199 | LRSDVLL | 1.029 |
| 3197 | **V** | 3194 | 3200 | RSDVLLP | 0.997 |
| 3198 | **L** | 3195 | 3201 | SDVLLPL | 0.97 |
| 3199 | **L** | 3196 | 3202 | DVLLPLT | 0.958 |
| 3200 | **P** | 3197 | 3203 | VLLPLTQ | 0.963 |
| 3201 | **L** | 3198 | 3204 | LLPLTQY | 0.98 |
| 3202 | **T** | 3199 | 3205 | LPLTQYN | 0.996 |
| 3203 | **Q** | 3200 | 3206 | PLTQYNR | 1.003 |
| 3204 | **Y** | 3201 | 3207 | LTQYNRY | 1.0 |
| 3205 | **N** | 3202 | 3208 | TQYNRYL | 0.992 |
| 3206 | **R** | 3203 | 3209 | QYNRYLA | 0.975 |
| 3207 | **Y** | 3204 | 3210 | YNRYLAL | 0.953 |
| 3208 | **L** | 3205 | 3211 | NRYLALY | 0.937 |
| 3209 | **A** | 3206 | 3212 | RYLALYN | 0.928 |
| 3210 | **L** | 3207 | 3213 | YLALYNK | 0.939 |
| 3211 | **Y** | 3208 | 3214 | LALYNKY | 0.958 |
| 3212 | **N** | 3209 | 3215 | ALYNKYK | 0.986 |
| 3213 | **K** | 3210 | 3216 | LYNKYKY | 1.003 |
| 3214 | **Y** | 3211 | 3217 | YNKYKYF | 0.998 |
| 3215 | **K** | 3212 | 3218 | NKYKYFS | 0.996 |
| 3216 | **Y** | 3213 | 3219 | KYKYFSG | 0.986 |
| 3217 | **F** | 3214 | 3220 | YKYFSGA | 0.98 |
| 3218 | **S** | 3215 | 3221 | KYFSGAM | 0.983 |
| 3219 | **G** | 3216 | 3222 | YFSGAMD | 0.982 |
| 3220 | **A** | 3217 | 3223 | FSGAMDT | 0.985 |
| 3221 | **M** | 3218 | 3224 | SGAMDTT | 0.994 |
| 3222 | **D** | 3219 | 3225 | GAMDTTS | 1.018 |
| 3223 | **T** | 3220 | 3226 | AMDTTSY | 1.032 |
| 3224 | **T** | 3221 | 3227 | MDTTSYR | 1.04 |
| 3225 | **S** | 3222 | 3228 | DTTSYRE | 1.039 |
| 3226 | **Y** | 3223 | 3229 | TTSYREA | 1.019 |
| 3227 | **R** | 3224 | 3230 | TSYREAA | 1.004 |
| 3228 | **E** | 3225 | 3231 | SYREAAC | 0.984 |
| 3229 | **A** | 3226 | 3232 | YREAACC | 0.96 |
| 3230 | **A** | 3227 | 3233 | REAACCH | 0.939 |
| 3231 | **C** | 3228 | 3234 | EAACCHL | 0.924 |
| 3232 | **C** | 3229 | 3235 | AACCHLA | 0.917 |
| 3233 | **H** | 3230 | 3236 | ACCHLAK | 0.925 |
| 3234 | **L** | 3231 | 3237 | CCHLAKA | 0.94 |
| 3235 | **A** | 3232 | 3238 | CHLAKAL | 0.955 |
| 3236 | **K** | 3233 | 3239 | HLAKALN | 0.973 |
| 3237 | **A** | 3234 | 3240 | LAKALND | 0.985 |
| 3238 | **L** | 3235 | 3241 | AKALNDF | 0.992 |
| 3239 | **N** | 3236 | 3242 | KALNDFS | 1.002 |
| 3240 | **D** | 3237 | 3243 | ALNDFSN | 1.015 |
| 3241 | **F** | 3238 | 3244 | LNDFSNS | 1.032 |
| 3242 | **S** | 3239 | 3245 | NDFSNSG | 1.062 |
| 3243 | **N** | 3240 | 3246 | DFSNSGS | 1.095 |
| 3244 | **S** | 3241 | 3247 | FSNSGSD | 1.119 |
| 3245 | **G** | 3242 | 3248 | SNSGSDV | 1.123 |
| 3246 | **S** | 3243 | 3249 | NSGSDVL | 1.1 |
| 3247 | **D** | 3244 | 3250 | SGSDVLY | 1.054 |
| 3248 | **V** | 3245 | 3251 | GSDVLYQ | 1.007 |
| 3249 | **L** | 3246 | 3252 | SDVLYQP | 0.982 |
| 3250 | **Y** | 3247 | 3253 | DVLYQPP | 0.98 |
| 3251 | **Q** | 3248 | 3254 | VLYQPPQ | 1.007 |
| 3252 | **P** | 3249 | 3255 | LYQPPQT | 1.041 |
| 3253 | **P** | 3250 | 3256 | YQPPQTS | 1.066 |
| 3254 | **Q** | 3251 | 3257 | QPPQTSI | 1.08 |
| 3255 | **T** | 3252 | 3258 | PPQTSIT | 1.072 |
| 3256 | **S** | 3253 | 3259 | PQTSITS | 1.059 |
| 3257 | **I** | 3254 | 3260 | QTSITSA | 1.041 |
| 3258 | **T** | 3255 | 3261 | TSITSAV | 1.02 |
| 3259 | **S** | 3256 | 3262 | SITSAVL | 1.001 |
| 3260 | **A** | 3257 | 3263 | ITSAVLQ | 0.983 |
| 3261 | **V** | 3258 | 3264 | TSAVLQS | 0.984 |
| 3262 | **L** | 3259 | 3265 | SAVLQSG | 0.999 |
| 3263 | **Q** | 3260 | 3266 | AVLQSGF | 1.018 |
| 3264 | **S** | 3261 | 3267 | VLQSGFR | 1.038 |
| 3265 | **G** | 3262 | 3268 | LQSGFRK | 1.039 |
| 3266 | **F** | 3263 | 3269 | QSGFRKM | 1.02 |
| 3267 | **R** | 3264 | 3270 | SGFRKMA | 1.001 |
| 3268 | **K** | 3265 | 3271 | GFRKMAF | 0.975 |
| 3269 | **M** | 3266 | 3272 | FRKMAFP | 0.954 |
| 3270 | **A** | 3267 | 3273 | RKMAFPS | 0.964 |
| 3271 | **F** | 3268 | 3274 | KMAFPSG | 0.992 |
| 3272 | **P** | 3269 | 3275 | MAFPSGK | 1.037 |
| 3273 | **S** | 3270 | 3276 | AFPSGKV | 1.076 |
| 3274 | **G** | 3271 | 3277 | FPSGKVE | 1.088 |
| 3275 | **K** | 3272 | 3278 | PSGKVEG | 1.077 |
| 3276 | **V** | 3273 | 3279 | SGKVEGC | 1.044 |
| 3277 | **E** | 3274 | 3280 | GKVEGCM | 1.005 |
| 3278 | **G** | 3275 | 3281 | KVEGCMV | 0.968 |
| 3279 | **C** | 3276 | 3282 | VEGCMVQ | 0.928 |
| 3280 | **M** | 3277 | 3283 | EGCMVQV | 0.906 |
| 3281 | **V** | 3278 | 3284 | GCMVQVT | 0.904 |
| 3282 | **Q** | 3279 | 3285 | CMVQVTC | 0.911 |
| 3283 | **V** | 3280 | 3286 | MVQVTCG | 0.938 |
| 3284 | **T** | 3281 | 3287 | VQVTCGT | 0.964 |
| 3285 | **C** | 3282 | 3288 | QVTCGTT | 0.99 |
| 3286 | **G** | 3283 | 3289 | VTCGTTT | 1.02 |
| 3287 | **T** | 3284 | 3290 | TCGTTTL | 1.035 |
| 3288 | **T** | 3285 | 3291 | CGTTTLN | 1.04 |
| 3289 | **T** | 3286 | 3292 | GTTTLNG | 1.035 |
| 3290 | **L** | 3287 | 3293 | TTTLNGL | 1.019 |
| 3291 | **N** | 3288 | 3294 | TTLNGLW | 0.997 |
| 3292 | **G** | 3289 | 3295 | TLNGLWL | 0.976 |
| 3293 | **L** | 3290 | 3296 | LNGLWLD | 0.961 |
| 3294 | **W** | 3291 | 3297 | NGLWLDD | 0.958 |
| 3295 | **L** | 3292 | 3298 | GLWLDDV | 0.974 |
| 3296 | **D** | 3293 | 3299 | LWLDDVV | 0.99 |
| 3297 | **D** | 3294 | 3300 | WLDDVVY | 0.987 |
| 3298 | **V** | 3295 | 3301 | LDDVVYC | 0.963 |
| 3299 | **V** | 3296 | 3302 | DDVVYCP | 0.941 |
| 3300 | **Y** | 3297 | 3303 | DVVYCPR | 0.929 |
| 3301 | **C** | 3298 | 3304 | VVYCPRH | 0.94 |
| 3302 | **P** | 3299 | 3305 | VYCPRHV | 0.966 |
| 3303 | **R** | 3300 | 3306 | YCPRHVI | 0.97 |
| 3304 | **H** | 3301 | 3307 | CPRHVIC | 0.956 |
| 3305 | **V** | 3302 | 3308 | PRHVICT | 0.942 |
| 3306 | **I** | 3303 | 3309 | RHVICTS | 0.942 |
| 3307 | **C** | 3304 | 3310 | HVICTSE | 0.968 |
| 3308 | **T** | 3305 | 3311 | VICTSED | 1.017 |
| 3309 | **S** | 3306 | 3312 | ICTSEDM | 1.049 |
| 3310 | **E** | 3307 | 3313 | CTSEDML | 1.051 |
| 3311 | **D** | 3308 | 3314 | TSEDMLN | 1.034 |
| 3312 | **M** | 3309 | 3315 | SEDMLNP | 1.001 |
| 3313 | **L** | 3310 | 3316 | EDMLNPN | 0.99 |
| 3314 | **N** | 3311 | 3317 | DMLNPNY | 0.991 |
| 3315 | **P** | 3312 | 3318 | MLNPNYE | 1.0 |
| 3316 | **N** | 3313 | 3319 | LNPNYED | 1.013 |
| 3317 | **Y** | 3314 | 3320 | NPNYEDL | 1.014 |
| 3318 | **E** | 3315 | 3321 | PNYEDLL | 1.013 |
| 3319 | **D** | 3316 | 3322 | NYEDLLI | 1.001 |
| 3320 | **L** | 3317 | 3323 | YEDLLIR | 0.982 |
| 3321 | **L** | 3318 | 3324 | EDLLIRK | 0.975 |
| 3322 | **I** | 3319 | 3325 | DLLIRKS | 0.986 |
| 3323 | **R** | 3320 | 3326 | LLIRKSN | 1.013 |
| 3324 | **K** | 3321 | 3327 | LIRKSNH | 1.041 |
| 3325 | **S** | 3322 | 3328 | IRKSNHN | 1.051 |
| 3326 | **N** | 3323 | 3329 | RKSNHNF | 1.029 |
| 3327 | **H** | 3324 | 3330 | KSNHNFL | 0.994 |
| 3328 | **N** | 3325 | 3331 | SNHNFLV | 0.96 |
| 3329 | **F** | 3326 | 3332 | NHNFLVQ | 0.932 |
| 3330 | **L** | 3327 | 3333 | HNFLVQA | 0.928 |
| 3331 | **V** | 3328 | 3334 | NFLVQAG | 0.938 |
| 3332 | **Q** | 3329 | 3335 | FLVQAGN | 0.955 |
| 3333 | **A** | 3330 | 3336 | LVQAGNV | 0.982 |
| 3334 | **G** | 3331 | 3337 | VQAGNVQ | 0.991 |
| 3335 | **N** | 3332 | 3338 | QAGNVQL | 0.988 |
| 3336 | **V** | 3333 | 3339 | AGNVQLR | 0.973 |
| 3337 | **Q** | 3334 | 3340 | GNVQLRV | 0.948 |
| 3338 | **L** | 3335 | 3341 | NVQLRVI | 0.934 |
| 3339 | **R** | 3336 | 3342 | VQLRVIG | 0.922 |
| 3340 | **V** | 3337 | 3343 | QLRVIGH | 0.921 |
| 3341 | **I** | 3338 | 3344 | LRVIGHS | 0.923 |
| 3342 | **G** | 3339 | 3345 | RVIGHSM | 0.929 |
| 3343 | **H** | 3340 | 3346 | VIGHSMQ | 0.944 |
| 3344 | **S** | 3341 | 3347 | IGHSMQN | 0.955 |
| 3345 | **M** | 3342 | 3348 | GHSMQNC | 0.964 |
| 3346 | **Q** | 3343 | 3349 | HSMQNCV | 0.967 |
| 3347 | **N** | 3344 | 3350 | SMQNCVL | 0.959 |
| 3348 | **C** | 3345 | 3351 | MQNCVLK | 0.953 |
| 3349 | **V** | 3346 | 3352 | QNCVLKL | 0.956 |
| 3350 | **L** | 3347 | 3353 | NCVLKLK | 0.969 |
| 3351 | **K** | 3348 | 3354 | CVLKLKV | 0.988 |
| 3352 | **L** | 3349 | 3355 | VLKLKVD | 1.006 |
| 3353 | **K** | 3350 | 3356 | LKLKVDT | 1.024 |
| 3354 | **V** | 3351 | 3357 | KLKVDTA | 1.031 |
| 3355 | **D** | 3352 | 3358 | LKVDTAN | 1.039 |
| 3356 | **T** | 3353 | 3359 | KVDTANP | 1.043 |
| 3357 | **A** | 3354 | 3360 | VDTANPK | 1.044 |
| 3358 | **N** | 3355 | 3361 | DTANPKT | 1.048 |
| 3359 | **P** | 3356 | 3362 | TANPKTP | 1.053 |
| 3360 | **K** | 3357 | 3363 | ANPKTPK | 1.063 |
| 3361 | **T** | 3358 | 3364 | NPKTPKY | 1.061 |
| 3362 | **P** | 3359 | 3365 | PKTPKYK | 1.057 |
| 3363 | **K** | 3360 | 3366 | KTPKYKF | 1.04 |
| 3364 | **Y** | 3361 | 3367 | TPKYKFV | 1.012 |
| 3365 | **K** | 3362 | 3368 | PKYKFVR | 0.989 |
| 3366 | **F** | 3363 | 3369 | KYKFVRI | 0.963 |
| 3367 | **V** | 3364 | 3370 | YKFVRIQ | 0.953 |
| 3368 | **R** | 3365 | 3371 | KFVRIQP | 0.961 |
| 3369 | **I** | 3366 | 3372 | FVRIQPG | 0.988 |
| 3370 | **Q** | 3367 | 3373 | VRIQPGQ | 1.029 |
| 3371 | **P** | 3368 | 3374 | RIQPGQT | 1.063 |
| 3372 | **G** | 3369 | 3375 | IQPGQTF | 1.082 |
| 3373 | **Q** | 3370 | 3376 | QPGQTFS | 1.072 |
| 3374 | **T** | 3371 | 3377 | PGQTFSV | 1.038 |
| 3375 | **F** | 3372 | 3378 | GQTFSVL | 0.993 |
| 3376 | **S** | 3373 | 3379 | QTFSVLA | 0.954 |
| 3377 | **V** | 3374 | 3380 | TFSVLAC | 0.929 |
| 3378 | **L** | 3375 | 3381 | FSVLACY | 0.919 |
| 3379 | **A** | 3376 | 3382 | SVLACYN | 0.922 |
| 3380 | **C** | 3377 | 3383 | VLACYNG | 0.943 |
| 3381 | **Y** | 3378 | 3384 | LACYNGS | 0.979 |
| 3382 | **N** | 3379 | 3385 | ACYNGSP | 1.023 |
| 3383 | **G** | 3380 | 3386 | CYNGSPS | 1.072 |
| 3384 | **S** | 3381 | 3387 | YNGSPSG | 1.099 |
| 3385 | **P** | 3382 | 3388 | NGSPSGV | 1.096 |
| 3386 | **S** | 3383 | 3389 | GSPSGVY | 1.077 |
| 3387 | **G** | 3384 | 3390 | SPSGVYQ | 1.03 |
| 3388 | **V** | 3385 | 3391 | PSGVYQC | 0.979 |
| 3389 | **Y** | 3386 | 3392 | SGVYQCA | 0.941 |
| 3390 | **Q** | 3387 | 3393 | GVYQCAM | 0.907 |
| 3391 | **C** | 3388 | 3394 | VYQCAMR | 0.899 |
| 3392 | **A** | 3389 | 3395 | YQCAMRP | 0.912 |
| 3393 | **M** | 3390 | 3396 | QCAMRPN | 0.935 |
| 3394 | **R** | 3391 | 3397 | CAMRPNF | 0.967 |
| 3395 | **P** | 3392 | 3398 | AMRPNFT | 0.983 |
| 3396 | **N** | 3393 | 3399 | MRPNFTI | 0.985 |
| 3397 | **F** | 3394 | 3400 | RPNFTIK | 0.985 |
| 3398 | **T** | 3395 | 3401 | PNFTIKG | 0.994 |
| 3399 | **I** | 3396 | 3402 | NFTIKGS | 1.016 |
| 3400 | **K** | 3397 | 3403 | FTIKGSF | 1.035 |
| 3401 | **G** | 3398 | 3404 | TIKGSFL | 1.043 |
| 3402 | **S** | 3399 | 3405 | IKGSFLN | 1.028 |
| 3403 | **F** | 3400 | 3406 | KGSFLNG | 1.012 |
| 3404 | **L** | 3401 | 3407 | GSFLNGS | 1.01 |
| 3405 | **N** | 3402 | 3408 | SFLNGSC | 1.016 |
| 3406 | **G** | 3403 | 3409 | FLNGSCG | 1.033 |
| 3407 | **S** | 3404 | 3410 | LNGSCGS | 1.038 |
| 3408 | **C** | 3405 | 3411 | NGSCGSV | 1.03 |
| 3409 | **G** | 3406 | 3412 | GSCGSVG | 1.02 |
| 3410 | **S** | 3407 | 3413 | SCGSVGF | 1.001 |
| 3411 | **V** | 3408 | 3414 | CGSVGFN | 0.98 |
| 3412 | **G** | 3409 | 3415 | GSVGFNI | 0.964 |
| 3413 | **F** | 3410 | 3416 | SVGFNID | 0.952 |
| 3414 | **N** | 3411 | 3417 | VGFNIDY | 0.948 |
| 3415 | **I** | 3412 | 3418 | GFNIDYD | 0.952 |
| 3416 | **D** | 3413 | 3419 | FNIDYDC | 0.947 |
| 3417 | **Y** | 3414 | 3420 | NIDYDCV | 0.941 |
| 3418 | **D** | 3415 | 3421 | IDYDCVS | 0.93 |
| 3419 | **C** | 3416 | 3422 | DYDCVSF | 0.919 |
| 3420 | **V** | 3417 | 3423 | YDCVSFC | 0.918 |
| 3421 | **S** | 3418 | 3424 | DCVSFCY | 0.912 |
| 3422 | **F** | 3419 | 3425 | CVSFCYM | 0.9 |
| 3423 | **C** | 3420 | 3426 | VSFCYMH | 0.889 |
| 3424 | **Y** | 3421 | 3427 | SFCYMHH | 0.873 |
| 3425 | **M** | 3422 | 3428 | FCYMHHM | 0.864 |
| 3426 | **H** | 3423 | 3429 | CYMHHME | 0.87 |
| 3427 | **H** | 3424 | 3430 | YMHHMEL | 0.883 |
| 3428 | **M** | 3425 | 3431 | MHHMELP | 0.909 |
| 3429 | **E** | 3426 | 3432 | HHMELPT | 0.947 |
| 3430 | **L** | 3427 | 3433 | HMELPTG | 0.983 |
| 3431 | **P** | 3428 | 3434 | MELPTGV | 1.012 |
| 3432 | **T** | 3429 | 3435 | ELPTGVH | 1.018 |
| 3433 | **G** | 3430 | 3436 | LPTGVHA | 1.002 |
| 3434 | **V** | 3431 | 3437 | PTGVHAG | 0.98 |
| 3435 | **H** | 3432 | 3438 | TGVHAGT | 0.969 |
| 3436 | **A** | 3433 | 3439 | GVHAGTD | 0.983 |
| 3437 | **G** | 3434 | 3440 | VHAGTDL | 1.005 |
| 3438 | **T** | 3435 | 3441 | HAGTDLE | 1.028 |
| 3439 | **D** | 3436 | 3442 | AGTDLEG | 1.045 |
| 3440 | **L** | 3437 | 3443 | GTDLEGN | 1.045 |
| 3441 | **E** | 3438 | 3444 | TDLEGNF | 1.04 |
| 3442 | **G** | 3439 | 3445 | DLEGNFY | 1.029 |
| 3443 | **N** | 3440 | 3446 | LEGNFYG | 1.008 |
| 3444 | **F** | 3441 | 3447 | EGNFYGP | 0.997 |
| 3445 | **Y** | 3442 | 3448 | GNFYGPF | 0.989 |
| 3446 | **G** | 3443 | 3449 | NFYGPFV | 0.987 |
| 3447 | **P** | 3444 | 3450 | FYGPFVD | 0.995 |
| 3448 | **F** | 3445 | 3451 | YGPFVDR | 0.995 |
| 3449 | **V** | 3446 | 3452 | GPFVDRQ | 1.01 |
| 3450 | **D** | 3447 | 3453 | PFVDRQT | 1.034 |
| 3451 | **R** | 3448 | 3454 | FVDRQTA | 1.051 |
| 3452 | **Q** | 3449 | 3455 | VDRQTAQ | 1.062 |
| 3453 | **T** | 3450 | 3456 | DRQTAQA | 1.044 |
| 3454 | **A** | 3451 | 3457 | RQTAQAA | 1.011 |
| 3455 | **Q** | 3452 | 3458 | QTAQAAG | 0.981 |
| 3456 | **A** | 3453 | 3459 | TAQAAGT | 0.973 |
| 3457 | **A** | 3454 | 3460 | AQAAGTD | 0.984 |
| 3458 | **G** | 3455 | 3461 | QAAGTDT | 1.009 |
| 3459 | **T** | 3456 | 3462 | AAGTDTT | 1.034 |
| 3460 | **D** | 3457 | 3463 | AGTDTTI | 1.044 |
| 3461 | **T** | 3458 | 3464 | GTDTTIT | 1.042 |
| 3462 | **T** | 3459 | 3465 | TDTTITV | 1.026 |
| 3463 | **I** | 3460 | 3466 | DTTITVN | 1.002 |
| 3464 | **T** | 3461 | 3467 | TTITVNV | 0.978 |
| 3465 | **V** | 3462 | 3468 | TITVNVL | 0.959 |
| 3466 | **N** | 3463 | 3469 | ITVNVLA | 0.941 |
| 3467 | **V** | 3464 | 3470 | TVNVLAW | 0.922 |
| 3468 | **L** | 3465 | 3471 | VNVLAWL | 0.907 |
| 3469 | **A** | 3466 | 3472 | NVLAWLY | 0.888 |
| 3470 | **W** | 3467 | 3473 | VLAWLYA | 0.874 |
| 3471 | **L** | 3468 | 3474 | LAWLYAA | 0.874 |
| 3472 | **Y** | 3469 | 3475 | AWLYAAV | 0.874 |
| 3473 | **A** | 3470 | 3476 | WLYAAVI | 0.882 |
| 3474 | **A** | 3471 | 3477 | LYAAVIN | 0.898 |
| 3475 | **V** | 3472 | 3478 | YAAVING | 0.924 |
| 3476 | **I** | 3473 | 3479 | AAVINGD | 0.957 |
| 3477 | **N** | 3474 | 3480 | AVINGDR | 0.996 |
| 3478 | **G** | 3475 | 3481 | VINGDRW | 1.022 |
| 3479 | **D** | 3476 | 3482 | INGDRWF | 1.018 |
| 3480 | **R** | 3477 | 3483 | NGDRWFL | 1.003 |
| 3481 | **W** | 3478 | 3484 | GDRWFLN | 0.977 |
| 3482 | **F** | 3479 | 3485 | DRWFLNR | 0.964 |
| 3483 | **L** | 3480 | 3486 | RWFLNRF | 0.966 |
| 3484 | **N** | 3481 | 3487 | WFLNRFT | 0.978 |
| 3485 | **R** | 3482 | 3488 | FLNRFTT | 0.996 |
| 3486 | **F** | 3483 | 3489 | LNRFTTT | 1.008 |
| 3487 | **T** | 3484 | 3490 | NRFTTTL | 1.022 |
| 3488 | **T** | 3485 | 3491 | RFTTTLN | 1.027 |
| 3489 | **T** | 3486 | 3492 | FTTTLND | 1.029 |
| 3490 | **L** | 3487 | 3493 | TTTLNDF | 1.022 |
| 3491 | **N** | 3488 | 3494 | TTLNDFN | 1.01 |
| 3492 | **D** | 3489 | 3495 | TLNDFNL | 0.998 |
| 3493 | **F** | 3490 | 3496 | LNDFNLV | 0.974 |
| 3494 | **N** | 3491 | 3497 | NDFNLVA | 0.956 |
| 3495 | **L** | 3492 | 3498 | DFNLVAM | 0.935 |
| 3496 | **V** | 3493 | 3499 | FNLVAMK | 0.924 |
| 3497 | **A** | 3494 | 3500 | NLVAMKY | 0.926 |
| 3498 | **M** | 3495 | 3501 | LVAMKYN | 0.933 |
| 3499 | **K** | 3496 | 3502 | VAMKYNY | 0.951 |
| 3500 | **Y** | 3497 | 3503 | AMKYNYE | 0.961 |
| 3501 | **N** | 3498 | 3504 | MKYNYEP | 0.976 |
| 3502 | **Y** | 3499 | 3505 | KYNYEPL | 0.991 |
| 3503 | **E** | 3500 | 3506 | YNYEPLT | 1.006 |
| 3504 | **P** | 3501 | 3507 | NYEPLTQ | 1.029 |
| 3505 | **L** | 3502 | 3508 | YEPLTQD | 1.046 |
| 3506 | **T** | 3503 | 3509 | EPLTQDH | 1.058 |
| 3507 | **Q** | 3504 | 3510 | PLTQDHV | 1.058 |
| 3508 | **D** | 3505 | 3511 | LTQDHVD | 1.035 |
| 3509 | **H** | 3506 | 3512 | TQDHVDI | 1.0 |
| 3510 | **V** | 3507 | 3513 | QDHVDIL | 0.966 |
| 3511 | **D** | 3508 | 3514 | DHVDILG | 0.946 |
| 3512 | **I** | 3509 | 3515 | HVDILGP | 0.951 |
| 3513 | **L** | 3510 | 3516 | VDILGPL | 0.973 |
| 3514 | **G** | 3511 | 3517 | DILGPLS | 0.992 |
| 3515 | **P** | 3512 | 3518 | ILGPLSA | 1.004 |
| 3516 | **L** | 3513 | 3519 | LGPLSAQ | 1.003 |
| 3517 | **S** | 3514 | 3520 | GPLSAQT | 1.004 |
| 3518 | **A** | 3515 | 3521 | PLSAQTG | 1.014 |
| 3519 | **Q** | 3516 | 3522 | LSAQTGI | 1.015 |
| 3520 | **T** | 3517 | 3523 | SAQTGIA | 1.011 |
| 3521 | **G** | 3518 | 3524 | AQTGIAV | 0.991 |
| 3522 | **I** | 3519 | 3525 | QTGIAVL | 0.958 |
| 3523 | **A** | 3520 | 3526 | TGIAVLD | 0.937 |
| 3524 | **V** | 3521 | 3527 | GIAVLDM | 0.923 |
| 3525 | **L** | 3522 | 3528 | IAVLDMC | 0.919 |
| 3526 | **D** | 3523 | 3529 | AVLDMCA | 0.919 |
| 3527 | **M** | 3524 | 3530 | VLDMCAS | 0.917 |
| 3528 | **C** | 3525 | 3531 | LDMCASL | 0.923 |
| 3529 | **A** | 3526 | 3532 | DMCASLK | 0.938 |
| 3530 | **S** | 3527 | 3533 | MCASLKE | 0.959 |
| 3531 | **L** | 3528 | 3534 | CASLKEL | 0.983 |
| 3532 | **K** | 3529 | 3535 | ASLKELL | 1.001 |
| 3533 | **E** | 3530 | 3536 | SLKELLQ | 1.005 |
| 3534 | **L** | 3531 | 3537 | LKELLQN | 1.009 |
| 3535 | **L** | 3532 | 3538 | KELLQNG | 1.015 |
| 3536 | **Q** | 3533 | 3539 | ELLQNGM | 1.021 |
| 3537 | **N** | 3534 | 3540 | LLQNGMN | 1.029 |
| 3538 | **G** | 3535 | 3541 | LQNGMNG | 1.033 |
| 3539 | **M** | 3536 | 3542 | QNGMNGR | 1.032 |
| 3540 | **N** | 3537 | 3543 | NGMNGRT | 1.039 |
| 3541 | **G** | 3538 | 3544 | GMNGRTI | 1.039 |
| 3542 | **R** | 3539 | 3545 | MNGRTIL | 1.027 |
| 3543 | **T** | 3540 | 3546 | NGRTILG | 1.016 |
| 3544 | **I** | 3541 | 3547 | GRTILGS | 0.997 |
| 3545 | **L** | 3542 | 3548 | RTILGSA | 0.989 |
| 3546 | **G** | 3543 | 3549 | TILGSAL | 0.99 |
| 3547 | **S** | 3544 | 3550 | ILGSALL | 0.986 |
| 3548 | **A** | 3545 | 3551 | LGSALLE | 0.981 |
| 3549 | **L** | 3546 | 3552 | GSALLED | 0.978 |
| 3550 | **L** | 3547 | 3553 | SALLEDE | 0.985 |
| 3551 | **E** | 3548 | 3554 | ALLEDEF | 0.995 |
| 3552 | **D** | 3549 | 3555 | LLEDEFT | 1.006 |
| 3553 | **E** | 3550 | 3556 | LEDEFTP | 1.016 |
| 3554 | **F** | 3551 | 3557 | EDEFTPF | 1.011 |
| 3555 | **T** | 3552 | 3558 | DEFTPFD | 1.009 |
| 3556 | **P** | 3553 | 3559 | EFTPFDV | 0.998 |
| 3557 | **F** | 3554 | 3560 | FTPFDVV | 0.974 |
| 3558 | **D** | 3555 | 3561 | TPFDVVR | 0.963 |
| 3559 | **V** | 3556 | 3562 | PFDVVRQ | 0.957 |
| 3560 | **V** | 3557 | 3563 | FDVVRQC | 0.961 |
| 3561 | **R** | 3558 | 3564 | DVVRQCS | 0.983 |
| 3562 | **Q** | 3559 | 3565 | VVRQCSG | 1.0 |
| 3563 | **C** | 3560 | 3566 | VRQCSGV | 1.007 |
| 3564 | **S** | 3561 | 3567 | RQCSGVT | 1.011 |
| 3565 | **G** | 3562 | 3568 | QCSGVTF | 1.0 |
| 3566 | **V** | 3563 | 3569 | CSGVTFQ | 0.987 |
| 3567 | **T** | 3564 | 3570 | SGVTFQS | 0.982 |
| 3568 | **F** | 3565 | 3571 | GVTFQSA | 0.978 |
| 3569 | **Q** | 3566 | 3572 | VTFQSAV | 0.982 |
| 3570 | **S** | 3567 | 3573 | TFQSAVK | 0.99 |
| 3571 | **A** | 3568 | 3574 | FQSAVKR | 0.994 |
| 3572 | **V** | 3569 | 3575 | QSAVKRT | 1.003 |
| 3573 | **K** | 3570 | 3576 | SAVKRTI | 1.017 |
| 3574 | **R** | 3571 | 3577 | AVKRTIK | 1.027 |
| 3575 | **T** | 3572 | 3578 | VKRTIKG | 1.045 |
| 3576 | **I** | 3573 | 3579 | KRTIKGT | 1.056 |
| 3577 | **K** | 3574 | 3580 | RTIKGTH | 1.06 |
| 3578 | **G** | 3575 | 3581 | TIKGTHH | 1.051 |
| 3579 | **T** | 3576 | 3582 | IKGTHHW | 1.015 |
| 3580 | **H** | 3577 | 3583 | KGTHHWL | 0.971 |
| 3581 | **H** | 3578 | 3584 | GTHHWLL | 0.928 |
| 3582 | **W** | 3579 | 3585 | THHWLLL | 0.901 |
| 3583 | **L** | 3580 | 3586 | HHWLLLT | 0.903 |
| 3584 | **L** | 3581 | 3587 | HWLLLTI | 0.912 |
| 3585 | **L** | 3582 | 3588 | WLLLTIL | 0.925 |
| 3586 | **T** | 3583 | 3589 | LLLTILT | 0.939 |
| 3587 | **I** | 3584 | 3590 | LLTILTS | 0.953 |
| 3588 | **L** | 3585 | 3591 | LTILTSL | 0.972 |
| 3589 | **T** | 3586 | 3592 | TILTSLL | 0.987 |
| 3590 | **S** | 3587 | 3593 | ILTSLLV | 0.987 |
| 3591 | **L** | 3588 | 3594 | LTSLLVL | 0.973 |
| 3592 | **L** | 3589 | 3595 | TSLLVLV | 0.951 |
| 3593 | **V** | 3590 | 3596 | SLLVLVQ | 0.939 |
| 3594 | **L** | 3591 | 3597 | LLVLVQS | 0.952 |
| 3595 | **V** | 3592 | 3598 | LVLVQST | 0.982 |
| 3596 | **Q** | 3593 | 3599 | VLVQSTQ | 1.021 |
| 3597 | **S** | 3594 | 3600 | LVQSTQW | 1.046 |
| 3598 | **T** | 3595 | 3601 | VQSTQWS | 1.04 |
| 3599 | **Q** | 3596 | 3602 | QSTQWSL | 1.017 |
| 3600 | **W** | 3597 | 3603 | STQWSLF | 0.982 |
| 3601 | **S** | 3598 | 3604 | TQWSLFF | 0.951 |
| 3602 | **L** | 3599 | 3605 | QWSLFFF | 0.936 |
| 3603 | **F** | 3600 | 3606 | WSLFFFL | 0.925 |
| 3604 | **F** | 3601 | 3607 | SLFFFLY | 0.922 |
| 3605 | **F** | 3602 | 3608 | LFFFLYE | 0.928 |
| 3606 | **L** | 3603 | 3609 | FFFLYEN | 0.94 |
| 3607 | **Y** | 3604 | 3610 | FFLYENA | 0.956 |
| 3608 | **E** | 3605 | 3611 | FLYENAF | 0.97 |
| 3609 | **N** | 3606 | 3612 | LYENAFL | 0.971 |
| 3610 | **A** | 3607 | 3613 | YENAFLP | 0.963 |
| 3611 | **F** | 3608 | 3614 | ENAFLPF | 0.95 |
| 3612 | **L** | 3609 | 3615 | NAFLPFA | 0.937 |
| 3613 | **P** | 3610 | 3616 | AFLPFAM | 0.923 |
| 3614 | **F** | 3611 | 3617 | FLPFAMG | 0.913 |
| 3615 | **A** | 3612 | 3618 | LPFAMGI | 0.903 |
| 3616 | **M** | 3613 | 3619 | PFAMGII | 0.894 |
| 3617 | **G** | 3614 | 3620 | FAMGIIA | 0.893 |
| 3618 | **I** | 3615 | 3621 | AMGIIAM | 0.888 |
| 3619 | **I** | 3616 | 3622 | MGIIAMS | 0.887 |
| 3620 | **A** | 3617 | 3623 | GIIAMSA | 0.892 |
| 3621 | **M** | 3618 | 3624 | IIAMSAF | 0.896 |
| 3622 | **S** | 3619 | 3625 | IAMSAFA | 0.906 |
| 3623 | **A** | 3620 | 3626 | AMSAFAM | 0.906 |
| 3624 | **F** | 3621 | 3627 | MSAFAMM | 0.893 |
| 3625 | **A** | 3622 | 3628 | SAFAMMF | 0.879 |
| 3626 | **M** | 3623 | 3629 | AFAMMFV | 0.865 |
| 3627 | **M** | 3624 | 3630 | FAMMFVK | 0.874 |
| 3628 | **F** | 3625 | 3631 | AMMFVKH | 0.903 |
| 3629 | **V** | 3626 | 3632 | MMFVKHK | 0.941 |
| 3630 | **K** | 3627 | 3633 | MFVKHKH | 0.978 |
| 3631 | **H** | 3628 | 3634 | FVKHKHA | 0.99 |
| 3632 | **K** | 3629 | 3635 | VKHKHAF | 0.986 |
| 3633 | **H** | 3630 | 3636 | KHKHAFL | 0.964 |
| 3634 | **A** | 3631 | 3637 | HKHAFLC | 0.939 |
| 3635 | **F** | 3632 | 3638 | KHAFLCL | 0.927 |
| 3636 | **L** | 3633 | 3639 | HAFLCLF | 0.918 |
| 3637 | **C** | 3634 | 3640 | AFLCLFL | 0.918 |
| 3638 | **L** | 3635 | 3641 | FLCLFLL | 0.923 |
| 3639 | **F** | 3636 | 3642 | LCLFLLP | 0.935 |
| 3640 | **L** | 3637 | 3643 | CLFLLPS | 0.956 |
| 3641 | **L** | 3638 | 3644 | LFLLPSL | 0.979 |
| 3642 | **P** | 3639 | 3645 | FLLPSLA | 0.999 |
| 3643 | **S** | 3640 | 3646 | LLPSLAT | 1.0 |
| 3644 | **L** | 3641 | 3647 | LPSLATV | 0.985 |
| 3645 | **A** | 3642 | 3648 | PSLATVA | 0.962 |
| 3646 | **T** | 3643 | 3649 | SLATVAY | 0.934 |
| 3647 | **V** | 3644 | 3650 | LATVAYF | 0.914 |
| 3648 | **A** | 3645 | 3651 | ATVAYFN | 0.902 |
| 3649 | **Y** | 3646 | 3652 | TVAYFNM | 0.892 |
| 3650 | **F** | 3647 | 3653 | VAYFNMV | 0.894 |
| 3651 | **N** | 3648 | 3654 | AYFNMVY | 0.892 |
| 3652 | **M** | 3649 | 3655 | YFNMVYM | 0.886 |
| 3653 | **V** | 3650 | 3656 | FNMVYMP | 0.886 |
| 3654 | **Y** | 3651 | 3657 | NMVYMPA | 0.89 |
| 3655 | **M** | 3652 | 3658 | MVYMPAS | 0.903 |
| 3656 | **P** | 3653 | 3659 | VYMPASW | 0.924 |
| 3657 | **A** | 3654 | 3660 | YMPASWV | 0.94 |
| 3658 | **S** | 3655 | 3661 | MPASWVM | 0.936 |
| 3659 | **W** | 3656 | 3662 | PASWVMR | 0.926 |
| 3660 | **V** | 3657 | 3663 | ASWVMRI | 0.911 |
| 3661 | **M** | 3658 | 3664 | SWVMRIM | 0.893 |
| 3662 | **R** | 3659 | 3665 | WVMRIMT | 0.892 |
| 3663 | **I** | 3660 | 3666 | VMRIMTW | 0.892 |
| 3664 | **M** | 3661 | 3667 | MRIMTWL | 0.899 |
| 3665 | **T** | 3662 | 3668 | RIMTWLD | 0.913 |
| 3666 | **W** | 3663 | 3669 | IMTWLDM | 0.918 |
| 3667 | **L** | 3664 | 3670 | MTWLDMV | 0.923 |
| 3668 | **D** | 3665 | 3671 | TWLDMVD | 0.932 |
| 3669 | **M** | 3666 | 3672 | WLDMVDT | 0.945 |
| 3670 | **V** | 3667 | 3673 | LDMVDTS | 0.974 |
| 3671 | **D** | 3668 | 3674 | DMVDTSL | 1.005 |
| 3672 | **T** | 3669 | 3675 | MVDTSLS | 1.025 |
| 3673 | **S** | 3670 | 3676 | VDTSLSG | 1.035 |
| 3674 | **L** | 3671 | 3677 | DTSLSGF | 1.025 |
| 3675 | **S** | 3672 | 3678 | TSLSGFK | 1.019 |
| 3676 | **G** | 3673 | 3679 | SLSGFKL | 1.01 |
| 3677 | **F** | 3674 | 3680 | LSGFKLK | 1.006 |
| 3678 | **K** | 3675 | 3681 | SGFKLKD | 1.019 |
| 3679 | **L** | 3676 | 3682 | GFKLKDC | 1.015 |
| 3680 | **K** | 3677 | 3683 | FKLKDCV | 1.013 |
| 3681 | **D** | 3678 | 3684 | KLKDCVM | 0.991 |
| 3682 | **C** | 3679 | 3685 | LKDCVMY | 0.943 |
| 3683 | **V** | 3680 | 3686 | KDCVMYA | 0.911 |
| 3684 | **M** | 3681 | 3687 | DCVMYAS | 0.883 |
| 3685 | **Y** | 3682 | 3688 | CVMYASA | 0.881 |
| 3686 | **A** | 3683 | 3689 | VMYASAV | 0.899 |
| 3687 | **S** | 3684 | 3690 | MYASAVV | 0.912 |
| 3688 | **A** | 3685 | 3691 | YASAVVL | 0.923 |
| 3689 | **V** | 3686 | 3692 | ASAVVLL | 0.924 |
| 3690 | **V** | 3687 | 3693 | SAVVLLI | 0.918 |
| 3691 | **L** | 3688 | 3694 | AVVLLIL | 0.914 |
| 3692 | **L** | 3689 | 3695 | VVLLILM | 0.907 |
| 3693 | **I** | 3690 | 3696 | VLLILMT | 0.902 |
| 3694 | **L** | 3691 | 3697 | LLILMTA | 0.91 |
| 3695 | **M** | 3692 | 3698 | LILMTAR | 0.924 |
| 3696 | **T** | 3693 | 3699 | ILMTART | 0.954 |
| 3697 | **A** | 3694 | 3700 | LMTARTV | 0.983 |
| 3698 | **R** | 3695 | 3701 | MTARTVY | 0.994 |
| 3699 | **T** | 3696 | 3702 | TARTVYD | 1.002 |
| 3700 | **V** | 3697 | 3703 | ARTVYDD | 0.997 |
| 3701 | **Y** | 3698 | 3704 | RTVYDDG | 1.0 |
| 3702 | **D** | 3699 | 3705 | TVYDDGA | 1.017 |
| 3703 | **D** | 3700 | 3706 | VYDDGAR | 1.026 |
| 3704 | **G** | 3701 | 3707 | YDDGARR | 1.036 |
| 3705 | **A** | 3702 | 3708 | DDGARRV | 1.032 |
| 3706 | **R** | 3703 | 3709 | DGARRVW | 1.013 |
| 3707 | **R** | 3704 | 3710 | GARRVWT | 0.992 |
| 3708 | **V** | 3705 | 3711 | ARRVWTL | 0.967 |
| 3709 | **W** | 3706 | 3712 | RRVWTLM | 0.945 |
| 3710 | **T** | 3707 | 3713 | RVWTLMN | 0.932 |
| 3711 | **L** | 3708 | 3714 | VWTLMNV | 0.924 |
| 3712 | **M** | 3709 | 3715 | WTLMNVL | 0.92 |
| 3713 | **N** | 3710 | 3716 | TLMNVLT | 0.925 |
| 3714 | **V** | 3711 | 3717 | LMNVLTL | 0.931 |
| 3715 | **L** | 3712 | 3718 | MNVLTLV | 0.936 |
| 3716 | **T** | 3713 | 3719 | NVLTLVY | 0.94 |
| 3717 | **L** | 3714 | 3720 | VLTLVYK | 0.947 |
| 3718 | **V** | 3715 | 3721 | LTLVYKV | 0.95 |
| 3719 | **Y** | 3716 | 3722 | TLVYKVY | 0.949 |
| 3720 | **K** | 3717 | 3723 | LVYKVYY | 0.949 |
| 3721 | **V** | 3718 | 3724 | VYKVYYG | 0.942 |
| 3722 | **Y** | 3719 | 3725 | YKVYYGN | 0.941 |
| 3723 | **Y** | 3720 | 3726 | KVYYGNA | 0.952 |
| 3724 | **G** | 3721 | 3727 | VYYGNAL | 0.964 |
| 3725 | **N** | 3722 | 3728 | YYGNALD | 0.981 |
| 3726 | **A** | 3723 | 3729 | YGNALDQ | 0.994 |
| 3727 | **L** | 3724 | 3730 | GNALDQA | 1.0 |
| 3728 | **D** | 3725 | 3731 | NALDQAI | 1.0 |
| 3729 | **Q** | 3726 | 3732 | ALDQAIS | 0.987 |
| 3730 | **A** | 3727 | 3733 | LDQAISM | 0.962 |
| 3731 | **I** | 3728 | 3734 | DQAISMW | 0.928 |
| 3732 | **S** | 3729 | 3735 | QAISMWA | 0.898 |
| 3733 | **M** | 3730 | 3736 | AISMWAL | 0.879 |
| 3734 | **W** | 3731 | 3737 | ISMWALI | 0.87 |
| 3735 | **A** | 3732 | 3738 | SMWALII | 0.876 |
| 3736 | **L** | 3733 | 3739 | MWALIIS | 0.884 |
| 3737 | **I** | 3734 | 3740 | WALIISV | 0.896 |
| 3738 | **I** | 3735 | 3741 | ALIISVT | 0.919 |
| 3739 | **S** | 3736 | 3742 | LIISVTS | 0.953 |
| 3740 | **V** | 3737 | 3743 | IISVTSN | 0.991 |
| 3741 | **T** | 3738 | 3744 | ISVTSNY | 1.023 |
| 3742 | **S** | 3739 | 3745 | SVTSNYS | 1.044 |
| 3743 | **N** | 3740 | 3746 | VTSNYSG | 1.04 |
| 3744 | **Y** | 3741 | 3747 | TSNYSGV | 1.025 |
| 3745 | **S** | 3742 | 3748 | SNYSGVV | 1.01 |
| 3746 | **G** | 3743 | 3749 | NYSGVVT | 0.995 |
| 3747 | **V** | 3744 | 3750 | YSGVVTT | 0.989 |
| 3748 | **V** | 3745 | 3751 | SGVVTTV | 0.988 |
| 3749 | **T** | 3746 | 3752 | GVVTTVM | 0.981 |
| 3750 | **T** | 3747 | 3753 | VVTTVMF | 0.965 |
| 3751 | **V** | 3748 | 3754 | VTTVMFL | 0.941 |
| 3752 | **M** | 3749 | 3755 | TTVMFLA | 0.917 |
| 3753 | **F** | 3750 | 3756 | TVMFLAR | 0.916 |
| 3754 | **L** | 3751 | 3757 | VMFLARG | 0.931 |
| 3755 | **A** | 3752 | 3758 | MFLARGI | 0.952 |
| 3756 | **R** | 3753 | 3759 | FLARGIV | 0.971 |
| 3757 | **G** | 3754 | 3760 | LARGIVF | 0.968 |
| 3758 | **I** | 3755 | 3761 | ARGIVFM | 0.942 |
| 3759 | **V** | 3756 | 3762 | RGIVFMC | 0.92 |
| 3760 | **F** | 3757 | 3763 | GIVFMCV | 0.901 |
| 3761 | **M** | 3758 | 3764 | IVFMCVE | 0.89 |
| 3762 | **C** | 3759 | 3765 | VFMCVEY | 0.902 |
| 3763 | **V** | 3760 | 3766 | FMCVEYC | 0.909 |
| 3764 | **E** | 3761 | 3767 | MCVEYCP | 0.915 |
| 3765 | **Y** | 3762 | 3768 | CVEYCPI | 0.918 |
| 3766 | **C** | 3763 | 3769 | VEYCPIF | 0.912 |
| 3767 | **P** | 3764 | 3770 | EYCPIFF | 0.911 |
| 3768 | **I** | 3765 | 3771 | YCPIFFI | 0.907 |
| 3769 | **F** | 3766 | 3772 | CPIFFIT | 0.916 |
| 3770 | **F** | 3767 | 3773 | PIFFITG | 0.94 |
| 3771 | **I** | 3768 | 3774 | IFFITGN | 0.974 |
| 3772 | **T** | 3769 | 3775 | FFITGNT | 1.021 |
| 3773 | **G** | 3770 | 3776 | FITGNTL | 1.052 |
| 3774 | **N** | 3771 | 3777 | ITGNTLQ | 1.054 |
| 3775 | **T** | 3772 | 3778 | TGNTLQC | 1.027 |
| 3776 | **L** | 3773 | 3779 | GNTLQCI | 0.98 |
| 3777 | **Q** | 3774 | 3780 | NTLQCIM | 0.928 |
| 3778 | **C** | 3775 | 3781 | TLQCIML | 0.894 |
| 3779 | **I** | 3776 | 3782 | LQCIMLV | 0.877 |
| 3780 | **M** | 3777 | 3783 | QCIMLVY | 0.869 |
| 3781 | **L** | 3778 | 3784 | CIMLVYC | 0.879 |
| 3782 | **V** | 3779 | 3785 | IMLVYCF | 0.886 |
| 3783 | **Y** | 3780 | 3786 | MLVYCFL | 0.894 |
| 3784 | **C** | 3781 | 3787 | LVYCFLG | 0.909 |
| 3785 | **F** | 3782 | 3788 | VYCFLGY | 0.917 |
| 3786 | **L** | 3783 | 3789 | YCFLGYF | 0.926 |
| 3787 | **G** | 3784 | 3790 | CFLGYFC | 0.926 |
| 3788 | **Y** | 3785 | 3791 | FLGYFCT | 0.922 |
| 3789 | **F** | 3786 | 3792 | LGYFCTC | 0.915 |
| 3790 | **C** | 3787 | 3793 | GYFCTCY | 0.902 |
| 3791 | **T** | 3788 | 3794 | YFCTCYF | 0.897 |
| 3792 | **C** | 3789 | 3795 | FCTCYFG | 0.89 |
| 3793 | **Y** | 3790 | 3796 | CTCYFGL | 0.892 |
| 3794 | **F** | 3791 | 3797 | TCYFGLF | 0.903 |
| 3795 | **G** | 3792 | 3798 | CYFGLFC | 0.913 |
| 3796 | **L** | 3793 | 3799 | YFGLFCL | 0.924 |
| 3797 | **F** | 3794 | 3800 | FGLFCLL | 0.93 |
| 3798 | **C** | 3795 | 3801 | GLFCLLN | 0.936 |
| 3799 | **L** | 3796 | 3802 | LFCLLNR | 0.948 |
| 3800 | **L** | 3797 | 3803 | FCLLNRY | 0.961 |
| 3801 | **N** | 3798 | 3804 | CLLNRYF | 0.971 |
| 3802 | **R** | 3799 | 3805 | LLNRYFR | 0.968 |
| 3803 | **Y** | 3800 | 3806 | LNRYFRL | 0.955 |
| 3804 | **F** | 3801 | 3807 | NRYFRLT | 0.942 |
| 3805 | **R** | 3802 | 3808 | RYFRLTL | 0.935 |
| 3806 | **L** | 3803 | 3809 | YFRLTLG | 0.936 |
| 3807 | **T** | 3804 | 3810 | FRLTLGV | 0.939 |
| 3808 | **L** | 3805 | 3811 | RLTLGVY | 0.941 |
| 3809 | **G** | 3806 | 3812 | LTLGVYD | 0.938 |
| 3810 | **V** | 3807 | 3813 | TLGVYDY | 0.933 |
| 3811 | **Y** | 3808 | 3814 | LGVYDYL | 0.931 |
| 3812 | **D** | 3809 | 3815 | GVYDYLV | 0.928 |
| 3813 | **Y** | 3810 | 3816 | VYDYLVS | 0.936 |
| 3814 | **L** | 3811 | 3817 | YDYLVST | 0.951 |
| 3815 | **V** | 3812 | 3818 | DYLVSTQ | 0.982 |
| 3816 | **S** | 3813 | 3819 | YLVSTQE | 1.021 |
| 3817 | **T** | 3814 | 3820 | LVSTQEF | 1.044 |
| 3818 | **Q** | 3815 | 3821 | VSTQEFR | 1.048 |
| 3819 | **E** | 3816 | 3822 | STQEFRY | 1.022 |
| 3820 | **F** | 3817 | 3823 | TQEFRYM | 0.979 |
| 3821 | **R** | 3818 | 3824 | QEFRYMN | 0.947 |
| 3822 | **Y** | 3819 | 3825 | EFRYMNS | 0.943 |
| 3823 | **M** | 3820 | 3826 | FRYMNSQ | 0.968 |
| 3824 | **N** | 3821 | 3827 | RYMNSQG | 1.016 |
| 3825 | **S** | 3822 | 3828 | YMNSQGL | 1.055 |
| 3826 | **Q** | 3823 | 3829 | MNSQGLL | 1.066 |
| 3827 | **G** | 3824 | 3830 | NSQGLLP | 1.056 |
| 3828 | **L** | 3825 | 3831 | SQGLLPP | 1.036 |
| 3829 | **L** | 3826 | 3832 | QGLLPPK | 1.027 |
| 3830 | **P** | 3827 | 3833 | GLLPPKN | 1.041 |
| 3831 | **P** | 3828 | 3834 | LLPPKNS | 1.057 |
| 3832 | **K** | 3829 | 3835 | LPPKNSI | 1.07 |
| 3833 | **N** | 3830 | 3836 | PPKNSID | 1.064 |
| 3834 | **S** | 3831 | 3837 | PKNSIDA | 1.038 |
| 3835 | **I** | 3832 | 3838 | KNSIDAF | 1.005 |
| 3836 | **D** | 3833 | 3839 | NSIDAFK | 0.979 |
| 3837 | **A** | 3834 | 3840 | SIDAFKL | 0.966 |
| 3838 | **F** | 3835 | 3841 | IDAFKLN | 0.962 |
| 3839 | **K** | 3836 | 3842 | DAFKLNI | 0.972 |
| 3840 | **L** | 3837 | 3843 | AFKLNIK | 0.979 |
| 3841 | **N** | 3838 | 3844 | FKLNIKL | 0.983 |
| 3842 | **I** | 3839 | 3845 | KLNIKLL | 0.99 |
| 3843 | **K** | 3840 | 3846 | LNIKLLG | 0.987 |
| 3844 | **L** | 3841 | 3847 | NIKLLGV | 0.979 |
| 3845 | **L** | 3842 | 3848 | IKLLGVG | 0.976 |
| 3846 | **G** | 3843 | 3849 | KLLGVGG | 0.983 |
| 3847 | **V** | 3844 | 3850 | LLGVGGK | 1.005 |
| 3848 | **G** | 3845 | 3851 | LGVGGKP | 1.039 |
| 3849 | **G** | 3846 | 3852 | GVGGKPC | 1.057 |
| 3850 | **K** | 3847 | 3853 | VGGKPCI | 1.048 |
| 3851 | **P** | 3848 | 3854 | GGKPCIK | 1.026 |
| 3852 | **C** | 3849 | 3855 | GKPCIKV | 0.988 |
| 3853 | **I** | 3850 | 3856 | KPCIKVA | 0.965 |
| 3854 | **K** | 3851 | 3857 | PCIKVAT | 0.96 |
| 3855 | **V** | 3852 | 3858 | CIKVATV | 0.952 |
| 3856 | **A** | 3853 | 3859 | IKVATVQ | 0.96 |
| 3857 | **T** | 3854 | 3860 | KVATVQS | 0.979 |
| 3858 | **V** | 3855 | 3861 | VATVQSK | 1.003 |
| 3859 | **Q** | 3856 | 3862 | ATVQSKM | 1.031 |
| 3860 | **S** | 3857 | 3863 | TVQSKMS | 1.053 |
| 3861 | **K** | 3858 | 3864 | VQSKMSD | 1.056 |
| 3862 | **M** | 3859 | 3865 | QSKMSDV | 1.044 |
| 3863 | **S** | 3860 | 3866 | SKMSDVK | 1.041 |
| 3864 | **D** | 3861 | 3867 | KMSDVKC | 1.031 |
| 3865 | **V** | 3862 | 3868 | MSDVKCT | 1.024 |
| 3866 | **K** | 3863 | 3869 | SDVKCTS | 1.027 |
| 3867 | **C** | 3864 | 3870 | DVKCTSV | 1.015 |
| 3868 | **T** | 3865 | 3871 | VKCTSVV | 1.006 |
| 3869 | **S** | 3866 | 3872 | KCTSVVL | 0.991 |
| 3870 | **V** | 3867 | 3873 | CTSVVLL | 0.966 |
| 3871 | **V** | 3868 | 3874 | TSVVLLS | 0.949 |
| 3872 | **L** | 3869 | 3875 | SVVLLSV | 0.936 |
| 3873 | **L** | 3870 | 3876 | VVLLSVL | 0.934 |
| 3874 | **S** | 3871 | 3877 | VLLSVLQ | 0.941 |
| 3875 | **V** | 3872 | 3878 | LLSVLQQ | 0.955 |
| 3876 | **L** | 3873 | 3879 | LSVLQQL | 0.971 |
| 3877 | **Q** | 3874 | 3880 | SVLQQLR | 0.981 |
| 3878 | **Q** | 3875 | 3881 | VLQQLRV | 0.983 |
| 3879 | **L** | 3876 | 3882 | LQQLRVE | 0.98 |
| 3880 | **R** | 3877 | 3883 | QQLRVES | 0.986 |
| 3881 | **V** | 3878 | 3884 | QLRVESS | 1.013 |
| 3882 | **E** | 3879 | 3885 | LRVESSS | 1.055 |
| 3883 | **S** | 3880 | 3886 | RVESSSK | 1.099 |
| 3884 | **S** | 3881 | 3887 | VESSSKL | 1.117 |
| 3885 | **S** | 3882 | 3888 | ESSSKLW | 1.096 |
| 3886 | **K** | 3883 | 3889 | SSSKLWA | 1.049 |
| 3887 | **L** | 3884 | 3890 | SSKLWAQ | 0.986 |
| 3888 | **W** | 3885 | 3891 | SKLWAQC | 0.932 |
| 3889 | **A** | 3886 | 3892 | KLWAQCV | 0.908 |
| 3890 | **Q** | 3887 | 3893 | LWAQCVQ | 0.895 |
| 3891 | **C** | 3888 | 3894 | WAQCVQL | 0.898 |
| 3892 | **V** | 3889 | 3895 | AQCVQLH | 0.912 |
| 3893 | **Q** | 3890 | 3896 | QCVQLHN | 0.922 |
| 3894 | **L** | 3891 | 3897 | CVQLHND | 0.949 |
| 3895 | **H** | 3892 | 3898 | VQLHNDI | 0.967 |
| 3896 | **N** | 3893 | 3899 | QLHNDIL | 0.979 |
| 3897 | **D** | 3894 | 3900 | LHNDILL | 0.98 |
| 3898 | **I** | 3895 | 3901 | HNDILLA | 0.959 |
| 3899 | **L** | 3896 | 3902 | NDILLAK | 0.955 |
| 3900 | **L** | 3897 | 3903 | DILLAKD | 0.96 |
| 3901 | **A** | 3898 | 3904 | ILLAKDT | 0.979 |
| 3902 | **K** | 3899 | 3905 | LLAKDTT | 1.015 |
| 3903 | **D** | 3900 | 3906 | LAKDTTE | 1.037 |
| 3904 | **T** | 3901 | 3907 | AKDTTEA | 1.046 |
| 3905 | **T** | 3902 | 3908 | KDTTEAF | 1.036 |
| 3906 | **E** | 3903 | 3909 | DTTEAFE | 1.015 |
| 3907 | **A** | 3904 | 3910 | TTEAFEK | 1.0 |
| 3908 | **F** | 3905 | 3911 | TEAFEKM | 0.985 |
| 3909 | **E** | 3906 | 3912 | EAFEKMV | 0.982 |
| 3910 | **K** | 3907 | 3913 | AFEKMVS | 0.973 |
| 3911 | **M** | 3908 | 3914 | FEKMVSL | 0.954 |
| 3912 | **V** | 3909 | 3915 | EKMVSLL | 0.947 |
| 3913 | **S** | 3910 | 3916 | KMVSLLS | 0.938 |
| 3914 | **L** | 3911 | 3917 | MVSLLSV | 0.937 |
| 3915 | **L** | 3912 | 3918 | VSLLSVL | 0.94 |
| 3916 | **S** | 3913 | 3919 | SLLSVLL | 0.938 |
| 3917 | **V** | 3914 | 3920 | LLSVLLS | 0.935 |
| 3918 | **L** | 3915 | 3921 | LSVLLSM | 0.934 |
| 3919 | **L** | 3916 | 3922 | SVLLSMQ | 0.942 |
| 3920 | **S** | 3917 | 3923 | VLLSMQG | 0.955 |
| 3921 | **M** | 3918 | 3924 | LLSMQGA | 0.97 |
| 3922 | **Q** | 3919 | 3925 | LSMQGAV | 0.982 |
| 3923 | **G** | 3920 | 3926 | SMQGAVD | 0.981 |
| 3924 | **A** | 3921 | 3927 | MQGAVDI | 0.973 |
| 3925 | **V** | 3922 | 3928 | QGAVDIN | 0.967 |
| 3926 | **D** | 3923 | 3929 | GAVDINK | 0.971 |
| 3927 | **I** | 3924 | 3930 | AVDINKL | 0.984 |
| 3928 | **N** | 3925 | 3931 | VDINKLC | 0.992 |
| 3929 | **K** | 3926 | 3932 | DINKLCE | 0.997 |
| 3930 | **L** | 3927 | 3933 | INKLCEE | 0.99 |
| 3931 | **C** | 3928 | 3934 | NKLCEEM | 0.975 |
| 3932 | **E** | 3929 | 3935 | KLCEEML | 0.973 |
| 3933 | **E** | 3930 | 3936 | LCEEMLD | 0.973 |
| 3934 | **M** | 3931 | 3937 | CEEMLDN | 0.98 |
| 3935 | **L** | 3932 | 3938 | EEMLDNR | 1.004 |
| 3936 | **D** | 3933 | 3939 | EMLDNRA | 1.028 |
| 3937 | **N** | 3934 | 3940 | MLDNRAT | 1.039 |
| 3938 | **R** | 3935 | 3941 | LDNRATL | 1.035 |
| 3939 | **A** | 3936 | 3942 | DNRATLQ | 1.012 |
| 3940 | **T** | 3937 | 3943 | NRATLQA | 0.978 |
| 3941 | **L** | 3938 | 3944 | RATLQAI | 0.95 |
| 3942 | **Q** | 3939 | 3945 | ATLQAIA | 0.93 |
| 3943 | **A** | 3940 | 3946 | TLQAIAS | 0.929 |
| 3944 | **I** | 3941 | 3947 | LQAIASE | 0.94 |
| 3945 | **A** | 3942 | 3948 | QAIASEF | 0.958 |
| 3946 | **S** | 3943 | 3949 | AIASEFS | 0.984 |
| 3947 | **E** | 3944 | 3950 | IASEFSS | 0.999 |
| 3948 | **F** | 3945 | 3951 | ASEFSSL | 1.005 |
| 3949 | **S** | 3946 | 3952 | SEFSSLP | 1.017 |
| 3950 | **S** | 3947 | 3953 | EFSSLPS | 1.022 |
| 3951 | **L** | 3948 | 3954 | FSSLPSY | 1.02 |
| 3952 | **P** | 3949 | 3955 | SSLPSYA | 1.018 |
| 3953 | **S** | 3950 | 3956 | SLPSYAA | 0.993 |
| 3954 | **Y** | 3951 | 3957 | LPSYAAF | 0.961 |
| 3955 | **A** | 3952 | 3958 | PSYAAFA | 0.937 |
| 3956 | **A** | 3953 | 3959 | SYAAFAT | 0.92 |
| 3957 | **F** | 3954 | 3960 | YAAFATA | 0.925 |
| 3958 | **A** | 3955 | 3961 | AAFATAQ | 0.945 |
| 3959 | **T** | 3956 | 3962 | AFATAQE | 0.969 |
| 3960 | **A** | 3957 | 3963 | FATAQEA | 0.992 |
| 3961 | **Q** | 3958 | 3964 | ATAQEAY | 0.999 |
| 3962 | **E** | 3959 | 3965 | TAQEAYE | 0.999 |
| 3963 | **A** | 3960 | 3966 | AQEAYEQ | 0.992 |
| 3964 | **Y** | 3961 | 3967 | QEAYEQA | 0.986 |
| 3965 | **E** | 3962 | 3968 | EAYEQAV | 0.984 |
| 3966 | **Q** | 3963 | 3969 | AYEQAVA | 0.977 |
| 3967 | **A** | 3964 | 3970 | YEQAVAN | 0.969 |
| 3968 | **V** | 3965 | 3971 | EQAVANG | 0.973 |
| 3969 | **A** | 3966 | 3972 | QAVANGD | 0.986 |
| 3970 | **N** | 3967 | 3973 | AVANGDS | 1.018 |
| 3971 | **G** | 3968 | 3974 | VANGDSE | 1.054 |
| 3972 | **D** | 3969 | 3975 | ANGDSEV | 1.064 |
| 3973 | **S** | 3970 | 3976 | NGDSEVV | 1.059 |
| 3974 | **E** | 3971 | 3977 | GDSEVVL | 1.027 |
| 3975 | **V** | 3972 | 3978 | DSEVVLK | 0.996 |
| 3976 | **V** | 3973 | 3979 | SEVVLKK | 0.988 |
| 3977 | **L** | 3974 | 3980 | EVVLKKL | 0.991 |
| 3978 | **K** | 3975 | 3981 | VVLKKLK | 1.014 |
| 3979 | **K** | 3976 | 3982 | VLKKLKK | 1.035 |
| 3980 | **L** | 3977 | 3983 | LKKLKKS | 1.045 |
| 3981 | **K** | 3978 | 3984 | KKLKKSL | 1.051 |
| 3982 | **K** | 3979 | 3985 | KLKKSLN | 1.04 |
| 3983 | **S** | 3980 | 3986 | LKKSLNV | 1.018 |
| 3984 | **L** | 3981 | 3987 | KKSLNVA | 0.992 |
| 3985 | **N** | 3982 | 3988 | KSLNVAK | 0.973 |
| 3986 | **V** | 3983 | 3989 | SLNVAKS | 0.978 |
| 3987 | **A** | 3984 | 3990 | LNVAKSE | 1.001 |
| 3988 | **K** | 3985 | 3991 | NVAKSEF | 1.029 |
| 3989 | **S** | 3986 | 3992 | VAKSEFD | 1.05 |
| 3990 | **E** | 3987 | 3993 | AKSEFDR | 1.048 |
| 3991 | **F** | 3988 | 3994 | KSEFDRD | 1.042 |
| 3992 | **D** | 3989 | 3995 | SEFDRDA | 1.039 |
| 3993 | **R** | 3990 | 3996 | EFDRDAA | 1.023 |
| 3994 | **D** | 3991 | 3997 | FDRDAAM | 1.004 |
| 3995 | **A** | 3992 | 3998 | DRDAAMQ | 0.978 |
| 3996 | **A** | 3993 | 3999 | RDAAMQR | 0.956 |
| 3997 | **M** | 3994 | 4000 | DAAMQRK | 0.959 |
| 3998 | **Q** | 3995 | 4001 | AAMQRKL | 0.98 |
| 3999 | **R** | 3996 | 4002 | AMQRKLE | 1.004 |
| 4000 | **K** | 3997 | 4003 | MQRKLEK | 1.026 |
| 4001 | **L** | 3998 | 4004 | QRKLEKM | 1.022 |
| 4002 | **E** | 3999 | 4005 | RKLEKMA | 1.01 |
| 4003 | **K** | 4000 | 4006 | KLEKMAD | 1.001 |
| 4004 | **M** | 4001 | 4007 | LEKMADQ | 0.986 |
| 4005 | **A** | 4002 | 4008 | EKMADQA | 0.99 |
| 4006 | **D** | 4003 | 4009 | KMADQAM | 0.99 |
| 4007 | **Q** | 4004 | 4010 | MADQAMT | 0.984 |
| 4008 | **A** | 4005 | 4011 | ADQAMTQ | 0.982 |
| 4009 | **M** | 4006 | 4012 | DQAMTQM | 0.969 |
| 4010 | **T** | 4007 | 4013 | QAMTQMY | 0.966 |
| 4011 | **Q** | 4008 | 4014 | AMTQMYK | 0.966 |
| 4012 | **M** | 4009 | 4015 | MTQMYKQ | 0.967 |
| 4013 | **Y** | 4010 | 4016 | TQMYKQA | 0.985 |
| 4014 | **K** | 4011 | 4017 | QMYKQAR | 1.004 |
| 4015 | **Q** | 4012 | 4018 | MYKQARS | 1.027 |
| 4016 | **A** | 4013 | 4019 | YKQARSE | 1.054 |
| 4017 | **R** | 4014 | 4020 | KQARSED | 1.069 |
| 4018 | **S** | 4015 | 4021 | QARSEDK | 1.082 |
| 4019 | **E** | 4016 | 4022 | ARSEDKR | 1.081 |
| 4020 | **D** | 4017 | 4023 | RSEDKRA | 1.072 |
| 4021 | **K** | 4018 | 4024 | SEDKRAK | 1.066 |
| 4022 | **R** | 4019 | 4025 | EDKRAKV | 1.048 |
| 4023 | **A** | 4020 | 4026 | DKRAKVT | 1.041 |
| 4024 | **K** | 4021 | 4027 | KRAKVTS | 1.037 |
| 4025 | **V** | 4022 | 4028 | RAKVTSA | 1.025 |
| 4026 | **T** | 4023 | 4029 | AKVTSAM | 1.013 |
| 4027 | **S** | 4024 | 4030 | KVTSAMQ | 0.997 |
| 4028 | **A** | 4025 | 4031 | VTSAMQT | 0.982 |
| 4029 | **M** | 4026 | 4032 | TSAMQTM | 0.968 |
| 4030 | **Q** | 4027 | 4033 | SAMQTML | 0.965 |
| 4031 | **T** | 4028 | 4034 | AMQTMLF | 0.956 |
| 4032 | **M** | 4029 | 4035 | MQTMLFT | 0.94 |
| 4033 | **L** | 4030 | 4036 | QTMLFTM | 0.929 |
| 4034 | **F** | 4031 | 4037 | TMLFTML | 0.917 |
| 4035 | **T** | 4032 | 4038 | MLFTMLR | 0.92 |
| 4036 | **M** | 4033 | 4039 | LFTMLRK | 0.939 |
| 4037 | **L** | 4034 | 4040 | FTMLRKL | 0.964 |
| 4038 | **R** | 4035 | 4041 | TMLRKLD | 0.995 |
| 4039 | **K** | 4036 | 4042 | MLRKLDN | 1.025 |
| 4040 | **L** | 4037 | 4043 | LRKLDND | 1.045 |
| 4041 | **D** | 4038 | 4044 | RKLDNDA | 1.058 |
| 4042 | **N** | 4039 | 4045 | KLDNDAL | 1.054 |
| 4043 | **D** | 4040 | 4046 | LDNDALN | 1.039 |
| 4044 | **A** | 4041 | 4047 | DNDALNN | 1.017 |
| 4045 | **L** | 4042 | 4048 | NDALNNI | 0.994 |
| 4046 | **N** | 4043 | 4049 | DALNNII | 0.974 |
| 4047 | **N** | 4044 | 4050 | ALNNIIN | 0.961 |
| 4048 | **I** | 4045 | 4051 | LNNIINN | 0.953 |
| 4049 | **I** | 4046 | 4052 | NNIINNA | 0.958 |
| 4050 | **N** | 4047 | 4053 | NIINNAR | 0.976 |
| 4051 | **N** | 4048 | 4054 | IINNARD | 0.996 |
| 4052 | **A** | 4049 | 4055 | INNARDG | 1.017 |
| 4053 | **R** | 4050 | 4056 | NNARDGC | 1.02 |
| 4054 | **D** | 4051 | 4057 | NARDGCV | 1.007 |
| 4055 | **G** | 4052 | 4058 | ARDGCVP | 0.987 |
| 4056 | **C** | 4053 | 4059 | RDGCVPL | 0.959 |
| 4057 | **V** | 4054 | 4060 | DGCVPLN | 0.945 |
| 4058 | **P** | 4055 | 4061 | GCVPLNI | 0.935 |
| 4059 | **L** | 4056 | 4062 | CVPLNII | 0.929 |
| 4060 | **N** | 4057 | 4063 | VPLNIIP | 0.925 |
| 4061 | **I** | 4058 | 4064 | PLNIIPL | 0.921 |
| 4062 | **I** | 4059 | 4065 | LNIIPLT | 0.928 |
| 4063 | **P** | 4060 | 4066 | NIIPLTT | 0.948 |
| 4064 | **L** | 4061 | 4067 | IIPLTTA | 0.971 |
| 4065 | **T** | 4062 | 4068 | IPLTTAA | 0.991 |
| 4066 | **T** | 4063 | 4069 | PLTTAAK | 1.001 |
| 4067 | **A** | 4064 | 4070 | LTTAAKL | 0.995 |
| 4068 | **A** | 4065 | 4071 | TTAAKLM | 0.979 |
| 4069 | **K** | 4066 | 4072 | TAAKLMV | 0.963 |
| 4070 | **L** | 4067 | 4073 | AAKLMVV | 0.938 |
| 4071 | **M** | 4068 | 4074 | AKLMVVI | 0.913 |
| 4072 | **V** | 4069 | 4075 | KLMVVIP | 0.916 |
| 4073 | **V** | 4070 | 4076 | LMVVIPD | 0.931 |
| 4074 | **I** | 4071 | 4077 | MVVIPDY | 0.958 |
| 4075 | **P** | 4072 | 4078 | VVIPDYN | 0.997 |
| 4076 | **D** | 4073 | 4079 | VIPDYNT | 1.016 |
| 4077 | **Y** | 4074 | 4080 | IPDYNTY | 1.016 |
| 4078 | **N** | 4075 | 4081 | PDYNTYK | 1.02 |
| 4079 | **T** | 4076 | 4082 | DYNTYKN | 1.025 |
| 4080 | **Y** | 4077 | 4083 | YNTYKNT | 1.031 |
| 4081 | **K** | 4078 | 4084 | NTYKNTC | 1.046 |
| 4082 | **N** | 4079 | 4085 | TYKNTCD | 1.053 |
| 4083 | **T** | 4080 | 4086 | YKNTCDG | 1.055 |
| 4084 | **C** | 4081 | 4087 | KNTCDGT | 1.058 |
| 4085 | **D** | 4082 | 4088 | NTCDGTT | 1.067 |
| 4086 | **G** | 4083 | 4089 | TCDGTTF | 1.066 |
| 4087 | **T** | 4084 | 4090 | CDGTTFT | 1.05 |
| 4088 | **T** | 4085 | 4091 | DGTTFTY | 1.024 |
| 4089 | **F** | 4086 | 4092 | GTTFTYA | 0.986 |
| 4090 | **T** | 4087 | 4093 | TTFTYAS | 0.957 |
| 4091 | **Y** | 4088 | 4094 | TFTYASA | 0.941 |
| 4092 | **A** | 4089 | 4095 | FTYASAL | 0.935 |
| 4093 | **S** | 4090 | 4096 | TYASALW | 0.933 |
| 4094 | **A** | 4091 | 4097 | YASALWE | 0.932 |
| 4095 | **L** | 4092 | 4098 | ASALWEI | 0.933 |
| 4096 | **W** | 4093 | 4099 | SALWEIQ | 0.942 |
| 4097 | **E** | 4094 | 4100 | ALWEIQQ | 0.96 |
| 4098 | **I** | 4095 | 4101 | LWEIQQV | 0.977 |
| 4099 | **Q** | 4096 | 4102 | WEIQQVV | 0.985 |
| 4100 | **Q** | 4097 | 4103 | EIQQVVD | 0.981 |
| 4101 | **V** | 4098 | 4104 | IQQVVDA | 0.971 |
| 4102 | **V** | 4099 | 4105 | QQVVDAD | 0.971 |
| 4103 | **D** | 4100 | 4106 | QVVDADS | 0.991 |
| 4104 | **A** | 4101 | 4107 | VVDADSK | 1.027 |
| 4105 | **D** | 4102 | 4108 | VDADSKI | 1.052 |
| 4106 | **S** | 4103 | 4109 | DADSKIV | 1.057 |
| 4107 | **K** | 4104 | 4110 | ADSKIVQ | 1.029 |
| 4108 | **I** | 4105 | 4111 | DSKIVQL | 0.985 |
| 4109 | **V** | 4106 | 4112 | SKIVQLS | 0.96 |
| 4110 | **Q** | 4107 | 4113 | KIVQLSE | 0.951 |
| 4111 | **L** | 4108 | 4114 | IVQLSEI | 0.968 |
| 4112 | **S** | 4109 | 4115 | VQLSEIS | 0.989 |
| 4113 | **E** | 4110 | 4116 | QLSEISM | 0.994 |
| 4114 | **I** | 4111 | 4117 | LSEISMD | 0.996 |
| 4115 | **S** | 4112 | 4118 | SEISMDN | 0.997 |
| 4116 | **M** | 4113 | 4119 | EISMDNS | 1.017 |
| 4117 | **D** | 4114 | 4120 | ISMDNSP | 1.049 |
| 4118 | **N** | 4115 | 4121 | SMDNSPN | 1.073 |
| 4119 | **S** | 4116 | 4122 | MDNSPNL | 1.081 |
| 4120 | **P** | 4117 | 4123 | DNSPNLA | 1.055 |
| 4121 | **N** | 4118 | 4124 | NSPNLAW | 1.014 |
| 4122 | **L** | 4119 | 4125 | SPNLAWP | 0.974 |
| 4123 | **A** | 4120 | 4126 | PNLAWPL | 0.943 |
| 4124 | **W** | 4121 | 4127 | NLAWPLI | 0.929 |
| 4125 | **P** | 4122 | 4128 | LAWPLIV | 0.924 |
| 4126 | **L** | 4123 | 4129 | AWPLIVT | 0.923 |
| 4127 | **I** | 4124 | 4130 | WPLIVTA | 0.922 |
| 4128 | **V** | 4125 | 4131 | PLIVTAL | 0.927 |
| 4129 | **T** | 4126 | 4132 | LIVTALR | 0.93 |
| 4130 | **A** | 4127 | 4133 | IVTALRA | 0.94 |
| 4131 | **L** | 4128 | 4134 | VTALRAN | 0.954 |
| 4132 | **R** | 4129 | 4135 | TALRANS | 0.968 |
| 4133 | **A** | 4130 | 4136 | ALRANSA | 0.987 |
| 4134 | **N** | 4131 | 4137 | LRANSAV | 0.992 |
| 4135 | **S** | 4132 | 4138 | RANSAVK | 0.996 |
| 4136 | **A** | 4133 | 4139 | ANSAVKL | 0.99 |
| 4137 | **V** | 4134 | 4140 | NSAVKLQ | 0.986 |
| 4138 | **K** | 4135 | 4141 | SAVKLQN | 1.001 |
| 4139 | **L** | 4136 | 4142 | AVKLQNN | 1.017 |
| 4140 | **Q** | 4137 | 4143 | VKLQNNE | 1.042 |
| 4141 | **N** | 4138 | 4144 | KLQNNEL | 1.058 |
| 4142 | **N** | 4139 | 4145 | LQNNELS | 1.058 |
| 4143 | **E** | 4140 | 4146 | QNNELSP | 1.052 |
| 4144 | **L** | 4141 | 4147 | NNELSPV | 1.036 |
| 4145 | **S** | 4142 | 4148 | NELSPVA | 1.018 |
| 4146 | **P** | 4143 | 4149 | ELSPVAL | 0.999 |
| 4147 | **V** | 4144 | 4150 | LSPVALR | 0.978 |
| 4148 | **A** | 4145 | 4151 | SPVALRQ | 0.971 |
| 4149 | **L** | 4146 | 4152 | PVALRQM | 0.972 |
| 4150 | **R** | 4147 | 4153 | VALRQMS | 0.975 |
| 4151 | **Q** | 4148 | 4154 | ALRQMSC | 0.973 |
| 4152 | **M** | 4149 | 4155 | LRQMSCA | 0.957 |
| 4153 | **S** | 4150 | 4156 | RQMSCAA | 0.936 |
| 4154 | **C** | 4151 | 4157 | QMSCAAG | 0.926 |
| 4155 | **A** | 4152 | 4158 | MSCAAGT | 0.937 |
| 4156 | **A** | 4153 | 4159 | SCAAGTT | 0.968 |
| 4157 | **G** | 4154 | 4160 | CAAGTTQ | 1.012 |
| 4158 | **T** | 4155 | 4161 | AAGTTQT | 1.05 |
| 4159 | **T** | 4156 | 4162 | AGTTQTA | 1.068 |
| 4160 | **Q** | 4157 | 4163 | GTTQTAC | 1.062 |
| 4161 | **T** | 4158 | 4164 | TTQTACT | 1.035 |
| 4162 | **A** | 4159 | 4165 | TQTACTD | 1.007 |
| 4163 | **C** | 4160 | 4166 | QTACTDD | 0.992 |
| 4164 | **T** | 4161 | 4167 | TACTDDN | 0.997 |
| 4165 | **D** | 4162 | 4168 | ACTDDNA | 1.003 |
| 4166 | **D** | 4163 | 4169 | CTDDNAL | 1.003 |
| 4167 | **N** | 4164 | 4170 | TDDNALA | 0.988 |
| 4168 | **A** | 4165 | 4171 | DDNALAY | 0.955 |
| 4169 | **L** | 4166 | 4172 | DNALAYY | 0.928 |
| 4170 | **A** | 4167 | 4173 | NALAYYN | 0.913 |
| 4171 | **Y** | 4168 | 4174 | ALAYYNT | 0.918 |
| 4172 | **Y** | 4169 | 4175 | LAYYNTT | 0.948 |
| 4173 | **N** | 4170 | 4176 | AYYNTTK | 0.99 |
| 4174 | **T** | 4171 | 4177 | YYNTTKG | 1.035 |
| 4175 | **T** | 4172 | 4178 | YNTTKGG | 1.072 |
| 4176 | **K** | 4173 | 4179 | NTTKGGR | 1.093 |
| 4177 | **G** | 4174 | 4180 | TTKGGRF | 1.091 |
| 4178 | **G** | 4175 | 4181 | TKGGRFV | 1.067 |
| 4179 | **R** | 4176 | 4182 | KGGRFVL | 1.025 |
| 4180 | **F** | 4177 | 4183 | GGRFVLA | 0.977 |
| 4181 | **V** | 4178 | 4184 | GRFVLAL | 0.941 |
| 4182 | **L** | 4179 | 4185 | RFVLALL | 0.922 |
| 4183 | **A** | 4180 | 4186 | FVLALLS | 0.925 |
| 4184 | **L** | 4181 | 4187 | VLALLSD | 0.949 |
| 4185 | **L** | 4182 | 4188 | LALLSDL | 0.975 |
| 4186 | **S** | 4183 | 4189 | ALLSDLQ | 1.002 |
| 4187 | **D** | 4184 | 4190 | LLSDLQD | 1.024 |
| 4188 | **L** | 4185 | 4191 | LSDLQDL | 1.026 |
| 4189 | **Q** | 4186 | 4192 | SDLQDLK | 1.031 |
| 4190 | **D** | 4187 | 4193 | DLQDLKW | 1.025 |
| 4191 | **L** | 4188 | 4194 | LQDLKWA | 1.007 |
| 4192 | **K** | 4189 | 4195 | QDLKWAR | 0.992 |
| 4193 | **W** | 4190 | 4196 | DLKWARF | 0.964 |
| 4194 | **A** | 4191 | 4197 | LKWARFP | 0.954 |
| 4195 | **R** | 4192 | 4198 | KWARFPK | 0.962 |
| 4196 | **F** | 4193 | 4199 | WARFPKS | 0.99 |
| 4197 | **P** | 4194 | 4200 | ARFPKSD | 1.033 |
| 4198 | **K** | 4195 | 4201 | RFPKSDG | 1.069 |
| 4199 | **S** | 4196 | 4202 | FPKSDGT | 1.094 |
| 4200 | **D** | 4197 | 4203 | PKSDGTG | 1.101 |
| 4201 | **G** | 4198 | 4204 | KSDGTGT | 1.103 |
| 4202 | **T** | 4199 | 4205 | SDGTGTI | 1.086 |
| 4203 | **G** | 4200 | 4206 | DGTGTIY | 1.061 |
| 4204 | **T** | 4201 | 4207 | GTGTIYT | 1.032 |
| 4205 | **I** | 4202 | 4208 | TGTIYTE | 1.0 |
| 4206 | **Y** | 4203 | 4209 | GTIYTEL | 0.989 |
| 4207 | **T** | 4204 | 4210 | TIYTELE | 0.994 |
| 4208 | **E** | 4205 | 4211 | IYTELEP | 1.005 |
| 4209 | **L** | 4206 | 4212 | YTELEPP | 1.019 |
| 4210 | **E** | 4207 | 4213 | TELEPPC | 1.029 |
| 4211 | **P** | 4208 | 4214 | ELEPPCR | 1.023 |
| 4212 | **P** | 4209 | 4215 | LEPPCRF | 1.009 |
| 4213 | **C** | 4210 | 4216 | EPPCRFV | 0.981 |
| 4214 | **R** | 4211 | 4217 | PPCRFVT | 0.959 |
| 4215 | **F** | 4212 | 4218 | PCRFVTD | 0.955 |
| 4216 | **V** | 4213 | 4219 | CRFVTDT | 0.969 |
| 4217 | **T** | 4214 | 4220 | RFVTDTP | 1.001 |
| 4218 | **D** | 4215 | 4221 | FVTDTPK | 1.03 |
| 4219 | **T** | 4216 | 4222 | VTDTPKG | 1.057 |
| 4220 | **P** | 4217 | 4223 | TDTPKGP | 1.073 |
| 4221 | **K** | 4218 | 4224 | DTPKGPK | 1.083 |
| 4222 | **G** | 4219 | 4225 | TPKGPKV | 1.084 |
| 4223 | **P** | 4220 | 4226 | PKGPKVK | 1.072 |
| 4224 | **K** | 4221 | 4227 | KGPKVKY | 1.054 |
| 4225 | **V** | 4222 | 4228 | GPKVKYL | 1.024 |
| 4226 | **K** | 4223 | 4229 | PKVKYLY | 0.991 |
| 4227 | **Y** | 4224 | 4230 | KVKYLYF | 0.956 |
| 4228 | **L** | 4225 | 4231 | VKYLYFI | 0.925 |
| 4229 | **Y** | 4226 | 4232 | KYLYFIK | 0.915 |
| 4230 | **F** | 4227 | 4233 | YLYFIKG | 0.926 |
| 4231 | **I** | 4228 | 4234 | LYFIKGL | 0.95 |
| 4232 | **K** | 4229 | 4235 | YFIKGLN | 0.983 |
| 4233 | **G** | 4230 | 4236 | FIKGLNN | 1.002 |
| 4234 | **L** | 4231 | 4237 | IKGLNNL | 1.003 |
| 4235 | **N** | 4232 | 4238 | KGLNNLN | 1.003 |
| 4236 | **N** | 4233 | 4239 | GLNNLNR | 0.998 |
| 4237 | **L** | 4234 | 4240 | LNNLNRG | 1.0 |
| 4238 | **N** | 4235 | 4241 | NNLNRGM | 1.0 |
| 4239 | **R** | 4236 | 4242 | NLNRGMV | 0.992 |
| 4240 | **G** | 4237 | 4243 | LNRGMVL | 0.977 |
| 4241 | **M** | 4238 | 4244 | NRGMVLG | 0.96 |
| 4242 | **V** | 4239 | 4245 | RGMVLGS | 0.961 |
| 4243 | **L** | 4240 | 4246 | GMVLGSL | 0.971 |
| 4244 | **G** | 4241 | 4247 | MVLGSLA | 0.981 |
| 4245 | **S** | 4242 | 4248 | VLGSLAA | 0.985 |
| 4246 | **L** | 4243 | 4249 | LGSLAAT | 0.971 |
| 4247 | **A** | 4244 | 4250 | GSLAATV | 0.955 |
| 4248 | **A** | 4245 | 4251 | SLAATVR | 0.944 |
| 4249 | **T** | 4246 | 4252 | LAATVRL | 0.94 |
| 4250 | **V** | 4247 | 4253 | AATVRLQ | 0.94 |
| 4251 | **R** | 4248 | 4254 | ATVRLQA | 0.942 |
| 4252 | **L** | 4249 | 4255 | TVRLQAG | 0.953 |
| 4253 | **Q** | 4250 | 4256 | VRLQAGN | 0.965 |
| 4254 | **A** | 4251 | 4257 | RLQAGNA | 0.99 |
| 4255 | **G** | 4252 | 4258 | LQAGNAT | 1.011 |
| 4256 | **N** | 4253 | 4259 | QAGNATE | 1.024 |
| 4257 | **A** | 4254 | 4260 | AGNATEV | 1.032 |
| 4258 | **T** | 4255 | 4261 | GNATEVP | 1.024 |
| 4259 | **E** | 4256 | 4262 | NATEVPA | 1.015 |
| 4260 | **V** | 4257 | 4263 | ATEVPAN | 1.004 |
| 4261 | **P** | 4258 | 4264 | TEVPANS | 1.006 |
| 4262 | **A** | 4259 | 4265 | EVPANST | 1.023 |
| 4263 | **N** | 4260 | 4266 | VPANSTV | 1.033 |
| 4264 | **S** | 4261 | 4267 | PANSTVL | 1.042 |
| 4265 | **T** | 4262 | 4268 | ANSTVLS | 1.024 |
| 4266 | **V** | 4263 | 4269 | NSTVLSF | 0.99 |
| 4267 | **L** | 4264 | 4270 | STVLSFC | 0.963 |
| 4268 | **S** | 4265 | 4271 | TVLSFCA | 0.935 |
| 4269 | **F** | 4266 | 4272 | VLSFCAF | 0.921 |
| 4270 | **C** | 4267 | 4273 | LSFCAFA | 0.915 |
| 4271 | **A** | 4268 | 4274 | SFCAFAV | 0.909 |
| 4272 | **F** | 4269 | 4275 | FCAFAVD | 0.91 |
| 4273 | **A** | 4270 | 4276 | CAFAVDA | 0.913 |
| 4274 | **V** | 4271 | 4277 | AFAVDAA | 0.921 |
| 4275 | **D** | 4272 | 4278 | FAVDAAK | 0.937 |
| 4276 | **A** | 4273 | 4279 | AVDAAKA | 0.952 |
| 4277 | **A** | 4274 | 4280 | VDAAKAY | 0.963 |
| 4278 | **K** | 4275 | 4281 | DAAKAYK | 0.979 |
| 4279 | **A** | 4276 | 4282 | AAKAYKD | 0.99 |
| 4280 | **Y** | 4277 | 4283 | AKAYKDY | 0.998 |
| 4281 | **K** | 4278 | 4284 | KAYKDYL | 1.007 |
| 4282 | **D** | 4279 | 4285 | AYKDYLA | 0.999 |
| 4283 | **Y** | 4280 | 4286 | YKDYLAS | 0.987 |
| 4284 | **L** | 4281 | 4287 | KDYLASG | 0.988 |
| 4285 | **A** | 4282 | 4288 | DYLASGG | 1.004 |
| 4286 | **S** | 4283 | 4289 | YLASGGQ | 1.042 |
| 4287 | **G** | 4284 | 4290 | LASGGQP | 1.085 |
| 4288 | **G** | 4285 | 4291 | ASGGQPI | 1.106 |
| 4289 | **Q** | 4286 | 4292 | SGGQPIT | 1.108 |
| 4290 | **P** | 4287 | 4293 | GGQPITN | 1.086 |
| 4291 | **I** | 4288 | 4294 | GQPITNC | 1.049 |
| 4292 | **T** | 4289 | 4295 | QPITNCV | 1.015 |
| 4293 | **N** | 4290 | 4296 | PITNCVK | 0.988 |
| 4294 | **C** | 4291 | 4297 | ITNCVKM | 0.962 |
| 4295 | **V** | 4292 | 4298 | TNCVKML | 0.951 |
| 4296 | **K** | 4293 | 4299 | NCVKMLC | 0.942 |
| 4297 | **M** | 4294 | 4300 | CVKMLCT | 0.925 |
| 4298 | **L** | 4295 | 4301 | VKMLCTH | 0.925 |
| 4299 | **C** | 4296 | 4302 | KMLCTHT | 0.93 |
| 4300 | **T** | 4297 | 4303 | MLCTHTG | 0.954 |
| 4301 | **H** | 4298 | 4304 | LCTHTGT | 0.995 |
| 4302 | **T** | 4299 | 4305 | CTHTGTG | 1.038 |
| 4303 | **G** | 4300 | 4306 | THTGTGQ | 1.072 |
| 4304 | **T** | 4301 | 4307 | HTGTGQA | 1.077 |
| 4305 | **G** | 4302 | 4308 | TGTGQAI | 1.062 |
| 4306 | **Q** | 4303 | 4309 | GTGQAIT | 1.024 |
| 4307 | **A** | 4304 | 4310 | TGQAITV | 0.984 |
| 4308 | **I** | 4305 | 4311 | GQAITVT | 0.964 |
| 4309 | **T** | 4306 | 4312 | QAITVTP | 0.965 |
| 4310 | **V** | 4307 | 4313 | AITVTPE | 0.985 |
| 4311 | **T** | 4308 | 4314 | ITVTPEA | 1.012 |
| 4312 | **P** | 4309 | 4315 | TVTPEAN | 1.024 |
| 4313 | **E** | 4310 | 4316 | VTPEANM | 1.02 |
| 4314 | **A** | 4311 | 4317 | TPEANMD | 1.013 |
| 4315 | **N** | 4312 | 4318 | PEANMDQ | 1.01 |
| 4316 | **M** | 4313 | 4319 | EANMDQE | 1.024 |
| 4317 | **D** | 4314 | 4320 | ANMDQES | 1.052 |
| 4318 | **Q** | 4315 | 4321 | NMDQESF | 1.066 |
| 4319 | **E** | 4316 | 4322 | MDQESFG | 1.065 |
| 4320 | **S** | 4317 | 4323 | DQESFGG | 1.051 |
| 4321 | **F** | 4318 | 4324 | QESFGGA | 1.029 |
| 4322 | **G** | 4319 | 4325 | ESFGGAS | 1.017 |
| 4323 | **G** | 4320 | 4326 | SFGGASC | 1.003 |
| 4324 | **A** | 4321 | 4327 | FGGASCC | 0.985 |
| 4325 | **S** | 4322 | 4328 | GGASCCL | 0.959 |
| 4326 | **C** | 4323 | 4329 | GASCCLY | 0.929 |
| 4327 | **C** | 4324 | 4330 | ASCCLYC | 0.908 |
| 4328 | **L** | 4325 | 4331 | SCCLYCR | 0.894 |
| 4329 | **Y** | 4326 | 4332 | CCLYCRC | 0.885 |
| 4330 | **C** | 4327 | 4333 | CLYCRCH | 0.884 |
| 4331 | **R** | 4328 | 4334 | LYCRCHI | 0.884 |
| 4332 | **C** | 4329 | 4335 | YCRCHID | 0.888 |
| 4333 | **H** | 4330 | 4336 | CRCHIDH | 0.901 |
| 4334 | **I** | 4331 | 4337 | RCHIDHP | 0.922 |
| 4335 | **D** | 4332 | 4338 | CHIDHPN | 0.956 |
| 4336 | **H** | 4333 | 4339 | HIDHPNP | 0.997 |
| 4337 | **P** | 4334 | 4340 | IDHPNPK | 1.037 |
| 4338 | **N** | 4335 | 4341 | DHPNPKG | 1.063 |
| 4339 | **P** | 4336 | 4342 | HPNPKGF | 1.062 |
| 4340 | **K** | 4337 | 4343 | PNPKGFC | 1.043 |
| 4341 | **G** | 4338 | 4344 | NPKGFCD | 1.006 |
| 4342 | **F** | 4339 | 4345 | PKGFCDL | 0.968 |
| 4343 | **C** | 4340 | 4346 | KGFCDLK | 0.952 |
| 4344 | **D** | 4341 | 4347 | GFCDLKG | 0.965 |
| 4345 | **L** | 4342 | 4348 | FCDLKGK | 0.997 |
| 4346 | **K** | 4343 | 4349 | CDLKGKY | 1.031 |
| 4347 | **G** | 4344 | 4350 | DLKGKYV | 1.045 |
| 4348 | **K** | 4345 | 4351 | LKGKYVQ | 1.026 |
| 4349 | **Y** | 4346 | 4352 | KGKYVQI | 0.993 |
| 4350 | **V** | 4347 | 4353 | GKYVQIP | 0.972 |
| 4351 | **Q** | 4348 | 4354 | KYVQIPT | 0.97 |
| 4352 | **I** | 4349 | 4355 | YVQIPTT | 0.994 |
| 4353 | **P** | 4350 | 4356 | VQIPTTC | 1.015 |
| 4354 | **T** | 4351 | 4357 | QIPTTCA | 1.018 |
| 4355 | **T** | 4352 | 4358 | IPTTCAN | 1.008 |
| 4356 | **C** | 4353 | 4359 | PTTCAND | 0.986 |
| 4357 | **A** | 4354 | 4360 | TTCANDP | 0.985 |
| 4358 | **N** | 4355 | 4361 | TCANDPV | 0.995 |
| 4359 | **D** | 4356 | 4362 | CANDPVG | 1.004 |
| 4360 | **P** | 4357 | 4363 | ANDPVGF | 1.007 |
| 4361 | **V** | 4358 | 4364 | NDPVGFT | 0.989 |
| 4362 | **G** | 4359 | 4365 | DPVGFTL | 0.967 |
| 4363 | **F** | 4360 | 4366 | PVGFTLK | 0.96 |
| 4364 | **T** | 4361 | 4367 | VGFTLKN | 0.971 |
| 4365 | **L** | 4362 | 4368 | GFTLKNT | 0.999 |
| 4366 | **K** | 4363 | 4369 | FTLKNTV | 1.025 |
| 4367 | **N** | 4364 | 4370 | TLKNTVC | 1.029 |
| 4368 | **T** | 4365 | 4371 | LKNTVCT | 1.01 |
| 4369 | **V** | 4366 | 4372 | KNTVCTV | 0.975 |
| 4370 | **C** | 4367 | 4373 | NTVCTVC | 0.94 |
| 4371 | **T** | 4368 | 4374 | TVCTVCG | 0.921 |
| 4372 | **V** | 4369 | 4375 | VCTVCGM | 0.908 |
| 4373 | **C** | 4370 | 4376 | CTVCGMW | 0.903 |
| 4374 | **G** | 4371 | 4377 | TVCGMWK | 0.914 |
| 4375 | **M** | 4372 | 4378 | VCGMWKG | 0.929 |
| 4376 | **W** | 4373 | 4379 | CGMWKGY | 0.954 |
| 4377 | **K** | 4374 | 4380 | GMWKGYG | 0.981 |
| 4378 | **G** | 4375 | 4381 | MWKGYGC | 0.987 |
| 4379 | **Y** | 4376 | 4382 | WKGYGCS | 0.979 |
| 4380 | **G** | 4377 | 4383 | KGYGCSC | 0.964 |
| 4381 | **C** | 4378 | 4384 | GYGCSCD | 0.959 |
| 4382 | **S** | 4379 | 4385 | YGCSCDQ | 0.967 |
| 4383 | **C** | 4380 | 4386 | GCSCDQL | 0.984 |
| 4384 | **D** | 4381 | 4387 | CSCDQLR | 1.005 |
| 4385 | **Q** | 4382 | 4388 | SCDQLRE | 1.017 |
| 4386 | **L** | 4383 | 4389 | CDQLREP | 1.028 |
| 4387 | **R** | 4384 | 4390 | DQLREPM | 1.029 |
| 4388 | **E** | 4385 | 4391 | QLREPML | 1.023 |
| 4389 | **P** | 4386 | 4392 | LREPMLQ | 1.011 |
| 4390 | **M** | 4387 | 4393 | REPMLQS | 0.994 |
| 4391 | **L** | 4388 | 4394 | EPMLQSA | 0.995 |
| 4392 | **Q** | 4389 | 4395 | PMLQSAD | 0.998 |
| 4393 | **S** | 4390 | 4396 | MLQSADA | 1.006 |
| 4394 | **A** | 4391 | 4397 | LQSADAQ | 1.014 |
| 4395 | **D** | 4392 | 4398 | QSADAQS | 1.013 |
| 4396 | **A** | 4393 | 4399 | SADAQSF | 1.011 |
| 4397 | **Q** | 4394 | 4400 | ADAQSFL | 1.004 |
| 4398 | **S** | 4395 | 4401 | DAQSFLN | 0.997 |
| 4399 | **F** | 4396 | 4402 | AQSFLNR | 0.988 |
| 4400 | **L** | 4397 | 4403 | QSFLNRV | 0.982 |
| 4401 | **N** | 4398 | 4404 | SFLNRVC | 0.975 |
| 4402 | **R** | 4399 | 4405 | FLNRVCG | 0.964 |
| 4403 | **V** | 4400 | 4406 | LNRVCGV | 0.952 |
| 4404 | **C** | 4401 | 4407 | NRVCGVS | 0.938 |
| 4405 | **G** | 4402 | 4408 | RVCGVSA | 0.934 |
| 4406 | **V** | 4403 | 4409 | VCGVSAA | 0.937 |
| 4407 | **S** | 4404 | 4410 | CGVSAAR | 0.937 |
| 4408 | **A** | 4405 | 4411 | GVSAARL | 0.94 |
| 4409 | **A** | 4406 | 4412 | VSAARLT | 0.946 |
| 4410 | **R** | 4407 | 4413 | SAARLTP | 0.959 |
| 4411 | **L** | 4408 | 4414 | AARLTPC | 0.981 |
| 4412 | **T** | 4409 | 4415 | ARLTPCG | 1.004 |
| 4413 | **P** | 4410 | 4416 | RLTPCGT | 1.023 |
| 4414 | **C** | 4411 | 4417 | LTPCGTG | 1.036 |
| 4415 | **G** | 4412 | 4418 | TPCGTGT | 1.053 |
| 4416 | **T** | 4413 | 4419 | PCGTGTS | 1.073 |
| 4417 | **G** | 4414 | 4420 | CGTGTST | 1.091 |
| 4418 | **T** | 4415 | 4421 | GTGTSTD | 1.103 |
| 4419 | **S** | 4416 | 4422 | TGTSTDV | 1.098 |
| 4420 | **T** | 4417 | 4423 | GTSTDVV | 1.07 |
| 4421 | **D** | 4418 | 4424 | TSTDVVY | 1.033 |
| 4422 | **V** | 4419 | 4425 | STDVVYR | 0.987 |
| 4423 | **V** | 4420 | 4426 | TDVVYRA | 0.951 |
| 4424 | **Y** | 4421 | 4427 | DVVYRAF | 0.932 |
| 4425 | **R** | 4422 | 4428 | VVYRAFD | 0.921 |
| 4426 | **A** | 4423 | 4429 | VYRAFDI | 0.922 |
| 4427 | **F** | 4424 | 4430 | YRAFDIY | 0.92 |
| 4428 | **D** | 4425 | 4431 | RAFDIYN | 0.925 |
| 4429 | **I** | 4426 | 4432 | AFDIYND | 0.936 |
| 4430 | **Y** | 4427 | 4433 | FDIYNDK | 0.959 |
| 4431 | **N** | 4428 | 4434 | DIYNDKV | 0.983 |
| 4432 | **D** | 4429 | 4435 | IYNDKVA | 0.997 |
| 4433 | **K** | 4430 | 4436 | YNDKVAG | 0.998 |
| 4434 | **V** | 4431 | 4437 | NDKVAGF | 0.976 |
| 4435 | **A** | 4432 | 4438 | DKVAGFA | 0.956 |
| 4436 | **G** | 4433 | 4439 | KVAGFAK | 0.947 |
| 4437 | **F** | 4434 | 4440 | VAGFAKF | 0.944 |
| 4438 | **A** | 4435 | 4441 | AGFAKFL | 0.954 |
| 4439 | **K** | 4436 | 4442 | GFAKFLK | 0.972 |
| 4440 | **F** | 4437 | 4443 | FAKFLKT | 0.984 |
| 4441 | **L** | 4438 | 4444 | AKFLKTN | 1.002 |
| 4442 | **K** | 4439 | 4445 | KFLKTNC | 1.013 |
| 4443 | **T** | 4440 | 4446 | FLKTNCC | 1.002 |
| 4444 | **N** | 4441 | 4447 | LKTNCCR | 0.979 |
| 4445 | **C** | 4442 | 4448 | KTNCCRF | 0.945 |
| 4446 | **C** | 4443 | 4449 | TNCCRFQ | 0.926 |
| 4447 | **R** | 4444 | 4450 | NCCRFQE | 0.934 |
| 4448 | **F** | 4445 | 4451 | CCRFQEK | 0.964 |
| 4449 | **Q** | 4446 | 4452 | CRFQEKD | 1.005 |
| 4450 | **E** | 4447 | 4453 | RFQEKDE | 1.041 |
| 4451 | **K** | 4448 | 4454 | FQEKDED | 1.06 |
| 4452 | **D** | 4449 | 4455 | QEKDEDD | 1.063 |
| 4453 | **E** | 4450 | 4456 | EKDEDDN | 1.058 |
| 4454 | **D** | 4451 | 4457 | KDEDDNL | 1.04 |
| 4455 | **D** | 4452 | 4458 | DEDDNLI | 1.018 |
| 4456 | **N** | 4453 | 4459 | EDDNLID | 1.002 |
| 4457 | **L** | 4454 | 4460 | DDNLIDS | 0.991 |
| 4458 | **I** | 4455 | 4461 | DNLIDSY | 0.987 |
| 4459 | **D** | 4456 | 4462 | NLIDSYF | 0.992 |
| 4460 | **S** | 4457 | 4463 | LIDSYFV | 0.983 |
| 4461 | **Y** | 4458 | 4464 | IDSYFVV | 0.964 |
| 4462 | **F** | 4459 | 4465 | DSYFVVK | 0.957 |
| 4463 | **V** | 4460 | 4466 | SYFVVKR | 0.955 |
| 4464 | **V** | 4461 | 4467 | YFVVKRH | 0.968 |
| 4465 | **K** | 4462 | 4468 | FVVKRHT | 0.989 |
| 4466 | **R** | 4463 | 4469 | VVKRHTF | 0.992 |
| 4467 | **H** | 4464 | 4470 | VKRHTFS | 0.988 |
| 4468 | **T** | 4465 | 4471 | KRHTFSN | 0.982 |
| 4469 | **F** | 4466 | 4472 | RHTFSNY | 0.977 |
| 4470 | **S** | 4467 | 4473 | HTFSNYQ | 0.979 |
| 4471 | **N** | 4468 | 4474 | TFSNYQH | 0.975 |
| 4472 | **Y** | 4469 | 4475 | FSNYQHE | 0.971 |
| 4473 | **Q** | 4470 | 4476 | SNYQHEE | 0.975 |
| 4474 | **H** | 4471 | 4477 | NYQHEET | 0.991 |
| 4475 | **E** | 4472 | 4478 | YQHEETI | 1.006 |
| 4476 | **E** | 4473 | 4479 | QHEETIY | 1.013 |
| 4477 | **T** | 4474 | 4480 | HEETIYN | 1.0 |
| 4478 | **I** | 4475 | 4481 | EETIYNL | 0.972 |
| 4479 | **Y** | 4476 | 4482 | ETIYNLL | 0.954 |
| 4480 | **N** | 4477 | 4483 | TIYNLLK | 0.952 |
| 4481 | **L** | 4478 | 4484 | IYNLLKD | 0.97 |
| 4482 | **L** | 4479 | 4485 | YNLLKDC | 0.994 |
| 4483 | **K** | 4480 | 4486 | NLLKDCP | 1.011 |
| 4484 | **D** | 4481 | 4487 | LLKDCPA | 1.011 |
| 4485 | **C** | 4482 | 4488 | LKDCPAV | 0.99 |
| 4486 | **P** | 4483 | 4489 | KDCPAVA | 0.967 |
| 4487 | **A** | 4484 | 4490 | DCPAVAK | 0.955 |
| 4488 | **V** | 4485 | 4491 | CPAVAKH | 0.953 |
| 4489 | **A** | 4486 | 4492 | PAVAKHD | 0.963 |
| 4490 | **K** | 4487 | 4493 | AVAKHDF | 0.973 |
| 4491 | **H** | 4488 | 4494 | VAKHDFF | 0.965 |
| 4492 | **D** | 4489 | 4495 | AKHDFFK | 0.959 |
| 4493 | **F** | 4490 | 4496 | KHDFFKF | 0.953 |
| 4494 | **F** | 4491 | 4497 | HDFFKFR | 0.948 |
| 4495 | **K** | 4492 | 4498 | DFFKFRI | 0.957 |
| 4496 | **F** | 4493 | 4499 | FFKFRID | 0.964 |
| 4497 | **R** | 4494 | 4500 | FKFRIDG | 0.984 |
| 4498 | **I** | 4495 | 4501 | KFRIDGD | 1.017 |
| 4499 | **D** | 4496 | 4502 | FRIDGDM | 1.035 |
| 4500 | **G** | 4497 | 4503 | RIDGDMV | 1.041 |
| 4501 | **D** | 4498 | 4504 | IDGDMVP | 1.021 |
| 4502 | **M** | 4499 | 4505 | DGDMVPH | 0.98 |
| 4503 | **V** | 4500 | 4506 | GDMVPHI | 0.95 |
| 4504 | **P** | 4501 | 4507 | DMVPHIS | 0.938 |
| 4505 | **H** | 4502 | 4508 | MVPHISR | 0.946 |
| 4506 | **I** | 4503 | 4509 | VPHISRQ | 0.975 |
| 4507 | **S** | 4504 | 4510 | PHISRQR | 1.011 |
| 4508 | **R** | 4505 | 4511 | HISRQRL | 1.034 |
| 4509 | **Q** | 4506 | 4512 | ISRQRLT | 1.052 |
| 4510 | **R** | 4507 | 4513 | SRQRLTK | 1.051 |
| 4511 | **L** | 4508 | 4514 | RQRLTKY | 1.037 |
| 4512 | **T** | 4509 | 4515 | QRLTKYT | 1.027 |
| 4513 | **K** | 4510 | 4516 | RLTKYTM | 1.004 |
| 4514 | **Y** | 4511 | 4517 | LTKYTMA | 0.977 |
| 4515 | **T** | 4512 | 4518 | TKYTMAD | 0.953 |
| 4516 | **M** | 4513 | 4519 | KYTMADL | 0.932 |
| 4517 | **A** | 4514 | 4520 | YTMADLV | 0.927 |
| 4518 | **D** | 4515 | 4521 | TMADLVY | 0.923 |
| 4519 | **L** | 4516 | 4522 | MADLVYA | 0.919 |
| 4520 | **V** | 4517 | 4523 | ADLVYAL | 0.913 |
| 4521 | **Y** | 4518 | 4524 | DLVYALR | 0.902 |
| 4522 | **A** | 4519 | 4525 | LVYALRH | 0.906 |
| 4523 | **L** | 4520 | 4526 | VYALRHF | 0.914 |
| 4524 | **R** | 4521 | 4527 | YALRHFD | 0.93 |
| 4525 | **H** | 4522 | 4528 | ALRHFDE | 0.958 |
| 4526 | **F** | 4523 | 4529 | LRHFDEG | 0.991 |
| 4527 | **D** | 4524 | 4530 | RHFDEGN | 1.029 |
| 4528 | **E** | 4525 | 4531 | HFDEGNC | 1.051 |
| 4529 | **G** | 4526 | 4532 | FDEGNCD | 1.06 |
| 4530 | **N** | 4527 | 4533 | DEGNCDT | 1.052 |
| 4531 | **C** | 4528 | 4534 | EGNCDTL | 1.036 |
| 4532 | **D** | 4529 | 4535 | GNCDTLK | 1.035 |
| 4533 | **T** | 4530 | 4536 | NCDTLKE | 1.031 |
| 4534 | **L** | 4531 | 4537 | CDTLKEI | 1.023 |
| 4535 | **K** | 4532 | 4538 | DTLKEIL | 1.015 |
| 4536 | **E** | 4533 | 4539 | TLKEILV | 0.989 |
| 4537 | **I** | 4534 | 4540 | LKEILVT | 0.96 |
| 4538 | **L** | 4535 | 4541 | KEILVTY | 0.945 |
| 4539 | **V** | 4536 | 4542 | EILVTYN | 0.934 |
| 4540 | **T** | 4537 | 4543 | ILVTYNC | 0.929 |
| 4541 | **Y** | 4538 | 4544 | LVTYNCC | 0.928 |
| 4542 | **N** | 4539 | 4545 | VTYNCCD | 0.93 |
| 4543 | **C** | 4540 | 4546 | TYNCCDD | 0.938 |
| 4544 | **C** | 4541 | 4547 | YNCCDDD | 0.962 |
| 4545 | **D** | 4542 | 4548 | NCCDDDY | 0.993 |
| 4546 | **D** | 4543 | 4549 | CCDDDYF | 1.004 |
| 4547 | **D** | 4544 | 4550 | CDDDYFN | 1.008 |
| 4548 | **Y** | 4545 | 4551 | DDDYFNK | 0.999 |
| 4549 | **F** | 4546 | 4552 | DDYFNKK | 0.997 |
| 4550 | **N** | 4547 | 4553 | DYFNKKD | 1.016 |
| 4551 | **K** | 4548 | 4554 | YFNKKDW | 1.032 |
| 4552 | **K** | 4549 | 4555 | FNKKDWY | 1.038 |
| 4553 | **D** | 4550 | 4556 | NKKDWYD | 1.023 |
| 4554 | **W** | 4551 | 4557 | KKDWYDF | 0.986 |
| 4555 | **Y** | 4552 | 4558 | KDWYDFV | 0.956 |
| 4556 | **D** | 4553 | 4559 | DWYDFVE | 0.942 |
| 4557 | **F** | 4554 | 4560 | WYDFVEN | 0.949 |
| 4558 | **V** | 4555 | 4561 | YDFVENP | 0.977 |
| 4559 | **E** | 4556 | 4562 | DFVENPD | 1.014 |
| 4560 | **N** | 4557 | 4563 | FVENPDI | 1.036 |
| 4561 | **P** | 4558 | 4564 | VENPDIL | 1.036 |
| 4562 | **D** | 4559 | 4565 | ENPDILR | 1.018 |
| 4563 | **I** | 4560 | 4566 | NPDILRV | 0.98 |
| 4564 | **L** | 4561 | 4567 | PDILRVY | 0.947 |
| 4565 | **R** | 4562 | 4568 | DILRVYA | 0.923 |
| 4566 | **V** | 4563 | 4569 | ILRVYAN | 0.91 |
| 4567 | **Y** | 4564 | 4570 | LRVYANL | 0.91 |
| 4568 | **A** | 4565 | 4571 | RVYANLG | 0.926 |
| 4569 | **N** | 4566 | 4572 | VYANLGE | 0.952 |
| 4570 | **L** | 4567 | 4573 | YANLGER | 0.983 |
| 4571 | **G** | 4568 | 4574 | ANLGERV | 1.012 |
| 4572 | **E** | 4569 | 4575 | NLGERVR | 1.028 |
| 4573 | **R** | 4570 | 4576 | LGERVRQ | 1.03 |
| 4574 | **V** | 4571 | 4577 | GERVRQA | 1.021 |
| 4575 | **R** | 4572 | 4578 | ERVRQAL | 1.007 |
| 4576 | **Q** | 4573 | 4579 | RVRQALL | 0.989 |
| 4577 | **A** | 4574 | 4580 | VRQALLK | 0.98 |
| 4578 | **L** | 4575 | 4581 | RQALLKT | 0.982 |
| 4579 | **L** | 4576 | 4582 | QALLKTV | 0.991 |
| 4580 | **K** | 4577 | 4583 | ALLKTVQ | 1.0 |
| 4581 | **T** | 4578 | 4584 | LLKTVQF | 0.995 |
| 4582 | **V** | 4579 | 4585 | LKTVQFC | 0.973 |
| 4583 | **Q** | 4580 | 4586 | KTVQFCD | 0.943 |
| 4584 | **F** | 4581 | 4587 | TVQFCDA | 0.923 |
| 4585 | **C** | 4582 | 4588 | VQFCDAM | 0.909 |
| 4586 | **D** | 4583 | 4589 | QFCDAMR | 0.916 |
| 4587 | **A** | 4584 | 4590 | FCDAMRN | 0.931 |
| 4588 | **M** | 4585 | 4591 | CDAMRNA | 0.948 |
| 4589 | **R** | 4586 | 4592 | DAMRNAG | 0.972 |
| 4590 | **N** | 4587 | 4593 | AMRNAGI | 0.977 |
| 4591 | **A** | 4588 | 4594 | MRNAGIV | 0.974 |
| 4592 | **G** | 4589 | 4595 | RNAGIVG | 0.957 |
| 4593 | **I** | 4590 | 4596 | NAGIVGV | 0.936 |
| 4594 | **V** | 4591 | 4597 | AGIVGVL | 0.928 |
| 4595 | **G** | 4592 | 4598 | GIVGVLT | 0.926 |
| 4596 | **V** | 4593 | 4599 | IVGVLTL | 0.934 |
| 4597 | **L** | 4594 | 4600 | VGVLTLD | 0.951 |
| 4598 | **T** | 4595 | 4601 | GVLTLDN | 0.975 |
| 4599 | **L** | 4596 | 4602 | VLTLDNQ | 1.012 |
| 4600 | **D** | 4597 | 4603 | LTLDNQD | 1.054 |
| 4601 | **N** | 4598 | 4604 | TLDNQDL | 1.078 |
| 4602 | **Q** | 4599 | 4605 | LDNQDLN | 1.085 |
| 4603 | **D** | 4600 | 4606 | DNQDLNG | 1.077 |
| 4604 | **L** | 4601 | 4607 | NQDLNGN | 1.056 |
| 4605 | **N** | 4602 | 4608 | QDLNGNW | 1.039 |
| 4606 | **G** | 4603 | 4609 | DLNGNWY | 1.024 |
| 4607 | **N** | 4604 | 4610 | LNGNWYD | 0.998 |
| 4608 | **W** | 4605 | 4611 | NGNWYDF | 0.972 |
| 4609 | **Y** | 4606 | 4612 | GNWYDFG | 0.958 |
| 4610 | **D** | 4607 | 4613 | NWYDFGD | 0.958 |
| 4611 | **F** | 4608 | 4614 | WYDFGDF | 0.969 |
| 4612 | **G** | 4609 | 4615 | YDFGDFI | 0.983 |
| 4613 | **D** | 4610 | 4616 | DFGDFIQ | 0.989 |
| 4614 | **F** | 4611 | 4617 | FGDFIQT | 0.983 |
| 4615 | **I** | 4612 | 4618 | GDFIQTT | 0.989 |
| 4616 | **Q** | 4613 | 4619 | DFIQTTP | 1.008 |
| 4617 | **T** | 4614 | 4620 | FIQTTPG | 1.034 |
| 4618 | **T** | 4615 | 4621 | IQTTPGS | 1.067 |
| 4619 | **P** | 4616 | 4622 | QTTPGSG | 1.089 |
| 4620 | **G** | 4617 | 4623 | TTPGSGV | 1.095 |
| 4621 | **S** | 4618 | 4624 | TPGSGVP | 1.082 |
| 4622 | **G** | 4619 | 4625 | PGSGVPV | 1.047 |
| 4623 | **V** | 4620 | 4626 | GSGVPVV | 1.007 |
| 4624 | **P** | 4621 | 4627 | SGVPVVD | 0.978 |
| 4625 | **V** | 4622 | 4628 | GVPVVDS | 0.97 |
| 4626 | **V** | 4623 | 4629 | VPVVDSY | 0.977 |
| 4627 | **D** | 4624 | 4630 | PVVDSYY | 0.991 |
| 4628 | **S** | 4625 | 4631 | VVDSYYS | 0.988 |
| 4629 | **Y** | 4626 | 4632 | VDSYYSL | 0.973 |
| 4630 | **Y** | 4627 | 4633 | DSYYSLL | 0.957 |
| 4631 | **S** | 4628 | 4634 | SYYSLLM | 0.936 |
| 4632 | **L** | 4629 | 4635 | YYSLLMP | 0.926 |
| 4633 | **L** | 4630 | 4636 | YSLLMPI | 0.918 |
| 4634 | **M** | 4631 | 4637 | SLLMPIL | 0.912 |
| 4635 | **P** | 4632 | 4638 | LLMPILT | 0.916 |
| 4636 | **I** | 4633 | 4639 | LMPILTL | 0.92 |
| 4637 | **L** | 4634 | 4640 | MPILTLT | 0.94 |
| 4638 | **T** | 4635 | 4641 | PILTLTR | 0.96 |
| 4639 | **L** | 4636 | 4642 | ILTLTRA | 0.977 |
| 4640 | **T** | 4637 | 4643 | LTLTRAL | 0.992 |
| 4641 | **R** | 4638 | 4644 | TLTRALT | 0.989 |
| 4642 | **A** | 4639 | 4645 | LTRALTA | 0.983 |
| 4643 | **L** | 4640 | 4646 | TRALTAE | 0.982 |
| 4644 | **T** | 4641 | 4647 | RALTAES | 0.986 |
| 4645 | **A** | 4642 | 4648 | ALTAESH | 0.999 |
| 4646 | **E** | 4643 | 4649 | LTAESHV | 1.004 |
| 4647 | **S** | 4644 | 4650 | TAESHVD | 1.007 |
| 4648 | **H** | 4645 | 4651 | AESHVDT | 1.006 |
| 4649 | **V** | 4646 | 4652 | ESHVDTD | 1.012 |
| 4650 | **D** | 4647 | 4653 | SHVDTDL | 1.028 |
| 4651 | **T** | 4648 | 4654 | HVDTDLT | 1.039 |
| 4652 | **D** | 4649 | 4655 | VDTDLTK | 1.048 |
| 4653 | **L** | 4650 | 4656 | DTDLTKP | 1.05 |
| 4654 | **T** | 4651 | 4657 | TDLTKPY | 1.046 |
| 4655 | **K** | 4652 | 4658 | DLTKPYI | 1.035 |
| 4656 | **P** | 4653 | 4659 | LTKPYIK | 1.02 |
| 4657 | **Y** | 4654 | 4660 | TKPYIKW | 0.995 |
| 4658 | **I** | 4655 | 4661 | KPYIKWD | 0.973 |
| 4659 | **K** | 4656 | 4662 | PYIKWDL | 0.966 |
| 4660 | **W** | 4657 | 4663 | YIKWDLL | 0.954 |
| 4661 | **D** | 4658 | 4664 | IKWDLLK | 0.961 |
| 4662 | **L** | 4659 | 4665 | KWDLLKY | 0.969 |
| 4663 | **L** | 4660 | 4666 | WDLLKYD | 0.971 |
| 4664 | **K** | 4661 | 4667 | DLLKYDF | 0.978 |
| 4665 | **Y** | 4662 | 4668 | LLKYDFT | 0.975 |
| 4666 | **D** | 4663 | 4669 | LKYDFTE | 0.979 |
| 4667 | **F** | 4664 | 4670 | KYDFTEE | 0.996 |
| 4668 | **T** | 4665 | 4671 | YDFTEER | 1.02 |
| 4669 | **E** | 4666 | 4672 | DFTEERL | 1.039 |
| 4670 | **E** | 4667 | 4673 | FTEERLK | 1.048 |
| 4671 | **R** | 4668 | 4674 | TEERLKL | 1.038 |
| 4672 | **L** | 4669 | 4675 | EERLKLF | 1.015 |
| 4673 | **K** | 4670 | 4676 | ERLKLFD | 1.005 |
| 4674 | **L** | 4671 | 4677 | RLKLFDR | 0.995 |
| 4675 | **F** | 4672 | 4678 | LKLFDRY | 0.992 |
| 4676 | **D** | 4673 | 4679 | KLFDRYF | 0.995 |
| 4677 | **R** | 4674 | 4680 | LFDRYFK | 0.99 |
| 4678 | **Y** | 4675 | 4681 | FDRYFKY | 0.98 |
| 4679 | **F** | 4676 | 4682 | DRYFKYW | 0.971 |
| 4680 | **K** | 4677 | 4683 | RYFKYWD | 0.973 |
| 4681 | **Y** | 4678 | 4684 | YFKYWDQ | 0.984 |
| 4682 | **W** | 4679 | 4685 | FKYWDQT | 1.008 |
| 4683 | **D** | 4680 | 4686 | KYWDQTY | 1.034 |
| 4684 | **Q** | 4681 | 4687 | YWDQTYH | 1.041 |
| 4685 | **T** | 4682 | 4688 | WDQTYHP | 1.036 |
| 4686 | **Y** | 4683 | 4689 | DQTYHPN | 1.02 |
| 4687 | **H** | 4684 | 4690 | QTYHPNC | 1.001 |
| 4688 | **P** | 4685 | 4691 | TYHPNCV | 0.988 |
| 4689 | **N** | 4686 | 4692 | YHPNCVN | 0.971 |
| 4690 | **C** | 4687 | 4693 | HPNCVNC | 0.948 |
| 4691 | **V** | 4688 | 4694 | PNCVNCL | 0.934 |
| 4692 | **N** | 4689 | 4695 | NCVNCLD | 0.932 |
| 4693 | **C** | 4690 | 4696 | CVNCLDD | 0.946 |
| 4694 | **L** | 4691 | 4697 | VNCLDDR | 0.977 |
| 4695 | **D** | 4692 | 4698 | NCLDDRC | 0.997 |
| 4696 | **D** | 4693 | 4699 | CLDDRCI | 0.994 |
| 4697 | **R** | 4694 | 4700 | LDDRCIL | 0.978 |
| 4698 | **C** | 4695 | 4701 | DDRCILH | 0.944 |
| 4699 | **I** | 4696 | 4702 | DRCILHC | 0.918 |
| 4700 | **L** | 4697 | 4703 | RCILHCA | 0.91 |
| 4701 | **H** | 4698 | 4704 | CILHCAN | 0.91 |
| 4702 | **C** | 4699 | 4705 | ILHCANF | 0.92 |
| 4703 | **A** | 4700 | 4706 | LHCANFN | 0.928 |
| 4704 | **N** | 4701 | 4707 | HCANFNV | 0.93 |
| 4705 | **F** | 4702 | 4708 | CANFNVL | 0.931 |
| 4706 | **N** | 4703 | 4709 | ANFNVLF | 0.928 |
| 4707 | **V** | 4704 | 4710 | NFNVLFS | 0.933 |
| 4708 | **L** | 4705 | 4711 | FNVLFST | 0.946 |
| 4709 | **F** | 4706 | 4712 | NVLFSTV | 0.961 |
| 4710 | **S** | 4707 | 4713 | VLFSTVF | 0.976 |
| 4711 | **T** | 4708 | 4714 | LFSTVFP | 0.986 |
| 4712 | **V** | 4709 | 4715 | FSTVFPP | 0.989 |
| 4713 | **F** | 4710 | 4716 | STVFPPT | 1.001 |
| 4714 | **P** | 4711 | 4717 | TVFPPTS | 1.021 |
| 4715 | **P** | 4712 | 4718 | VFPPTSF | 1.029 |
| 4716 | **T** | 4713 | 4719 | FPPTSFG | 1.037 |
| 4717 | **S** | 4714 | 4720 | PPTSFGP | 1.035 |
| 4718 | **F** | 4715 | 4721 | PTSFGPL | 1.02 |
| 4719 | **G** | 4716 | 4722 | TSFGPLV | 1.014 |
| 4720 | **P** | 4717 | 4723 | SFGPLVR | 1.009 |
| 4721 | **L** | 4718 | 4724 | FGPLVRK | 1.002 |
| 4722 | **V** | 4719 | 4725 | GPLVRKI | 0.996 |
| 4723 | **R** | 4720 | 4726 | PLVRKIF | 0.99 |
| 4724 | **K** | 4721 | 4727 | LVRKIFV | 0.979 |
| 4725 | **I** | 4722 | 4728 | VRKIFVD | 0.968 |
| 4726 | **F** | 4723 | 4729 | RKIFVDG | 0.97 |
| 4727 | **V** | 4724 | 4730 | KIFVDGV | 0.978 |
| 4728 | **D** | 4725 | 4731 | IFVDGVP | 0.992 |
| 4729 | **G** | 4726 | 4732 | FVDGVPF | 0.995 |
| 4730 | **V** | 4727 | 4733 | VDGVPFV | 0.981 |
| 4731 | **P** | 4728 | 4734 | DGVPFVV | 0.959 |
| 4732 | **F** | 4729 | 4735 | GVPFVVS | 0.943 |
| 4733 | **V** | 4730 | 4736 | VPFVVST | 0.949 |
| 4734 | **V** | 4731 | 4737 | PFVVSTG | 0.971 |
| 4735 | **S** | 4732 | 4738 | FVVSTGY | 0.996 |
| 4736 | **T** | 4733 | 4739 | VVSTGYH | 1.005 |
| 4737 | **G** | 4734 | 4740 | VSTGYHF | 0.994 |
| 4738 | **Y** | 4735 | 4741 | STGYHFR | 0.973 |
| 4739 | **H** | 4736 | 4742 | TGYHFRE | 0.959 |
| 4740 | **F** | 4737 | 4743 | GYHFREL | 0.96 |
| 4741 | **R** | 4738 | 4744 | YHFRELG | 0.971 |
| 4742 | **E** | 4739 | 4745 | HFRELGV | 0.976 |
| 4743 | **L** | 4740 | 4746 | FRELGVV | 0.968 |
| 4744 | **G** | 4741 | 4747 | RELGVVH | 0.953 |
| 4745 | **V** | 4742 | 4748 | ELGVVHN | 0.944 |
| 4746 | **V** | 4743 | 4749 | LGVVHNQ | 0.955 |
| 4747 | **H** | 4744 | 4750 | GVVHNQD | 0.985 |
| 4748 | **N** | 4745 | 4751 | VVHNQDV | 1.018 |
| 4749 | **Q** | 4746 | 4752 | VHNQDVN | 1.041 |
| 4750 | **D** | 4747 | 4753 | HNQDVNL | 1.036 |
| 4751 | **V** | 4748 | 4754 | NQDVNLH | 1.012 |
| 4752 | **N** | 4749 | 4755 | QDVNLHS | 0.99 |
| 4753 | **L** | 4750 | 4756 | DVNLHSS | 0.988 |
| 4754 | **H** | 4751 | 4757 | VNLHSSR | 1.003 |
| 4755 | **S** | 4752 | 4758 | NLHSSRL | 1.027 |
| 4756 | **S** | 4753 | 4759 | LHSSRLS | 1.039 |
| 4757 | **R** | 4754 | 4760 | HSSRLSF | 1.022 |
| 4758 | **L** | 4755 | 4761 | SSRLSFK | 1.003 |
| 4759 | **S** | 4756 | 4762 | SRLSFKE | 0.988 |
| 4760 | **F** | 4757 | 4763 | RLSFKEL | 0.983 |
| 4761 | **K** | 4758 | 4764 | LSFKELL | 0.993 |
| 4762 | **E** | 4759 | 4765 | SFKELLV | 0.988 |
| 4763 | **L** | 4760 | 4766 | FKELLVY | 0.967 |
| 4764 | **L** | 4761 | 4767 | KELLVYA | 0.939 |
| 4765 | **V** | 4762 | 4768 | ELLVYAA | 0.913 |
| 4766 | **Y** | 4763 | 4769 | LLVYAAD | 0.909 |
| 4767 | **A** | 4764 | 4770 | LVYAADP | 0.933 |
| 4768 | **A** | 4765 | 4771 | VYAADPA | 0.964 |
| 4769 | **D** | 4766 | 4772 | YAADPAM | 0.985 |
| 4770 | **P** | 4767 | 4773 | AADPAMH | 0.983 |
| 4771 | **A** | 4768 | 4774 | ADPAMHA | 0.953 |
| 4772 | **M** | 4769 | 4775 | DPAMHAA | 0.92 |
| 4773 | **H** | 4770 | 4776 | PAMHAAS | 0.911 |
| 4774 | **A** | 4771 | 4777 | AMHAASG | 0.93 |
| 4775 | **A** | 4772 | 4778 | MHAASGN | 0.968 |
| 4776 | **S** | 4773 | 4779 | HAASGNL | 1.007 |
| 4777 | **G** | 4774 | 4780 | AASGNLL | 1.022 |
| 4778 | **N** | 4775 | 4781 | ASGNLLL | 1.011 |
| 4779 | **L** | 4776 | 4782 | SGNLLLD | 0.998 |
| 4780 | **L** | 4777 | 4783 | GNLLLDK | 0.992 |
| 4781 | **L** | 4778 | 4784 | NLLLDKR | 1.002 |
| 4782 | **D** | 4779 | 4785 | LLLDKRT | 1.029 |
| 4783 | **K** | 4780 | 4786 | LLDKRTT | 1.05 |
| 4784 | **R** | 4781 | 4787 | LDKRTTC | 1.048 |
| 4785 | **T** | 4782 | 4788 | DKRTTCF | 1.031 |
| 4786 | **T** | 4783 | 4789 | KRTTCFS | 0.998 |
| 4787 | **C** | 4784 | 4790 | RTTCFSV | 0.96 |
| 4788 | **F** | 4785 | 4791 | TTCFSVA | 0.936 |
| 4789 | **S** | 4786 | 4792 | TCFSVAA | 0.918 |
| 4790 | **V** | 4787 | 4793 | CFSVAAL | 0.913 |
| 4791 | **A** | 4788 | 4794 | FSVAALT | 0.922 |
| 4792 | **A** | 4789 | 4795 | SVAALTN | 0.945 |
| 4793 | **L** | 4790 | 4796 | VAALTNN | 0.977 |
| 4794 | **T** | 4791 | 4797 | AALTNNV | 1.003 |
| 4795 | **N** | 4792 | 4798 | ALTNNVA | 1.012 |
| 4796 | **N** | 4793 | 4799 | LTNNVAF | 0.995 |
| 4797 | **V** | 4794 | 4800 | TNNVAFQ | 0.971 |
| 4798 | **A** | 4795 | 4801 | NNVAFQT | 0.958 |
| 4799 | **F** | 4796 | 4802 | NVAFQTV | 0.96 |
| 4800 | **Q** | 4797 | 4803 | VAFQTVK | 0.985 |
| 4801 | **T** | 4798 | 4804 | AFQTVKP | 1.011 |
| 4802 | **V** | 4799 | 4805 | FQTVKPG | 1.034 |
| 4803 | **K** | 4800 | 4806 | QTVKPGN | 1.054 |
| 4804 | **P** | 4801 | 4807 | TVKPGNF | 1.053 |
| 4805 | **G** | 4802 | 4808 | VKPGNFN | 1.048 |
| 4806 | **N** | 4803 | 4809 | KPGNFNK | 1.034 |
| 4807 | **F** | 4804 | 4810 | PGNFNKD | 1.023 |
| 4808 | **N** | 4805 | 4811 | GNFNKDF | 1.021 |
| 4809 | **K** | 4806 | 4812 | NFNKDFY | 1.017 |
| 4810 | **D** | 4807 | 4813 | FNKDFYD | 1.007 |
| 4811 | **F** | 4808 | 4814 | NKDFYDF | 0.98 |
| 4812 | **Y** | 4809 | 4815 | KDFYDFA | 0.952 |
| 4813 | **D** | 4810 | 4816 | DFYDFAV | 0.93 |
| 4814 | **F** | 4811 | 4817 | FYDFAVS | 0.925 |
| 4815 | **A** | 4812 | 4818 | YDFAVSK | 0.942 |
| 4816 | **V** | 4813 | 4819 | DFAVSKG | 0.97 |
| 4817 | **S** | 4814 | 4820 | FAVSKGF | 0.997 |
| 4818 | **K** | 4815 | 4821 | AVSKGFF | 1.008 |
| 4819 | **G** | 4816 | 4822 | VSKGFFK | 1.007 |
| 4820 | **F** | 4817 | 4823 | SKGFFKE | 1.0 |
| 4821 | **F** | 4818 | 4824 | KGFFKEG | 1.009 |
| 4822 | **K** | 4819 | 4825 | GFFKEGS | 1.042 |
| 4823 | **E** | 4820 | 4826 | FFKEGSS | 1.073 |
| 4824 | **G** | 4821 | 4827 | FKEGSSV | 1.094 |
| 4825 | **S** | 4822 | 4828 | KEGSSVE | 1.088 |
| 4826 | **S** | 4823 | 4829 | EGSSVEL | 1.053 |
| 4827 | **V** | 4824 | 4830 | GSSVELK | 1.021 |
| 4828 | **E** | 4825 | 4831 | SSVELKH | 0.994 |
| 4829 | **L** | 4826 | 4832 | SVELKHF | 0.979 |
| 4830 | **K** | 4827 | 4833 | VELKHFF | 0.973 |
| 4831 | **H** | 4828 | 4834 | ELKHFFF | 0.958 |
| 4832 | **F** | 4829 | 4835 | LKHFFFA | 0.944 |
| 4833 | **F** | 4830 | 4836 | KHFFFAQ | 0.939 |
| 4834 | **F** | 4831 | 4837 | HFFFAQD | 0.944 |
| 4835 | **A** | 4832 | 4838 | FFFAQDG | 0.972 |
| 4836 | **Q** | 4833 | 4839 | FFAQDGN | 1.005 |
| 4837 | **D** | 4834 | 4840 | FAQDGNA | 1.025 |
| 4838 | **G** | 4835 | 4841 | AQDGNAA | 1.029 |
| 4839 | **N** | 4836 | 4842 | QDGNAAI | 1.004 |
| 4840 | **A** | 4837 | 4843 | DGNAAIS | 0.977 |
| 4841 | **A** | 4838 | 4844 | GNAAISD | 0.964 |
| 4842 | **I** | 4839 | 4845 | NAAISDY | 0.964 |
| 4843 | **S** | 4840 | 4846 | AAISDYD | 0.982 |
| 4844 | **D** | 4841 | 4847 | AISDYDY | 0.991 |
| 4845 | **Y** | 4842 | 4848 | ISDYDYY | 0.98 |
| 4846 | **D** | 4843 | 4849 | SDYDYYR | 0.962 |
| 4847 | **Y** | 4844 | 4850 | DYDYYRY | 0.943 |
| 4848 | **Y** | 4845 | 4851 | YDYYRYN | 0.931 |
| 4849 | **R** | 4846 | 4852 | DYYRYNL | 0.931 |
| 4850 | **Y** | 4847 | 4853 | YYRYNLP | 0.947 |
| 4851 | **N** | 4848 | 4854 | YRYNLPT | 0.966 |
| 4852 | **L** | 4849 | 4855 | RYNLPTM | 0.981 |
| 4853 | **P** | 4850 | 4856 | YNLPTMC | 0.989 |
| 4854 | **T** | 4851 | 4857 | NLPTMCD | 0.975 |
| 4855 | **M** | 4852 | 4858 | LPTMCDI | 0.952 |
| 4856 | **C** | 4853 | 4859 | PTMCDIR | 0.945 |
| 4857 | **D** | 4854 | 4860 | TMCDIRQ | 0.952 |
| 4858 | **I** | 4855 | 4861 | MCDIRQL | 0.97 |
| 4859 | **R** | 4856 | 4862 | CDIRQLL | 0.987 |
| 4860 | **Q** | 4857 | 4863 | DIRQLLF | 0.986 |
| 4861 | **L** | 4858 | 4864 | IRQLLFV | 0.968 |
| 4862 | **L** | 4859 | 4865 | RQLLFVV | 0.947 |
| 4863 | **F** | 4860 | 4866 | QLLFVVE | 0.931 |
| 4864 | **V** | 4861 | 4867 | LLFVVEV | 0.923 |
| 4865 | **V** | 4862 | 4868 | LFVVEVV | 0.923 |
| 4866 | **E** | 4863 | 4869 | FVVEVVD | 0.936 |
| 4867 | **V** | 4864 | 4870 | VVEVVDK | 0.957 |
| 4868 | **V** | 4865 | 4871 | VEVVDKY | 0.978 |
| 4869 | **D** | 4866 | 4872 | EVVDKYF | 0.996 |
| 4870 | **K** | 4867 | 4873 | VVDKYFD | 0.995 |
| 4871 | **Y** | 4868 | 4874 | VDKYFDC | 0.972 |
| 4872 | **F** | 4869 | 4875 | DKYFDCY | 0.948 |
| 4873 | **D** | 4870 | 4876 | KYFDCYD | 0.937 |
| 4874 | **C** | 4871 | 4877 | YFDCYDG | 0.948 |
| 4875 | **Y** | 4872 | 4878 | FDCYDGG | 0.983 |
| 4876 | **D** | 4873 | 4879 | DCYDGGC | 1.014 |
| 4877 | **G** | 4874 | 4880 | CYDGGCI | 1.022 |
| 4878 | **G** | 4875 | 4881 | YDGGCIN | 1.003 |
| 4879 | **C** | 4876 | 4882 | DGGCINA | 0.974 |
| 4880 | **I** | 4877 | 4883 | GGCINAN | 0.957 |
| 4881 | **N** | 4878 | 4884 | GCINANQ | 0.96 |
| 4882 | **A** | 4879 | 4885 | CINANQV | 0.976 |
| 4883 | **N** | 4880 | 4886 | INANQVI | 0.982 |
| 4884 | **Q** | 4881 | 4887 | NANQVIV | 0.975 |
| 4885 | **V** | 4882 | 4888 | ANQVIVN | 0.958 |
| 4886 | **I** | 4883 | 4889 | NQVIVNN | 0.946 |
| 4887 | **V** | 4884 | 4890 | QVIVNNL | 0.95 |
| 4888 | **N** | 4885 | 4891 | VIVNNLD | 0.97 |
| 4889 | **N** | 4886 | 4892 | IVNNLDK | 0.996 |
| 4890 | **L** | 4887 | 4893 | VNNLDKS | 1.02 |
| 4891 | **D** | 4888 | 4894 | NNLDKSA | 1.043 |
| 4892 | **K** | 4889 | 4895 | NLDKSAG | 1.046 |
| 4893 | **S** | 4890 | 4896 | LDKSAGF | 1.032 |
| 4894 | **A** | 4891 | 4897 | DKSAGFP | 1.009 |
| 4895 | **G** | 4892 | 4898 | KSAGFPF | 0.974 |
| 4896 | **F** | 4893 | 4899 | SAGFPFN | 0.955 |
| 4897 | **P** | 4894 | 4900 | AGFPFNK | 0.956 |
| 4898 | **F** | 4895 | 4901 | GFPFNKW | 0.963 |
| 4899 | **N** | 4896 | 4902 | FPFNKWG | 0.984 |
| 4900 | **K** | 4897 | 4903 | PFNKWGK | 1.005 |
| 4901 | **W** | 4898 | 4904 | FNKWGKA | 1.014 |
| 4902 | **G** | 4899 | 4905 | NKWGKAR | 1.021 |
| 4903 | **K** | 4900 | 4906 | KWGKARL | 1.017 |
| 4904 | **A** | 4901 | 4907 | WGKARLY | 0.993 |
| 4905 | **R** | 4902 | 4908 | GKARLYY | 0.964 |
| 4906 | **L** | 4903 | 4909 | KARLYYD | 0.948 |
| 4907 | **Y** | 4904 | 4910 | ARLYYDS | 0.943 |
| 4908 | **Y** | 4905 | 4911 | RLYYDSM | 0.96 |
| 4909 | **D** | 4906 | 4912 | LYYDSMS | 0.984 |
| 4910 | **S** | 4907 | 4913 | YYDSMSY | 0.988 |
| 4911 | **M** | 4908 | 4914 | YDSMSYE | 0.985 |
| 4912 | **S** | 4909 | 4915 | DSMSYED | 0.982 |
| 4913 | **Y** | 4910 | 4916 | SMSYEDQ | 0.993 |
| 4914 | **E** | 4911 | 4917 | MSYEDQD | 1.021 |
| 4915 | **D** | 4912 | 4918 | SYEDQDA | 1.044 |
| 4916 | **Q** | 4913 | 4919 | YEDQDAL | 1.054 |
| 4917 | **D** | 4914 | 4920 | EDQDALF | 1.034 |
| 4918 | **A** | 4915 | 4921 | DQDALFA | 0.994 |
| 4919 | **L** | 4916 | 4922 | QDALFAY | 0.958 |
| 4920 | **F** | 4917 | 4923 | DALFAYT | 0.937 |
| 4921 | **A** | 4918 | 4924 | ALFAYTK | 0.943 |
| 4922 | **Y** | 4919 | 4925 | LFAYTKR | 0.97 |
| 4923 | **T** | 4920 | 4926 | FAYTKRN | 1.003 |
| 4924 | **K** | 4921 | 4927 | AYTKRNV | 1.021 |
| 4925 | **R** | 4922 | 4928 | YTKRNVI | 1.014 |
| 4926 | **N** | 4923 | 4929 | TKRNVIP | 1.002 |
| 4927 | **V** | 4924 | 4930 | KRNVIPT | 0.987 |
| 4928 | **I** | 4925 | 4931 | RNVIPTI | 0.985 |
| 4929 | **P** | 4926 | 4932 | NVIPTIT | 1.005 |
| 4930 | **T** | 4927 | 4933 | VIPTITQ | 1.019 |
| 4931 | **I** | 4928 | 4934 | IPTITQM | 1.024 |
| 4932 | **T** | 4929 | 4935 | PTITQMN | 1.019 |
| 4933 | **Q** | 4930 | 4936 | TITQMNL | 0.999 |
| 4934 | **M** | 4931 | 4937 | ITQMNLK | 0.985 |
| 4935 | **N** | 4932 | 4938 | TQMNLKY | 0.976 |
| 4936 | **L** | 4933 | 4939 | QMNLKYA | 0.969 |
| 4937 | **K** | 4934 | 4940 | MNLKYAI | 0.963 |
| 4938 | **Y** | 4935 | 4941 | NLKYAIS | 0.946 |
| 4939 | **A** | 4936 | 4942 | LKYAISA | 0.938 |
| 4940 | **I** | 4937 | 4943 | KYAISAK | 0.943 |
| 4941 | **S** | 4938 | 4944 | YAISAKN | 0.968 |
| 4942 | **A** | 4939 | 4945 | AISAKNR | 1.007 |
| 4943 | **K** | 4940 | 4946 | ISAKNRA | 1.04 |
| 4944 | **N** | 4941 | 4947 | SAKNRAR | 1.057 |
| 4945 | **R** | 4942 | 4948 | AKNRART | 1.056 |
| 4946 | **A** | 4943 | 4949 | KNRARTV | 1.042 |
| 4947 | **R** | 4944 | 4950 | NRARTVA | 1.023 |
| 4948 | **T** | 4945 | 4951 | RARTVAG | 1.0 |
| 4949 | **V** | 4946 | 4952 | ARTVAGV | 0.976 |
| 4950 | **A** | 4947 | 4953 | RTVAGVS | 0.959 |
| 4951 | **G** | 4948 | 4954 | TVAGVSI | 0.945 |
| 4952 | **V** | 4949 | 4955 | VAGVSIC | 0.934 |
| 4953 | **S** | 4950 | 4956 | AGVSICS | 0.932 |
| 4954 | **I** | 4951 | 4957 | GVSICST | 0.938 |
| 4955 | **C** | 4952 | 4958 | VSICSTM | 0.951 |
| 4956 | **S** | 4953 | 4959 | SICSTMT | 0.977 |
| 4957 | **T** | 4954 | 4960 | ICSTMTN | 1.003 |
| 4958 | **M** | 4955 | 4961 | CSTMTNR | 1.021 |
| 4959 | **T** | 4956 | 4962 | STMTNRQ | 1.041 |
| 4960 | **N** | 4957 | 4963 | TMTNRQF | 1.04 |
| 4961 | **R** | 4958 | 4964 | MTNRQFH | 1.026 |
| 4962 | **Q** | 4959 | 4965 | TNRQFHQ | 1.011 |
| 4963 | **F** | 4960 | 4966 | NRQFHQK | 0.995 |
| 4964 | **H** | 4961 | 4967 | RQFHQKL | 0.99 |
| 4965 | **Q** | 4962 | 4968 | QFHQKLL | 0.997 |
| 4966 | **K** | 4963 | 4969 | FHQKLLK | 1.007 |
| 4967 | **L** | 4964 | 4970 | HQKLLKS | 1.012 |
| 4968 | **L** | 4965 | 4971 | QKLLKSI | 1.01 |
| 4969 | **K** | 4966 | 4972 | KLLKSIA | 1.002 |
| 4970 | **S** | 4967 | 4973 | LLKSIAA | 0.983 |
| 4971 | **I** | 4968 | 4974 | LKSIAAT | 0.966 |
| 4972 | **A** | 4969 | 4975 | KSIAATR | 0.963 |
| 4973 | **A** | 4970 | 4976 | SIAATRG | 0.973 |
| 4974 | **T** | 4971 | 4977 | IAATRGA | 0.997 |
| 4975 | **R** | 4972 | 4978 | AATRGAT | 1.014 |
| 4976 | **G** | 4973 | 4979 | ATRGATV | 1.016 |
| 4977 | **A** | 4974 | 4980 | TRGATVV | 0.999 |
| 4978 | **T** | 4975 | 4981 | RGATVVI | 0.968 |
| 4979 | **V** | 4976 | 4982 | GATVVIG | 0.95 |
| 4980 | **V** | 4977 | 4983 | ATVVIGT | 0.948 |
| 4981 | **I** | 4978 | 4984 | TVVIGTS | 0.971 |
| 4982 | **G** | 4979 | 4985 | VVIGTSK | 1.015 |
| 4983 | **T** | 4980 | 4986 | VIGTSKF | 1.044 |
| 4984 | **S** | 4981 | 4987 | IGTSKFY | 1.054 |
| 4985 | **K** | 4982 | 4988 | GTSKFYG | 1.041 |
| 4986 | **F** | 4983 | 4989 | TSKFYGG | 1.014 |
| 4987 | **Y** | 4984 | 4990 | SKFYGGW | 0.995 |
| 4988 | **G** | 4985 | 4991 | KFYGGWH | 0.986 |
| 4989 | **G** | 4986 | 4992 | FYGGWHN | 0.978 |
| 4990 | **W** | 4987 | 4993 | YGGWHNM | 0.962 |
| 4991 | **H** | 4988 | 4994 | GGWHNML | 0.948 |
| 4992 | **N** | 4989 | 4995 | GWHNMLK | 0.94 |
| 4993 | **M** | 4990 | 4996 | WHNMLKT | 0.947 |
| 4994 | **L** | 4991 | 4997 | HNMLKTV | 0.97 |
| 4995 | **K** | 4992 | 4998 | NMLKTVY | 0.988 |
| 4996 | **T** | 4993 | 4999 | MLKTVYS | 0.995 |
| 4997 | **V** | 4994 | 5000 | LKTVYSD | 0.998 |
| 4998 | **Y** | 4995 | 5001 | KTVYSDV | 0.999 |
| 4999 | **S** | 4996 | 5002 | TVYSDVE | 1.01 |
| 5000 | **D** | 4997 | 5003 | VYSDVEN | 1.026 |
| 5001 | **V** | 4998 | 5004 | YSDVENP | 1.04 |
| 5002 | **E** | 4999 | 5005 | SDVENPH | 1.049 |
| 5003 | **N** | 5000 | 5006 | DVENPHL | 1.044 |
| 5004 | **P** | 5001 | 5007 | VENPHLM | 1.019 |
| 5005 | **H** | 5002 | 5008 | ENPHLMG | 0.984 |
| 5006 | **L** | 5003 | 5009 | NPHLMGW | 0.949 |
| 5007 | **M** | 5004 | 5010 | PHLMGWD | 0.922 |
| 5008 | **G** | 5005 | 5011 | HLMGWDY | 0.917 |
| 5009 | **W** | 5006 | 5012 | LMGWDYP | 0.932 |
| 5010 | **D** | 5007 | 5013 | MGWDYPK | 0.959 |
| 5011 | **Y** | 5008 | 5014 | GWDYPKC | 0.987 |
| 5012 | **P** | 5009 | 5015 | WDYPKCD | 1.017 |
| 5013 | **K** | 5010 | 5016 | DYPKCDR | 1.033 |
| 5014 | **C** | 5011 | 5017 | YPKCDRA | 1.03 |
| 5015 | **D** | 5012 | 5018 | PKCDRAM | 1.02 |
| 5016 | **R** | 5013 | 5019 | KCDRAMP | 1.002 |
| 5017 | **A** | 5014 | 5020 | CDRAMPN | 0.986 |
| 5018 | **M** | 5015 | 5021 | DRAMPNM | 0.973 |
| 5019 | **P** | 5016 | 5022 | RAMPNML | 0.972 |
| 5020 | **N** | 5017 | 5023 | AMPNMLR | 0.96 |
| 5021 | **M** | 5018 | 5024 | MPNMLRI | 0.942 |
| 5022 | **L** | 5019 | 5025 | PNMLRIM | 0.926 |
| 5023 | **R** | 5020 | 5026 | NMLRIMA | 0.903 |
| 5024 | **I** | 5021 | 5027 | MLRIMAS | 0.893 |
| 5025 | **M** | 5022 | 5028 | LRIMASL | 0.894 |
| 5026 | **A** | 5023 | 5029 | RIMASLV | 0.905 |
| 5027 | **S** | 5024 | 5030 | IMASLVL | 0.916 |
| 5028 | **L** | 5025 | 5031 | MASLVLA | 0.927 |
| 5029 | **V** | 5026 | 5032 | ASLVLAR | 0.938 |
| 5030 | **L** | 5027 | 5033 | SLVLARK | 0.953 |
| 5031 | **A** | 5028 | 5034 | LVLARKH | 0.973 |
| 5032 | **R** | 5029 | 5035 | VLARKHT | 0.998 |
| 5033 | **K** | 5030 | 5036 | LARKHTT | 1.02 |
| 5034 | **H** | 5031 | 5037 | ARKHTTC | 1.019 |
| 5035 | **T** | 5032 | 5038 | RKHTTCC | 1.008 |
| 5036 | **T** | 5033 | 5039 | KHTTCCS | 0.982 |
| 5037 | **C** | 5034 | 5040 | HTTCCSL | 0.95 |
| 5038 | **C** | 5035 | 5041 | TTCCSLS | 0.933 |
| 5039 | **S** | 5036 | 5042 | TCCSLSH | 0.929 |
| 5040 | **L** | 5037 | 5043 | CCSLSHR | 0.932 |
| 5041 | **S** | 5038 | 5044 | CSLSHRF | 0.936 |
| 5042 | **H** | 5039 | 5045 | SLSHRFY | 0.938 |
| 5043 | **R** | 5040 | 5046 | LSHRFYR | 0.929 |
| 5044 | **F** | 5041 | 5047 | SHRFYRL | 0.924 |
| 5045 | **Y** | 5042 | 5048 | HRFYRLA | 0.926 |
| 5046 | **R** | 5043 | 5049 | RFYRLAN | 0.931 |
| 5047 | **L** | 5044 | 5050 | FYRLANE | 0.95 |
| 5048 | **A** | 5045 | 5051 | YRLANEC | 0.96 |
| 5049 | **N** | 5046 | 5052 | RLANECA | 0.968 |
| 5050 | **E** | 5047 | 5053 | LANECAQ | 0.964 |
| 5051 | **C** | 5048 | 5054 | ANECAQV | 0.945 |
| 5052 | **A** | 5049 | 5055 | NECAQVL | 0.935 |
| 5053 | **Q** | 5050 | 5056 | ECAQVLS | 0.933 |
| 5054 | **V** | 5051 | 5057 | CAQVLSE | 0.947 |
| 5055 | **L** | 5052 | 5058 | AQVLSEM | 0.964 |
| 5056 | **S** | 5053 | 5059 | QVLSEMV | 0.972 |
| 5057 | **E** | 5054 | 5060 | VLSEMVM | 0.96 |
| 5058 | **M** | 5055 | 5061 | LSEMVMC | 0.927 |
| 5059 | **V** | 5056 | 5062 | SEMVMCG | 0.91 |
| 5060 | **M** | 5057 | 5063 | EMVMCGG | 0.911 |
| 5061 | **C** | 5058 | 5064 | MVMCGGS | 0.942 |
| 5062 | **G** | 5059 | 5065 | VMCGGSL | 0.988 |
| 5063 | **G** | 5060 | 5066 | MCGGSLY | 1.01 |
| 5064 | **S** | 5061 | 5067 | CGGSLYV | 1.004 |
| 5065 | **L** | 5062 | 5068 | GGSLYVK | 0.985 |
| 5066 | **Y** | 5063 | 5069 | GSLYVKP | 0.967 |
| 5067 | **V** | 5064 | 5070 | SLYVKPG | 0.98 |
| 5068 | **K** | 5065 | 5071 | LYVKPGG | 1.021 |
| 5069 | **P** | 5066 | 5072 | YVKPGGT | 1.059 |
| 5070 | **G** | 5067 | 5073 | VKPGGTS | 1.098 |
| 5071 | **G** | 5068 | 5074 | KPGGTSS | 1.119 |
| 5072 | **T** | 5069 | 5075 | PGGTSSG | 1.128 |
| 5073 | **S** | 5070 | 5076 | GGTSSGD | 1.138 |
| 5074 | **S** | 5071 | 5077 | GTSSGDA | 1.132 |
| 5075 | **G** | 5072 | 5078 | TSSGDAT | 1.118 |
| 5076 | **D** | 5073 | 5079 | SSGDATT | 1.098 |
| 5077 | **A** | 5074 | 5080 | SGDATTA | 1.068 |
| 5078 | **T** | 5075 | 5081 | GDATTAY | 1.033 |
| 5079 | **T** | 5076 | 5082 | DATTAYA | 0.999 |
| 5080 | **A** | 5077 | 5083 | ATTAYAN | 0.968 |
| 5081 | **Y** | 5078 | 5084 | TTAYANS | 0.952 |
| 5082 | **A** | 5079 | 5085 | TAYANSV | 0.955 |
| 5083 | **N** | 5080 | 5086 | AYANSVF | 0.962 |
| 5084 | **S** | 5081 | 5087 | YANSVFN | 0.967 |
| 5085 | **V** | 5082 | 5088 | ANSVFNI | 0.956 |
| 5086 | **F** | 5083 | 5089 | NSVFNIC | 0.936 |
| 5087 | **N** | 5084 | 5090 | SVFNICQ | 0.917 |
| 5088 | **I** | 5085 | 5091 | VFNICQA | 0.904 |
| 5089 | **C** | 5086 | 5092 | FNICQAV | 0.902 |
| 5090 | **Q** | 5087 | 5093 | NICQAVT | 0.907 |
| 5091 | **A** | 5088 | 5094 | ICQAVTA | 0.924 |
| 5092 | **V** | 5089 | 5095 | CQAVTAN | 0.938 |
| 5093 | **T** | 5090 | 5096 | QAVTANV | 0.954 |
| 5094 | **A** | 5091 | 5097 | AVTANVN | 0.966 |
| 5095 | **N** | 5092 | 5098 | VTANVNA | 0.962 |
| 5096 | **V** | 5093 | 5099 | TANVNAL | 0.959 |
| 5097 | **N** | 5094 | 5100 | ANVNALL | 0.951 |
| 5098 | **A** | 5095 | 5101 | NVNALLS | 0.95 |
| 5099 | **L** | 5096 | 5102 | VNALLST | 0.964 |
| 5100 | **L** | 5097 | 5103 | NALLSTD | 0.985 |
| 5101 | **S** | 5098 | 5104 | ALLSTDG | 1.018 |
| 5102 | **T** | 5099 | 5105 | LLSTDGN | 1.049 |
| 5103 | **D** | 5100 | 5106 | LSTDGNK | 1.072 |
| 5104 | **G** | 5101 | 5107 | STDGNKI | 1.079 |
| 5105 | **N** | 5102 | 5108 | TDGNKIA | 1.063 |
| 5106 | **K** | 5103 | 5109 | DGNKIAD | 1.041 |
| 5107 | **I** | 5104 | 5110 | GNKIADK | 1.018 |
| 5108 | **A** | 5105 | 5111 | NKIADKY | 1.006 |
| 5109 | **D** | 5106 | 5112 | KIADKYV | 1.006 |
| 5110 | **K** | 5107 | 5113 | IADKYVR | 1.003 |
| 5111 | **Y** | 5108 | 5114 | ADKYVRN | 0.996 |
| 5112 | **V** | 5109 | 5115 | DKYVRNL | 0.988 |
| 5113 | **R** | 5110 | 5116 | KYVRNLQ | 0.98 |
| 5114 | **N** | 5111 | 5117 | YVRNLQH | 0.972 |
| 5115 | **L** | 5112 | 5118 | VRNLQHR | 0.962 |
| 5116 | **Q** | 5113 | 5119 | RNLQHRL | 0.949 |
| 5117 | **H** | 5114 | 5120 | NLQHRLY | 0.942 |
| 5118 | **R** | 5115 | 5121 | LQHRLYE | 0.935 |
| 5119 | **L** | 5116 | 5122 | QHRLYEC | 0.933 |
| 5120 | **Y** | 5117 | 5123 | HRLYECL | 0.929 |
| 5121 | **E** | 5118 | 5124 | RLYECLY | 0.922 |
| 5122 | **C** | 5119 | 5125 | LYECLYR | 0.924 |
| 5123 | **L** | 5120 | 5126 | YECLYRN | 0.942 |
| 5124 | **Y** | 5121 | 5127 | ECLYRNR | 0.971 |
| 5125 | **R** | 5122 | 5128 | CLYRNRD | 1.009 |
| 5126 | **N** | 5123 | 5129 | LYRNRDV | 1.038 |
| 5127 | **R** | 5124 | 5130 | YRNRDVD | 1.051 |
| 5128 | **D** | 5125 | 5131 | RNRDVDT | 1.058 |
| 5129 | **V** | 5126 | 5132 | NRDVDTD | 1.056 |
| 5130 | **D** | 5127 | 5133 | RDVDTDF | 1.052 |
| 5131 | **T** | 5128 | 5134 | DVDTDFV | 1.039 |
| 5132 | **D** | 5129 | 5135 | VDTDFVN | 1.021 |
| 5133 | **F** | 5130 | 5136 | DTDFVNE | 0.999 |
| 5134 | **V** | 5131 | 5137 | TDFVNEF | 0.981 |
| 5135 | **N** | 5132 | 5138 | DFVNEFY | 0.968 |
| 5136 | **E** | 5133 | 5139 | FVNEFYA | 0.952 |
| 5137 | **F** | 5134 | 5140 | VNEFYAY | 0.927 |
| 5138 | **Y** | 5135 | 5141 | NEFYAYL | 0.905 |
| 5139 | **A** | 5136 | 5142 | EFYAYLR | 0.9 |
| 5140 | **Y** | 5137 | 5143 | FYAYLRK | 0.914 |
| 5141 | **L** | 5138 | 5144 | YAYLRKH | 0.948 |
| 5142 | **R** | 5139 | 5145 | AYLRKHF | 0.976 |
| 5143 | **K** | 5140 | 5146 | YLRKHFS | 0.986 |
| 5144 | **H** | 5141 | 5147 | LRKHFSM | 0.97 |
| 5145 | **F** | 5142 | 5148 | RKHFSMM | 0.937 |
| 5146 | **S** | 5143 | 5149 | KHFSMMI | 0.905 |
| 5147 | **M** | 5144 | 5150 | HFSMMIL | 0.882 |
| 5148 | **M** | 5145 | 5151 | FSMMILS | 0.884 |
| 5149 | **I** | 5146 | 5152 | SMMILSD | 0.91 |
| 5150 | **L** | 5147 | 5153 | MMILSDD | 0.952 |
| 5151 | **S** | 5148 | 5154 | MILSDDA | 0.99 |
| 5152 | **D** | 5149 | 5155 | ILSDDAV | 1.009 |
| 5153 | **D** | 5150 | 5156 | LSDDAVV | 1.006 |
| 5154 | **A** | 5151 | 5157 | SDDAVVC | 0.978 |
| 5155 | **V** | 5152 | 5158 | DDAVVCF | 0.95 |
| 5156 | **V** | 5153 | 5159 | DAVVCFN | 0.936 |
| 5157 | **C** | 5154 | 5160 | AVVCFNS | 0.946 |
| 5158 | **F** | 5155 | 5161 | VVCFNST | 0.973 |
| 5159 | **N** | 5156 | 5162 | VCFNSTY | 1.004 |
| 5160 | **S** | 5157 | 5163 | CFNSTYA | 1.025 |
| 5161 | **T** | 5158 | 5164 | FNSTYAS | 1.023 |
| 5162 | **Y** | 5159 | 5165 | NSTYASQ | 1.02 |
| 5163 | **A** | 5160 | 5166 | STYASQG | 1.023 |
| 5164 | **S** | 5161 | 5167 | TYASQGL | 1.03 |
| 5165 | **Q** | 5162 | 5168 | YASQGLV | 1.037 |
| 5166 | **G** | 5163 | 5169 | ASQGLVA | 1.023 |
| 5167 | **L** | 5164 | 5170 | SQGLVAS | 0.994 |
| 5168 | **V** | 5165 | 5171 | QGLVASI | 0.967 |
| 5169 | **A** | 5166 | 5172 | GLVASIK | 0.959 |
| 5170 | **S** | 5167 | 5173 | LVASIKN | 0.968 |
| 5171 | **I** | 5168 | 5174 | VASIKNF | 0.986 |
| 5172 | **K** | 5169 | 5175 | ASIKNFK | 1.005 |
| 5173 | **N** | 5170 | 5176 | SIKNFKS | 1.012 |
| 5174 | **F** | 5171 | 5177 | IKNFKSV | 1.011 |
| 5175 | **K** | 5172 | 5178 | KNFKSVL | 1.008 |
| 5176 | **S** | 5173 | 5179 | NFKSVLY | 0.985 |
| 5177 | **V** | 5174 | 5180 | FKSVLYY | 0.957 |
| 5178 | **L** | 5175 | 5181 | KSVLYYQ | 0.94 |
| 5179 | **Y** | 5176 | 5182 | SVLYYQN | 0.936 |
| 5180 | **Y** | 5177 | 5183 | VLYYQNN | 0.958 |
| 5181 | **Q** | 5178 | 5184 | LYYQNNV | 0.987 |
| 5182 | **N** | 5179 | 5185 | YYQNNVF | 1.002 |
| 5183 | **N** | 5180 | 5186 | YQNNVFM | 0.99 |
| 5184 | **V** | 5181 | 5187 | QNNVFMS | 0.969 |
| 5185 | **F** | 5182 | 5188 | NNVFMSE | 0.955 |
| 5186 | **M** | 5183 | 5189 | NVFMSEA | 0.957 |
| 5187 | **S** | 5184 | 5190 | VFMSEAK | 0.986 |
| 5188 | **E** | 5185 | 5191 | FMSEAKC | 1.003 |
| 5189 | **A** | 5186 | 5192 | MSEAKCW | 1.005 |
| 5190 | **K** | 5187 | 5193 | SEAKCWT | 0.999 |
| 5191 | **C** | 5188 | 5194 | EAKCWTE | 0.984 |
| 5192 | **W** | 5189 | 5195 | AKCWTET | 0.992 |
| 5193 | **T** | 5190 | 5196 | KCWTETD | 1.018 |
| 5194 | **E** | 5191 | 5197 | CWTETDL | 1.038 |
| 5195 | **T** | 5192 | 5198 | WTETDLT | 1.053 |
| 5196 | **D** | 5193 | 5199 | TETDLTK | 1.057 |
| 5197 | **L** | 5194 | 5200 | ETDLTKG | 1.053 |
| 5198 | **T** | 5195 | 5201 | TDLTKGP | 1.063 |
| 5199 | **K** | 5196 | 5202 | DLTKGPH | 1.07 |
| 5200 | **G** | 5197 | 5203 | LTKGPHE | 1.067 |
| 5201 | **P** | 5198 | 5204 | TKGPHEF | 1.045 |
| 5202 | **H** | 5199 | 5205 | KGPHEFC | 1.004 |
| 5203 | **E** | 5200 | 5206 | GPHEFCS | 0.971 |
| 5204 | **F** | 5201 | 5207 | PHEFCSQ | 0.953 |
| 5205 | **C** | 5202 | 5208 | HEFCSQH | 0.955 |
| 5206 | **S** | 5203 | 5209 | EFCSQHT | 0.973 |
| 5207 | **Q** | 5204 | 5210 | FCSQHTM | 0.975 |
| 5208 | **H** | 5205 | 5211 | CSQHTML | 0.965 |
| 5209 | **T** | 5206 | 5212 | SQHTMLV | 0.946 |
| 5210 | **M** | 5207 | 5213 | QHTMLVK | 0.934 |
| 5211 | **L** | 5208 | 5214 | HTMLVKQ | 0.952 |
| 5212 | **V** | 5209 | 5215 | TMLVKQG | 0.99 |
| 5213 | **K** | 5210 | 5216 | MLVKQGD | 1.039 |
| 5214 | **Q** | 5211 | 5217 | LVKQGDD | 1.079 |
| 5215 | **G** | 5212 | 5218 | VKQGDDY | 1.085 |
| 5216 | **D** | 5213 | 5219 | KQGDDYV | 1.063 |
| 5217 | **D** | 5214 | 5220 | QGDDYVY | 1.022 |
| 5218 | **Y** | 5215 | 5221 | GDDYVYL | 0.973 |
| 5219 | **V** | 5216 | 5222 | DDYVYLP | 0.939 |
| 5220 | **Y** | 5217 | 5223 | DYVYLPY | 0.922 |
| 5221 | **L** | 5218 | 5224 | YVYLPYP | 0.932 |
| 5222 | **P** | 5219 | 5225 | VYLPYPD | 0.955 |
| 5223 | **Y** | 5220 | 5226 | YLPYPDP | 0.986 |
| 5224 | **P** | 5221 | 5227 | LPYPDPS | 1.027 |
| 5225 | **D** | 5222 | 5228 | PYPDPSR | 1.048 |
| 5226 | **P** | 5223 | 5229 | YPDPSRI | 1.057 |
| 5227 | **S** | 5224 | 5230 | PDPSRIL | 1.051 |
| 5228 | **R** | 5225 | 5231 | DPSRILG | 1.018 |
| 5229 | **I** | 5226 | 5232 | PSRILGA | 0.988 |
| 5230 | **L** | 5227 | 5233 | SRILGAG | 0.97 |
| 5231 | **G** | 5228 | 5234 | RILGAGC | 0.952 |
| 5232 | **A** | 5229 | 5235 | ILGAGCF | 0.949 |
| 5233 | **G** | 5230 | 5236 | LGAGCFV | 0.938 |
| 5234 | **C** | 5231 | 5237 | GAGCFVD | 0.936 |
| 5235 | **F** | 5232 | 5238 | AGCFVDD | 0.952 |
| 5236 | **V** | 5233 | 5239 | GCFVDDI | 0.967 |
| 5237 | **D** | 5234 | 5240 | CFVDDIV | 0.989 |
| 5238 | **D** | 5235 | 5241 | FVDDIVK | 1.0 |
| 5239 | **I** | 5236 | 5242 | VDDIVKT | 0.997 |
| 5240 | **V** | 5237 | 5243 | DDIVKTD | 1.005 |
| 5241 | **K** | 5238 | 5244 | DIVKTDG | 1.026 |
| 5242 | **T** | 5239 | 5245 | IVKTDGT | 1.045 |
| 5243 | **D** | 5240 | 5246 | VKTDGTL | 1.058 |
| 5244 | **G** | 5241 | 5247 | KTDGTLM | 1.048 |
| 5245 | **T** | 5242 | 5248 | TDGTLMI | 1.009 |
| 5246 | **L** | 5243 | 5249 | DGTLMIE | 0.972 |
| 5247 | **M** | 5244 | 5250 | GTLMIER | 0.945 |
| 5248 | **I** | 5245 | 5251 | TLMIERF | 0.939 |
| 5249 | **E** | 5246 | 5252 | LMIERFV | 0.951 |
| 5250 | **R** | 5247 | 5253 | MIERFVS | 0.958 |
| 5251 | **F** | 5248 | 5254 | IERFVSL | 0.955 |
| 5252 | **V** | 5249 | 5255 | ERFVSLA | 0.945 |
| 5253 | **S** | 5250 | 5256 | RFVSLAI | 0.93 |
| 5254 | **L** | 5251 | 5257 | FVSLAID | 0.923 |
| 5255 | **A** | 5252 | 5258 | VSLAIDA | 0.919 |
| 5256 | **I** | 5253 | 5259 | SLAIDAY | 0.919 |
| 5257 | **D** | 5254 | 5260 | LAIDAYP | 0.924 |
| 5258 | **A** | 5255 | 5261 | AIDAYPL | 0.93 |
| 5259 | **Y** | 5256 | 5262 | IDAYPLT | 0.943 |
| 5260 | **P** | 5257 | 5263 | DAYPLTK | 0.964 |
| 5261 | **L** | 5258 | 5264 | AYPLTKH | 0.986 |
| 5262 | **T** | 5259 | 5265 | YPLTKHP | 1.012 |
| 5263 | **K** | 5260 | 5266 | PLTKHPN | 1.036 |
| 5264 | **H** | 5261 | 5267 | LTKHPNQ | 1.056 |
| 5265 | **P** | 5262 | 5268 | TKHPNQE | 1.076 |
| 5266 | **N** | 5263 | 5269 | KHPNQEY | 1.079 |
| 5267 | **Q** | 5264 | 5270 | HPNQEYA | 1.067 |
| 5268 | **E** | 5265 | 5271 | PNQEYAD | 1.036 |
| 5269 | **Y** | 5266 | 5272 | NQEYADV | 0.994 |
| 5270 | **A** | 5267 | 5273 | QEYADVF | 0.961 |
| 5271 | **D** | 5268 | 5274 | EYADVFH | 0.935 |
| 5272 | **V** | 5269 | 5275 | YADVFHL | 0.924 |
| 5273 | **F** | 5270 | 5276 | ADVFHLY | 0.913 |
| 5274 | **H** | 5271 | 5277 | DVFHLYL | 0.906 |
| 5275 | **L** | 5272 | 5278 | VFHLYLQ | 0.902 |
| 5276 | **Y** | 5273 | 5279 | FHLYLQY | 0.9 |
| 5277 | **L** | 5274 | 5280 | HLYLQYI | 0.906 |
| 5278 | **Q** | 5275 | 5281 | LYLQYIR | 0.914 |
| 5279 | **Y** | 5276 | 5282 | YLQYIRK | 0.934 |
| 5280 | **I** | 5277 | 5283 | LQYIRKL | 0.956 |
| 5281 | **R** | 5278 | 5284 | QYIRKLH | 0.978 |
| 5282 | **K** | 5279 | 5285 | YIRKLHD | 1.0 |
| 5283 | **L** | 5280 | 5286 | IRKLHDE | 1.007 |
| 5284 | **H** | 5281 | 5287 | RKLHDEL | 1.012 |
| 5285 | **D** | 5282 | 5288 | KLHDELT | 1.019 |
| 5286 | **E** | 5283 | 5289 | LHDELTG | 1.02 |
| 5287 | **L** | 5284 | 5290 | HDELTGH | 1.018 |
| 5288 | **T** | 5285 | 5291 | DELTGHM | 1.006 |
| 5289 | **G** | 5286 | 5292 | ELTGHML | 0.982 |
| 5290 | **H** | 5287 | 5293 | LTGHMLD | 0.954 |
| 5291 | **M** | 5288 | 5294 | TGHMLDM | 0.926 |
| 5292 | **L** | 5289 | 5295 | GHMLDMY | 0.916 |
| 5293 | **D** | 5290 | 5296 | HMLDMYS | 0.909 |
| 5294 | **M** | 5291 | 5297 | MLDMYSV | 0.908 |
| 5295 | **Y** | 5292 | 5298 | LDMYSVM | 0.91 |
| 5296 | **S** | 5293 | 5299 | DMYSVML | 0.906 |
| 5297 | **V** | 5294 | 5300 | MYSVMLT | 0.912 |
| 5298 | **M** | 5295 | 5301 | YSVMLTN | 0.93 |
| 5299 | **L** | 5296 | 5302 | SVMLTND | 0.966 |
| 5300 | **T** | 5297 | 5303 | VMLTNDN | 1.01 |
| 5301 | **N** | 5298 | 5304 | MLTNDNT | 1.047 |
| 5302 | **D** | 5299 | 5305 | LTNDNTS | 1.076 |
| 5303 | **N** | 5300 | 5306 | TNDNTSR | 1.09 |
| 5304 | **T** | 5301 | 5307 | NDNTSRY | 1.082 |
| 5305 | **S** | 5302 | 5308 | DNTSRYW | 1.064 |
| 5306 | **R** | 5303 | 5309 | NTSRYWE | 1.034 |
| 5307 | **Y** | 5304 | 5310 | TSRYWEP | 1.006 |
| 5308 | **W** | 5305 | 5311 | SRYWEPE | 0.996 |
| 5309 | **E** | 5306 | 5312 | RYWEPEF | 0.996 |
| 5310 | **P** | 5307 | 5313 | YWEPEFY | 0.997 |
| 5311 | **E** | 5308 | 5314 | WEPEFYE | 0.989 |
| 5312 | **F** | 5309 | 5315 | EPEFYEA | 0.969 |
| 5313 | **Y** | 5310 | 5316 | PEFYEAM | 0.942 |
| 5314 | **E** | 5311 | 5317 | EFYEAMY | 0.922 |
| 5315 | **A** | 5312 | 5318 | FYEAMYT | 0.917 |
| 5316 | **M** | 5313 | 5319 | YEAMYTP | 0.927 |
| 5317 | **Y** | 5314 | 5320 | EAMYTPH | 0.954 |
| 5318 | **T** | 5315 | 5321 | AMYTPHT | 0.981 |
| 5319 | **P** | 5316 | 5322 | MYTPHTV | 0.994 |
| 5320 | **H** | 5317 | 5323 | YTPHTVL | 0.989 |
| 5321 | **T** | 5318 | 5324 | TPHTVLQ | 0.968 |
| 5322 | **V** | 5319 | 5325 | PHTVLQA | 0.947 |
| 5323 | **L** | 5320 | 5326 | HTVLQAV | 0.934 |
| 5324 | **Q** | 5321 | 5327 | TVLQAVG | 0.927 |
| 5325 | **A** | 5322 | 5328 | VLQAVGA | 0.929 |
| 5326 | **V** | 5323 | 5329 | LQAVGAC | 0.929 |
| 5327 | **G** | 5324 | 5330 | QAVGACV | 0.928 |
| 5328 | **A** | 5325 | 5331 | AVGACVL | 0.93 |
| 5329 | **C** | 5326 | 5332 | VGACVLC | 0.923 |
| 5330 | **V** | 5327 | 5333 | GACVLCN | 0.923 |
| 5331 | **L** | 5328 | 5334 | ACVLCNS | 0.94 |
| 5332 | **C** | 5329 | 5335 | CVLCNSQ | 0.972 |
| 5333 | **N** | 5330 | 5336 | VLCNSQT | 1.02 |
| 5334 | **S** | 5331 | 5337 | LCNSQTS | 1.066 |
| 5335 | **Q** | 5332 | 5338 | CNSQTSL | 1.084 |
| 5336 | **T** | 5333 | 5339 | NSQTSLR | 1.069 |
| 5337 | **S** | 5334 | 5340 | SQTSLRC | 1.036 |
| 5338 | **L** | 5335 | 5341 | QTSLRCG | 0.992 |
| 5339 | **R** | 5336 | 5342 | TSLRCGA | 0.959 |
| 5340 | **C** | 5337 | 5343 | SLRCGAC | 0.944 |
| 5341 | **G** | 5338 | 5344 | LRCGACI | 0.933 |
| 5342 | **A** | 5339 | 5345 | RCGACIR | 0.935 |
| 5343 | **C** | 5340 | 5346 | CGACIRR | 0.945 |
| 5344 | **I** | 5341 | 5347 | GACIRRP | 0.963 |
| 5345 | **R** | 5342 | 5348 | ACIRRPF | 0.986 |
| 5346 | **R** | 5343 | 5349 | CIRRPFL | 0.997 |
| 5347 | **P** | 5344 | 5350 | IRRPFLC | 0.994 |
| 5348 | **F** | 5345 | 5351 | RRPFLCC | 0.969 |
| 5349 | **L** | 5346 | 5352 | RPFLCCK | 0.951 |
| 5350 | **C** | 5347 | 5353 | PFLCCKC | 0.937 |
| 5351 | **C** | 5348 | 5354 | FLCCKCC | 0.931 |
| 5352 | **K** | 5349 | 5355 | LCCKCCY | 0.94 |
| 5353 | **C** | 5350 | 5356 | CCKCCYD | 0.933 |
| 5354 | **C** | 5351 | 5357 | CKCCYDH | 0.933 |
| 5355 | **Y** | 5352 | 5358 | KCCYDHV | 0.933 |
| 5356 | **D** | 5353 | 5359 | CCYDHVI | 0.926 |
| 5357 | **H** | 5354 | 5360 | CYDHVIS | 0.935 |
| 5358 | **V** | 5355 | 5361 | YDHVIST | 0.947 |
| 5359 | **I** | 5356 | 5362 | DHVISTS | 0.968 |
| 5360 | **S** | 5357 | 5363 | HVISTSH | 0.996 |
| 5361 | **T** | 5358 | 5364 | VISTSHK | 1.019 |
| 5362 | **S** | 5359 | 5365 | ISTSHKL | 1.026 |
| 5363 | **H** | 5360 | 5366 | STSHKLV | 1.017 |
| 5364 | **K** | 5361 | 5367 | TSHKLVL | 1.001 |
| 5365 | **L** | 5362 | 5368 | SHKLVLS | 0.976 |
| 5366 | **V** | 5363 | 5369 | HKLVLSV | 0.959 |
| 5367 | **L** | 5364 | 5370 | KLVLSVN | 0.956 |
| 5368 | **S** | 5365 | 5371 | LVLSVNP | 0.963 |
| 5369 | **V** | 5366 | 5372 | VLSVNPY | 0.978 |
| 5370 | **N** | 5367 | 5373 | LSVNPYV | 0.988 |
| 5371 | **P** | 5368 | 5374 | SVNPYVC | 0.984 |
| 5372 | **Y** | 5369 | 5375 | VNPYVCN | 0.962 |
| 5373 | **V** | 5370 | 5376 | NPYVCNA | 0.947 |
| 5374 | **C** | 5371 | 5377 | PYVCNAP | 0.947 |
| 5375 | **N** | 5372 | 5378 | YVCNAPG | 0.965 |
| 5376 | **A** | 5373 | 5379 | VCNAPGC | 0.995 |
| 5377 | **P** | 5374 | 5380 | CNAPGCD | 1.011 |
| 5378 | **G** | 5375 | 5381 | NAPGCDV | 1.01 |
| 5379 | **C** | 5376 | 5382 | APGCDVT | 0.999 |
| 5380 | **D** | 5377 | 5383 | PGCDVTD | 0.995 |
| 5381 | **V** | 5378 | 5384 | GCDVTDV | 1.0 |
| 5382 | **T** | 5379 | 5385 | CDVTDVT | 1.016 |
| 5383 | **D** | 5380 | 5386 | DVTDVTQ | 1.03 |
| 5384 | **V** | 5381 | 5387 | VTDVTQL | 1.028 |
| 5385 | **T** | 5382 | 5388 | TDVTQLY | 1.014 |
| 5386 | **Q** | 5383 | 5389 | DVTQLYL | 0.99 |
| 5387 | **L** | 5384 | 5390 | VTQLYLG | 0.968 |
| 5388 | **Y** | 5385 | 5391 | TQLYLGG | 0.959 |
| 5389 | **L** | 5386 | 5392 | QLYLGGM | 0.966 |
| 5390 | **G** | 5387 | 5393 | LYLGGMS | 0.977 |
| 5391 | **G** | 5388 | 5394 | YLGGMSY | 0.979 |
| 5392 | **M** | 5389 | 5395 | LGGMSYY | 0.964 |
| 5393 | **S** | 5390 | 5396 | GGMSYYC | 0.938 |
| 5394 | **Y** | 5391 | 5397 | GMSYYCK | 0.923 |
| 5395 | **Y** | 5392 | 5398 | MSYYCKS | 0.924 |
| 5396 | **C** | 5393 | 5399 | SYYCKSH | 0.946 |
| 5397 | **K** | 5394 | 5400 | YYCKSHK | 0.985 |
| 5398 | **S** | 5395 | 5401 | YCKSHKP | 1.012 |
| 5399 | **H** | 5396 | 5402 | CKSHKPP | 1.035 |
| 5400 | **K** | 5397 | 5403 | KSHKPPI | 1.049 |
| 5401 | **P** | 5398 | 5404 | SHKPPIS | 1.041 |
| 5402 | **P** | 5399 | 5405 | HKPPISF | 1.027 |
| 5403 | **I** | 5400 | 5406 | KPPISFP | 1.001 |
| 5404 | **S** | 5401 | 5407 | PPISFPL | 0.971 |
| 5405 | **F** | 5402 | 5408 | PISFPLC | 0.951 |
| 5406 | **P** | 5403 | 5409 | ISFPLCA | 0.94 |
| 5407 | **L** | 5404 | 5410 | SFPLCAN | 0.944 |
| 5408 | **C** | 5405 | 5411 | FPLCANG | 0.961 |
| 5409 | **A** | 5406 | 5412 | PLCANGQ | 0.984 |
| 5410 | **N** | 5407 | 5413 | LCANGQV | 1.005 |
| 5411 | **G** | 5408 | 5414 | CANGQVF | 1.016 |
| 5412 | **Q** | 5409 | 5415 | ANGQVFG | 1.002 |
| 5413 | **V** | 5410 | 5416 | NGQVFGL | 0.977 |
| 5414 | **F** | 5411 | 5417 | GQVFGLY | 0.953 |
| 5415 | **G** | 5412 | 5418 | QVFGLYK | 0.946 |
| 5416 | **L** | 5413 | 5419 | VFGLYKN | 0.965 |
| 5417 | **Y** | 5414 | 5420 | FGLYKNT | 0.993 |
| 5418 | **K** | 5415 | 5421 | GLYKNTC | 1.018 |
| 5419 | **N** | 5416 | 5422 | LYKNTCV | 1.023 |
| 5420 | **T** | 5417 | 5423 | YKNTCVG | 1.011 |
| 5421 | **C** | 5418 | 5424 | KNTCVGS | 1.001 |
| 5422 | **V** | 5419 | 5425 | NTCVGSD | 1.004 |
| 5423 | **G** | 5420 | 5426 | TCVGSDN | 1.021 |
| 5424 | **S** | 5421 | 5427 | CVGSDNV | 1.039 |
| 5425 | **D** | 5422 | 5428 | VGSDNVT | 1.043 |
| 5426 | **N** | 5423 | 5429 | GSDNVTD | 1.04 |
| 5427 | **V** | 5424 | 5430 | SDNVTDF | 1.028 |
| 5428 | **T** | 5425 | 5431 | DNVTDFN | 1.016 |
| 5429 | **D** | 5426 | 5432 | NVTDFNA | 1.005 |
| 5430 | **F** | 5427 | 5433 | VTDFNAI | 0.977 |
| 5431 | **N** | 5428 | 5434 | TDFNAIA | 0.954 |
| 5432 | **A** | 5429 | 5435 | DFNAIAT | 0.935 |
| 5433 | **I** | 5430 | 5436 | FNAIATC | 0.924 |
| 5434 | **A** | 5431 | 5437 | NAIATCD | 0.93 |
| 5435 | **T** | 5432 | 5438 | AIATCDW | 0.933 |
| 5436 | **C** | 5433 | 5439 | IATCDWT | 0.944 |
| 5437 | **D** | 5434 | 5440 | ATCDWTN | 0.957 |
| 5438 | **W** | 5435 | 5441 | TCDWTNA | 0.972 |
| 5439 | **T** | 5436 | 5442 | CDWTNAG | 0.997 |
| 5440 | **N** | 5437 | 5443 | DWTNAGD | 1.016 |
| 5441 | **A** | 5438 | 5444 | WTNAGDY | 1.028 |
| 5442 | **G** | 5439 | 5445 | TNAGDYI | 1.022 |
| 5443 | **D** | 5440 | 5446 | NAGDYIL | 1.002 |
| 5444 | **Y** | 5441 | 5447 | AGDYILA | 0.97 |
| 5445 | **I** | 5442 | 5448 | GDYILAN | 0.947 |
| 5446 | **L** | 5443 | 5449 | DYILANT | 0.947 |
| 5447 | **A** | 5444 | 5450 | YILANTC | 0.956 |
| 5448 | **N** | 5445 | 5451 | ILANTCT | 0.981 |
| 5449 | **T** | 5446 | 5452 | LANTCTE | 1.007 |
| 5450 | **C** | 5447 | 5453 | ANTCTER | 1.02 |
| 5451 | **T** | 5448 | 5454 | NTCTERL | 1.031 |
| 5452 | **E** | 5449 | 5455 | TCTERLK | 1.036 |
| 5453 | **R** | 5450 | 5456 | CTERLKL | 1.027 |
| 5454 | **L** | 5451 | 5457 | TERLKLF | 1.012 |
| 5455 | **K** | 5452 | 5458 | ERLKLFA | 0.993 |
| 5456 | **L** | 5453 | 5459 | RLKLFAA | 0.966 |
| 5457 | **F** | 5454 | 5460 | LKLFAAE | 0.952 |
| 5458 | **A** | 5455 | 5461 | KLFAAET | 0.953 |
| 5459 | **A** | 5456 | 5462 | LFAAETL | 0.964 |
| 5460 | **E** | 5457 | 5463 | FAAETLK | 0.989 |
| 5461 | **T** | 5458 | 5464 | AAETLKA | 1.01 |
| 5462 | **L** | 5459 | 5465 | AETLKAT | 1.021 |
| 5463 | **K** | 5460 | 5466 | ETLKATE | 1.037 |
| 5464 | **A** | 5461 | 5467 | TLKATEE | 1.047 |
| 5465 | **T** | 5462 | 5468 | LKATEET | 1.058 |
| 5466 | **E** | 5463 | 5469 | KATEETF | 1.061 |
| 5467 | **E** | 5464 | 5470 | ATEETFK | 1.054 |
| 5468 | **T** | 5465 | 5471 | TEETFKL | 1.037 |
| 5469 | **F** | 5466 | 5472 | EETFKLS | 1.011 |
| 5470 | **K** | 5467 | 5473 | ETFKLSY | 0.995 |
| 5471 | **L** | 5468 | 5474 | TFKLSYG | 0.973 |
| 5472 | **S** | 5469 | 5475 | FKLSYGI | 0.954 |
| 5473 | **Y** | 5470 | 5476 | KLSYGIA | 0.944 |
| 5474 | **G** | 5471 | 5477 | LSYGIAT | 0.931 |
| 5475 | **I** | 5472 | 5478 | SYGIATV | 0.929 |
| 5476 | **A** | 5473 | 5479 | YGIATVR | 0.941 |
| 5477 | **T** | 5474 | 5480 | GIATVRE | 0.957 |
| 5478 | **V** | 5475 | 5481 | IATVREV | 0.975 |
| 5479 | **R** | 5476 | 5482 | ATVREVL | 0.987 |
| 5480 | **E** | 5477 | 5483 | TVREVLS | 0.992 |
| 5481 | **V** | 5478 | 5484 | VREVLSD | 0.992 |
| 5482 | **L** | 5479 | 5485 | REVLSDR | 0.998 |
| 5483 | **S** | 5480 | 5486 | EVLSDRE | 1.011 |
| 5484 | **D** | 5481 | 5487 | VLSDREL | 1.017 |
| 5485 | **R** | 5482 | 5488 | LSDRELH | 1.014 |
| 5486 | **E** | 5483 | 5489 | SDRELHL | 1.0 |
| 5487 | **L** | 5484 | 5490 | DRELHLS | 0.974 |
| 5488 | **H** | 5485 | 5491 | RELHLSW | 0.951 |
| 5489 | **L** | 5486 | 5492 | ELHLSWE | 0.94 |
| 5490 | **S** | 5487 | 5493 | LHLSWEV | 0.934 |
| 5491 | **W** | 5488 | 5494 | HLSWEVG | 0.943 |
| 5492 | **E** | 5489 | 5495 | LSWEVGK | 0.965 |
| 5493 | **V** | 5490 | 5496 | SWEVGKP | 0.992 |
| 5494 | **G** | 5491 | 5497 | WEVGKPR | 1.021 |
| 5495 | **K** | 5492 | 5498 | EVGKPRP | 1.043 |
| 5496 | **P** | 5493 | 5499 | VGKPRPP | 1.055 |
| 5497 | **R** | 5494 | 5500 | GKPRPPL | 1.053 |
| 5498 | **P** | 5495 | 5501 | KPRPPLN | 1.046 |
| 5499 | **P** | 5496 | 5502 | PRPPLNR | 1.037 |
| 5500 | **L** | 5497 | 5503 | RPPLNRN | 1.023 |
| 5501 | **N** | 5498 | 5504 | PPLNRNY | 1.013 |
| 5502 | **R** | 5499 | 5505 | PLNRNYV | 0.999 |
| 5503 | **N** | 5500 | 5506 | LNRNYVF | 0.978 |
| 5504 | **Y** | 5501 | 5507 | NRNYVFT | 0.965 |
| 5505 | **V** | 5502 | 5508 | RNYVFTG | 0.961 |
| 5506 | **F** | 5503 | 5509 | NYVFTGY | 0.966 |
| 5507 | **T** | 5504 | 5510 | YVFTGYR | 0.978 |
| 5508 | **G** | 5505 | 5511 | VFTGYRV | 0.983 |
| 5509 | **Y** | 5506 | 5512 | FTGYRVT | 0.981 |
| 5510 | **R** | 5507 | 5513 | TGYRVTK | 0.985 |
| 5511 | **V** | 5508 | 5514 | GYRVTKN | 1.003 |
| 5512 | **T** | 5509 | 5515 | YRVTKNS | 1.037 |
| 5513 | **K** | 5510 | 5516 | RVTKNSK | 1.073 |
| 5514 | **N** | 5511 | 5517 | VTKNSKV | 1.093 |
| 5515 | **S** | 5512 | 5518 | TKNSKVQ | 1.085 |
| 5516 | **K** | 5513 | 5519 | KNSKVQI | 1.055 |
| 5517 | **V** | 5514 | 5520 | NSKVQIG | 1.021 |
| 5518 | **Q** | 5515 | 5521 | SKVQIGE | 0.997 |
| 5519 | **I** | 5516 | 5522 | KVQIGEY | 0.992 |
| 5520 | **G** | 5517 | 5523 | VQIGEYT | 0.993 |
| 5521 | **E** | 5518 | 5524 | QIGEYTF | 0.99 |
| 5522 | **Y** | 5519 | 5525 | IGEYTFE | 0.983 |
| 5523 | **T** | 5520 | 5526 | GEYTFEK | 0.98 |
| 5524 | **F** | 5521 | 5527 | EYTFEKG | 0.995 |
| 5525 | **E** | 5522 | 5528 | YTFEKGD | 1.026 |
| 5526 | **K** | 5523 | 5529 | TFEKGDY | 1.052 |
| 5527 | **G** | 5524 | 5530 | FEKGDYG | 1.068 |
| 5528 | **D** | 5525 | 5531 | EKGDYGD | 1.066 |
| 5529 | **Y** | 5526 | 5532 | KGDYGDA | 1.046 |
| 5530 | **G** | 5527 | 5533 | GDYGDAV | 1.028 |
| 5531 | **D** | 5528 | 5534 | DYGDAVV | 1.004 |
| 5532 | **A** | 5529 | 5535 | YGDAVVY | 0.974 |
| 5533 | **V** | 5530 | 5536 | GDAVVYR | 0.959 |
| 5534 | **V** | 5531 | 5537 | DAVVYRG | 0.96 |
| 5535 | **Y** | 5532 | 5538 | AVVYRGT | 0.98 |
| 5536 | **R** | 5533 | 5539 | VVYRGTT | 1.018 |
| 5537 | **G** | 5534 | 5540 | VYRGTTT | 1.053 |
| 5538 | **T** | 5535 | 5541 | YRGTTTY | 1.062 |
| 5539 | **T** | 5536 | 5542 | RGTTTYK | 1.06 |
| 5540 | **T** | 5537 | 5543 | GTTTYKL | 1.042 |
| 5541 | **Y** | 5538 | 5544 | TTTYKLN | 1.016 |
| 5542 | **K** | 5539 | 5545 | TTYKLNV | 1.002 |
| 5543 | **L** | 5540 | 5546 | TYKLNVG | 0.988 |
| 5544 | **N** | 5541 | 5547 | YKLNVGD | 0.988 |
| 5545 | **V** | 5542 | 5548 | KLNVGDY | 0.996 |
| 5546 | **G** | 5543 | 5549 | LNVGDYF | 0.999 |
| 5547 | **D** | 5544 | 5550 | NVGDYFV | 0.994 |
| 5548 | **Y** | 5545 | 5551 | VGDYFVL | 0.974 |
| 5549 | **F** | 5546 | 5552 | GDYFVLT | 0.961 |
| 5550 | **V** | 5547 | 5553 | DYFVLTS | 0.961 |
| 5551 | **L** | 5548 | 5554 | YFVLTSH | 0.973 |
| 5552 | **T** | 5549 | 5555 | FVLTSHT | 0.992 |
| 5553 | **S** | 5550 | 5556 | VLTSHTV | 0.995 |
| 5554 | **H** | 5551 | 5557 | LTSHTVM | 0.978 |
| 5555 | **T** | 5552 | 5558 | TSHTVMP | 0.954 |
| 5556 | **V** | 5553 | 5559 | SHTVMPL | 0.934 |
| 5557 | **M** | 5554 | 5560 | HTVMPLS | 0.921 |
| 5558 | **P** | 5555 | 5561 | TVMPLSA | 0.931 |
| 5559 | **L** | 5556 | 5562 | VMPLSAP | 0.953 |
| 5560 | **S** | 5557 | 5563 | MPLSAPT | 0.979 |
| 5561 | **A** | 5558 | 5564 | PLSAPTL | 1.007 |
| 5562 | **P** | 5559 | 5565 | LSAPTLV | 1.017 |
| 5563 | **T** | 5560 | 5566 | SAPTLVP | 1.018 |
| 5564 | **L** | 5561 | 5567 | APTLVPQ | 1.02 |
| 5565 | **V** | 5562 | 5568 | PTLVPQE | 1.025 |
| 5566 | **P** | 5563 | 5569 | TLVPQEH | 1.038 |
| 5567 | **Q** | 5564 | 5570 | LVPQEHY | 1.036 |
| 5568 | **E** | 5565 | 5571 | VPQEHYV | 1.012 |
| 5569 | **H** | 5566 | 5572 | PQEHYVR | 0.975 |
| 5570 | **Y** | 5567 | 5573 | QEHYVRI | 0.939 |
| 5571 | **V** | 5568 | 5574 | EHYVRIT | 0.932 |
| 5572 | **R** | 5569 | 5575 | HYVRITG | 0.948 |
| 5573 | **I** | 5570 | 5576 | YVRITGL | 0.975 |
| 5574 | **T** | 5571 | 5577 | VRITGLY | 0.995 |
| 5575 | **G** | 5572 | 5578 | RITGLYP | 1.003 |
| 5576 | **L** | 5573 | 5579 | ITGLYPT | 1.005 |
| 5577 | **Y** | 5574 | 5580 | TGLYPTL | 1.004 |
| 5578 | **P** | 5575 | 5581 | GLYPTLN | 1.007 |
| 5579 | **T** | 5576 | 5582 | LYPTLNI | 1.003 |
| 5580 | **L** | 5577 | 5583 | YPTLNIS | 0.998 |
| 5581 | **N** | 5578 | 5584 | PTLNISD | 0.996 |
| 5582 | **I** | 5579 | 5585 | TLNISDE | 1.002 |
| 5583 | **S** | 5580 | 5586 | LNISDEF | 1.008 |
| 5584 | **D** | 5581 | 5587 | NISDEFS | 1.014 |
| 5585 | **E** | 5582 | 5588 | ISDEFSS | 1.025 |
| 5586 | **F** | 5583 | 5589 | SDEFSSN | 1.027 |
| 5587 | **S** | 5584 | 5590 | DEFSSNV | 1.033 |
| 5588 | **S** | 5585 | 5591 | EFSSNVA | 1.033 |
| 5589 | **N** | 5586 | 5592 | FSSNVAN | 1.01 |
| 5590 | **V** | 5587 | 5593 | SSNVANY | 0.986 |
| 5591 | **A** | 5588 | 5594 | SNVANYQ | 0.968 |
| 5592 | **N** | 5589 | 5595 | NVANYQK | 0.965 |
| 5593 | **Y** | 5590 | 5596 | VANYQKV | 0.98 |
| 5594 | **Q** | 5591 | 5597 | ANYQKVG | 0.996 |
| 5595 | **K** | 5592 | 5598 | NYQKVGM | 1.0 |
| 5596 | **V** | 5593 | 5599 | YQKVGMQ | 0.993 |
| 5597 | **G** | 5594 | 5600 | QKVGMQK | 0.988 |
| 5598 | **M** | 5595 | 5601 | KVGMQKY | 0.988 |
| 5599 | **Q** | 5596 | 5602 | VGMQKYS | 1.0 |
| 5600 | **K** | 5597 | 5603 | GMQKYST | 1.016 |
| 5601 | **Y** | 5598 | 5604 | MQKYSTL | 1.019 |
| 5602 | **S** | 5599 | 5605 | QKYSTLQ | 1.024 |
| 5603 | **T** | 5600 | 5606 | KYSTLQG | 1.028 |
| 5604 | **L** | 5601 | 5607 | YSTLQGP | 1.031 |
| 5605 | **Q** | 5602 | 5608 | STLQGPP | 1.047 |
| 5606 | **G** | 5603 | 5609 | TLQGPPG | 1.066 |
| 5607 | **P** | 5604 | 5610 | LQGPPGT | 1.074 |
| 5608 | **P** | 5605 | 5611 | QGPPGTG | 1.088 |
| 5609 | **G** | 5606 | 5612 | GPPGTGK | 1.099 |
| 5610 | **T** | 5607 | 5613 | PPGTGKS | 1.096 |
| 5611 | **G** | 5608 | 5614 | PGTGKSH | 1.091 |
| 5612 | **K** | 5609 | 5615 | GTGKSHF | 1.066 |
| 5613 | **S** | 5610 | 5616 | TGKSHFA | 1.025 |
| 5614 | **H** | 5611 | 5617 | GKSHFAI | 0.981 |
| 5615 | **F** | 5612 | 5618 | KSHFAIG | 0.943 |
| 5616 | **A** | 5613 | 5619 | SHFAIGL | 0.922 |
| 5617 | **I** | 5614 | 5620 | HFAIGLA | 0.913 |
| 5618 | **G** | 5615 | 5621 | FAIGLAL | 0.916 |
| 5619 | **L** | 5616 | 5622 | AIGLALY | 0.915 |
| 5620 | **A** | 5617 | 5623 | IGLALYY | 0.91 |
| 5621 | **L** | 5618 | 5624 | GLALYYP | 0.916 |
| 5622 | **Y** | 5619 | 5625 | LALYYPS | 0.929 |
| 5623 | **Y** | 5620 | 5626 | ALYYPSA | 0.96 |
| 5624 | **P** | 5621 | 5627 | LYYPSAR | 0.991 |
| 5625 | **S** | 5622 | 5628 | YYPSARI | 0.998 |
| 5626 | **A** | 5623 | 5629 | YPSARIV | 0.988 |
| 5627 | **R** | 5624 | 5630 | PSARIVY | 0.959 |
| 5628 | **I** | 5625 | 5631 | SARIVYT | 0.933 |
| 5629 | **V** | 5626 | 5632 | ARIVYTA | 0.923 |
| 5630 | **Y** | 5627 | 5633 | RIVYTAC | 0.92 |
| 5631 | **T** | 5628 | 5634 | IVYTACS | 0.923 |
| 5632 | **A** | 5629 | 5635 | VYTACSH | 0.924 |
| 5633 | **C** | 5630 | 5636 | YTACSHA | 0.92 |
| 5634 | **S** | 5631 | 5637 | TACSHAA | 0.918 |
| 5635 | **H** | 5632 | 5638 | ACSHAAV | 0.917 |
| 5636 | **A** | 5633 | 5639 | CSHAAVD | 0.913 |
| 5637 | **A** | 5634 | 5640 | SHAAVDA | 0.916 |
| 5638 | **V** | 5635 | 5641 | HAAVDAL | 0.921 |
| 5639 | **D** | 5636 | 5642 | AAVDALC | 0.921 |
| 5640 | **A** | 5637 | 5643 | AVDALCE | 0.93 |
| 5641 | **L** | 5638 | 5644 | VDALCEK | 0.944 |
| 5642 | **C** | 5639 | 5645 | DALCEKA | 0.957 |
| 5643 | **E** | 5640 | 5646 | ALCEKAL | 0.979 |
| 5644 | **K** | 5641 | 5647 | LCEKALK | 0.996 |
| 5645 | **A** | 5642 | 5648 | CEKALKY | 0.994 |
| 5646 | **L** | 5643 | 5649 | EKALKYL | 0.992 |
| 5647 | **K** | 5644 | 5650 | KALKYLP | 0.983 |
| 5648 | **Y** | 5645 | 5651 | ALKYLPI | 0.969 |
| 5649 | **L** | 5646 | 5652 | LKYLPID | 0.975 |
| 5650 | **P** | 5647 | 5653 | KYLPIDK | 0.987 |
| 5651 | **I** | 5648 | 5654 | YLPIDKC | 1.003 |
| 5652 | **D** | 5649 | 5655 | LPIDKCS | 1.025 |
| 5653 | **K** | 5650 | 5656 | PIDKCSR | 1.033 |
| 5654 | **C** | 5651 | 5657 | IDKCSRI | 1.022 |
| 5655 | **S** | 5652 | 5658 | DKCSRII | 1.005 |
| 5656 | **R** | 5653 | 5659 | KCSRIIP | 0.978 |
| 5657 | **I** | 5654 | 5660 | CSRIIPA | 0.956 |
| 5658 | **I** | 5655 | 5661 | SRIIPAR | 0.945 |
| 5659 | **P** | 5656 | 5662 | RIIPARA | 0.949 |
| 5660 | **A** | 5657 | 5663 | IIPARAR | 0.958 |
| 5661 | **R** | 5658 | 5664 | IPARARV | 0.962 |
| 5662 | **A** | 5659 | 5665 | PARARVE | 0.968 |
| 5663 | **R** | 5660 | 5666 | ARARVEC | 0.954 |
| 5664 | **V** | 5661 | 5667 | RARVECF | 0.943 |
| 5665 | **E** | 5662 | 5668 | ARVECFD | 0.942 |
| 5666 | **C** | 5663 | 5669 | RVECFDK | 0.948 |
| 5667 | **F** | 5664 | 5670 | VECFDKF | 0.968 |
| 5668 | **D** | 5665 | 5671 | ECFDKFK | 0.997 |
| 5669 | **K** | 5666 | 5672 | CFDKFKV | 1.011 |
| 5670 | **F** | 5667 | 5673 | FDKFKVN | 1.012 |
| 5671 | **K** | 5668 | 5674 | DKFKVNS | 1.025 |
| 5672 | **V** | 5669 | 5675 | KFKVNST | 1.028 |
| 5673 | **N** | 5670 | 5676 | FKVNSTL | 1.037 |
| 5674 | **S** | 5671 | 5677 | KVNSTLE | 1.052 |
| 5675 | **T** | 5672 | 5678 | VNSTLEQ | 1.044 |
| 5676 | **L** | 5673 | 5679 | NSTLEQY | 1.029 |
| 5677 | **E** | 5674 | 5680 | STLEQYV | 1.011 |
| 5678 | **Q** | 5675 | 5681 | TLEQYVF | 0.983 |
| 5679 | **Y** | 5676 | 5682 | LEQYVFC | 0.956 |
| 5680 | **V** | 5677 | 5683 | EQYVFCT | 0.935 |
| 5681 | **F** | 5678 | 5684 | QYVFCTV | 0.923 |
| 5682 | **C** | 5679 | 5685 | YVFCTVN | 0.919 |
| 5683 | **T** | 5680 | 5686 | VFCTVNA | 0.929 |
| 5684 | **V** | 5681 | 5687 | FCTVNAL | 0.94 |
| 5685 | **N** | 5682 | 5688 | CTVNALP | 0.954 |
| 5686 | **A** | 5683 | 5689 | TVNALPE | 0.976 |
| 5687 | **L** | 5684 | 5690 | VNALPET | 1.002 |
| 5688 | **P** | 5685 | 5691 | NALPETT | 1.033 |
| 5689 | **E** | 5686 | 5692 | ALPETTA | 1.054 |
| 5690 | **T** | 5687 | 5693 | LPETTAD | 1.054 |
| 5691 | **T** | 5688 | 5694 | PETTADI | 1.036 |
| 5692 | **A** | 5689 | 5695 | ETTADIV | 1.003 |
| 5693 | **D** | 5690 | 5696 | TTADIVV | 0.965 |
| 5694 | **I** | 5691 | 5697 | TADIVVF | 0.936 |
| 5695 | **V** | 5692 | 5698 | ADIVVFD | 0.93 |
| 5696 | **V** | 5693 | 5699 | DIVVFDE | 0.941 |
| 5697 | **F** | 5694 | 5700 | IVVFDEI | 0.965 |
| 5698 | **D** | 5695 | 5701 | VVFDEIS | 0.992 |
| 5699 | **E** | 5696 | 5702 | VFDEISM | 0.991 |
| 5700 | **I** | 5697 | 5703 | FDEISMA | 0.978 |
| 5701 | **S** | 5698 | 5704 | DEISMAT | 0.962 |
| 5702 | **M** | 5699 | 5705 | EISMATN | 0.95 |
| 5703 | **A** | 5700 | 5706 | ISMATNY | 0.959 |
| 5704 | **T** | 5701 | 5707 | SMATNYD | 0.973 |
| 5705 | **N** | 5702 | 5708 | MATNYDL | 0.978 |
| 5706 | **Y** | 5703 | 5709 | ATNYDLS | 0.973 |
| 5707 | **D** | 5704 | 5710 | TNYDLSV | 0.959 |
| 5708 | **L** | 5705 | 5711 | NYDLSVV | 0.946 |
| 5709 | **S** | 5706 | 5712 | YDLSVVN | 0.936 |
| 5710 | **V** | 5707 | 5713 | DLSVVNA | 0.939 |
| 5711 | **V** | 5708 | 5714 | LSVVNAR | 0.942 |
| 5712 | **N** | 5709 | 5715 | SVVNARL | 0.948 |
| 5713 | **A** | 5710 | 5716 | VVNARLR | 0.955 |
| 5714 | **R** | 5711 | 5717 | VNARLRA | 0.954 |
| 5715 | **L** | 5712 | 5718 | NARLRAK | 0.964 |
| 5716 | **R** | 5713 | 5719 | ARLRAKH | 0.971 |
| 5717 | **A** | 5714 | 5720 | RLRAKHY | 0.976 |
| 5718 | **K** | 5715 | 5721 | LRAKHYV | 0.973 |
| 5719 | **H** | 5716 | 5722 | RAKHYVY | 0.946 |
| 5720 | **Y** | 5717 | 5723 | AKHYVYI | 0.916 |
| 5721 | **V** | 5718 | 5724 | KHYVYIG | 0.904 |
| 5722 | **Y** | 5719 | 5725 | HYVYIGD | 0.907 |
| 5723 | **I** | 5720 | 5726 | YVYIGDP | 0.939 |
| 5724 | **G** | 5721 | 5727 | VYIGDPA | 0.984 |
| 5725 | **D** | 5722 | 5728 | YIGDPAQ | 1.006 |
| 5726 | **P** | 5723 | 5729 | IGDPAQL | 1.017 |
| 5727 | **A** | 5724 | 5730 | GDPAQLP | 1.003 |
| 5728 | **Q** | 5725 | 5731 | DPAQLPA | 0.98 |
| 5729 | **L** | 5726 | 5732 | PAQLPAP | 0.978 |
| 5730 | **P** | 5727 | 5733 | AQLPAPR | 0.986 |
| 5731 | **A** | 5728 | 5734 | QLPAPRT | 1.01 |
| 5732 | **P** | 5729 | 5735 | LPAPRTL | 1.029 |
| 5733 | **R** | 5730 | 5736 | PAPRTLL | 1.029 |
| 5734 | **T** | 5731 | 5737 | APRTLLT | 1.024 |
| 5735 | **L** | 5732 | 5738 | PRTLLTK | 1.015 |
| 5736 | **L** | 5733 | 5739 | RTLLTKG | 1.022 |
| 5737 | **T** | 5734 | 5740 | TLLTKGT | 1.042 |
| 5738 | **K** | 5735 | 5741 | LLTKGTL | 1.056 |
| 5739 | **G** | 5736 | 5742 | LTKGTLE | 1.065 |
| 5740 | **T** | 5737 | 5743 | TKGTLEP | 1.056 |
| 5741 | **L** | 5738 | 5744 | KGTLEPE | 1.041 |
| 5742 | **E** | 5739 | 5745 | GTLEPEY | 1.028 |
| 5743 | **P** | 5740 | 5746 | TLEPEYF | 1.012 |
| 5744 | **E** | 5741 | 5747 | LEPEYFN | 0.998 |
| 5745 | **Y** | 5742 | 5748 | EPEYFNS | 0.986 |
| 5746 | **F** | 5743 | 5749 | PEYFNSV | 0.975 |
| 5747 | **N** | 5744 | 5750 | EYFNSVC | 0.971 |
| 5748 | **S** | 5745 | 5751 | YFNSVCR | 0.963 |
| 5749 | **V** | 5746 | 5752 | FNSVCRL | 0.948 |
| 5750 | **C** | 5747 | 5753 | NSVCRLM | 0.93 |
| 5751 | **R** | 5748 | 5754 | SVCRLMK | 0.927 |
| 5752 | **L** | 5749 | 5755 | VCRLMKT | 0.939 |
| 5753 | **M** | 5750 | 5756 | CRLMKTI | 0.96 |
| 5754 | **K** | 5751 | 5757 | RLMKTIG | 0.996 |
| 5755 | **T** | 5752 | 5758 | LMKTIGP | 1.018 |
| 5756 | **I** | 5753 | 5759 | MKTIGPD | 1.032 |
| 5757 | **G** | 5754 | 5760 | KTIGPDM | 1.035 |
| 5758 | **P** | 5755 | 5761 | TIGPDMF | 1.019 |
| 5759 | **D** | 5756 | 5762 | IGPDMFL | 0.999 |
| 5760 | **M** | 5757 | 5763 | GPDMFLG | 0.973 |
| 5761 | **F** | 5758 | 5764 | PDMFLGT | 0.968 |
| 5762 | **L** | 5759 | 5765 | DMFLGTC | 0.974 |
| 5763 | **G** | 5760 | 5766 | MFLGTCR | 0.99 |
| 5764 | **T** | 5761 | 5767 | FLGTCRR | 1.008 |
| 5765 | **C** | 5762 | 5768 | LGTCRRC | 1.009 |
| 5766 | **R** | 5763 | 5769 | GTCRRCP | 1.004 |
| 5767 | **R** | 5764 | 5770 | TCRRCPA | 0.997 |
| 5768 | **C** | 5765 | 5771 | CRRCPAE | 0.984 |
| 5769 | **P** | 5766 | 5772 | RRCPAEI | 0.973 |
| 5770 | **A** | 5767 | 5773 | RCPAEIV | 0.964 |
| 5771 | **E** | 5768 | 5774 | CPAEIVD | 0.956 |
| 5772 | **I** | 5769 | 5775 | PAEIVDT | 0.962 |
| 5773 | **V** | 5770 | 5776 | AEIVDTV | 0.978 |
| 5774 | **D** | 5771 | 5777 | EIVDTVS | 0.993 |
| 5775 | **T** | 5772 | 5778 | IVDTVSA | 0.997 |
| 5776 | **V** | 5773 | 5779 | VDTVSAL | 0.985 |
| 5777 | **S** | 5774 | 5780 | DTVSALV | 0.964 |
| 5778 | **A** | 5775 | 5781 | TVSALVY | 0.943 |
| 5779 | **L** | 5776 | 5782 | VSALVYD | 0.94 |
| 5780 | **V** | 5777 | 5783 | SALVYDN | 0.957 |
| 5781 | **Y** | 5778 | 5784 | ALVYDNK | 0.989 |
| 5782 | **D** | 5779 | 5785 | LVYDNKL | 1.023 |
| 5783 | **N** | 5780 | 5786 | VYDNKLK | 1.047 |
| 5784 | **K** | 5781 | 5787 | YDNKLKA | 1.047 |
| 5785 | **L** | 5782 | 5788 | DNKLKAH | 1.028 |
| 5786 | **K** | 5783 | 5789 | NKLKAHK | 1.015 |
| 5787 | **A** | 5784 | 5790 | KLKAHKD | 1.002 |
| 5788 | **H** | 5785 | 5791 | LKAHKDK | 1.008 |
| 5789 | **K** | 5786 | 5792 | KAHKDKS | 1.029 |
| 5790 | **D** | 5787 | 5793 | AHKDKSA | 1.04 |
| 5791 | **K** | 5788 | 5794 | HKDKSAQ | 1.044 |
| 5792 | **S** | 5789 | 5795 | KDKSAQC | 1.024 |
| 5793 | **A** | 5790 | 5796 | DKSAQCF | 0.991 |
| 5794 | **Q** | 5791 | 5797 | KSAQCFK | 0.96 |
| 5795 | **C** | 5792 | 5798 | SAQCFKM | 0.938 |
| 5796 | **F** | 5793 | 5799 | AQCFKMF | 0.931 |
| 5797 | **K** | 5794 | 5800 | QCFKMFY | 0.934 |
| 5798 | **M** | 5795 | 5801 | CFKMFYK | 0.938 |
| 5799 | **F** | 5796 | 5802 | FKMFYKG | 0.954 |
| 5800 | **Y** | 5797 | 5803 | KMFYKGV | 0.969 |
| 5801 | **K** | 5798 | 5804 | MFYKGVI | 0.98 |
| 5802 | **G** | 5799 | 5805 | FYKGVIT | 0.98 |
| 5803 | **V** | 5800 | 5806 | YKGVITH | 0.966 |
| 5804 | **I** | 5801 | 5807 | KGVITHD | 0.951 |
| 5805 | **T** | 5802 | 5808 | GVITHDV | 0.944 |
| 5806 | **H** | 5803 | 5809 | VITHDVS | 0.953 |
| 5807 | **D** | 5804 | 5810 | ITHDVSS | 0.97 |
| 5808 | **V** | 5805 | 5811 | THDVSSA | 0.988 |
| 5809 | **S** | 5806 | 5812 | HDVSSAI | 0.994 |
| 5810 | **S** | 5807 | 5813 | DVSSAIN | 0.991 |
| 5811 | **A** | 5808 | 5814 | VSSAINR | 0.983 |
| 5812 | **I** | 5809 | 5815 | SSAINRP | 0.981 |
| 5813 | **N** | 5810 | 5816 | SAINRPQ | 0.993 |
| 5814 | **R** | 5811 | 5817 | AINRPQI | 1.008 |
| 5815 | **P** | 5812 | 5818 | INRPQIG | 1.016 |
| 5816 | **Q** | 5813 | 5819 | NRPQIGV | 1.009 |
| 5817 | **I** | 5814 | 5820 | RPQIGVV | 0.987 |
| 5818 | **G** | 5815 | 5821 | PQIGVVR | 0.967 |
| 5819 | **V** | 5816 | 5822 | QIGVVRE | 0.961 |
| 5820 | **V** | 5817 | 5823 | IGVVREF | 0.963 |
| 5821 | **R** | 5818 | 5824 | GVVREFL | 0.973 |
| 5822 | **E** | 5819 | 5825 | VVREFLT | 0.982 |
| 5823 | **F** | 5820 | 5826 | VREFLTR | 0.985 |
| 5824 | **L** | 5821 | 5827 | REFLTRN | 1.002 |
| 5825 | **T** | 5822 | 5828 | EFLTRNP | 1.024 |
| 5826 | **R** | 5823 | 5829 | FLTRNPA | 1.038 |
| 5827 | **N** | 5824 | 5830 | LTRNPAW | 1.044 |
| 5828 | **P** | 5825 | 5831 | TRNPAWR | 1.032 |
| 5829 | **A** | 5826 | 5832 | RNPAWRK | 1.013 |
| 5830 | **W** | 5827 | 5833 | NPAWRKA | 0.999 |
| 5831 | **R** | 5828 | 5834 | PAWRKAV | 0.993 |
| 5832 | **K** | 5829 | 5835 | AWRKAVF | 0.986 |
| 5833 | **A** | 5830 | 5836 | WRKAVFI | 0.966 |
| 5834 | **V** | 5831 | 5837 | RKAVFIS | 0.954 |
| 5835 | **F** | 5832 | 5838 | KAVFISP | 0.952 |
| 5836 | **I** | 5833 | 5839 | AVFISPY | 0.96 |
| 5837 | **S** | 5834 | 5840 | VFISPYN | 0.987 |
| 5838 | **P** | 5835 | 5841 | FISPYNS | 1.015 |
| 5839 | **Y** | 5836 | 5842 | ISPYNSQ | 1.038 |
| 5840 | **N** | 5837 | 5843 | SPYNSQN | 1.06 |
| 5841 | **S** | 5838 | 5844 | PYNSQNA | 1.072 |
| 5842 | **Q** | 5839 | 5845 | YNSQNAV | 1.06 |
| 5843 | **N** | 5840 | 5846 | NSQNAVA | 1.03 |
| 5844 | **A** | 5841 | 5847 | SQNAVAS | 0.999 |
| 5845 | **V** | 5842 | 5848 | QNAVASK | 0.98 |
| 5846 | **A** | 5843 | 5849 | NAVASKI | 0.976 |
| 5847 | **S** | 5844 | 5850 | AVASKIL | 0.987 |
| 5848 | **K** | 5845 | 5851 | VASKILG | 0.987 |
| 5849 | **I** | 5846 | 5852 | ASKILGL | 0.972 |
| 5850 | **L** | 5847 | 5853 | SKILGLP | 0.97 |
| 5851 | **G** | 5848 | 5854 | KILGLPT | 0.974 |
| 5852 | **L** | 5849 | 5855 | ILGLPTQ | 1.001 |
| 5853 | **P** | 5850 | 5856 | LGLPTQT | 1.04 |
| 5854 | **T** | 5851 | 5857 | GLPTQTV | 1.061 |
| 5855 | **Q** | 5852 | 5858 | LPTQTVD | 1.076 |
| 5856 | **T** | 5853 | 5859 | PTQTVDS | 1.076 |
| 5857 | **V** | 5854 | 5860 | TQTVDSS | 1.079 |
| 5858 | **D** | 5855 | 5861 | QTVDSSQ | 1.099 |
| 5859 | **S** | 5856 | 5862 | TVDSSQG | 1.121 |
| 5860 | **S** | 5857 | 5863 | VDSSQGS | 1.143 |
| 5861 | **Q** | 5858 | 5864 | DSSQGSE | 1.15 |
| 5862 | **G** | 5859 | 5865 | SSQGSEY | 1.132 |
| 5863 | **S** | 5860 | 5866 | SQGSEYD | 1.098 |
| 5864 | **E** | 5861 | 5867 | QGSEYDY | 1.049 |
| 5865 | **Y** | 5862 | 5868 | GSEYDYV | 1.0 |
| 5866 | **D** | 5863 | 5869 | SEYDYVI | 0.959 |
| 5867 | **Y** | 5864 | 5870 | EYDYVIF | 0.929 |
| 5868 | **V** | 5865 | 5871 | YDYVIFT | 0.922 |
| 5869 | **I** | 5866 | 5872 | DYVIFTQ | 0.939 |
| 5870 | **F** | 5867 | 5873 | YVIFTQT | 0.973 |
| 5871 | **T** | 5868 | 5874 | VIFTQTT | 1.019 |
| 5872 | **Q** | 5869 | 5875 | IFTQTTE | 1.06 |
| 5873 | **T** | 5870 | 5876 | FTQTTET | 1.079 |
| 5874 | **T** | 5871 | 5877 | TQTTETA | 1.076 |
| 5875 | **E** | 5872 | 5878 | QTTETAH | 1.056 |
| 5876 | **T** | 5873 | 5879 | TTETAHS | 1.023 |
| 5877 | **A** | 5874 | 5880 | TETAHSC | 0.991 |
| 5878 | **H** | 5875 | 5881 | ETAHSCN | 0.966 |
| 5879 | **S** | 5876 | 5882 | TAHSCNV | 0.951 |
| 5880 | **C** | 5877 | 5883 | AHSCNVN | 0.951 |
| 5881 | **N** | 5878 | 5884 | HSCNVNR | 0.962 |
| 5882 | **V** | 5879 | 5885 | SCNVNRF | 0.973 |
| 5883 | **N** | 5880 | 5886 | CNVNRFN | 0.979 |
| 5884 | **R** | 5881 | 5887 | NVNRFNV | 0.975 |
| 5885 | **F** | 5882 | 5888 | VNRFNVA | 0.958 |
| 5886 | **N** | 5883 | 5889 | NRFNVAI | 0.939 |
| 5887 | **V** | 5884 | 5890 | RFNVAIT | 0.931 |
| 5888 | **A** | 5885 | 5891 | FNVAITR | 0.934 |
| 5889 | **I** | 5886 | 5892 | NVAITRA | 0.955 |
| 5890 | **T** | 5887 | 5893 | VAITRAK | 0.988 |
| 5891 | **R** | 5888 | 5894 | AITRAKV | 1.01 |
| 5892 | **A** | 5889 | 5895 | ITRAKVG | 1.019 |
| 5893 | **K** | 5890 | 5896 | TRAKVGI | 1.009 |
| 5894 | **V** | 5891 | 5897 | RAKVGIL | 0.98 |
| 5895 | **G** | 5892 | 5898 | AKVGILC | 0.952 |
| 5896 | **I** | 5893 | 5899 | KVGILCI | 0.928 |
| 5897 | **L** | 5894 | 5900 | VGILCIM | 0.911 |
| 5898 | **C** | 5895 | 5901 | GILCIMS | 0.91 |
| 5899 | **I** | 5896 | 5902 | ILCIMSD | 0.919 |
| 5900 | **M** | 5897 | 5903 | LCIMSDR | 0.943 |
| 5901 | **S** | 5898 | 5904 | CIMSDRD | 0.982 |
| 5902 | **D** | 5899 | 5905 | IMSDRDL | 1.008 |
| 5903 | **R** | 5900 | 5906 | MSDRDLY | 1.02 |
| 5904 | **D** | 5901 | 5907 | SDRDLYD | 1.026 |
| 5905 | **L** | 5902 | 5908 | DRDLYDK | 1.017 |
| 5906 | **Y** | 5903 | 5909 | RDLYDKL | 1.014 |
| 5907 | **D** | 5904 | 5910 | DLYDKLQ | 1.014 |
| 5908 | **K** | 5905 | 5911 | LYDKLQF | 1.001 |
| 5909 | **L** | 5906 | 5912 | YDKLQFT | 0.987 |
| 5910 | **Q** | 5907 | 5913 | DKLQFTS | 0.977 |
| 5911 | **F** | 5908 | 5914 | KLQFTSL | 0.975 |
| 5912 | **T** | 5909 | 5915 | LQFTSLE | 0.984 |
| 5913 | **S** | 5910 | 5916 | QFTSLEI | 0.991 |
| 5914 | **L** | 5911 | 5917 | FTSLEIP | 0.996 |
| 5915 | **E** | 5912 | 5918 | TSLEIPR | 1.0 |
| 5916 | **I** | 5913 | 5919 | SLEIPRR | 1.01 |
| 5917 | **P** | 5914 | 5920 | LEIPRRN | 1.023 |
| 5918 | **R** | 5915 | 5921 | EIPRRNV | 1.025 |
| 5919 | **R** | 5916 | 5922 | IPRRNVA | 1.016 |
| 5920 | **N** | 5917 | 5923 | PRRNVAT | 0.994 |
| 5921 | **V** | 5918 | 5924 | RRNVATL | 0.97 |
| 5922 | **A** | 5919 | 5925 | RNVATLQ | 0.952 |
| 5923 | **T** | 5920 | 5926 | NVATLQA | 0.947 |
| 5924 | **L** | 5921 | 5927 | VATLQAE | 0.954 |
| 5925 | **Q** | 5922 | 5928 | ATLQAEN | 0.966 |
| 5926 | **A** | 5923 | 5929 | TLQAENV | 0.987 |
| 5927 | **E** | 5924 | 5930 | LQAENVT | 1.002 |
| 5928 | **N** | 5925 | 5931 | QAENVTG | 1.012 |
| 5929 | **V** | 5926 | 5932 | AENVTGL | 1.016 |
| 5930 | **T** | 5927 | 5933 | ENVTGLF | 1.01 |
| 5931 | **G** | 5928 | 5934 | NVTGLFK | 1.005 |
| 5932 | **L** | 5929 | 5935 | VTGLFKD | 1.003 |
| 5933 | **F** | 5930 | 5936 | TGLFKDC | 1.003 |
| 5934 | **K** | 5931 | 5937 | GLFKDCS | 1.016 |
| 5935 | **D** | 5932 | 5938 | LFKDCSK | 1.028 |
| 5936 | **C** | 5933 | 5939 | FKDCSKV | 1.026 |
| 5937 | **S** | 5934 | 5940 | KDCSKVI | 1.02 |
| 5938 | **K** | 5935 | 5941 | DCSKVIT | 1.006 |
| 5939 | **V** | 5936 | 5942 | CSKVITG | 0.989 |
| 5940 | **I** | 5937 | 5943 | SKVITGL | 0.985 |
| 5941 | **T** | 5938 | 5944 | KVITGLH | 0.989 |
| 5942 | **G** | 5939 | 5945 | VITGLHP | 0.994 |
| 5943 | **L** | 5940 | 5946 | ITGLHPT | 1.004 |
| 5944 | **H** | 5941 | 5947 | TGLHPTQ | 1.016 |
| 5945 | **P** | 5942 | 5948 | GLHPTQA | 1.03 |
| 5946 | **T** | 5943 | 5949 | LHPTQAP | 1.041 |
| 5947 | **Q** | 5944 | 5950 | HPTQAPT | 1.05 |
| 5948 | **A** | 5945 | 5951 | PTQAPTH | 1.049 |
| 5949 | **P** | 5946 | 5952 | TQAPTHL | 1.038 |
| 5950 | **T** | 5947 | 5953 | QAPTHLS | 1.017 |
| 5951 | **H** | 5948 | 5954 | APTHLSV | 0.993 |
| 5952 | **L** | 5949 | 5955 | PTHLSVD | 0.982 |
| 5953 | **S** | 5950 | 5956 | THLSVDT | 0.983 |
| 5954 | **V** | 5951 | 5957 | HLSVDTK | 1.004 |
| 5955 | **D** | 5952 | 5958 | LSVDTKF | 1.026 |
| 5956 | **T** | 5953 | 5959 | SVDTKFK | 1.04 |
| 5957 | **K** | 5954 | 5960 | VDTKFKT | 1.046 |
| 5958 | **F** | 5955 | 5961 | DTKFKTE | 1.043 |
| 5959 | **K** | 5956 | 5962 | TKFKTEG | 1.05 |
| 5960 | **T** | 5957 | 5963 | KFKTEGL | 1.05 |
| 5961 | **E** | 5958 | 5964 | FKTEGLC | 1.041 |
| 5962 | **G** | 5959 | 5965 | KTEGLCV | 1.021 |
| 5963 | **L** | 5960 | 5966 | TEGLCVD | 0.987 |
| 5964 | **C** | 5961 | 5967 | EGLCVDI | 0.963 |
| 5965 | **V** | 5962 | 5968 | GLCVDIP | 0.958 |
| 5966 | **D** | 5963 | 5969 | LCVDIPG | 0.971 |
| 5967 | **I** | 5964 | 5970 | CVDIPGI | 0.995 |
| 5968 | **P** | 5965 | 5971 | VDIPGIP | 1.022 |
| 5969 | **G** | 5966 | 5972 | DIPGIPK | 1.039 |
| 5970 | **I** | 5967 | 5973 | IPGIPKD | 1.052 |
| 5971 | **P** | 5968 | 5974 | PGIPKDM | 1.057 |
| 5972 | **K** | 5969 | 5975 | GIPKDMT | 1.048 |
| 5973 | **D** | 5970 | 5976 | IPKDMTY | 1.03 |
| 5974 | **M** | 5971 | 5977 | PKDMTYR | 1.005 |
| 5975 | **T** | 5972 | 5978 | KDMTYRR | 0.988 |
| 5976 | **Y** | 5973 | 5979 | DMTYRRL | 0.983 |
| 5977 | **R** | 5974 | 5980 | MTYRRLI | 0.982 |
| 5978 | **R** | 5975 | 5981 | TYRRLIS | 0.978 |
| 5979 | **L** | 5976 | 5982 | YRRLISM | 0.958 |
| 5980 | **I** | 5977 | 5983 | RRLISMM | 0.931 |
| 5981 | **S** | 5978 | 5984 | RLISMMG | 0.909 |
| 5982 | **M** | 5979 | 5985 | LISMMGF | 0.895 |
| 5983 | **M** | 5980 | 5986 | ISMMGFK | 0.904 |
| 5984 | **G** | 5981 | 5987 | SMMGFKM | 0.923 |
| 5985 | **F** | 5982 | 5988 | MMGFKMN | 0.942 |
| 5986 | **K** | 5983 | 5989 | MGFKMNY | 0.962 |
| 5987 | **M** | 5984 | 5990 | GFKMNYQ | 0.959 |
| 5988 | **N** | 5985 | 5991 | FKMNYQV | 0.952 |
| 5989 | **Y** | 5986 | 5992 | KMNYQVN | 0.951 |
| 5990 | **Q** | 5987 | 5993 | MNYQVNG | 0.952 |
| 5991 | **V** | 5988 | 5994 | NYQVNGY | 0.969 |
| 5992 | **N** | 5989 | 5995 | YQVNGYP | 0.99 |
| 5993 | **G** | 5990 | 5996 | QVNGYPN | 1.006 |
| 5994 | **Y** | 5991 | 5997 | VNGYPNM | 1.006 |
| 5995 | **P** | 5992 | 5998 | NGYPNMF | 0.998 |
| 5996 | **N** | 5993 | 5999 | GYPNMFI | 0.972 |
| 5997 | **M** | 5994 | 6000 | YPNMFIT | 0.948 |
| 5998 | **F** | 5995 | 6001 | PNMFITR | 0.947 |
| 5999 | **I** | 5996 | 6002 | NMFITRE | 0.96 |
| 6000 | **T** | 5997 | 6003 | MFITREE | 0.994 |
| 6001 | **R** | 5998 | 6004 | FITREEA | 1.019 |
| 6002 | **E** | 5999 | 6005 | ITREEAI | 1.024 |
| 6003 | **E** | 6000 | 6006 | TREEAIR | 1.005 |
| 6004 | **A** | 6001 | 6007 | REEAIRH | 0.972 |
| 6005 | **I** | 6002 | 6008 | EEAIRHV | 0.944 |
| 6006 | **R** | 6003 | 6009 | EAIRHVR | 0.926 |
| 6007 | **H** | 6004 | 6010 | AIRHVRA | 0.923 |
| 6008 | **V** | 6005 | 6011 | IRHVRAW | 0.916 |
| 6009 | **R** | 6006 | 6012 | RHVRAWI | 0.908 |
| 6010 | **A** | 6007 | 6013 | HVRAWIG | 0.901 |
| 6011 | **W** | 6008 | 6014 | VRAWIGF | 0.889 |
| 6012 | **I** | 6009 | 6015 | RAWIGFD | 0.896 |
| 6013 | **G** | 6010 | 6016 | AWIGFDV | 0.91 |
| 6014 | **F** | 6011 | 6017 | WIGFDVE | 0.929 |
| 6015 | **D** | 6012 | 6018 | IGFDVEG | 0.957 |
| 6016 | **V** | 6013 | 6019 | GFDVEGC | 0.974 |
| 6017 | **E** | 6014 | 6020 | FDVEGCH | 0.979 |
| 6018 | **G** | 6015 | 6021 | DVEGCHA | 0.971 |
| 6019 | **C** | 6016 | 6022 | VEGCHAT | 0.957 |
| 6020 | **H** | 6017 | 6023 | EGCHATR | 0.957 |
| 6021 | **A** | 6018 | 6024 | GCHATRE | 0.971 |
| 6022 | **T** | 6019 | 6025 | CHATREA | 0.99 |
| 6023 | **R** | 6020 | 6026 | HATREAV | 1.001 |
| 6024 | **E** | 6021 | 6027 | ATREAVG | 1.003 |
| 6025 | **A** | 6022 | 6028 | TREAVGT | 0.997 |
| 6026 | **V** | 6023 | 6029 | REAVGTN | 0.996 |
| 6027 | **G** | 6024 | 6030 | EAVGTNL | 1.005 |
| 6028 | **T** | 6025 | 6031 | AVGTNLP | 1.006 |
| 6029 | **N** | 6026 | 6032 | VGTNLPL | 0.999 |
| 6030 | **L** | 6027 | 6033 | GTNLPLQ | 0.981 |
| 6031 | **P** | 6028 | 6034 | TNLPLQL | 0.96 |
| 6032 | **L** | 6029 | 6035 | NLPLQLG | 0.945 |
| 6033 | **Q** | 6030 | 6036 | LPLQLGF | 0.934 |
| 6034 | **L** | 6031 | 6037 | PLQLGFS | 0.942 |
| 6035 | **G** | 6032 | 6038 | LQLGFST | 0.956 |
| 6036 | **F** | 6033 | 6039 | QLGFSTG | 0.977 |
| 6037 | **S** | 6034 | 6040 | LGFSTGV | 1.005 |
| 6038 | **T** | 6035 | 6041 | GFSTGVN | 1.015 |
| 6039 | **G** | 6036 | 6042 | FSTGVNL | 1.011 |
| 6040 | **V** | 6037 | 6043 | STGVNLV | 0.992 |
| 6041 | **N** | 6038 | 6044 | TGVNLVA | 0.964 |
| 6042 | **L** | 6039 | 6045 | GVNLVAV | 0.943 |
| 6043 | **V** | 6040 | 6046 | VNLVAVP | 0.937 |
| 6044 | **A** | 6041 | 6047 | NLVAVPT | 0.949 |
| 6045 | **V** | 6042 | 6048 | LVAVPTG | 0.976 |
| 6046 | **P** | 6043 | 6049 | VAVPTGY | 1.003 |
| 6047 | **T** | 6044 | 6050 | AVPTGYV | 1.013 |
| 6048 | **G** | 6045 | 6051 | VPTGYVD | 1.014 |
| 6049 | **Y** | 6046 | 6052 | PTGYVDT | 1.007 |
| 6050 | **V** | 6047 | 6053 | TGYVDTP | 1.008 |
| 6051 | **D** | 6048 | 6054 | GYVDTPN | 1.03 |
| 6052 | **T** | 6049 | 6055 | YVDTPNN | 1.054 |
| 6053 | **P** | 6050 | 6056 | VDTPNNT | 1.075 |
| 6054 | **N** | 6051 | 6057 | DTPNNTD | 1.091 |
| 6055 | **N** | 6052 | 6058 | TPNNTDF | 1.083 |
| 6056 | **T** | 6053 | 6059 | PNNTDFS | 1.069 |
| 6057 | **D** | 6054 | 6060 | NNTDFSR | 1.052 |
| 6058 | **F** | 6055 | 6061 | NTDFSRV | 1.027 |
| 6059 | **S** | 6056 | 6062 | TDFSRVS | 1.013 |
| 6060 | **R** | 6057 | 6063 | DFSRVSA | 1.002 |
| 6061 | **V** | 6058 | 6064 | FSRVSAK | 0.998 |
| 6062 | **S** | 6059 | 6065 | SRVSAKP | 1.005 |
| 6063 | **A** | 6060 | 6066 | RVSAKPP | 1.021 |
| 6064 | **K** | 6061 | 6067 | VSAKPPP | 1.038 |
| 6065 | **P** | 6062 | 6068 | SAKPPPG | 1.055 |
| 6066 | **P** | 6063 | 6069 | AKPPPGD | 1.067 |
| 6067 | **P** | 6064 | 6070 | KPPPGDQ | 1.069 |
| 6068 | **G** | 6065 | 6071 | PPPGDQF | 1.061 |
| 6069 | **D** | 6066 | 6072 | PPGDQFK | 1.045 |
| 6070 | **Q** | 6067 | 6073 | PGDQFKH | 1.025 |
| 6071 | **F** | 6068 | 6074 | GDQFKHL | 1.0 |
| 6072 | **K** | 6069 | 6075 | DQFKHLI | 0.981 |
| 6073 | **H** | 6070 | 6076 | QFKHLIP | 0.959 |
| 6074 | **L** | 6071 | 6077 | FKHLIPL | 0.943 |
| 6075 | **I** | 6072 | 6078 | KHLIPLM | 0.926 |
| 6076 | **P** | 6073 | 6079 | HLIPLMY | 0.914 |
| 6077 | **L** | 6074 | 6080 | LIPLMYK | 0.919 |
| 6078 | **M** | 6075 | 6081 | IPLMYKG | 0.928 |
| 6079 | **Y** | 6076 | 6082 | PLMYKGL | 0.955 |
| 6080 | **K** | 6077 | 6083 | LMYKGLP | 0.98 |
| 6081 | **G** | 6078 | 6084 | MYKGLPW | 0.986 |
| 6082 | **L** | 6079 | 6085 | YKGLPWN | 0.979 |
| 6083 | **P** | 6080 | 6086 | KGLPWNV | 0.96 |
| 6084 | **W** | 6081 | 6087 | GLPWNVV | 0.94 |
| 6085 | **N** | 6082 | 6088 | LPWNVVR | 0.929 |
| 6086 | **V** | 6083 | 6089 | PWNVVRI | 0.929 |
| 6087 | **V** | 6084 | 6090 | WNVVRIK | 0.94 |
| 6088 | **R** | 6085 | 6091 | NVVRIKI | 0.949 |
| 6089 | **I** | 6086 | 6092 | VVRIKIV | 0.961 |
| 6090 | **K** | 6087 | 6093 | VRIKIVQ | 0.961 |
| 6091 | **I** | 6088 | 6094 | RIKIVQM | 0.941 |
| 6092 | **V** | 6089 | 6095 | IKIVQML | 0.928 |
| 6093 | **Q** | 6090 | 6096 | KIVQMLS | 0.92 |
| 6094 | **M** | 6091 | 6097 | IVQMLSD | 0.929 |
| 6095 | **L** | 6092 | 6098 | VQMLSDT | 0.962 |
| 6096 | **S** | 6093 | 6099 | QMLSDTL | 0.991 |
| 6097 | **D** | 6094 | 6100 | MLSDTLK | 1.014 |
| 6098 | **T** | 6095 | 6101 | LSDTLKN | 1.027 |
| 6099 | **L** | 6096 | 6102 | SDTLKNL | 1.022 |
| 6100 | **K** | 6097 | 6103 | DTLKNLS | 1.023 |
| 6101 | **N** | 6098 | 6104 | TLKNLSD | 1.018 |
| 6102 | **L** | 6099 | 6105 | LKNLSDR | 1.016 |
| 6103 | **S** | 6100 | 6106 | KNLSDRV | 1.017 |
| 6104 | **D** | 6101 | 6107 | NLSDRVV | 1.004 |
| 6105 | **R** | 6102 | 6108 | LSDRVVF | 0.987 |
| 6106 | **V** | 6103 | 6109 | SDRVVFV | 0.962 |
| 6107 | **V** | 6104 | 6110 | DRVVFVL | 0.938 |
| 6108 | **F** | 6105 | 6111 | RVVFVLW | 0.916 |
| 6109 | **V** | 6106 | 6112 | VVFVLWA | 0.9 |
| 6110 | **L** | 6107 | 6113 | VFVLWAH | 0.894 |
| 6111 | **W** | 6108 | 6114 | FVLWAHG | 0.889 |
| 6112 | **A** | 6109 | 6115 | VLWAHGF | 0.898 |
| 6113 | **H** | 6110 | 6116 | LWAHGFE | 0.911 |
| 6114 | **G** | 6111 | 6117 | WAHGFEL | 0.922 |
| 6115 | **F** | 6112 | 6118 | AHGFELT | 0.942 |
| 6116 | **E** | 6113 | 6119 | HGFELTS | 0.961 |
| 6117 | **L** | 6114 | 6120 | GFELTSM | 0.978 |
| 6118 | **T** | 6115 | 6121 | FELTSMK | 1.0 |
| 6119 | **S** | 6116 | 6122 | ELTSMKY | 1.006 |
| 6120 | **M** | 6117 | 6123 | LTSMKYF | 0.997 |
| 6121 | **K** | 6118 | 6124 | TSMKYFV | 0.985 |
| 6122 | **Y** | 6119 | 6125 | SMKYFVK | 0.968 |
| 6123 | **F** | 6120 | 6126 | MKYFVKI | 0.963 |
| 6124 | **V** | 6121 | 6127 | KYFVKIG | 0.974 |
| 6125 | **K** | 6122 | 6128 | YFVKIGP | 0.994 |
| 6126 | **I** | 6123 | 6129 | FVKIGPE | 1.017 |
| 6127 | **G** | 6124 | 6130 | VKIGPER | 1.038 |
| 6128 | **P** | 6125 | 6131 | KIGPERT | 1.051 |
| 6129 | **E** | 6126 | 6132 | IGPERTC | 1.045 |
| 6130 | **R** | 6127 | 6133 | GPERTCC | 1.026 |
| 6131 | **T** | 6128 | 6134 | PERTCCL | 0.998 |
| 6132 | **C** | 6129 | 6135 | ERTCCLC | 0.958 |
| 6133 | **C** | 6130 | 6136 | RTCCLCD | 0.943 |
| 6134 | **L** | 6131 | 6137 | TCCLCDR | 0.945 |
| 6135 | **C** | 6132 | 6138 | CCLCDRR | 0.961 |
| 6136 | **D** | 6133 | 6139 | CLCDRRA | 0.998 |
| 6137 | **R** | 6134 | 6140 | LCDRRAT | 1.012 |
| 6138 | **R** | 6135 | 6141 | CDRRATC | 1.009 |
| 6139 | **A** | 6136 | 6142 | DRRATCF | 0.993 |
| 6140 | **T** | 6137 | 6143 | RRATCFS | 0.966 |
| 6141 | **C** | 6138 | 6144 | RATCFST | 0.957 |
| 6142 | **F** | 6139 | 6145 | ATCFSTA | 0.967 |
| 6143 | **S** | 6140 | 6146 | TCFSTAS | 0.994 |
| 6144 | **T** | 6141 | 6147 | CFSTASD | 1.019 |
| 6145 | **A** | 6142 | 6148 | FSTASDT | 1.037 |
| 6146 | **S** | 6143 | 6149 | STASDTY | 1.037 |
| 6147 | **D** | 6144 | 6150 | TASDTYA | 1.02 |
| 6148 | **T** | 6145 | 6151 | ASDTYAC | 0.996 |
| 6149 | **Y** | 6146 | 6152 | SDTYACW | 0.957 |
| 6150 | **A** | 6147 | 6153 | DTYACWH | 0.923 |
| 6151 | **C** | 6148 | 6154 | TYACWHH | 0.902 |
| 6152 | **W** | 6149 | 6155 | YACWHHS | 0.888 |
| 6153 | **H** | 6150 | 6156 | ACWHHSI | 0.902 |
| 6154 | **H** | 6151 | 6157 | CWHHSIG | 0.92 |
| 6155 | **S** | 6152 | 6158 | WHHSIGF | 0.933 |
| 6156 | **I** | 6153 | 6159 | HHSIGFD | 0.946 |
| 6157 | **G** | 6154 | 6160 | HSIGFDY | 0.943 |
| 6158 | **F** | 6155 | 6161 | SIGFDYV | 0.937 |
| 6159 | **D** | 6156 | 6162 | IGFDYVY | 0.932 |
| 6160 | **Y** | 6157 | 6163 | GFDYVYN | 0.931 |
| 6161 | **V** | 6158 | 6164 | FDYVYNP | 0.945 |
| 6162 | **Y** | 6159 | 6165 | DYVYNPF | 0.959 |
| 6163 | **N** | 6160 | 6166 | YVYNPFM | 0.966 |
| 6164 | **P** | 6161 | 6167 | VYNPFMI | 0.96 |
| 6165 | **F** | 6162 | 6168 | YNPFMID | 0.936 |
| 6166 | **M** | 6163 | 6169 | NPFMIDV | 0.916 |
| 6167 | **I** | 6164 | 6170 | PFMIDVQ | 0.917 |
| 6168 | **D** | 6165 | 6171 | FMIDVQQ | 0.935 |
| 6169 | **V** | 6166 | 6172 | MIDVQQW | 0.961 |
| 6170 | **Q** | 6167 | 6173 | IDVQQWG | 0.979 |
| 6171 | **Q** | 6168 | 6174 | DVQQWGF | 0.978 |
| 6172 | **W** | 6169 | 6175 | VQQWGFT | 0.969 |
| 6173 | **G** | 6170 | 6176 | QQWGFTG | 0.974 |
| 6174 | **F** | 6171 | 6177 | QWGFTGN | 0.988 |
| 6175 | **T** | 6172 | 6178 | WGFTGNL | 1.011 |
| 6176 | **G** | 6173 | 6179 | GFTGNLQ | 1.03 |
| 6177 | **N** | 6174 | 6180 | FTGNLQS | 1.038 |
| 6178 | **L** | 6175 | 6181 | TGNLQSN | 1.041 |
| 6179 | **Q** | 6176 | 6182 | GNLQSNH | 1.042 |
| 6180 | **S** | 6177 | 6183 | NLQSNHD | 1.038 |
| 6181 | **N** | 6178 | 6184 | LQSNHDL | 1.02 |
| 6182 | **H** | 6179 | 6185 | QSNHDLY | 0.992 |
| 6183 | **D** | 6180 | 6186 | SNHDLYC | 0.956 |
| 6184 | **L** | 6181 | 6187 | NHDLYCQ | 0.923 |
| 6185 | **Y** | 6182 | 6188 | HDLYCQV | 0.9 |
| 6186 | **C** | 6183 | 6189 | DLYCQVH | 0.896 |
| 6187 | **Q** | 6184 | 6190 | LYCQVHG | 0.909 |
| 6188 | **V** | 6185 | 6191 | YCQVHGN | 0.931 |
| 6189 | **H** | 6186 | 6192 | CQVHGNA | 0.958 |
| 6190 | **G** | 6187 | 6193 | QVHGNAH | 0.973 |
| 6191 | **N** | 6188 | 6194 | VHGNAHV | 0.97 |
| 6192 | **A** | 6189 | 6195 | HGNAHVA | 0.956 |
| 6193 | **H** | 6190 | 6196 | GNAHVAS | 0.939 |
| 6194 | **V** | 6191 | 6197 | NAHVASC | 0.93 |
| 6195 | **A** | 6192 | 6198 | AHVASCD | 0.93 |
| 6196 | **S** | 6193 | 6199 | HVASCDA | 0.934 |
| 6197 | **C** | 6194 | 6200 | VASCDAI | 0.936 |
| 6198 | **D** | 6195 | 6201 | ASCDAIM | 0.928 |
| 6199 | **A** | 6196 | 6202 | SCDAIMT | 0.926 |
| 6200 | **I** | 6197 | 6203 | CDAIMTR | 0.929 |
| 6201 | **M** | 6198 | 6204 | DAIMTRC | 0.936 |
| 6202 | **T** | 6199 | 6205 | AIMTRCL | 0.953 |
| 6203 | **R** | 6200 | 6206 | IMTRCLA | 0.952 |
| 6204 | **C** | 6201 | 6207 | MTRCLAV | 0.939 |
| 6205 | **L** | 6202 | 6208 | TRCLAVH | 0.93 |
| 6206 | **A** | 6203 | 6209 | RCLAVHE | 0.918 |
| 6207 | **V** | 6204 | 6210 | CLAVHEC | 0.916 |
| 6208 | **H** | 6205 | 6211 | LAVHECF | 0.92 |
| 6209 | **E** | 6206 | 6212 | AVHECFV | 0.918 |
| 6210 | **C** | 6207 | 6213 | VHECFVK | 0.925 |
| 6211 | **F** | 6208 | 6214 | HECFVKR | 0.943 |
| 6212 | **V** | 6209 | 6215 | ECFVKRV | 0.964 |
| 6213 | **K** | 6210 | 6216 | CFVKRVD | 0.987 |
| 6214 | **R** | 6211 | 6217 | FVKRVDW | 0.991 |
| 6215 | **V** | 6212 | 6218 | VKRVDWT | 0.98 |
| 6216 | **D** | 6213 | 6219 | KRVDWTI | 0.966 |
| 6217 | **W** | 6214 | 6220 | RVDWTIE | 0.952 |
| 6218 | **T** | 6215 | 6221 | VDWTIEY | 0.949 |
| 6219 | **I** | 6216 | 6222 | DWTIEYP | 0.953 |
| 6220 | **E** | 6217 | 6223 | WTIEYPI | 0.948 |
| 6221 | **Y** | 6218 | 6224 | TIEYPII | 0.941 |
| 6222 | **P** | 6219 | 6225 | IEYPIIG | 0.936 |
| 6223 | **I** | 6220 | 6226 | EYPIIGD | 0.938 |
| 6224 | **I** | 6221 | 6227 | YPIIGDE | 0.956 |
| 6225 | **G** | 6222 | 6228 | PIIGDEL | 0.981 |
| 6226 | **D** | 6223 | 6229 | IIGDELK | 1.002 |
| 6227 | **E** | 6224 | 6230 | IGDELKI | 1.015 |
| 6228 | **L** | 6225 | 6231 | GDELKIN | 1.012 |
| 6229 | **K** | 6226 | 6232 | DELKINA | 1.003 |
| 6230 | **I** | 6227 | 6233 | ELKINAA | 0.983 |
| 6231 | **N** | 6228 | 6234 | LKINAAC | 0.957 |
| 6232 | **A** | 6229 | 6235 | KINAACR | 0.943 |
| 6233 | **A** | 6230 | 6236 | INAACRK | 0.94 |
| 6234 | **C** | 6231 | 6237 | NAACRKV | 0.952 |
| 6235 | **R** | 6232 | 6238 | AACRKVQ | 0.973 |
| 6236 | **K** | 6233 | 6239 | ACRKVQH | 0.983 |
| 6237 | **V** | 6234 | 6240 | CRKVQHM | 0.967 |
| 6238 | **Q** | 6235 | 6241 | RKVQHMV | 0.941 |
| 6239 | **H** | 6236 | 6242 | KVQHMVV | 0.917 |
| 6240 | **M** | 6237 | 6243 | VQHMVVK | 0.905 |
| 6241 | **V** | 6238 | 6244 | QHMVVKA | 0.918 |
| 6242 | **V** | 6239 | 6245 | HMVVKAA | 0.935 |
| 6243 | **K** | 6240 | 6246 | MVVKAAL | 0.949 |
| 6244 | **A** | 6241 | 6247 | VVKAALL | 0.948 |
| 6245 | **A** | 6242 | 6248 | VKAALLA | 0.937 |
| 6246 | **L** | 6243 | 6249 | KAALLAD | 0.941 |
| 6247 | **L** | 6244 | 6250 | AALLADK | 0.955 |
| 6248 | **A** | 6245 | 6251 | ALLADKF | 0.978 |
| 6249 | **D** | 6246 | 6252 | LLADKFP | 1.0 |
| 6250 | **K** | 6247 | 6253 | LADKFPV | 0.999 |
| 6251 | **F** | 6248 | 6254 | ADKFPVL | 0.979 |
| 6252 | **P** | 6249 | 6255 | DKFPVLH | 0.959 |
| 6253 | **V** | 6250 | 6256 | KFPVLHD | 0.94 |
| 6254 | **L** | 6251 | 6257 | FPVLHDI | 0.936 |
| 6255 | **H** | 6252 | 6258 | PVLHDIG | 0.95 |
| 6256 | **D** | 6253 | 6259 | VLHDIGN | 0.972 |
| 6257 | **I** | 6254 | 6260 | LHDIGNP | 1.003 |
| 6258 | **G** | 6255 | 6261 | HDIGNPK | 1.033 |
| 6259 | **N** | 6256 | 6262 | DIGNPKA | 1.05 |
| 6260 | **P** | 6257 | 6263 | IGNPKAI | 1.043 |
| 6261 | **K** | 6258 | 6264 | GNPKAIK | 1.028 |
| 6262 | **A** | 6259 | 6265 | NPKAIKC | 0.995 |
| 6263 | **I** | 6260 | 6266 | PKAIKCV | 0.967 |
| 6264 | **K** | 6261 | 6267 | KAIKCVP | 0.966 |
| 6265 | **C** | 6262 | 6268 | AIKCVPQ | 0.962 |
| 6266 | **V** | 6263 | 6269 | IKCVPQA | 0.981 |
| 6267 | **P** | 6264 | 6270 | KCVPQAD | 1.002 |
| 6268 | **Q** | 6265 | 6271 | CVPQADV | 1.004 |
| 6269 | **A** | 6266 | 6272 | VPQADVE | 1.002 |
| 6270 | **D** | 6267 | 6273 | PQADVEW | 0.983 |
| 6271 | **V** | 6268 | 6274 | QADVEWK | 0.971 |
| 6272 | **E** | 6269 | 6275 | ADVEWKF | 0.962 |
| 6273 | **W** | 6270 | 6276 | DVEWKFY | 0.957 |
| 6274 | **K** | 6271 | 6277 | VEWKFYD | 0.961 |
| 6275 | **F** | 6272 | 6278 | EWKFYDA | 0.956 |
| 6276 | **Y** | 6273 | 6279 | WKFYDAQ | 0.963 |
| 6277 | **D** | 6274 | 6280 | KFYDAQP | 0.979 |
| 6278 | **A** | 6275 | 6281 | FYDAQPC | 0.997 |
| 6279 | **Q** | 6276 | 6282 | YDAQPCS | 1.016 |
| 6280 | **P** | 6277 | 6283 | DAQPCSD | 1.028 |
| 6281 | **C** | 6278 | 6284 | AQPCSDK | 1.03 |
| 6282 | **S** | 6279 | 6285 | QPCSDKA | 1.03 |
| 6283 | **D** | 6280 | 6286 | PCSDKAY | 1.022 |
| 6284 | **K** | 6281 | 6287 | CSDKAYK | 1.015 |
| 6285 | **A** | 6282 | 6288 | SDKAYKI | 1.003 |
| 6286 | **Y** | 6283 | 6289 | DKAYKIE | 0.998 |
| 6287 | **K** | 6284 | 6290 | KAYKIEE | 1.007 |
| 6288 | **I** | 6285 | 6291 | AYKIEEL | 1.008 |
| 6289 | **E** | 6286 | 6292 | YKIEELF | 1.009 |
| 6290 | **E** | 6287 | 6293 | KIEELFY | 0.997 |
| 6291 | **L** | 6288 | 6294 | IEELFYS | 0.972 |
| 6292 | **F** | 6289 | 6295 | EELFYSY | 0.951 |
| 6293 | **Y** | 6290 | 6296 | ELFYSYA | 0.937 |
| 6294 | **S** | 6291 | 6297 | LFYSYAT | 0.93 |
| 6295 | **Y** | 6292 | 6298 | FYSYATH | 0.934 |
| 6296 | **A** | 6293 | 6299 | YSYATHS | 0.948 |
| 6297 | **T** | 6294 | 6300 | SYATHSD | 0.965 |
| 6298 | **H** | 6295 | 6301 | YATHSDK | 0.99 |
| 6299 | **S** | 6296 | 6302 | ATHSDKF | 1.009 |
| 6300 | **D** | 6297 | 6303 | THSDKFT | 1.021 |
| 6301 | **K** | 6298 | 6304 | HSDKFTD | 1.027 |
| 6302 | **F** | 6299 | 6305 | SDKFTDG | 1.021 |
| 6303 | **T** | 6300 | 6306 | DKFTDGV | 1.019 |
| 6304 | **D** | 6301 | 6307 | KFTDGVC | 1.008 |
| 6305 | **G** | 6302 | 6308 | FTDGVCL | 0.991 |
| 6306 | **V** | 6303 | 6309 | TDGVCLF | 0.968 |
| 6307 | **C** | 6304 | 6310 | DGVCLFW | 0.944 |
| 6308 | **L** | 6305 | 6311 | GVCLFWN | 0.929 |
| 6309 | **F** | 6306 | 6312 | VCLFWNC | 0.923 |
| 6310 | **W** | 6307 | 6313 | CLFWNCN | 0.926 |
| 6311 | **N** | 6308 | 6314 | LFWNCNV | 0.934 |
| 6312 | **C** | 6309 | 6315 | FWNCNVD | 0.951 |
| 6313 | **N** | 6310 | 6316 | WNCNVDR | 0.971 |
| 6314 | **V** | 6311 | 6317 | NCNVDRY | 0.991 |
| 6315 | **D** | 6312 | 6318 | CNVDRYP | 1.004 |
| 6316 | **R** | 6313 | 6319 | NVDRYPA | 1.004 |
| 6317 | **Y** | 6314 | 6320 | VDRYPAN | 0.998 |
| 6318 | **P** | 6315 | 6321 | DRYPANS | 0.996 |
| 6319 | **A** | 6316 | 6322 | RYPANSI | 0.995 |
| 6320 | **N** | 6317 | 6323 | YPANSIV | 0.988 |
| 6321 | **S** | 6318 | 6324 | PANSIVC | 0.975 |
| 6322 | **I** | 6319 | 6325 | ANSIVCR | 0.948 |
| 6323 | **V** | 6320 | 6326 | NSIVCRF | 0.925 |
| 6324 | **C** | 6321 | 6327 | SIVCRFD | 0.921 |
| 6325 | **R** | 6322 | 6328 | IVCRFDT | 0.937 |
| 6326 | **F** | 6323 | 6329 | VCRFDTR | 0.971 |
| 6327 | **D** | 6324 | 6330 | CRFDTRV | 1.002 |
| 6328 | **T** | 6325 | 6331 | RFDTRVL | 1.014 |
| 6329 | **R** | 6326 | 6332 | FDTRVLS | 1.012 |
| 6330 | **V** | 6327 | 6333 | DTRVLSN | 1.001 |
| 6331 | **L** | 6328 | 6334 | TRVLSNL | 0.992 |
| 6332 | **S** | 6329 | 6335 | RVLSNLN | 0.99 |
| 6333 | **N** | 6330 | 6336 | VLSNLNL | 0.984 |
| 6334 | **L** | 6331 | 6337 | LSNLNLP | 0.984 |
| 6335 | **N** | 6332 | 6338 | SNLNLPG | 0.987 |
| 6336 | **L** | 6333 | 6339 | NLNLPGC | 0.994 |
| 6337 | **P** | 6334 | 6340 | LNLPGCD | 1.013 |
| 6338 | **G** | 6335 | 6341 | NLPGCDG | 1.03 |
| 6339 | **C** | 6336 | 6342 | LPGCDGG | 1.05 |
| 6340 | **D** | 6337 | 6343 | PGCDGGS | 1.073 |
| 6341 | **G** | 6338 | 6344 | GCDGGSL | 1.08 |
| 6342 | **G** | 6339 | 6345 | CDGGSLY | 1.065 |
| 6343 | **S** | 6340 | 6346 | DGGSLYV | 1.03 |
| 6344 | **L** | 6341 | 6347 | GGSLYVN | 0.987 |
| 6345 | **Y** | 6342 | 6348 | GSLYVNK | 0.959 |
| 6346 | **V** | 6343 | 6349 | SLYVNKH | 0.958 |
| 6347 | **N** | 6344 | 6350 | LYVNKHA | 0.968 |
| 6348 | **K** | 6345 | 6351 | YVNKHAF | 0.974 |
| 6349 | **H** | 6346 | 6352 | VNKHAFH | 0.966 |
| 6350 | **A** | 6347 | 6353 | NKHAFHT | 0.956 |
| 6351 | **F** | 6348 | 6354 | KHAFHTP | 0.96 |
| 6352 | **H** | 6349 | 6355 | HAFHTPA | 0.972 |
| 6353 | **T** | 6350 | 6356 | AFHTPAF | 0.99 |
| 6354 | **P** | 6351 | 6357 | FHTPAFD | 1.004 |
| 6355 | **A** | 6352 | 6358 | HTPAFDK | 1.006 |
| 6356 | **F** | 6353 | 6359 | TPAFDKS | 1.013 |
| 6357 | **D** | 6354 | 6360 | PAFDKSA | 1.024 |
| 6358 | **K** | 6355 | 6361 | AFDKSAF | 1.022 |
| 6359 | **S** | 6356 | 6362 | FDKSAFV | 1.01 |
| 6360 | **A** | 6357 | 6363 | DKSAFVN | 0.983 |
| 6361 | **F** | 6358 | 6364 | KSAFVNL | 0.956 |
| 6362 | **V** | 6359 | 6365 | SAFVNLK | 0.95 |
| 6363 | **N** | 6360 | 6366 | AFVNLKQ | 0.961 |
| 6364 | **L** | 6361 | 6367 | FVNLKQL | 0.981 |
| 6365 | **K** | 6362 | 6368 | VNLKQLP | 0.998 |
| 6366 | **Q** | 6363 | 6369 | NLKQLPF | 0.994 |
| 6367 | **L** | 6364 | 6370 | LKQLPFF | 0.976 |
| 6368 | **P** | 6365 | 6371 | KQLPFFY | 0.948 |
| 6369 | **F** | 6366 | 6372 | QLPFFYY | 0.922 |
| 6370 | **F** | 6367 | 6373 | LPFFYYS | 0.915 |
| 6371 | **Y** | 6368 | 6374 | PFFYYSD | 0.923 |
| 6372 | **Y** | 6369 | 6375 | FFYYSDS | 0.959 |
| 6373 | **S** | 6370 | 6376 | FYYSDSP | 1.006 |
| 6374 | **D** | 6371 | 6377 | YYSDSPC | 1.038 |
| 6375 | **S** | 6372 | 6378 | YSDSPCE | 1.063 |
| 6376 | **P** | 6373 | 6379 | SDSPCES | 1.06 |
| 6377 | **C** | 6374 | 6380 | DSPCESH | 1.041 |
| 6378 | **E** | 6375 | 6381 | SPCESHG | 1.032 |
| 6379 | **S** | 6376 | 6382 | PCESHGK | 1.027 |
| 6380 | **H** | 6377 | 6383 | CESHGKQ | 1.028 |
| 6381 | **G** | 6378 | 6384 | ESHGKQV | 1.032 |
| 6382 | **K** | 6379 | 6385 | SHGKQVV | 1.023 |
| 6383 | **Q** | 6380 | 6386 | HGKQVVS | 1.009 |
| 6384 | **V** | 6381 | 6387 | GKQVVSD | 0.999 |
| 6385 | **V** | 6382 | 6388 | KQVVSDI | 0.998 |
| 6386 | **S** | 6383 | 6389 | QVVSDID | 1.004 |
| 6387 | **D** | 6384 | 6390 | VVSDIDY | 1.005 |
| 6388 | **I** | 6385 | 6391 | VSDIDYV | 0.993 |
| 6389 | **D** | 6386 | 6392 | SDIDYVP | 0.971 |
| 6390 | **Y** | 6387 | 6393 | DIDYVPL | 0.951 |
| 6391 | **V** | 6388 | 6394 | IDYVPLK | 0.949 |
| 6392 | **P** | 6389 | 6395 | DYVPLKS | 0.963 |
| 6393 | **L** | 6390 | 6396 | YVPLKSA | 0.989 |
| 6394 | **K** | 6391 | 6397 | VPLKSAT | 1.011 |
| 6395 | **S** | 6392 | 6398 | PLKSATC | 1.011 |
| 6396 | **A** | 6393 | 6399 | LKSATCI | 0.993 |
| 6397 | **T** | 6394 | 6400 | KSATCIT | 0.969 |
| 6398 | **C** | 6395 | 6401 | SATCITR | 0.953 |
| 6399 | **I** | 6396 | 6402 | ATCITRC | 0.955 |
| 6400 | **T** | 6397 | 6403 | TCITRCN | 0.969 |
| 6401 | **R** | 6398 | 6404 | CITRCNL | 0.977 |
| 6402 | **C** | 6399 | 6405 | ITRCNLG | 0.98 |
| 6403 | **N** | 6400 | 6406 | TRCNLGG | 0.983 |
| 6404 | **L** | 6401 | 6407 | RCNLGGA | 0.985 |
| 6405 | **G** | 6402 | 6408 | CNLGGAV | 0.989 |
| 6406 | **G** | 6403 | 6409 | NLGGAVC | 0.981 |
| 6407 | **A** | 6404 | 6410 | LGGAVCR | 0.96 |
| 6408 | **V** | 6405 | 6411 | GGAVCRH | 0.938 |
| 6409 | **C** | 6406 | 6412 | GAVCRHH | 0.918 |
| 6410 | **R** | 6407 | 6413 | AVCRHHA | 0.913 |
| 6411 | **H** | 6408 | 6414 | VCRHHAN | 0.922 |
| 6412 | **H** | 6409 | 6415 | CRHHANE | 0.937 |
| 6413 | **A** | 6410 | 6416 | RHHANEY | 0.958 |
| 6414 | **N** | 6411 | 6417 | HHANEYR | 0.971 |
| 6415 | **E** | 6412 | 6418 | HANEYRL | 0.975 |
| 6416 | **Y** | 6413 | 6419 | ANEYRLY | 0.961 |
| 6417 | **R** | 6414 | 6420 | NEYRLYL | 0.943 |
| 6418 | **L** | 6415 | 6421 | EYRLYLD | 0.929 |
| 6419 | **Y** | 6416 | 6422 | YRLYLDA | 0.918 |
| 6420 | **L** | 6417 | 6423 | RLYLDAY | 0.925 |
| 6421 | **D** | 6418 | 6424 | LYLDAYN | 0.93 |
| 6422 | **A** | 6419 | 6425 | YLDAYNM | 0.928 |
| 6423 | **Y** | 6420 | 6426 | LDAYNMM | 0.919 |
| 6424 | **N** | 6421 | 6427 | DAYNMMI | 0.901 |
| 6425 | **M** | 6422 | 6428 | AYNMMIS | 0.885 |
| 6426 | **M** | 6423 | 6429 | YNMMISA | 0.885 |
| 6427 | **I** | 6424 | 6430 | NMMISAG | 0.9 |
| 6428 | **S** | 6425 | 6431 | MMISAGF | 0.921 |
| 6429 | **A** | 6426 | 6432 | MISAGFS | 0.942 |
| 6430 | **G** | 6427 | 6433 | ISAGFSL | 0.947 |
| 6431 | **F** | 6428 | 6434 | SAGFSLW | 0.937 |
| 6432 | **S** | 6429 | 6435 | AGFSLWV | 0.923 |
| 6433 | **L** | 6430 | 6436 | GFSLWVY | 0.91 |
| 6434 | **W** | 6431 | 6437 | FSLWVYK | 0.909 |
| 6435 | **V** | 6432 | 6438 | SLWVYKQ | 0.93 |
| 6436 | **Y** | 6433 | 6439 | LWVYKQF | 0.954 |
| 6437 | **K** | 6434 | 6440 | WVYKQFD | 0.986 |
| 6438 | **Q** | 6435 | 6441 | VYKQFDT | 1.009 |
| 6439 | **F** | 6436 | 6442 | YKQFDTY | 1.014 |
| 6440 | **D** | 6437 | 6443 | KQFDTYN | 1.018 |
| 6441 | **T** | 6438 | 6444 | QFDTYNL | 1.004 |
| 6442 | **Y** | 6439 | 6445 | FDTYNLW | 0.983 |
| 6443 | **N** | 6440 | 6446 | DTYNLWN | 0.97 |
| 6444 | **L** | 6441 | 6447 | TYNLWNT | 0.964 |
| 6445 | **W** | 6442 | 6448 | YNLWNTF | 0.964 |
| 6446 | **N** | 6443 | 6449 | NLWNTFT | 0.981 |
| 6447 | **T** | 6444 | 6450 | LWNTFTR | 0.996 |
| 6448 | **F** | 6445 | 6451 | WNTFTRL | 0.999 |
| 6449 | **T** | 6446 | 6452 | NTFTRLQ | 1.009 |
| 6450 | **R** | 6447 | 6453 | TFTRLQS | 1.011 |
| 6451 | **L** | 6448 | 6454 | FTRLQSL | 1.008 |
| 6452 | **Q** | 6449 | 6455 | TRLQSLE | 1.015 |
| 6453 | **S** | 6450 | 6456 | RLQSLEN | 1.014 |
| 6454 | **L** | 6451 | 6457 | LQSLENV | 1.005 |
| 6455 | **E** | 6452 | 6458 | QSLENVA | 0.996 |
| 6456 | **N** | 6453 | 6459 | SLENVAF | 0.974 |
| 6457 | **V** | 6454 | 6460 | LENVAFN | 0.95 |
| 6458 | **A** | 6455 | 6461 | ENVAFNV | 0.932 |
| 6459 | **F** | 6456 | 6462 | NVAFNVV | 0.922 |
| 6460 | **N** | 6457 | 6463 | VAFNVVN | 0.926 |
| 6461 | **V** | 6458 | 6464 | AFNVVNK | 0.944 |
| 6462 | **V** | 6459 | 6465 | FNVVNKG | 0.969 |
| 6463 | **N** | 6460 | 6466 | NVVNKGH | 0.994 |
| 6464 | **K** | 6461 | 6467 | VVNKGHF | 1.007 |
| 6465 | **G** | 6462 | 6468 | VNKGHFD | 1.01 |
| 6466 | **H** | 6463 | 6469 | NKGHFDG | 1.011 |
| 6467 | **F** | 6464 | 6470 | KGHFDGQ | 1.025 |
| 6468 | **D** | 6465 | 6471 | GHFDGQQ | 1.06 |
| 6469 | **G** | 6466 | 6472 | HFDGQQG | 1.099 |
| 6470 | **Q** | 6467 | 6473 | FDGQQGE | 1.124 |
| 6471 | **Q** | 6468 | 6474 | DGQQGEV | 1.125 |
| 6472 | **G** | 6469 | 6475 | GQQGEVP | 1.096 |
| 6473 | **E** | 6470 | 6476 | QQGEVPV | 1.054 |
| 6474 | **V** | 6471 | 6477 | QGEVPVS | 1.01 |
| 6475 | **P** | 6472 | 6478 | GEVPVSI | 0.973 |
| 6476 | **V** | 6473 | 6479 | EVPVSII | 0.948 |
| 6477 | **S** | 6474 | 6480 | VPVSIIN | 0.934 |
| 6478 | **I** | 6475 | 6481 | PVSIINN | 0.94 |
| 6479 | **I** | 6476 | 6482 | VSIINNT | 0.961 |
| 6480 | **N** | 6477 | 6483 | SIINNTV | 0.987 |
| 6481 | **N** | 6478 | 6484 | IINNTVY | 1.006 |
| 6482 | **T** | 6479 | 6485 | INNTVYT | 1.009 |
| 6483 | **V** | 6480 | 6486 | NNTVYTK | 1.005 |
| 6484 | **Y** | 6481 | 6487 | NTVYTKV | 1.001 |
| 6485 | **T** | 6482 | 6488 | TVYTKVD | 1.012 |
| 6486 | **K** | 6483 | 6489 | VYTKVDG | 1.027 |
| 6487 | **V** | 6484 | 6490 | YTKVDGV | 1.034 |
| 6488 | **D** | 6485 | 6491 | TKVDGVD | 1.034 |
| 6489 | **G** | 6486 | 6492 | KVDGVDV | 1.017 |
| 6490 | **V** | 6487 | 6493 | VDGVDVE | 0.994 |
| 6491 | **D** | 6488 | 6494 | DGVDVEL | 0.976 |
| 6492 | **V** | 6489 | 6495 | GVDVELF | 0.96 |
| 6493 | **E** | 6490 | 6496 | VDVELFE | 0.954 |
| 6494 | **L** | 6491 | 6497 | DVELFEN | 0.967 |
| 6495 | **F** | 6492 | 6498 | VELFENK | 0.988 |
| 6496 | **E** | 6493 | 6499 | ELFENKT | 1.022 |
| 6497 | **N** | 6494 | 6500 | LFENKTT | 1.052 |
| 6498 | **K** | 6495 | 6501 | FENKTTL | 1.062 |
| 6499 | **T** | 6496 | 6502 | ENKTTLP | 1.054 |
| 6500 | **T** | 6497 | 6503 | NKTTLPV | 1.03 |
| 6501 | **L** | 6498 | 6504 | KTTLPVN | 0.997 |
| 6502 | **P** | 6499 | 6505 | TTLPVNV | 0.971 |
| 6503 | **V** | 6500 | 6506 | TLPVNVA | 0.953 |
| 6504 | **N** | 6501 | 6507 | LPVNVAF | 0.936 |
| 6505 | **V** | 6502 | 6508 | PVNVAFE | 0.927 |
| 6506 | **A** | 6503 | 6509 | VNVAFEL | 0.922 |
| 6507 | **F** | 6504 | 6510 | NVAFELW | 0.915 |
| 6508 | **E** | 6505 | 6511 | VAFELWA | 0.913 |
| 6509 | **L** | 6506 | 6512 | AFELWAK | 0.921 |
| 6510 | **W** | 6507 | 6513 | FELWAKR | 0.932 |
| 6511 | **A** | 6508 | 6514 | ELWAKRN | 0.961 |
| 6512 | **K** | 6509 | 6515 | LWAKRNI | 0.991 |
| 6513 | **R** | 6510 | 6516 | WAKRNIK | 1.012 |
| 6514 | **N** | 6511 | 6517 | AKRNIKP | 1.031 |
| 6515 | **I** | 6512 | 6518 | KRNIKPV | 1.036 |
| 6516 | **K** | 6513 | 6519 | RNIKPVP | 1.043 |
| 6517 | **P** | 6514 | 6520 | NIKPVPE | 1.047 |
| 6518 | **V** | 6515 | 6521 | IKPVPEV | 1.041 |
| 6519 | **P** | 6516 | 6522 | KPVPEVK | 1.042 |
| 6520 | **E** | 6517 | 6523 | PVPEVKI | 1.025 |
| 6521 | **V** | 6518 | 6524 | VPEVKIL | 1.006 |
| 6522 | **K** | 6519 | 6525 | PEVKILN | 0.996 |
| 6523 | **I** | 6520 | 6526 | EVKILNN | 0.978 |
| 6524 | **L** | 6521 | 6527 | VKILNNL | 0.976 |
| 6525 | **N** | 6522 | 6528 | KILNNLG | 0.976 |
| 6526 | **N** | 6523 | 6529 | ILNNLGV | 0.974 |
| 6527 | **L** | 6524 | 6530 | LNNLGVD | 0.97 |
| 6528 | **G** | 6525 | 6531 | NNLGVDI | 0.957 |
| 6529 | **V** | 6526 | 6532 | NLGVDIA | 0.944 |
| 6530 | **D** | 6527 | 6533 | LGVDIAA | 0.931 |
| 6531 | **I** | 6528 | 6534 | GVDIAAN | 0.927 |
| 6532 | **A** | 6529 | 6535 | VDIAANT | 0.937 |
| 6533 | **A** | 6530 | 6536 | DIAANTV | 0.951 |
| 6534 | **N** | 6531 | 6537 | IAANTVI | 0.964 |
| 6535 | **T** | 6532 | 6538 | AANTVIW | 0.966 |
| 6536 | **V** | 6533 | 6539 | ANTVIWD | 0.95 |
| 6537 | **I** | 6534 | 6540 | NTVIWDY | 0.935 |
| 6538 | **W** | 6535 | 6541 | TVIWDYK | 0.937 |
| 6539 | **D** | 6536 | 6542 | VIWDYKR | 0.952 |
| 6540 | **Y** | 6537 | 6543 | IWDYKRD | 0.985 |
| 6541 | **K** | 6538 | 6544 | WDYKRDA | 1.02 |
| 6542 | **R** | 6539 | 6545 | DYKRDAP | 1.033 |
| 6543 | **D** | 6540 | 6546 | YKRDAPA | 1.033 |
| 6544 | **A** | 6541 | 6547 | KRDAPAH | 1.011 |
| 6545 | **P** | 6542 | 6548 | RDAPAHI | 0.974 |
| 6546 | **A** | 6543 | 6549 | DAPAHIS | 0.954 |
| 6547 | **H** | 6544 | 6550 | APAHIST | 0.946 |
| 6548 | **I** | 6545 | 6551 | PAHISTI | 0.958 |
| 6549 | **S** | 6546 | 6552 | AHISTIG | 0.98 |
| 6550 | **T** | 6547 | 6553 | HISTIGV | 0.988 |
| 6551 | **I** | 6548 | 6554 | ISTIGVC | 0.978 |
| 6552 | **G** | 6549 | 6555 | STIGVCS | 0.957 |
| 6553 | **V** | 6550 | 6556 | TIGVCSM | 0.935 |
| 6554 | **C** | 6551 | 6557 | IGVCSMT | 0.928 |
| 6555 | **S** | 6552 | 6558 | GVCSMTD | 0.946 |
| 6556 | **M** | 6553 | 6559 | VCSMTDI | 0.966 |
| 6557 | **T** | 6554 | 6560 | CSMTDIA | 0.985 |
| 6558 | **D** | 6555 | 6561 | SMTDIAK | 0.999 |
| 6559 | **I** | 6556 | 6562 | MTDIAKK | 0.999 |
| 6560 | **A** | 6557 | 6563 | TDIAKKP | 1.011 |
| 6561 | **K** | 6558 | 6564 | DIAKKPT | 1.031 |
| 6562 | **K** | 6559 | 6565 | IAKKPTE | 1.05 |
| 6563 | **P** | 6560 | 6566 | AKKPTET | 1.067 |
| 6564 | **T** | 6561 | 6567 | KKPTETI | 1.063 |
| 6565 | **E** | 6562 | 6568 | KPTETIC | 1.041 |
| 6566 | **T** | 6563 | 6569 | PTETICA | 1.01 |
| 6567 | **I** | 6564 | 6570 | TETICAP | 0.974 |
| 6568 | **C** | 6565 | 6571 | ETICAPL | 0.953 |
| 6569 | **A** | 6566 | 6572 | TICAPLT | 0.941 |
| 6570 | **P** | 6567 | 6573 | ICAPLTV | 0.938 |
| 6571 | **L** | 6568 | 6574 | CAPLTVF | 0.941 |
| 6572 | **T** | 6569 | 6575 | APLTVFF | 0.936 |
| 6573 | **V** | 6570 | 6576 | PLTVFFD | 0.939 |
| 6574 | **F** | 6571 | 6577 | LTVFFDG | 0.958 |
| 6575 | **F** | 6572 | 6578 | TVFFDGR | 0.985 |
| 6576 | **D** | 6573 | 6579 | VFFDGRV | 1.019 |
| 6577 | **G** | 6574 | 6580 | FFDGRVD | 1.044 |
| 6578 | **R** | 6575 | 6581 | FDGRVDG | 1.056 |
| 6579 | **V** | 6576 | 6582 | DGRVDGQ | 1.06 |
| 6580 | **D** | 6577 | 6583 | GRVDGQV | 1.06 |
| 6581 | **G** | 6578 | 6584 | RVDGQVD | 1.05 |
| 6582 | **Q** | 6579 | 6585 | VDGQVDL | 1.029 |
| 6583 | **V** | 6580 | 6586 | DGQVDLF | 1.001 |
| 6584 | **D** | 6581 | 6587 | GQVDLFR | 0.975 |
| 6585 | **L** | 6582 | 6588 | QVDLFRN | 0.964 |
| 6586 | **F** | 6583 | 6589 | VDLFRNA | 0.97 |
| 6587 | **R** | 6584 | 6590 | DLFRNAR | 0.989 |
| 6588 | **N** | 6585 | 6591 | LFRNARN | 1.012 |
| 6589 | **A** | 6586 | 6592 | FRNARNG | 1.032 |
| 6590 | **R** | 6587 | 6593 | RNARNGV | 1.04 |
| 6591 | **N** | 6588 | 6594 | NARNGVL | 1.034 |
| 6592 | **G** | 6589 | 6595 | ARNGVLI | 1.008 |
| 6593 | **V** | 6590 | 6596 | RNGVLIT | 0.981 |
| 6594 | **L** | 6591 | 6597 | NGVLITE | 0.971 |
| 6595 | **I** | 6592 | 6598 | GVLITEG | 0.982 |
| 6596 | **T** | 6593 | 6599 | VLITEGS | 1.016 |
| 6597 | **E** | 6594 | 6600 | LITEGSV | 1.046 |
| 6598 | **G** | 6595 | 6601 | ITEGSVK | 1.065 |
| 6599 | **S** | 6596 | 6602 | TEGSVKG | 1.063 |
| 6600 | **V** | 6597 | 6603 | EGSVKGL | 1.047 |
| 6601 | **K** | 6598 | 6604 | GSVKGLQ | 1.038 |
| 6602 | **G** | 6599 | 6605 | SVKGLQP | 1.027 |
| 6603 | **L** | 6600 | 6606 | VKGLQPS | 1.024 |
| 6604 | **Q** | 6601 | 6607 | KGLQPSV | 1.026 |
| 6605 | **P** | 6602 | 6608 | GLQPSVG | 1.027 |
| 6606 | **S** | 6603 | 6609 | LQPSVGP | 1.029 |
| 6607 | **V** | 6604 | 6610 | QPSVGPK | 1.034 |
| 6608 | **G** | 6605 | 6611 | PSVGPKQ | 1.04 |
| 6609 | **P** | 6606 | 6612 | SVGPKQA | 1.046 |
| 6610 | **K** | 6607 | 6613 | VGPKQAS | 1.043 |
| 6611 | **Q** | 6608 | 6614 | GPKQASL | 1.03 |
| 6612 | **A** | 6609 | 6615 | PKQASLN | 1.012 |
| 6613 | **S** | 6610 | 6616 | KQASLNG | 0.995 |
| 6614 | **L** | 6611 | 6617 | QASLNGV | 0.989 |
| 6615 | **N** | 6612 | 6618 | ASLNGVT | 0.99 |
| 6616 | **G** | 6613 | 6619 | SLNGVTL | 0.989 |
| 6617 | **V** | 6614 | 6620 | LNGVTLI | 0.978 |
| 6618 | **T** | 6615 | 6621 | NGVTLIG | 0.968 |
| 6619 | **L** | 6616 | 6622 | GVTLIGE | 0.965 |
| 6620 | **I** | 6617 | 6623 | VTLIGEA | 0.965 |
| 6621 | **G** | 6618 | 6624 | TLIGEAV | 0.977 |
| 6622 | **E** | 6619 | 6625 | LIGEAVK | 0.987 |
| 6623 | **A** | 6620 | 6626 | IGEAVKT | 0.993 |
| 6624 | **V** | 6621 | 6627 | GEAVKTQ | 1.005 |
| 6625 | **K** | 6622 | 6628 | EAVKTQF | 1.015 |
| 6626 | **T** | 6623 | 6629 | AVKTQFN | 1.013 |
| 6627 | **Q** | 6624 | 6630 | VKTQFNY | 1.0 |
| 6628 | **F** | 6625 | 6631 | KTQFNYY | 0.976 |
| 6629 | **N** | 6626 | 6632 | TQFNYYK | 0.961 |
| 6630 | **Y** | 6627 | 6633 | QFNYYKK | 0.965 |
| 6631 | **Y** | 6628 | 6634 | FNYYKKV | 0.981 |
| 6632 | **K** | 6629 | 6635 | NYYKKVD | 1.013 |
| 6633 | **K** | 6630 | 6636 | YYKKVDG | 1.033 |
| 6634 | **V** | 6631 | 6637 | YKKVDGV | 1.034 |
| 6635 | **D** | 6632 | 6638 | KKVDGVV | 1.028 |
| 6636 | **G** | 6633 | 6639 | KVDGVVQ | 1.009 |
| 6637 | **V** | 6634 | 6640 | VDGVVQQ | 0.991 |
| 6638 | **V** | 6635 | 6641 | DGVVQQL | 0.986 |
| 6639 | **Q** | 6636 | 6642 | GVVQQLP | 0.993 |
| 6640 | **Q** | 6637 | 6643 | VVQQLPE | 1.009 |
| 6641 | **L** | 6638 | 6644 | VQQLPET | 1.027 |
| 6642 | **P** | 6639 | 6645 | QQLPETY | 1.04 |
| 6643 | **E** | 6640 | 6646 | QLPETYF | 1.032 |
| 6644 | **T** | 6641 | 6647 | LPETYFT | 1.018 |
| 6645 | **Y** | 6642 | 6648 | PETYFTQ | 1.009 |
| 6646 | **F** | 6643 | 6649 | ETYFTQS | 1.018 |
| 6647 | **T** | 6644 | 6650 | TYFTQSR | 1.045 |
| 6648 | **Q** | 6645 | 6651 | YFTQSRN | 1.072 |
| 6649 | **S** | 6646 | 6652 | FTQSRNL | 1.08 |
| 6650 | **R** | 6647 | 6653 | TQSRNLQ | 1.064 |
| 6651 | **N** | 6648 | 6654 | QSRNLQE | 1.04 |
| 6652 | **L** | 6649 | 6655 | SRNLQEF | 1.014 |
| 6653 | **Q** | 6650 | 6656 | RNLQEFK | 1.007 |
| 6654 | **E** | 6651 | 6657 | NLQEFKP | 1.011 |
| 6655 | **F** | 6652 | 6658 | LQEFKPR | 1.015 |
| 6656 | **K** | 6653 | 6659 | QEFKPRS | 1.039 |
| 6657 | **P** | 6654 | 6660 | EFKPRSQ | 1.053 |
| 6658 | **R** | 6655 | 6661 | FKPRSQM | 1.058 |
| 6659 | **S** | 6656 | 6662 | KPRSQME | 1.056 |
| 6660 | **Q** | 6657 | 6663 | PRSQMEI | 1.029 |
| 6661 | **M** | 6658 | 6664 | RSQMEID | 0.999 |
| 6662 | **E** | 6659 | 6665 | SQMEIDF | 0.974 |
| 6663 | **I** | 6660 | 6666 | QMEIDFL | 0.957 |
| 6664 | **D** | 6661 | 6667 | MEIDFLE | 0.946 |
| 6665 | **F** | 6662 | 6668 | EIDFLEL | 0.943 |
| 6666 | **L** | 6663 | 6669 | IDFLELA | 0.941 |
| 6667 | **E** | 6664 | 6670 | DFLELAM | 0.931 |
| 6668 | **L** | 6665 | 6671 | FLELAMD | 0.935 |
| 6669 | **A** | 6666 | 6672 | LELAMDE | 0.942 |
| 6670 | **M** | 6667 | 6673 | ELAMDEF | 0.952 |
| 6671 | **D** | 6668 | 6674 | LAMDEFI | 0.969 |
| 6672 | **E** | 6669 | 6675 | AMDEFIE | 0.974 |
| 6673 | **F** | 6670 | 6676 | MDEFIER | 0.973 |
| 6674 | **I** | 6671 | 6677 | DEFIERY | 0.974 |
| 6675 | **E** | 6672 | 6678 | EFIERYK | 0.984 |
| 6676 | **R** | 6673 | 6679 | FIERYKL | 0.993 |
| 6677 | **Y** | 6674 | 6680 | IERYKLE | 1.002 |
| 6678 | **K** | 6675 | 6681 | ERYKLEG | 1.014 |
| 6679 | **L** | 6676 | 6682 | RYKLEGY | 1.007 |
| 6680 | **E** | 6677 | 6683 | YKLEGYA | 1.0 |
| 6681 | **G** | 6678 | 6684 | KLEGYAF | 0.985 |
| 6682 | **Y** | 6679 | 6685 | LEGYAFE | 0.957 |
| 6683 | **A** | 6680 | 6686 | EGYAFEH | 0.94 |
| 6684 | **F** | 6681 | 6687 | GYAFEHI | 0.925 |
| 6685 | **E** | 6682 | 6688 | YAFEHIV | 0.918 |
| 6686 | **H** | 6683 | 6689 | AFEHIVY | 0.918 |
| 6687 | **I** | 6684 | 6690 | FEHIVYG | 0.923 |
| 6688 | **V** | 6685 | 6691 | EHIVYGD | 0.942 |
| 6689 | **Y** | 6686 | 6692 | HIVYGDF | 0.962 |
| 6690 | **G** | 6687 | 6693 | IVYGDFS | 0.982 |
| 6691 | **D** | 6688 | 6694 | VYGDFSH | 0.992 |
| 6692 | **F** | 6689 | 6695 | YGDFSHS | 0.986 |
| 6693 | **S** | 6690 | 6696 | GDFSHSQ | 0.986 |
| 6694 | **H** | 6691 | 6697 | DFSHSQL | 0.988 |
| 6695 | **S** | 6692 | 6698 | FSHSQLG | 0.998 |
| 6696 | **Q** | 6693 | 6699 | SHSQLGG | 1.011 |
| 6697 | **L** | 6694 | 6700 | HSQLGGL | 1.013 |
| 6698 | **G** | 6695 | 6701 | SQLGGLH | 1.007 |
| 6699 | **G** | 6696 | 6702 | QLGGLHL | 0.99 |
| 6700 | **L** | 6697 | 6703 | LGGLHLL | 0.967 |
| 6701 | **H** | 6698 | 6704 | GGLHLLI | 0.943 |
| 6702 | **L** | 6699 | 6705 | GLHLLIG | 0.925 |
| 6703 | **L** | 6700 | 6706 | LHLLIGL | 0.917 |
| 6704 | **I** | 6701 | 6707 | HLLIGLA | 0.919 |
| 6705 | **G** | 6702 | 6708 | LLIGLAK | 0.937 |
| 6706 | **L** | 6703 | 6709 | LIGLAKR | 0.959 |
| 6707 | **A** | 6704 | 6710 | IGLAKRF | 0.977 |
| 6708 | **K** | 6705 | 6711 | GLAKRFK | 1.002 |
| 6709 | **R** | 6706 | 6712 | LAKRFKE | 1.016 |
| 6710 | **F** | 6707 | 6713 | AKRFKES | 1.032 |
| 6711 | **K** | 6708 | 6714 | KRFKESP | 1.06 |
| 6712 | **E** | 6709 | 6715 | RFKESPF | 1.07 |
| 6713 | **S** | 6710 | 6716 | FKESPFE | 1.069 |
| 6714 | **P** | 6711 | 6717 | KESPFEL | 1.046 |
| 6715 | **F** | 6712 | 6718 | ESPFELE | 1.011 |
| 6716 | **E** | 6713 | 6719 | SPFELED | 0.995 |
| 6717 | **L** | 6714 | 6720 | PFELEDF | 0.988 |
| 6718 | **E** | 6715 | 6721 | FELEDFI | 0.989 |
| 6719 | **D** | 6716 | 6722 | ELEDFIP | 0.986 |
| 6720 | **F** | 6717 | 6723 | LEDFIPM | 0.965 |
| 6721 | **I** | 6718 | 6724 | EDFIPMD | 0.956 |
| 6722 | **P** | 6719 | 6725 | DFIPMDS | 0.969 |
| 6723 | **M** | 6720 | 6726 | FIPMDST | 0.996 |
| 6724 | **D** | 6721 | 6727 | IPMDSTV | 1.034 |
| 6725 | **S** | 6722 | 6728 | PMDSTVK | 1.06 |
| 6726 | **T** | 6723 | 6729 | MDSTVKN | 1.059 |
| 6727 | **V** | 6724 | 6730 | DSTVKNY | 1.044 |
| 6728 | **K** | 6725 | 6731 | STVKNYF | 1.021 |
| 6729 | **N** | 6726 | 6732 | TVKNYFI | 0.987 |
| 6730 | **Y** | 6727 | 6733 | VKNYFIT | 0.966 |
| 6731 | **F** | 6728 | 6734 | KNYFITD | 0.963 |
| 6732 | **I** | 6729 | 6735 | NYFITDA | 0.972 |
| 6733 | **T** | 6730 | 6736 | YFITDAQ | 1.001 |
| 6734 | **D** | 6731 | 6737 | FITDAQT | 1.029 |
| 6735 | **A** | 6732 | 6738 | ITDAQTG | 1.05 |
| 6736 | **Q** | 6733 | 6739 | TDAQTGS | 1.071 |
| 6737 | **T** | 6734 | 6740 | DAQTGSS | 1.093 |
| 6738 | **G** | 6735 | 6741 | AQTGSSK | 1.113 |
| 6739 | **S** | 6736 | 6742 | QTGSSKC | 1.114 |
| 6740 | **S** | 6737 | 6743 | TGSSKCV | 1.091 |
| 6741 | **K** | 6738 | 6744 | GSSKCVC | 1.041 |
| 6742 | **C** | 6739 | 6745 | SSKCVCS | 0.98 |
| 6743 | **V** | 6740 | 6746 | SKCVCSV | 0.939 |
| 6744 | **C** | 6741 | 6747 | KCVCSVI | 0.913 |
| 6745 | **S** | 6742 | 6748 | CVCSVID | 0.908 |
| 6746 | **V** | 6743 | 6749 | VCSVIDL | 0.915 |
| 6747 | **I** | 6744 | 6750 | CSVIDLL | 0.919 |
| 6748 | **D** | 6745 | 6751 | SVIDLLL | 0.929 |
| 6749 | **L** | 6746 | 6752 | VIDLLLD | 0.945 |
| 6750 | **L** | 6747 | 6753 | IDLLLDD | 0.967 |
| 6751 | **L** | 6748 | 6754 | DLLLDDF | 0.989 |
| 6752 | **D** | 6749 | 6755 | LLLDDFV | 1.004 |
| 6753 | **D** | 6750 | 6756 | LLDDFVE | 0.999 |
| 6754 | **F** | 6751 | 6757 | LDDFVEI | 0.975 |
| 6755 | **V** | 6752 | 6758 | DDFVEII | 0.949 |
| 6756 | **E** | 6753 | 6759 | DFVEIIK | 0.936 |
| 6757 | **I** | 6754 | 6760 | FVEIIKS | 0.946 |
| 6758 | **I** | 6755 | 6761 | VEIIKSQ | 0.987 |
| 6759 | **K** | 6756 | 6762 | EIIKSQD | 1.04 |
| 6760 | **S** | 6757 | 6763 | IIKSQDL | 1.077 |
| 6761 | **Q** | 6758 | 6764 | IKSQDLS | 1.084 |
| 6762 | **D** | 6759 | 6765 | KSQDLSV | 1.059 |
| 6763 | **L** | 6760 | 6766 | SQDLSVV | 1.012 |
| 6764 | **S** | 6761 | 6767 | QDLSVVS | 0.976 |
| 6765 | **V** | 6762 | 6768 | DLSVVSK | 0.966 |
| 6766 | **V** | 6763 | 6769 | LSVVSKV | 0.971 |
| 6767 | **S** | 6764 | 6770 | SVVSKVV | 0.986 |
| 6768 | **K** | 6765 | 6771 | VVSKVVK | 0.997 |
| 6769 | **V** | 6766 | 6772 | VSKVVKV | 0.991 |
| 6770 | **V** | 6767 | 6773 | SKVVKVT | 0.986 |
| 6771 | **K** | 6768 | 6774 | KVVKVTI | 0.985 |
| 6772 | **V** | 6769 | 6775 | VVKVTID | 0.976 |
| 6773 | **T** | 6770 | 6776 | VKVTIDY | 0.972 |
| 6774 | **I** | 6771 | 6777 | KVTIDYT | 0.975 |
| 6775 | **D** | 6772 | 6778 | VTIDYTE | 0.975 |
| 6776 | **Y** | 6773 | 6779 | TIDYTEI | 0.988 |
| 6777 | **T** | 6774 | 6780 | IDYTEIS | 0.999 |
| 6778 | **E** | 6775 | 6781 | DYTEISF | 0.995 |
| 6779 | **I** | 6776 | 6782 | YTEISFM | 0.973 |
| 6780 | **S** | 6777 | 6783 | TEISFML | 0.943 |
| 6781 | **F** | 6778 | 6784 | EISFMLW | 0.907 |
| 6782 | **M** | 6779 | 6785 | ISFMLWC | 0.878 |
| 6783 | **L** | 6780 | 6786 | SFMLWCK | 0.881 |
| 6784 | **W** | 6781 | 6787 | FMLWCKD | 0.895 |
| 6785 | **C** | 6782 | 6788 | MLWCKDG | 0.933 |
| 6786 | **K** | 6783 | 6789 | LWCKDGH | 0.977 |
| 6787 | **D** | 6784 | 6790 | WCKDGHV | 0.993 |
| 6788 | **G** | 6785 | 6791 | CKDGHVE | 1.003 |
| 6789 | **H** | 6786 | 6792 | KDGHVET | 0.999 |
| 6790 | **V** | 6787 | 6793 | DGHVETF | 0.988 |
| 6791 | **E** | 6788 | 6794 | GHVETFY | 0.986 |
| 6792 | **T** | 6789 | 6795 | HVETFYP | 0.987 |
| 6793 | **F** | 6790 | 6796 | VETFYPK | 0.99 |
| 6794 | **Y** | 6791 | 6797 | ETFYPKL | 0.999 |
| 6795 | **P** | 6792 | 6798 | TFYPKLQ | 1.013 |
| 6796 | **K** | 6793 | 6799 | FYPKLQS | 1.03 |
| 6797 | **L** | 6794 | 6800 | YPKLQSS | 1.05 |
| 6798 | **Q** | 6795 | 6801 | PKLQSSQ | 1.071 |
| 6799 | **S** | 6796 | 6802 | KLQSSQA | 1.08 |
| 6800 | **S** | 6797 | 6803 | LQSSQAW | 1.069 |
| 6801 | **Q** | 6798 | 6804 | QSSQAWQ | 1.043 |
| 6802 | **A** | 6799 | 6805 | SSQAWQP | 1.015 |
| 6803 | **W** | 6800 | 6806 | SQAWQPG | 0.998 |
| 6804 | **Q** | 6801 | 6807 | QAWQPGV | 0.997 |
| 6805 | **P** | 6802 | 6808 | AWQPGVA | 0.997 |
| 6806 | **G** | 6803 | 6809 | WQPGVAM | 0.983 |
| 6807 | **V** | 6804 | 6810 | QPGVAMP | 0.966 |
| 6808 | **A** | 6805 | 6811 | PGVAMPN | 0.953 |
| 6809 | **M** | 6806 | 6812 | GVAMPNL | 0.953 |
| 6810 | **P** | 6807 | 6813 | VAMPNLY | 0.969 |
| 6811 | **N** | 6808 | 6814 | AMPNLYK | 0.981 |
| 6812 | **L** | 6809 | 6815 | MPNLYKM | 0.984 |
| 6813 | **Y** | 6810 | 6816 | PNLYKMQ | 0.987 |
| 6814 | **K** | 6811 | 6817 | NLYKMQR | 0.992 |
| 6815 | **M** | 6812 | 6818 | LYKMQRM | 0.986 |
| 6816 | **Q** | 6813 | 6819 | YKMQRML | 0.983 |
| 6817 | **R** | 6814 | 6820 | KMQRMLL | 0.971 |
| 6818 | **M** | 6815 | 6821 | MQRMLLE | 0.954 |
| 6819 | **L** | 6816 | 6822 | QRMLLEK | 0.962 |
| 6820 | **L** | 6817 | 6823 | RMLLEKC | 0.974 |
| 6821 | **E** | 6818 | 6824 | MLLEKCD | 0.989 |
| 6822 | **K** | 6819 | 6825 | LLEKCDL | 0.999 |
| 6823 | **C** | 6820 | 6826 | LEKCDLQ | 0.992 |
| 6824 | **D** | 6821 | 6827 | EKCDLQN | 0.986 |
| 6825 | **L** | 6822 | 6828 | KCDLQNY | 0.983 |
| 6826 | **Q** | 6823 | 6829 | CDLQNYG | 0.989 |
| 6827 | **N** | 6824 | 6830 | DLQNYGD | 0.998 |
| 6828 | **Y** | 6825 | 6831 | LQNYGDS | 1.008 |
| 6829 | **G** | 6826 | 6832 | QNYGDSA | 1.02 |
| 6830 | **D** | 6827 | 6833 | NYGDSAT | 1.022 |
| 6831 | **S** | 6828 | 6834 | YGDSATL | 1.018 |
| 6832 | **A** | 6829 | 6835 | GDSATLP | 1.015 |
| 6833 | **T** | 6830 | 6836 | DSATLPK | 1.01 |
| 6834 | **L** | 6831 | 6837 | SATLPKG | 1.018 |
| 6835 | **P** | 6832 | 6838 | ATLPKGI | 1.025 |
| 6836 | **K** | 6833 | 6839 | TLPKGIM | 1.013 |
| 6837 | **G** | 6834 | 6840 | LPKGIMM | 0.983 |
| 6838 | **I** | 6835 | 6841 | PKGIMMN | 0.939 |
| 6839 | **M** | 6836 | 6842 | KGIMMNV | 0.903 |
| 6840 | **M** | 6837 | 6843 | GIMMNVA | 0.892 |
| 6841 | **N** | 6838 | 6844 | IMMNVAK | 0.909 |
| 6842 | **V** | 6839 | 6845 | MMNVAKY | 0.934 |
| 6843 | **A** | 6840 | 6846 | MNVAKYT | 0.964 |
| 6844 | **K** | 6841 | 6847 | NVAKYTQ | 0.991 |
| 6845 | **Y** | 6842 | 6848 | VAKYTQL | 1.0 |
| 6846 | **T** | 6843 | 6849 | AKYTQLC | 1.002 |
| 6847 | **Q** | 6844 | 6850 | KYTQLCQ | 0.985 |
| 6848 | **L** | 6845 | 6851 | YTQLCQY | 0.958 |
| 6849 | **C** | 6846 | 6852 | TQLCQYL | 0.936 |
| 6850 | **Q** | 6847 | 6853 | QLCQYLN | 0.928 |
| 6851 | **Y** | 6848 | 6854 | LCQYLNT | 0.94 |
| 6852 | **L** | 6849 | 6855 | CQYLNTL | 0.961 |
| 6853 | **N** | 6850 | 6856 | QYLNTLT | 0.98 |
| 6854 | **T** | 6851 | 6857 | YLNTLTL | 0.988 |
| 6855 | **L** | 6852 | 6858 | LNTLTLA | 0.976 |
| 6856 | **T** | 6853 | 6859 | NTLTLAV | 0.959 |
| 6857 | **L** | 6854 | 6860 | TLTLAVP | 0.943 |
| 6858 | **A** | 6855 | 6861 | LTLAVPY | 0.931 |
| 6859 | **V** | 6856 | 6862 | TLAVPYN | 0.931 |
| 6860 | **P** | 6857 | 6863 | LAVPYNM | 0.934 |
| 6861 | **Y** | 6858 | 6864 | AVPYNMR | 0.936 |
| 6862 | **N** | 6859 | 6865 | VPYNMRV | 0.935 |
| 6863 | **M** | 6860 | 6866 | PYNMRVI | 0.929 |
| 6864 | **R** | 6861 | 6867 | YNMRVIH | 0.918 |
| 6865 | **V** | 6862 | 6868 | NMRVIHF | 0.909 |
| 6866 | **I** | 6863 | 6869 | MRVIHFG | 0.903 |
| 6867 | **H** | 6864 | 6870 | RVIHFGA | 0.911 |
| 6868 | **F** | 6865 | 6871 | VIHFGAG | 0.931 |
| 6869 | **G** | 6866 | 6872 | IHFGAGS | 0.967 |
| 6870 | **A** | 6867 | 6873 | HFGAGSD | 1.009 |
| 6871 | **G** | 6868 | 6874 | FGAGSDK | 1.045 |
| 6872 | **S** | 6869 | 6875 | GAGSDKG | 1.071 |
| 6873 | **D** | 6870 | 6876 | AGSDKGV | 1.066 |
| 6874 | **K** | 6871 | 6877 | GSDKGVA | 1.049 |
| 6875 | **G** | 6872 | 6878 | SDKGVAP | 1.027 |
| 6876 | **V** | 6873 | 6879 | DKGVAPG | 1.013 |
| 6877 | **A** | 6874 | 6880 | KGVAPGT | 1.021 |
| 6878 | **P** | 6875 | 6881 | GVAPGTA | 1.034 |
| 6879 | **G** | 6876 | 6882 | VAPGTAV | 1.037 |
| 6880 | **T** | 6877 | 6883 | APGTAVL | 1.023 |
| 6881 | **A** | 6878 | 6884 | PGTAVLR | 1.0 |
| 6882 | **V** | 6879 | 6885 | GTAVLRQ | 0.981 |
| 6883 | **L** | 6880 | 6886 | TAVLRQW | 0.974 |
| 6884 | **R** | 6881 | 6887 | AVLRQWL | 0.978 |
| 6885 | **Q** | 6882 | 6888 | VLRQWLP | 0.987 |
| 6886 | **W** | 6883 | 6889 | LRQWLPT | 0.993 |
| 6887 | **L** | 6884 | 6890 | RQWLPTG | 1.014 |
| 6888 | **P** | 6885 | 6891 | QWLPTGT | 1.04 |
| 6889 | **T** | 6886 | 6892 | WLPTGTL | 1.055 |
| 6890 | **G** | 6887 | 6893 | LPTGTLL | 1.057 |
| 6891 | **T** | 6888 | 6894 | PTGTLLV | 1.032 |
| 6892 | **L** | 6889 | 6895 | TGTLLVD | 1.004 |
| 6893 | **L** | 6890 | 6896 | GTLLVDS | 0.996 |
| 6894 | **V** | 6891 | 6897 | TLLVDSD | 1.009 |
| 6895 | **D** | 6892 | 6898 | LLVDSDL | 1.037 |
| 6896 | **S** | 6893 | 6899 | LVDSDLN | 1.058 |
| 6897 | **D** | 6894 | 6900 | VDSDLND | 1.061 |
| 6898 | **L** | 6895 | 6901 | DSDLNDF | 1.042 |
| 6899 | **N** | 6896 | 6902 | SDLNDFV | 1.018 |
| 6900 | **D** | 6897 | 6903 | DLNDFVS | 1.002 |
| 6901 | **F** | 6898 | 6904 | LNDFVSD | 0.991 |
| 6902 | **V** | 6899 | 6905 | NDFVSDA | 0.999 |
| 6903 | **S** | 6900 | 6906 | DFVSDAD | 1.02 |
| 6904 | **D** | 6901 | 6907 | FVSDADS | 1.046 |
| 6905 | **A** | 6902 | 6908 | VSDADST | 1.069 |
| 6906 | **D** | 6903 | 6909 | SDADSTL | 1.08 |
| 6907 | **S** | 6904 | 6910 | DADSTLI | 1.068 |
| 6908 | **T** | 6905 | 6911 | ADSTLIG | 1.04 |
| 6909 | **L** | 6906 | 6912 | DSTLIGD | 1.017 |
| 6910 | **I** | 6907 | 6913 | STLIGDC | 0.994 |
| 6911 | **G** | 6908 | 6914 | TLIGDCA | 0.987 |
| 6912 | **D** | 6909 | 6915 | LIGDCAT | 0.98 |
| 6913 | **C** | 6910 | 6916 | IGDCATV | 0.962 |
| 6914 | **A** | 6911 | 6917 | GDCATVH | 0.953 |
| 6915 | **T** | 6912 | 6918 | DCATVHT | 0.94 |
| 6916 | **V** | 6913 | 6919 | CATVHTA | 0.94 |
| 6917 | **H** | 6914 | 6920 | ATVHTAN | 0.956 |
| 6918 | **T** | 6915 | 6921 | TVHTANK | 0.975 |
| 6919 | **A** | 6916 | 6922 | VHTANKW | 0.994 |
| 6920 | **N** | 6917 | 6923 | HTANKWD | 1.0 |
| 6921 | **K** | 6918 | 6924 | TANKWDL | 0.998 |
| 6922 | **W** | 6919 | 6925 | ANKWDLI | 0.974 |
| 6923 | **D** | 6920 | 6926 | NKWDLII | 0.95 |
| 6924 | **L** | 6921 | 6927 | KWDLIIS | 0.939 |
| 6925 | **I** | 6922 | 6928 | WDLIISD | 0.939 |
| 6926 | **I** | 6923 | 6929 | DLIISDM | 0.951 |
| 6927 | **S** | 6924 | 6930 | LIISDMY | 0.968 |
| 6928 | **D** | 6925 | 6931 | IISDMYD | 0.981 |
| 6929 | **M** | 6926 | 6932 | ISDMYDP | 0.983 |
| 6930 | **Y** | 6927 | 6933 | SDMYDPK | 1.0 |
| 6931 | **D** | 6928 | 6934 | DMYDPKT | 1.024 |
| 6932 | **P** | 6929 | 6935 | MYDPKTK | 1.045 |
| 6933 | **K** | 6930 | 6936 | YDPKTKN | 1.066 |
| 6934 | **T** | 6931 | 6937 | DPKTKNV | 1.065 |
| 6935 | **K** | 6932 | 6938 | PKTKNVT | 1.054 |
| 6936 | **N** | 6933 | 6939 | KTKNVTK | 1.043 |
| 6937 | **V** | 6934 | 6940 | TKNVTKE | 1.04 |
| 6938 | **T** | 6935 | 6941 | KNVTKEN | 1.052 |
| 6939 | **K** | 6936 | 6942 | NVTKEND | 1.065 |
| 6940 | **E** | 6937 | 6943 | VTKENDS | 1.083 |
| 6941 | **N** | 6938 | 6944 | TKENDSK | 1.095 |
| 6942 | **D** | 6939 | 6945 | KENDSKE | 1.097 |
| 6943 | **S** | 6940 | 6946 | ENDSKEG | 1.101 |
| 6944 | **K** | 6941 | 6947 | NDSKEGF | 1.084 |
| 6945 | **E** | 6942 | 6948 | DSKEGFF | 1.056 |
| 6946 | **G** | 6943 | 6949 | SKEGFFT | 1.019 |
| 6947 | **F** | 6944 | 6950 | KEGFFTY | 0.974 |
| 6948 | **F** | 6945 | 6951 | EGFFTYI | 0.943 |
| 6949 | **T** | 6946 | 6952 | GFFTYIC | 0.922 |
| 6950 | **Y** | 6947 | 6953 | FFTYICG | 0.91 |
| 6951 | **I** | 6948 | 6954 | FTYICGF | 0.903 |
| 6952 | **C** | 6949 | 6955 | TYICGFI | 0.9 |
| 6953 | **G** | 6950 | 6956 | YICGFIQ | 0.909 |
| 6954 | **F** | 6951 | 6957 | ICGFIQQ | 0.934 |
| 6955 | **I** | 6952 | 6958 | CGFIQQK | 0.97 |
| 6956 | **Q** | 6953 | 6959 | GFIQQKL | 1.01 |
| 6957 | **Q** | 6954 | 6960 | FIQQKLA | 1.031 |
| 6958 | **K** | 6955 | 6961 | IQQKLAL | 1.025 |
| 6959 | **L** | 6956 | 6962 | QQKLALG | 1.006 |
| 6960 | **A** | 6957 | 6963 | QKLALGG | 0.993 |
| 6961 | **L** | 6958 | 6964 | KLALGGS | 0.999 |
| 6962 | **G** | 6959 | 6965 | LALGGSV | 1.015 |
| 6963 | **G** | 6960 | 6966 | ALGGSVA | 1.025 |
| 6964 | **S** | 6961 | 6967 | LGGSVAI | 1.008 |
| 6965 | **V** | 6962 | 6968 | GGSVAIK | 0.981 |
| 6966 | **A** | 6963 | 6969 | GSVAIKI | 0.961 |
| 6967 | **I** | 6964 | 6970 | SVAIKIT | 0.961 |
| 6968 | **K** | 6965 | 6971 | VAIKITE | 0.985 |
| 6969 | **I** | 6966 | 6972 | AIKITEH | 1.004 |
| 6970 | **T** | 6967 | 6973 | IKITEHS | 1.013 |
| 6971 | **E** | 6968 | 6974 | KITEHSW | 1.005 |
| 6972 | **H** | 6969 | 6975 | ITEHSWN | 0.981 |
| 6973 | **S** | 6970 | 6976 | TEHSWNA | 0.965 |
| 6974 | **W** | 6971 | 6977 | EHSWNAD | 0.955 |
| 6975 | **N** | 6972 | 6978 | HSWNADL | 0.954 |
| 6976 | **A** | 6973 | 6979 | SWNADLY | 0.961 |
| 6977 | **D** | 6974 | 6980 | WNADLYK | 0.965 |
| 6978 | **L** | 6975 | 6981 | NADLYKL | 0.97 |
| 6979 | **Y** | 6976 | 6982 | ADLYKLM | 0.966 |
| 6980 | **K** | 6977 | 6983 | DLYKLMG | 0.963 |
| 6981 | **L** | 6978 | 6984 | LYKLMGH | 0.951 |
| 6982 | **M** | 6979 | 6985 | YKLMGHF | 0.935 |
| 6983 | **G** | 6980 | 6986 | KLMGHFA | 0.927 |
| 6984 | **H** | 6981 | 6987 | LMGHFAW | 0.912 |
| 6985 | **F** | 6982 | 6988 | MGHFAWW | 0.901 |
| 6986 | **A** | 6983 | 6989 | GHFAWWT | 0.895 |
| 6987 | **W** | 6984 | 6990 | HFAWWTA | 0.89 |
| 6988 | **W** | 6985 | 6991 | FAWWTAF | 0.899 |
| 6989 | **T** | 6986 | 6992 | AWWTAFV | 0.911 |
| 6990 | **A** | 6987 | 6993 | WWTAFVT | 0.929 |
| 6991 | **F** | 6988 | 6994 | WTAFVTN | 0.948 |
| 6992 | **V** | 6989 | 6995 | TAFVTNV | 0.964 |
| 6993 | **T** | 6990 | 6996 | AFVTNVN | 0.979 |
| 6994 | **N** | 6991 | 6997 | FVTNVNA | 0.987 |
| 6995 | **V** | 6992 | 6998 | VTNVNAS | 0.993 |
| 6996 | **N** | 6993 | 6999 | TNVNASS | 1.007 |
| 6997 | **A** | 6994 | 7000 | NVNASSS | 1.036 |
| 6998 | **S** | 6995 | 7001 | VNASSSE | 1.065 |
| 6999 | **S** | 6996 | 7002 | NASSSEA | 1.085 |
| 7000 | **S** | 6997 | 7003 | ASSSEAF | 1.077 |
| 7001 | **E** | 6998 | 7004 | SSSEAFL | 1.039 |
| 7002 | **A** | 6999 | 7005 | SSEAFLI | 0.992 |
| 7003 | **F** | 7000 | 7006 | SEAFLIG | 0.95 |
| 7004 | **L** | 7001 | 7007 | EAFLIGC | 0.927 |
| 7005 | **I** | 7002 | 7008 | AFLIGCN | 0.92 |
| 7006 | **G** | 7003 | 7009 | FLIGCNY | 0.925 |
| 7007 | **C** | 7004 | 7010 | LIGCNYL | 0.933 |
| 7008 | **N** | 7005 | 7011 | IGCNYLG | 0.943 |
| 7009 | **Y** | 7006 | 7012 | GCNYLGK | 0.963 |
| 7010 | **L** | 7007 | 7013 | CNYLGKP | 0.989 |
| 7011 | **G** | 7008 | 7014 | NYLGKPR | 1.017 |
| 7012 | **K** | 7009 | 7015 | YLGKPRE | 1.043 |
| 7013 | **P** | 7010 | 7016 | LGKPREQ | 1.055 |
| 7014 | **R** | 7011 | 7017 | GKPREQI | 1.055 |
| 7015 | **E** | 7012 | 7018 | KPREQID | 1.054 |
| 7016 | **Q** | 7013 | 7019 | PREQIDG | 1.047 |
| 7017 | **I** | 7014 | 7020 | REQIDGY | 1.037 |
| 7018 | **D** | 7015 | 7021 | EQIDGYV | 1.026 |
| 7019 | **G** | 7016 | 7022 | QIDGYVM | 0.993 |
| 7020 | **Y** | 7017 | 7023 | IDGYVMH | 0.954 |
| 7021 | **V** | 7018 | 7024 | DGYVMHA | 0.923 |
| 7022 | **M** | 7019 | 7025 | GYVMHAN | 0.898 |
| 7023 | **H** | 7020 | 7026 | YVMHANY | 0.898 |
| 7024 | **A** | 7021 | 7027 | VMHANYI | 0.907 |
| 7025 | **N** | 7022 | 7028 | MHANYIF | 0.912 |
| 7026 | **Y** | 7023 | 7029 | HANYIFW | 0.916 |
| 7027 | **I** | 7024 | 7030 | ANYIFWR | 0.918 |
| 7028 | **F** | 7025 | 7031 | NYIFWRN | 0.937 |
| 7029 | **W** | 7026 | 7032 | YIFWRNT | 0.967 |
| 7030 | **R** | 7027 | 7033 | IFWRNTN | 1.011 |
| 7031 | **N** | 7028 | 7034 | FWRNTNP | 1.052 |
| 7032 | **T** | 7029 | 7035 | WRNTNPI | 1.071 |
| 7033 | **N** | 7030 | 7036 | RNTNPIQ | 1.068 |
| 7034 | **P** | 7031 | 7037 | NTNPIQL | 1.044 |
| 7035 | **I** | 7032 | 7038 | TNPIQLS | 1.013 |
| 7036 | **Q** | 7033 | 7039 | NPIQLSS | 0.992 |
| 7037 | **L** | 7034 | 7040 | PIQLSSY | 0.988 |
| 7038 | **S** | 7035 | 7041 | IQLSSYS | 0.991 |
| 7039 | **S** | 7036 | 7042 | QLSSYSL | 0.99 |
| 7040 | **Y** | 7037 | 7043 | LSSYSLF | 0.978 |
| 7041 | **S** | 7038 | 7044 | SSYSLFD | 0.96 |
| 7042 | **L** | 7039 | 7045 | SYSLFDM | 0.946 |
| 7043 | **F** | 7040 | 7046 | YSLFDMS | 0.942 |
| 7044 | **D** | 7041 | 7047 | SLFDMSK | 0.958 |
| 7045 | **M** | 7042 | 7048 | LFDMSKF | 0.975 |
| 7046 | **S** | 7043 | 7049 | FDMSKFP | 0.99 |
| 7047 | **K** | 7044 | 7050 | DMSKFPL | 0.995 |
| 7048 | **F** | 7045 | 7051 | MSKFPLK | 0.987 |
| 7049 | **P** | 7046 | 7052 | SKFPLKL | 0.982 |
| 7050 | **L** | 7047 | 7053 | KFPLKLR | 0.984 |
| 7051 | **K** | 7048 | 7054 | FPLKLRG | 1.001 |
| 7052 | **L** | 7049 | 7055 | PLKLRGT | 1.02 |
| 7053 | **R** | 7050 | 7056 | LKLRGTA | 1.036 |
| 7054 | **G** | 7051 | 7057 | KLRGTAV | 1.038 |
| 7055 | **T** | 7052 | 7058 | LRGTAVM | 1.011 |
| 7056 | **A** | 7053 | 7059 | RGTAVMS | 0.977 |
| 7057 | **V** | 7054 | 7060 | GTAVMSL | 0.946 |
| 7058 | **M** | 7055 | 7061 | TAVMSLK | 0.932 |
| 7059 | **S** | 7056 | 7062 | AVMSLKE | 0.951 |
| 7060 | **L** | 7057 | 7063 | VMSLKEG | 0.991 |
| 7061 | **K** | 7058 | 7064 | MSLKEGQ | 1.033 |
| 7062 | **E** | 7059 | 7065 | SLKEGQI | 1.06 |
| 7063 | **G** | 7060 | 7066 | LKEGQIN | 1.067 |
| 7064 | **Q** | 7061 | 7067 | KEGQIND | 1.057 |
| 7065 | **I** | 7062 | 7068 | EGQINDM | 1.033 |
| 7066 | **N** | 7063 | 7069 | GQINDMI | 1.006 |
| 7067 | **D** | 7064 | 7070 | QINDMIL | 0.981 |
| 7068 | **M** | 7065 | 7071 | INDMILS | 0.949 |
| 7069 | **I** | 7066 | 7072 | NDMILSL | 0.934 |
| 7070 | **L** | 7067 | 7073 | DMILSLL | 0.933 |
| 7071 | **S** | 7068 | 7074 | MILSLLS | 0.94 |
| 7072 | **L** | 7069 | 7075 | ILSLLSK | 0.967 |
| 7073 | **L** | 7070 | 7076 | LSLLSKG | 1.0 |
| 7074 | **S** | 7071 | 7077 | SLLSKGR | 1.032 |
| 7075 | **K** | 7072 | 7078 | LLSKGRL | 1.053 |
| 7076 | **G** | 7073 | 7079 | LSKGRLI | 1.049 |
| 7077 | **R** | 7074 | 7080 | SKGRLII | 1.018 |
| 7078 | **L** | 7075 | 7081 | KGRLIIR | 0.983 |
| 7079 | **I** | 7076 | 7082 | GRLIIRE | 0.962 |
| 7080 | **I** | 7077 | 7083 | RLIIREN | 0.97 |
| 7081 | **R** | 7078 | 7084 | LIIRENN | 1.008 |
| 7082 | **E** | 7079 | 7085 | IIRENNR | 1.046 |
| 7083 | **N** | 7080 | 7086 | IRENNRV | 1.065 |
| 7084 | **N** | 7081 | 7087 | RENNRVV | 1.055 |
| 7085 | **R** | 7082 | 7088 | ENNRVVI | 1.018 |
| 7086 | **V** | 7083 | 7089 | NNRVVIS | 0.982 |
| 7087 | **V** | 7084 | 7090 | NRVVISS | 0.972 |
| 7088 | **I** | 7085 | 7091 | RVVISSD | 0.985 |
| 7089 | **S** | 7086 | 7092 | VVISSDV | 1.015 |
| 7090 | **S** | 7087 | 7093 | VISSDVL | 1.035 |
| 7091 | **D** | 7088 | 7094 | ISSDVLV | 1.025 |
| 7092 | **V** | 7089 | 7095 | SSDVLVN | 0.999 |
