# Supplementary figures and images for: Elucidation of novel compounds and epitope-based peptide vaccine design against C30 endopeptidase regions of SARS-CoV-2 using immunoinformatics approaches
Source: Front Cell Infect Microbiol. 2023 May 24;13:1134802. doi: 10.3389/fcimb.2023.1134802 (PMC10244718; doi:10.3389/fcimb.2023.1134802)

**Population coverage in China**


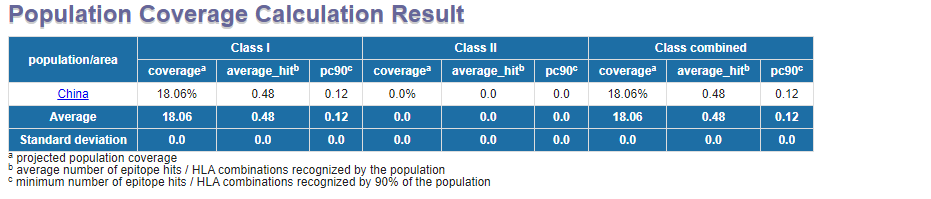


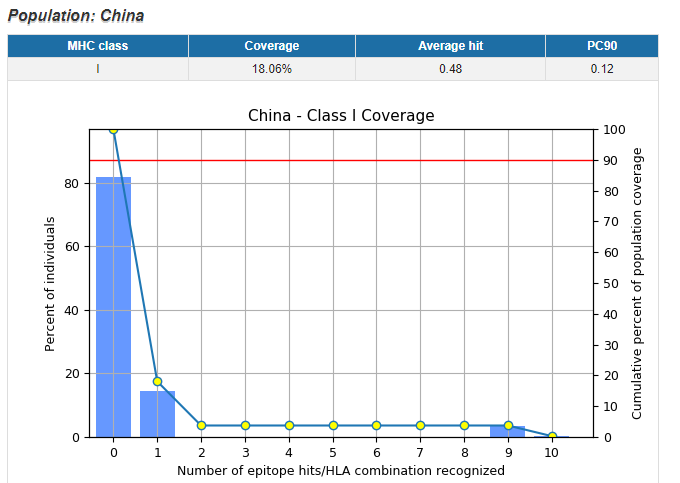


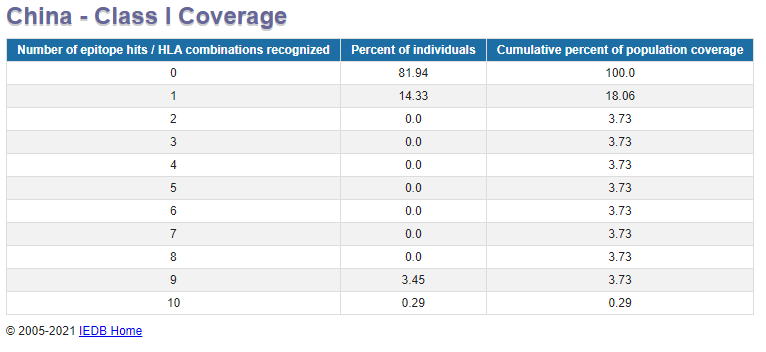


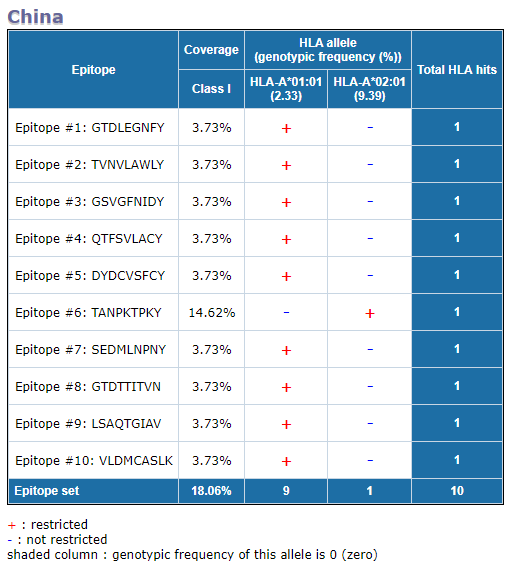


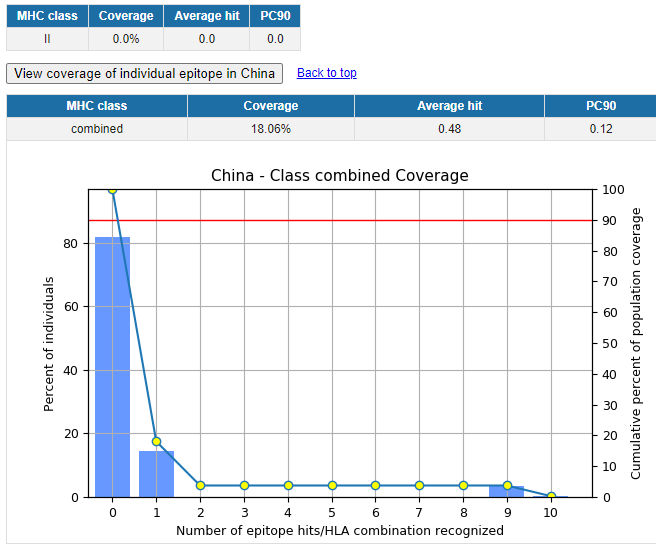


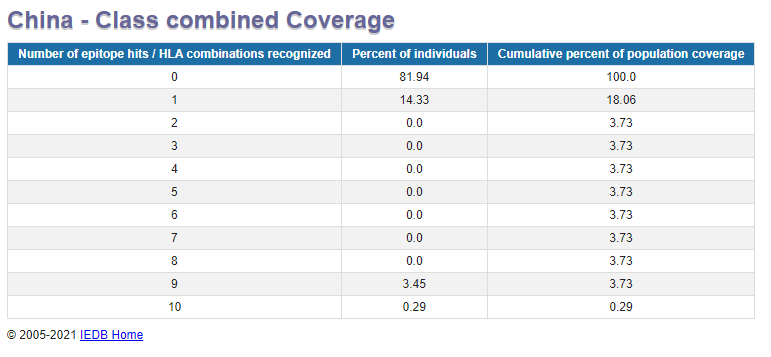


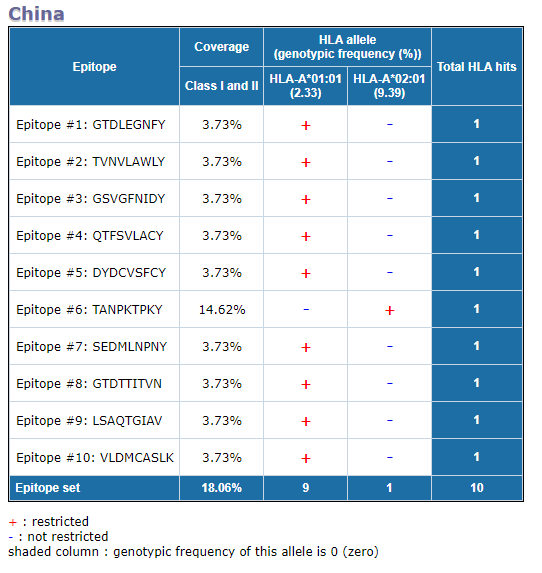


**Population coverage in Italy**


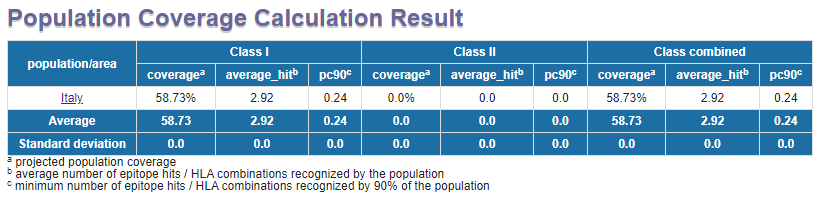


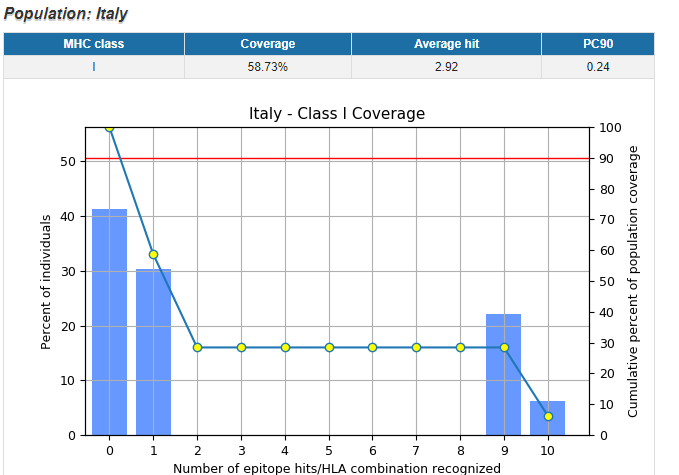

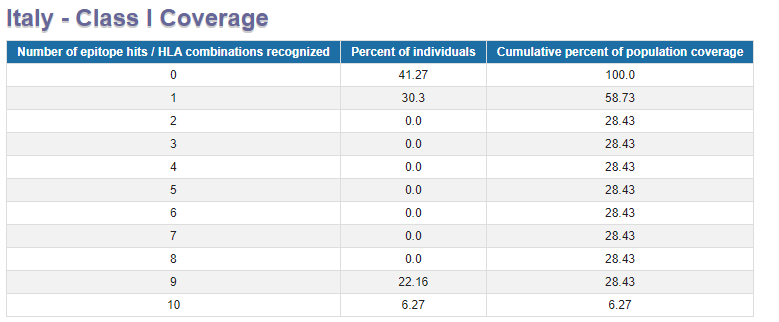


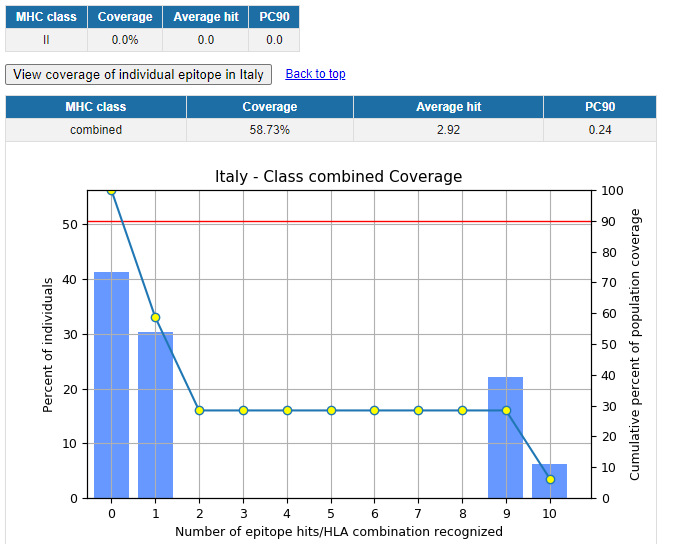


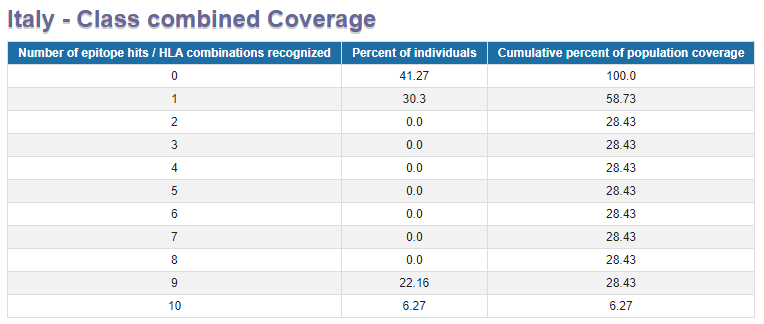


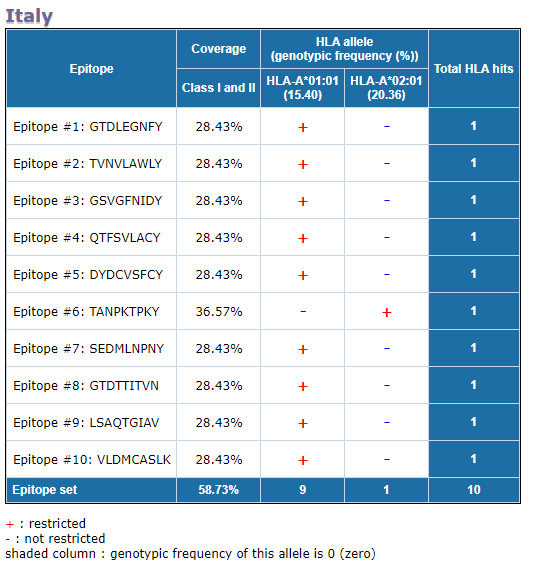

Supplement: Supplementary file 1 [file DataSheet_1.zip › S5 Population coverage.docx]
